# Development and Application of Small Molecule–Peptide Conjugates as Cathepsin K-Specific Covalent Irreversible Inhibitors in Human Osteoclast and Lung Cancer

**DOI:** 10.1021/jacsau.4c00840

**Published:** 2025-03-03

**Authors:** Gourab Dey, Evalyn Yakobovich, Jure Loboda, Reut Sinai-Turyansky, Chen Abramovitch-Dahan, Emmanuelle Merquiol, Nikhila Sridharan, Gal Itzhak, Boris Turk, Ori Wald, Dusan Turk, Simon Yona, Noam Levaot, Galia Blum

**Affiliations:** † The Institute for Drug Research, The School of Pharmacy, The Faculty of Medicine, The Hebrew University, Jerusalem 9112001, Israel; ‡ Department of Biochemistry and Molecular Biology, J. Stefan Institute, Jamova 39, SI-1000 Ljubljana, Sloveni; § Department of Physiology and Cell Biology Faculty of Health Sciences, Ben-Gurion University of the Negev, Shderot Ben Gurion 1, Beer-Sheva 844394, Israel; ∥ The Institute of Biomedical and Oral Research, The Faculty of Dental Medicine, The Hebrew University of Jerusalem, Jerusalem 9112001, Israel; ⊥ Department of Cardiothoracic Surgery, Hadassah Hebrew University Medical Center, The Faculty of Medicine, The Hebrew University of Jerusalem, Jerusalem 9112001, Israel

**Keywords:** cathepsin K, covalent irreversible inhibitors, small molecule–peptide conjugates, protease inhibitors, osteoclasts

## Abstract

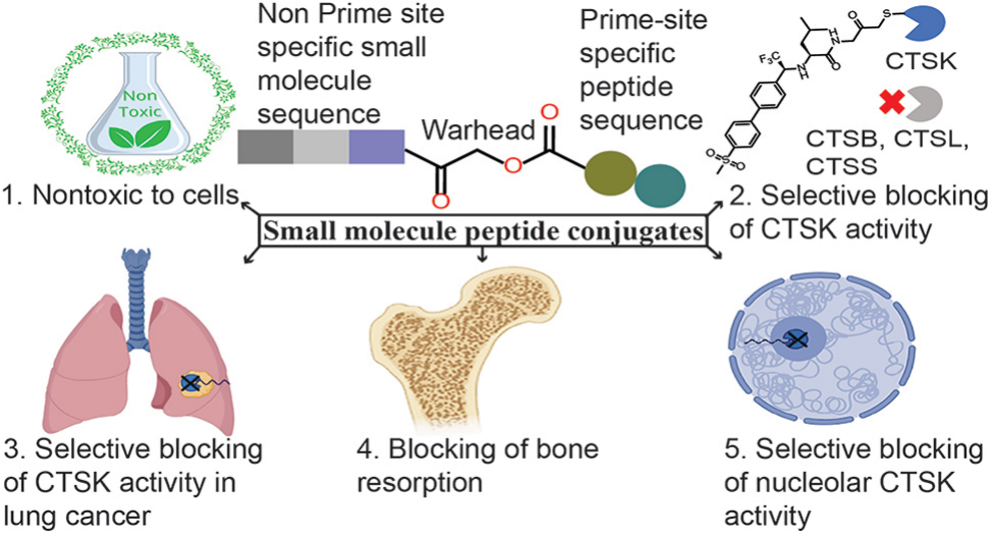

Cathepsin K (CTSK), a proteolytic enzyme that degrades the extracellular matrix, is recognized as a significant therapeutic target for osteoporosis, osteoarthritis, and rheumatoid arthritis. Due to adverse effects, no clinically approved drugs exist for CTSK. In order to develop safer therapeutics, highly selective CTSK inhibitors are required to elucidate the origins of side effects. Here, we developed various hybrid inhibitors by combining peptide sequences with small organic molecules. An acyloxymethyl ketone electrophile was incorporated as a bioisostere of the glycine–glycine cleavage site and inverse peptide sequences to enhance prime site interactions, as seen in the crystal structure. Additionally, a diphenyl group was incorporated to improve nonprime site interactions, culminating in highly selective and potent irreversible CTSK inhibitors with negligible off-target binding by closely related cathepsins. These novel inhibitors were also designed to attach to targeting moieties, further reducing off-target effects in vivo. Our findings demonstrate that these highly selective inhibitors are nontoxic, effectively inhibit bone resorption by human osteoclasts, block CTSK activity in cells and their nuclei, and inhibit activity in human lung cancer tissue. This study highlights significant advancements in designing CTSK inhibitors with potential clinical applications for lung cancer and osteoclast-related conditions.

## Introduction

The major functions of cysteine cathepsin enzymes are protein turnover and processing.^1^ While five members of the cysteine cathepsin family are ubiquitously expressed, the rest are found only in certain cell types and tissues.^2^ Of the latter, cathepsin K (CTSK) is largely expressed in osteoclasts and is a key player in bone remodeling by degrading endogenous proteins such as type I collagen, elastin, and gelatin, which are the bone building blocks.^3^

The bone is an inflexible yet adaptable organ that undergoes constant remodeling, shaping, and restoration. Bone remodeling is the primary metabolic process that governs the shape and function of bones in adulthood, in which osteoclasts play a prominent role. Osteoclast precursors (OCPs) are attracted to locations on bone surfaces intended for resorption. Upon arrival, the OCPs fuse to each other, forming multinucleated cells that are responsible for the resorption of calcified matrices. Alongside this, osteoblasts deposit type I collagen and other proteins into resorbed voids, effectively forming and mineralizing bone. Any disruption in the fine balance between bone deposition and resorption may lead to a wide range of bone disorders, including osteoporosis, rheumatoid arthritis, periodontitis, and bone metastases.^4−8^ Osteoporosis is a condition that causes a gradual decrease of bone mass, leading to an increased risk of fracture.^9^ The molecular knowledge of bone cell activity and osteoclast functions has opened the way for the development of many bone disease prevention therapies, including CTSK inhibitors.^10^ This interest stems from the unusual ability of CTSK inhibitors to reduce signs of bone resorption without impacting markers of bone growth.^11^

CTSK is found in specific tissues in the body; besides bone remodeling, it also regulates various cellular activities.^3,12,13^ A recent study suggests that the presence of CTSK is of utmost importance for the growth and functioning of the central nervous system.^14^ A strong correlation between elevated levels of CTSK in the blood and the occurrence of chronic heart failure was also reported.^15^ In addition to its involvement in cardiac failure, CTSK has been associated with the development of several cardiovascular ailments, such as abdominal aortic aneurysms, coronary artery diseases, and atherosclerosis.^15^ Moreover, CTSK expression patterns vary between lung cancer cells and stromal cells, indicating the potential prognostic relevance of this protease.^16^ CTSK is involved in the proliferation, migration, and invasion of nonsmall cell lung cancer cells, where it most likely facilitates the mTOR signaling pathway, suggesting options for novel lung cancer treatment.^16^ Recently, it has been discovered that CTSK acts as an epigenetic regulator in the nucleus for osteoclast activation.^17^ However, the specific regulatory systems governing the transportation of CTSK into the nucleus, as well as the subsequent biological consequences, have not been further investigated, unlike other cathepsins, even though their activity was detected in the nucleus.^18,19^ CTSK participation in other cellular activities highlights its potential as a therapeutic target for illnesses and ailments over and above bone-related disorders. Therefore, chemical tools that influence CTSK activity may be useful for elucidating these functions and can be used as an investigative tool.

Merck created a CTSK-specific covalent reversible inhibitor, odanacatib (ODN), intended to treat osteoporosis patients with high expression of this enzyme.^20,21^ It was pulled from the trail by Merck due to safety concerns.^22,23^ Other reported CTSK inhibitors are primarily based on a unique small chemical sequence, although most have not been verified in a biological context or explored further due to limited selectivity and an undisclosed distribution profile in the body.^24,25^ Still, there remains a high demand for new CTSK-specific inhibitors that block the enzyme in osteoclasts without interfering with its function in other tissues and organs.

Covalent irreversible binding of inhibitors to their target enzyme has shown various advantages, including a longer duration of action, reduced pharmacokinetic sensitivity, and the possibility of increased effectiveness despite tiny, shallow binding pockets.^26−30^ The use of these favorable features has resulted in the development of numerous FDA-approved covalent medications. However, covalent irreversible modifications to off-target proteins in vivo may raise immunogenicity and possible toxicity concerns.^26^ Therefore, it is essential to generate extremely selective and potent irreversible inhibitors to reduce off-target binding.

In modern pharmaceuticals, FDA-approved afatinib, ibrutinib, osimertinib, and neratinib employ acrylamides and acrylate moieties to specifically interact with the high nucleophilic thiol residue of cysteine within their targets.^31^ The cysteine thiol can undergo covalent alteration by forming Michael adducts with these electrophiles (termed warheads). In addition, several electrophiles such as diazo- and fluoromethyl ketones, vinyl sulfones, epoxides, and acyloxymethyl ketones (AOMKs) have been incorporated in inhibitors to target cysteine specifically.^32−38^ Among these, the AOMKs have a weak leaving group; they exhibit notable selectivity toward cysteine proteases in proteomes when they are positioned near the active-site cysteine, which can be achieved with careful design of the inhibitor.^35^ Recently, AOMK-based CTSK inhibitors and probes were reported to show high selectivity over other cathepsins, excluding CTSB.^35^

The X-ray structures of CTSK with its substrate infer its strong binding to both the nonprime (S) and the prime (S′) binding pockets.^36^ Most inhibitors of other cysteine cathepsins strongly bind with the S binding pocket, which is sufficient to generate strong binding inhibitors. However, our unique approach is to generate CTSK-specific covalent irreversible inhibitors that occupy both the S and S′ pockets, increasing both potency and selectivity toward CTSK.

Here, we generated and investigated a library of CTSK inhibitors that mainly include AOMK and phenoxymethyl ketone (PMK) as electrophilic warheads, inspired by previously published cysteine protease inhibitors.^37,38^ The inhibitor design combined a small molecule part with a peptidic part, generating tight binding to both the nonprime and the prime site of CTSK with extremely high efficacy, potency, and selectivity. Structure–activity relationship (SAR) studies offer important insights into this design principle and optimization of the inhibitors. The best two selective covalent irreversible compounds, **GD20** and **GD38**, include an AOMK warhead and offer prime and nonprime binding interactions, inhibiting CTSK in the nanomolar range. The crystal structure of the complex formed between CTSK and **GD20** ensures covalent modification to the active-site cysteine, which may aid in the design of future inhibitors. **GD20** and **GD38** showed selective inhibition of CTSK in vitro, in primary mice and human cells, in the cell nucleus, and in excised human lung tissue. Their high potency and selectivity toward CTSK prove our approaches to be successful, generating important candidates for future research of illnesses linked with CTSK dysregulation and possible therapeutic usage.

## Results and Discussion

### Design and Synthesis of CTSK-Specific Inhibitors

Our attempt to generate highly potent, selective, and covalent irreversible CTSK inhibitors started by generating inhibitors that combined the electrophile (an AOMK) and capping group of a covalent irreversible pan-cysteine cathepsin inhibitor **GB111-NH_2_**.^37,39^ The compounds were designed to include **GB111-NH**_**2**_ and ODN elements (see Schemes S1 and S2 in the Supporting Information (SI), Figure S1). A diphenyl, which is recognized for its selectivity for CTSK, was conjugated to a short peptide sequence, and leucine or fluoroleucine, a cyclopropane, a hexyl linker, and a PMK warhead were all tested. The diphenyl moiety was generated using the Suzuki coupling reaction. Next, it underwent the synthesis of imine intermediates utilizing the methyl ester of l-leucine or 4-fluoro l-leucine, followed by diastereoselective reductive amination employing NaBH_4_ and ZnCl_2_.^40^ The resultant mixtures of diastereomeric acids were purified to yield stereopure versions of the target acids **9** and **10**. The peptide (amide) coupling reactions of compounds **9** and **10** with appropriate amines produced the desired products (**GD1**–**GD5**) (see Schemes S1 and S2 in the SI).^37^

In-gel fluorescence analysis was used to detect the residual activity of the cathepsins by applying pan-cathepsin probe **GB123** to detect the stable enzyme–probe complex. This approach is used for testing inhibition and selectivity.^37,41^ Unfortunately, although both AOMKs and a PMK-containing compound were tested, none of these compounds (**GD1**–**GD5**) were potent toward CTSK or any other cathepsins tested (Figures S1, S4, Table S2 in the SI).

**Figure 1:**
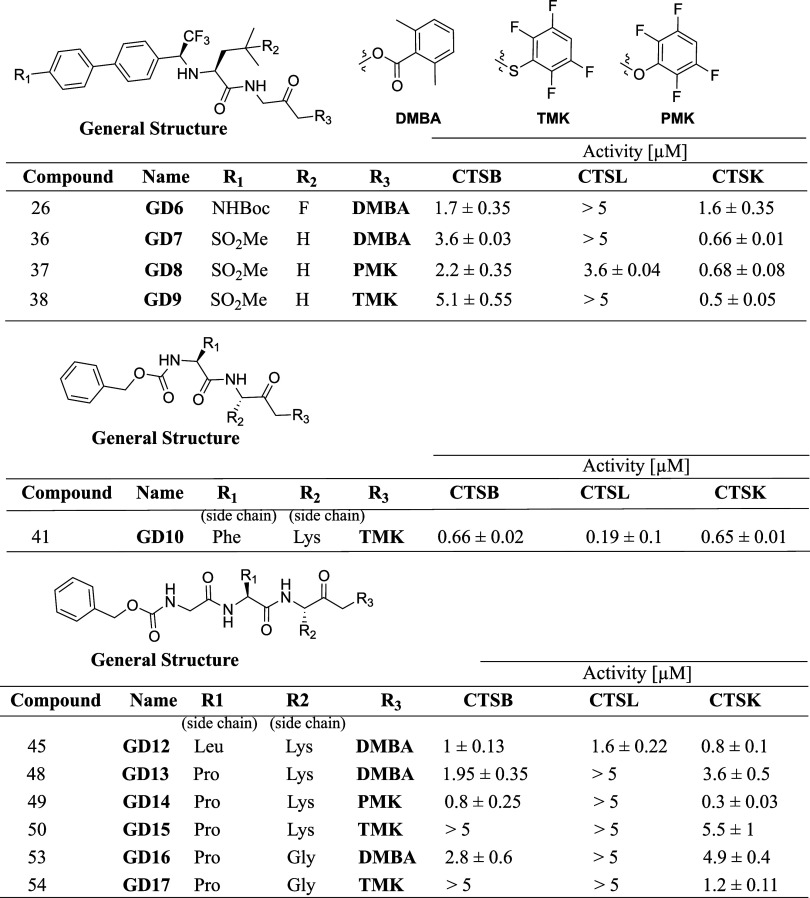
Chemical structure of compounds **GD6**–**GD10** and **GD12**–**GD17** with their IC_50_ values toward CTSB, K, and L. Here, dimethyl benzoic acid (DMBA), phenoxymethyl ketone (PMK), or thiomethyl ketone (TMK) form acyloxymethyl ketone (AOMK) warhead.

To reduce the impact of steric hindrance, the cyclopropane side chain was replaced with hydrogen to generate glycine at the P1 position, as Gly also appears at the P1 of the CTSK-specific substrates.^42^ In addition, the hexyl linker was eliminated in **GD6** (Figure 1). These changes significantly increased the potency of the inhibitor, however, without selectivity between CTSK and CSTB Figures 1, S4, Table S2). The addition of a methyl sulfonyl group to the compound’s biphenyl end increased the selectivity toward (CTSK) over the other cathepsins tested (**GD7**). While compounds (**GD7**–**GD9**) acquired some selectivity, their combined potency and selectivity were not good enough (Figures 1, S4, Table S2).

In parallel, to develop a CTSK-specific inhibitor with enhanced selectivity, we investigated various CTSK-specific substrate peptide sequences that were connected to an AOMK, PMK, or TMK warhead, which in turn were attached to different leaving groups (Figures 1 and S2). An examination of peptide sequences and warheads generated reveals that CTSK selectivity is better in compounds containing an AOMK electrophile and, in some cases, with better potency (**GD13** vs **GD15**) (Figure S4 and Table S2). We, therefore, shifted our focus to AOMK compounds.

The peptide sequence Abz-HPGGPQEDN_2_ph was recognized to be highly specific for CTSK over other competing cathepsins; it has been effectively utilized to monitor CTSK activity in diverse bodily fluids.^42^ Therefore, we also generated five novel inhibitors (**GD19**, **GD22**, **GD23**, **GD24**, and **GD25**) based on this substrate sequence in which we incorporated an acyloxymethyl ketone moiety as an electrophilic moiety between P1 and P1′ (Figure S3) in the middle of the molecule. Surprisingly, these compounds showed very low potency and no selectivity. Afterward, **GD13** was modified by replacing DMBA with the peptide-based AOMK warhead to produce **GD25** (Figure S3). Nevertheless, no improvement was observed (Figures S3, S4, and Table S2). Hence, we assume compounds that only include amino acids as binding moieties are too flexible for potent and selective interactions.

**Figure 2:**
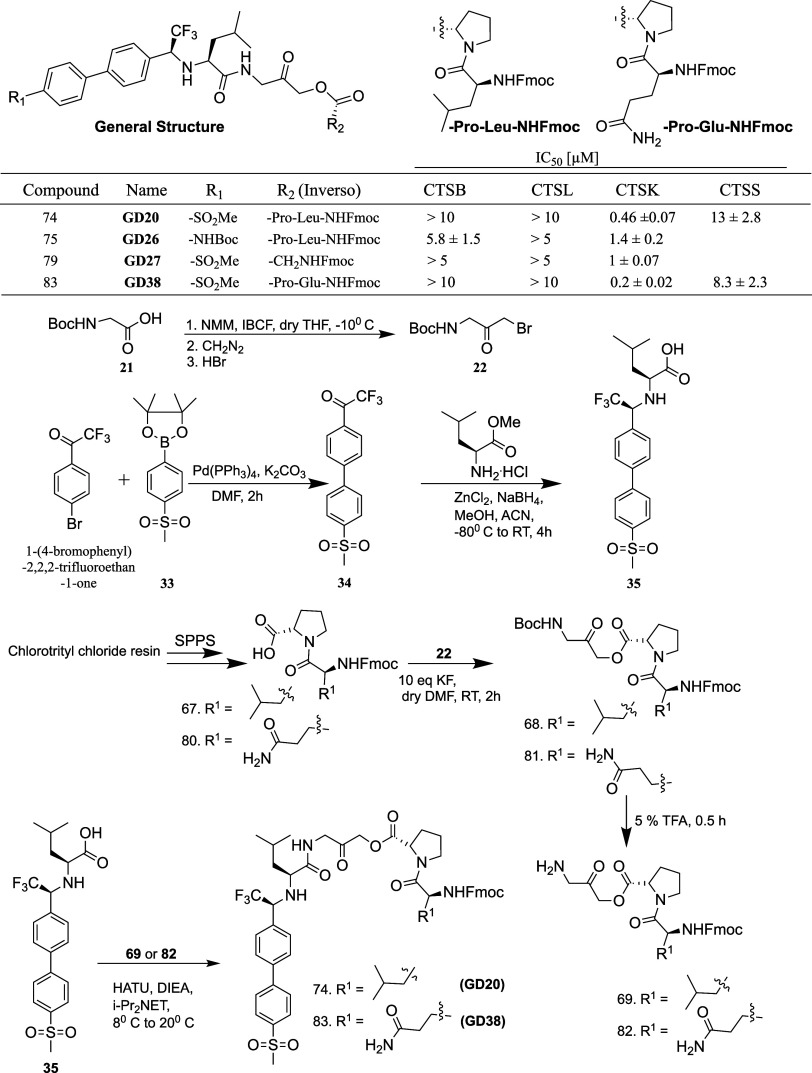
Chemical structure of compounds **GD20**, **GD26**, **GD27**, and **GD38**, with their IC_50_ values toward CTSB, CTSL, and CTSK. Synthetic scheme of compounds **GD20** and **GD38**. NMM, *N*-methylmorpholine; IBCF, isobutyl chloroformate; DIEA, *N*,*N*-diisopropylethylamine; DMF, dimethylformamide; ACN, acetonitrile; HATU, hexafluorophosphate azabenzotriazole tetramethyl uronium; and TFA, trifluoroacetic acid.

The AOMK was selected as the electrophile for a few reasons; since it serves as a good bioisostere of the glycine–glycine cleavage sequence within the substrate, it should produce selectivity and generate more rigid compounds. We continued to focus on hybrid small-molecule inhibitor compounds incorporating peptide mimics and the diphenyl moiety featured in odanacatib. Furthermore, using novel synthetic methods, the modified di-inverse amino acids sequence in the prime site of the **GD25** was linked through an AOMK to the nonprime site binding part of **GD6** and **GD7** (Figure 1), resulting in **GD20** and its analogue **GD26** (Figure 2). The inverse amino acids were linked to the AOMK to reduce the potential degradation of the inhibitors by other proteases and to increase selectivity. Their synthesis involved the utilization of Suzuki coupling, followed by the stereospecific reduction of an imine utilizing zinc chloride and sodium borohydride, and the peptide sequences were generated using solid-phase peptide synthesis. The bromine in α bromo glycine was replaced by the carboxylic group of the peptide moieties. Following the removal of the Boc-protecting group, the conjugated peptide segment was joined to the diphenyl segment using an acid–amine coupling process, yielding the intended products (Figure 2).

**GD20** has distinctive features that increase its potency and selectivity, such as a specific glycine, a bulky hydrophobic leucine moiety, and a biphenyl moiety at the P1, P2, and P3 positions, respectively, generating extra interactions with CTSK. To increase the specificity and potency of CTSK, proline and leucine were placed at the P2′ and P3′ positions while in an inverse orientation to increase selectivity. The AOMK electrophilic warhead at the interface between the two parts allows covalent irreversible binding of the protease’s cysteine nucleophile. The small-molecule-peptide inhibitors generated show high selective inhibition of CTSK over other cysteine proteases (Figures 2, S4 and Table S2). Furthermore, the compound Boc-protected amine (**GD26**) may serve as a viable chemical handle for attaching tissue-specific targeting moieties. **GD38** is an analogue of **GD20** that bears a similar sequence in the prime site as in the substrate Abz-HPGGPQ-EDN2ph and was found to be very potent and selective (Figures 2, S4, Table S2).

**Figure 3:**
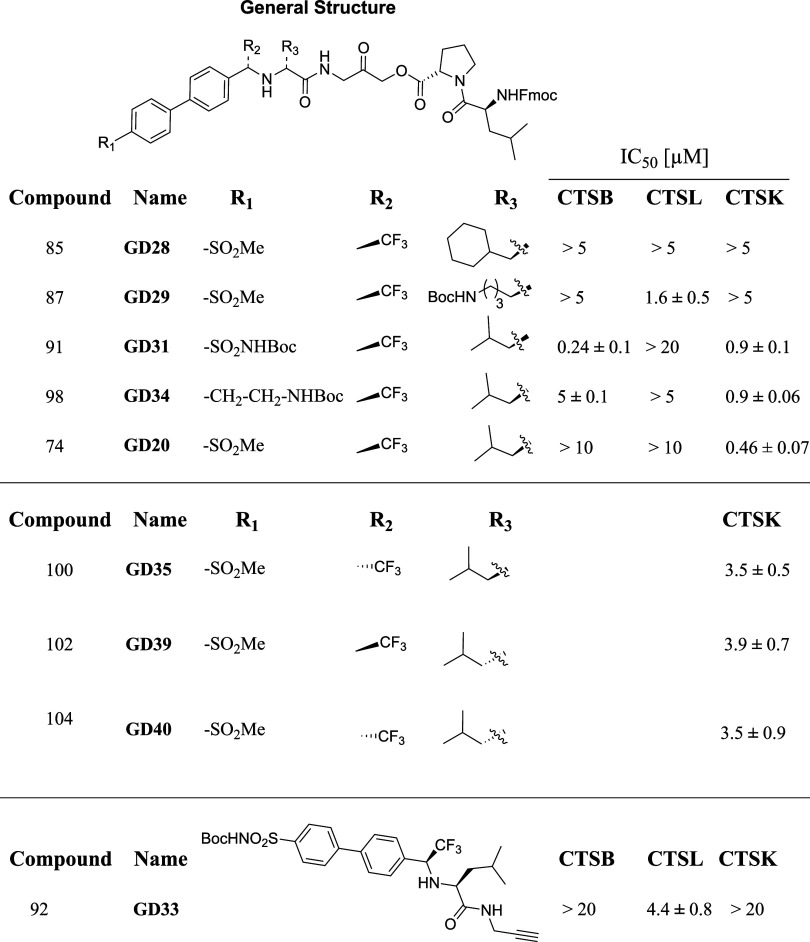
Chemical structure of compounds **GD28**–**29**, **31**, **33**–**35**, and **39**–**40**, with their selectivity potency toward CTSB, CTSL, and CTSK.

Based on the selective **GD20** and **GD38** (Figure 2), a small library of compounds bearing a methyl sulfonyl group was then generated (Figure 3); however, a lysine or cyclohexyl group at the P2 position (**GD28** and **GD29**) lowered the inhibitory activity against CTSK significantly. The SAR of the P2 position concluded that leucine is the best amino acid for this location, with high CTSK potency and selectivity against other cathepsins.

Efforts were aimed at providing the inhibitors with a chemical handle to allow future targeting of organs or cells, reducing the toxicity in vivo. **GD31** was synthesized with a minor alteration on the biphenyl ring, implanting a sulfonamide group (Figure 3). However, the methyl sulfonamide compound, **GD31**, was potent toward CTSB and lost its selectivity. This strongly indicates that the sulfonamide moiety is not suited for achieving CTSK selectivity in this particular position. Replacing the AOMK with an alkyne moiety as a warhead in **GD33** did not result in improved potency (Figures 3, S4, Table S2). Interestingly, when replacing the methyl group on the methyl sulfonyl group of **GD20** with a Boc-protected ethyl amine (**GD34**), the latter demonstrated only slightly lower potency and selectivity than the original molecule (Figures 3, S4, Table S2).

To investigate the fit to the active site and the impact of alternative spatial arrangements of the side chains at the P2 and P3 positions, three distinct diastereomers of **GD20**, with D amino acids (Leu and trifluoro Gly), were synthesized (**GD35**, **GD39**, and **GD40**). As predicted, these compounds lost 4–9 times their potency compared to **GD20**, emphasizing tight binding and the relevance of the specific chemical structure and spatial configuration, determining the binding efficiency to CTSK (Figures 3, S4, Table S2).

### Selectivity and Potency of Inhibitors

Both **GD20** and **GD38**, the top two inhibitors, were selected for testing their kinetic inhibition parameters toward CTSK and the other cathepsins, CTSB, CTSL, and CTSS, compared to ODN (Table 1). Both inhibitors selectively and irreversibly blocked recombinant CTSK with *K*_i_ in the nM range. Although the potency was lower than that of ODN, the selectivity over CTSS was dramatically higher in **GD20**. The kinetic values were corroborated with the recombinant data that show high selectivity of both **GD20** and **GD38**.

**Table 1:** IC_50_ and *K*_i_ Values (M) of the Compound Against Cathepsins^a^

cathepsin	**GD20**	**GD38**	ODN
CTSK	irreversible	irreversible	reversible (tight binding)
	*k*_inact_/*K*_i_: 231,714 M^–1^ s^–1^	*k*_inact_/*K*_i_: 229,653 M^–1^ s^–1^	IC_50_ = 0.942 nM
	*k*_inact_: 0.0026 s^–1^	*k*_inact_: 0.0032 s^–1^	
	*K*_i_^*^: 13.0 nM	*K*_i_^*^: 20.9 nM	
CTSS	*K*_i_ > 50 μM	irreversible	reversible
		*k*_inact_/*K*_i_: 178 M^–1^ s^–1^	*K*_i_ = 136 nM
		*k*_inact_: 0.009 s^–1^	
		*K*_i_^*^: 62.0 μM	
CTSL	*K*_i_ > 50 μM	irreversible	
		*k*_inact_ (at 50 μM): 0.0045 s^–1^	
		*K*_i_^*^ > 50 μM	
CTSB	*K*_i_ > 50 μM	irreversible	
		*k*_inact_ (at 50 μM): 0.0072 s^–1^	
		*K*_i_^*^ > 50 μM	

a The indicated cathepsin proteases and inhibitors were mixed together before the fluorogenic substrate Z-RR-AMC (CTSL) or Z-FR-AMC (CTSS, CTSB, and CTSK) were added. Inhibition was determined by blockage of the generation of a fluorescent signal over time; details are in the SI. For irreversible inhibitors, *K*_i_^*^ = *k*_obs_.

**Figure 4:**
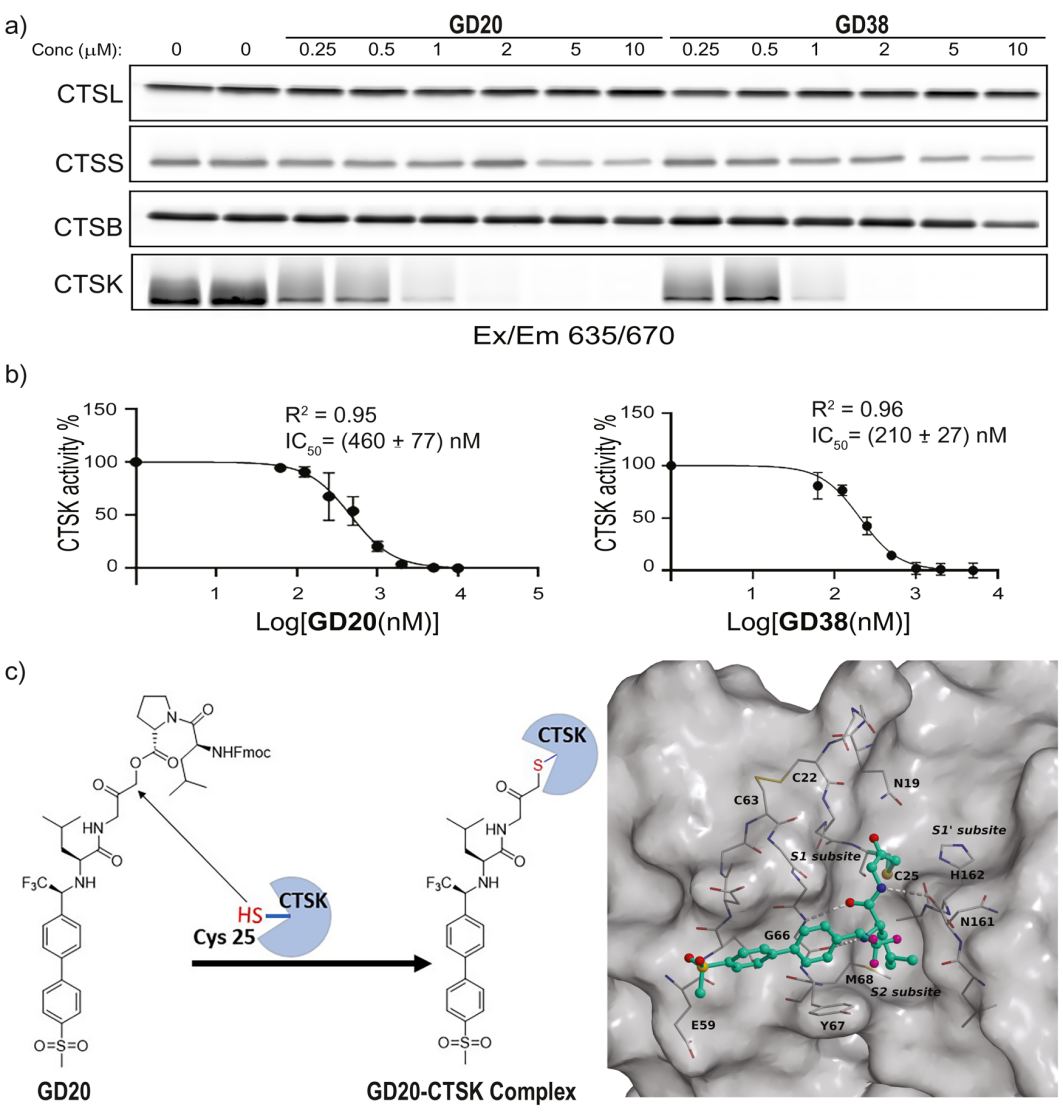
Inhibitor evolutions on recombinant cathepsins. (a) Inhibition of recombinant cathepsins by **GD20** and **GD38**. Recombinant cathepsins B, K, L, and S (0.5 μM) were treated with vehicle (dimethyl sulfoxide, DMSO) or increasing concentrations of inhibitors, followed by incubation with **GB123** (5 μM). Samples were separated on sodium dodecyl sulphate-polyacrylamide gel electrophoresis (SDS-PAGE) and scanned for fluorescence by a Typhoon scanner at 635/670 nm. (b) IC_50_ curves of **GD20** and **GD38** for inhibiting CTSK activity. (c) On the left, the structure of **GD20** before and after covalent bond formation with CTSK. On the right, the X-ray crystal structure of **GD20** bound to CTSK. A covalent bond exists between CTSK **Cys25** and the methylene of **GD20**, together with the position of the inhibitor in the S2 and S3 binding pockets. PDB ID: 9G6A. See the detailed method and crystal description in the SI.

To further corroborate the inhibition of **GD20** and **GD38**, the residual activities of cathepsins B, L, S, and CTSK were detected after treatment with the inhibitors. The residual recombinant cathepsin activity was detected by a pan-cysteine cathepsin fluorescent activity-based probe (ABP) **GB123**(37) (Figure 4a). Elimination of CTSK activity is evident at low inhibitor concentrations, while other cathepsins are barely affected at 10 μM. **GD20**, while slightly less potent toward CTSK compared to **GD38**, has extraordinary selectivity over other cathepsins, likely because of its interaction with the prime site binding pockets. IC_50_ curves show 460 ± 77 and 210 ± 27 nM for **GD20** and **GD38**, respectively (Figure 4b); significant CTSK inhibition starts at 0.25 and 0.062 μM for **GD20** and **GB38**, respectively (Figure S5).

### Inhibitor Binding Mechanism to the Catalytic Cysteine of CTSK

The covalent modification of **GD20** with CTSK is proven by a crystal structure that was resolved at 1.7 Å, in which CTSK active-site **Cys25** forms a stable covalent bond to the electrophilic methylene of the AOMK (Figures 4c and S6). This covalent link strongly confirms our hypothesis of a covalent irreversible binding mechanism between the inhibitor and the enzyme. After demonstrating the irreversible binding of **GD20**, we continued to evaluate the capabilities of the inhibitors in the natural cellular milieu.

### Selective Inhibition of CTSK Activity in Cell Lines

**Figure 5:**
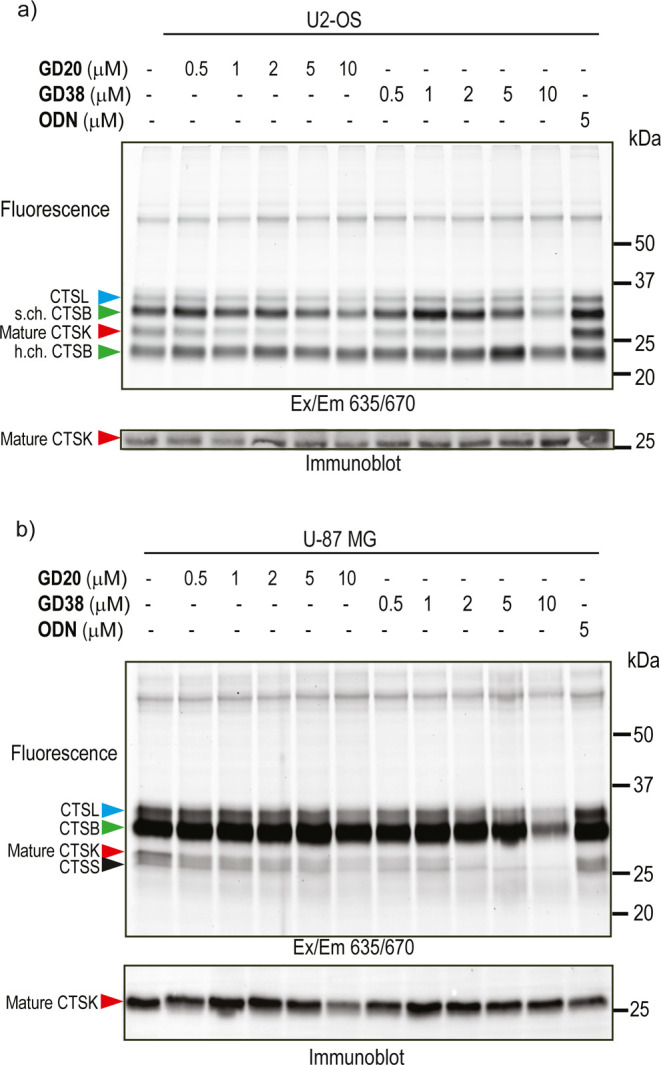
Direct inhibition of endogenous matured CTSK in intact U2-OS and U-87 MG cells. (a) Intact U2-OS cells were treated with DMSO, ODN, or increasing concentrations of the inhibitors **GD20** or **GD38** in their culture medium for 2 h. Cells were then treated with **GB123** (5 μM) for 2 h, lysed, and separated by SDS-PAGE, followed by fluorescent scanning of the gel using a Typhoon scanner at 635/670 nm. An immunoblot of CTSK of the same gel is presented below. Different cathepsins are marked by colored arrowheads; a red arrowhead marks mature CTSK. s. ch. = single chain, h. ch. = heavy chain.^45^ (b) Intact U-87 MG cells were treated with the inhibitor, followed by **GB123**, as described in panel (a).

Two cell lines, human osteosarcoma U2-OS and human glioblastoma U-87 MG, that naturally express CTSK along with other lysosomal cathepsins, were chosen to evaluate the inhibitors’ potency and selectivity.^43,44^ First, the intact cells were incubated for various durations with escalating doses of the inhibitors, and the ODN was used as a control. The residual cathepsin activity in the cells was detected by adding a pan-cathepsin ABP, **GB123**, after which the cells were lysed. The probe-labeled enzymes were then separated and visualized using fluorescent SDS-PAGE (Figures 5a,b and S7).^45^ In both cells, the activity of several cathepsins was detected, while the fluorescent band at approximately 25 kDa was correlated to CTSK by immunoblotting (shown below in Figure 5a, b). The identity of each labeled band was detected by immunoblotting (Figure S7).^45^ A selective dose-dependent suppression of CTSK activity was obtained in a range of inhibitor concentrations. However, while **GD20** was very selective at all concentrations tested, **GD38** slightly reduced the activity of other cathepsins at high concentrations and longer incubation times. As a result, specific conditions were selected in which selective CTSK inhibition was achieved with **GD38**. Remarkably, we could not detect ODN’s inhibition; we hypothesize that the covalent reversible nature of ODN leads to its replacement with the **GB123**(37) probe (having a stronger electrophile) in samples with relatively low CTSK activity.

### Inhibition of CTSK Activity in Primary Human Osteoclast

**Figure 6:**
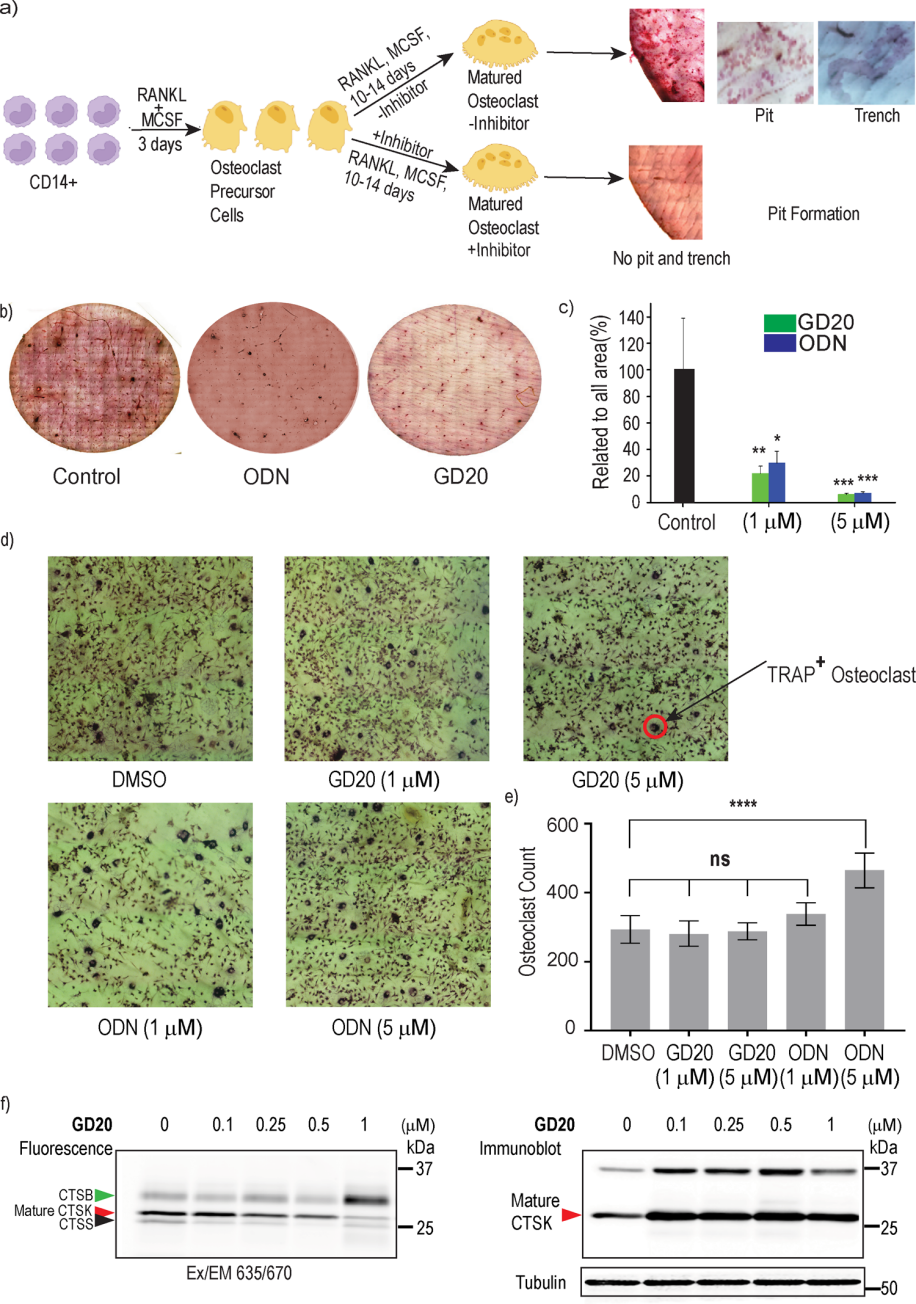
CTSK inhibition in human osteoclasts. (a) Scheme of the process of maturation of osteoclasts (OCs) derived from human monocytes in the presence of RANKL and M-CSF. (b) Representative pictures of bone resorption after incubation with human OCs treated with vehicle or inhibitors for 10–14 days. Bone resorption was observed by a toluidine blue stain. Increased staining indicates a higher number of resorption pits and greater bone resorption. (c) Quantitative analysis of pit formation in bone slices after incubation with OC treated with **GD20** or ODN (1, 5 μM) relative to vehicle treated slices. Data represent mean ± standard errors, and asterisks represent *p* values relative to control, *p < 0.05, **p < 0.01, ***p < 0.001, *n* = 3 bone slices. (d) Representative images of tartrate-resistant acid phosphatase (TRAP) staining of the human osteoclasts on bone slices in the presence of vehicle or inhibitors, as indicated. The red circle shows an example of a TRAP-positive osteoclast. (e) Quantification of osteoclasts number on bone slices after inhibitor treatment, detected by TRAP staining. (f) Human OCs were treated with vehicle or increasing concentrations of **GD20 for** 11 days (changing the medium every 2–3 days). The activity of CTSK in the cells was detected by 2 h labeling with a pan-cathepsin activity-based probe, **GB123**, followed by a fluorescence scan of the SDS-PAGE detecting CTSK attached to the probe; the red arrow points to the bands of CTSK (above 25 kDa). CTSK immunoblot of the same OCs is presented aside.

The selective blockage of cellular CTSK activity allowed us to assess the effect of the inhibitors on bone resorption induced by osteoclasts (OCs) as an important step toward therapeutic application in osteoporosis, bone metastases, and other diseases associated with excessive bone resorption. Human OCs were derived from CD14+ monocytes that were extracted from healthy donors’ blood and treated with M-CSF and RANKL, as described in Abdallah et al.^46^ The OC were grown on cortical bone slices over 14 days in the presence of the CTSK inhibitors to evaluate the impact of inhibitors on bone resorption; see scheme (Figure 6a).

Staining of the bone slices exposed the resorption caused by the OC as trenches and pits; representative images of the bone slices after the treatments are shown in (Figure 6b).^47^ The pits were quantified; while 5 μM ODN-treated OC inhibited (93 ± 1.45)% of the pit formation, 5 μM **GD20** inhibited (94 ± 1.57)%, compared to vehicle-treated OC (Figure 6b,c). Osteoclasts cultured on bone slices and treated with the inhibitors were also stained with TRAP and counted. An increase in osteoclast numbers was detected after the ODN treatment, while **GD20** exhibited no significant alterations in quantity (Figure 6d,e). Additionally, osteoclasts were grown on plastic in the presence of **GD20** and stained with TRAP to determine their size and maturation (Figure S8). The total osteoclast area was slightly decreased in the presence of the inhibitor, and on the bone slices, the number of osteoclasts was not affected compared to the control vehicle treatment. The OC were also examined for residual cathepsin activity after inhibitor treatment using the pan-cathepsin probe **GB123**. The OC cell lysates were then separated by SDS-PAGE, and the fluorescence band intensities were detected. As expected, the activity of CTSK was inhibited selectively in a dose–response manner; even 1 μM **GD20** blocked most of the CTSK activity, and this was accompanied by the increase in CTSB activity (Figures 6f and S9a). Interestingly, all inhibitor concentrations led to elevated CTSK protein levels as detected by immunoblot, even the lowest concentration (Figure 6f), as previously reported.^47^ The significant inhibition of CTSK’s activity correlates nicely with the blockage of bone resorption (Figure 6f).

### Inhibitors Lack Toxicity

Cell viability experiments were conducted to confirm that the decrease in bone resorption is a result of specific inhibition of CTSK’s activity and not cell death. These assays demonstrated that the cells remain viable at the requisite concentrations of inhibitors (Figure S10).

### Inhibition of CTSK Activity in Primary Mice Osteoclasts

**Figure 7:**
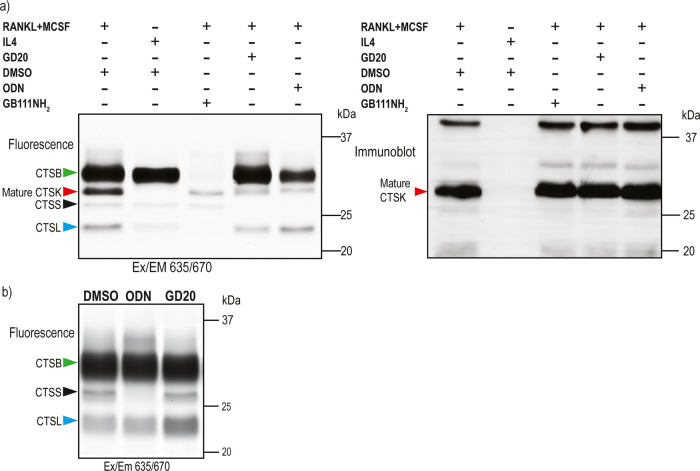
CTSK inhibition in mice osteoclasts. (a) Activity and expression of CTSK in mouse OCs were assessed in the presence of indicated inhibitors. Intact OCs were treated for 2 h with 5 μM inhibitor in the culture medium, followed by 2 h of labeling of residual enzyme activity by **GB123**. Samples were analyzed by SDS-PAGE and scanned for fluorescence with a Typhoon scanner at 635/670 nm. Western blot with anti-CTSK is shown aside. (b) IL-4-induced macrophage cells were treated for 2 h with 5 μM inhibitors or vehicle in a culture medium, followed by 2 h of labeling of residual enzyme activity by **GB123** (5 μM). Samples were analyzed by SDS-PAGE and scanned for fluorescence with a Typhoon scanner at 635/670 nm.

Human and mouse CTSK species have a high degree of structural commonality. Interestingly, three amino acid positions in CTSK may lead to functional variations between human and mouse CTSK.^48^ Therefore, to investigate the inhibitors’ cross-reactivity with mouse CTSK, mouse OCs were cultivated with mouse bone marrow cells stimulated with M-CSF and RANKL for 9–10 days. In addition, control cells differentiated into macrophages that highly express active cathepsins but not CTSK and were generated by treatment with M-CSF and IL-4.^49^ Both intact OC and macrophage cells were incubated for 2 h with **GD20**, vehicle, or ODN, followed by a 2 h incubation with pan-cathepsin probe **GB123**. The CTSK-specific fluorescent band near 25 kDa disappeared in cells treated with **GD20**, whereas the other competing band remained unaltered (Figure 7a). Mice OS samples were blotted to identify the different cathepsins in the sample (Figure S9b). The specificity of **GD20** was demonstrated by the lack of inhibition of any band in macrophages that lack CTSK (Figures 7b and S11). This study shows very clearly that **GD20** is very selective and effective against mice CTSK.

### Fluorescence Microscopy of CTSK Inhibition in Cell Lines

**Figure 8:**
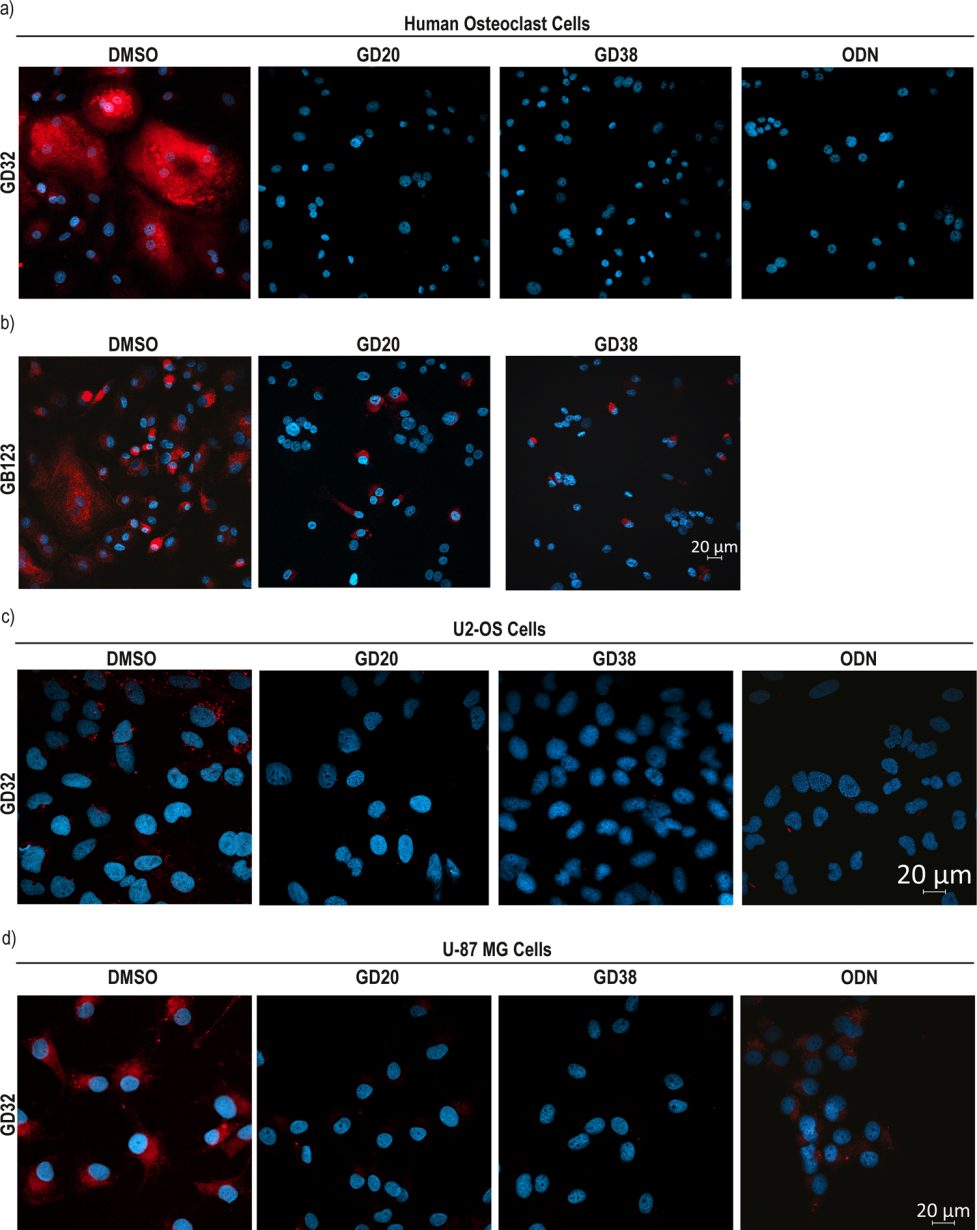
Detection of inhibition of endogenous cathepsin K in intact cells by fluorescence microscopy. Enriched human monocytes from peripheral blood mononuclear cells (PBMC) were isolated from healthy donors’ blood and were treated for 14 days with M-CSF and RANKL or M-CSF alone, as indicated. Osteoclast cells were treated with vehicle (DMSO) or indicated inhibitors [3 μM] in a culture medium for 2 h, followed by incubation with (a) **GD32** (a specific CTSK ABP) for 8 h or (b) **GB123** for 2 h. Similar to those described in panel (a), cultures of U2-OS cells (c) and U-87 MG cells (d) were grown in 8-well chambers. Cells were pretreated with either the DMSO or [3 μM] of indicated specific CTSK inhibitors for 2 h and then labeled by the addition of 1.25 μM **GD32** to the culture medium for 24 h (U2-OS) or 2 h (U-87 MG). After fixation, cells were stained with 4′,6-diamidino-2-phenylindole (DAPI) (nucleus), and images were captured by confocal microscopy.

We next corroborated CTSK-specific inhibition by fluorescence microscopy studies of OC. Human OC were grown on 8-well plates; the intact cells were treated with either of the CTSK inhibitors or with the vehicle and then with the CTSK-specific fluorescent ABP **GD32**.^19^ The high specificity of the **GD32** ABP for CTSK activity in primary human OC and cell lines is shown in Figure S12.^19^ The fluorescence microscopy pictures were acquired following cell fixation, which permitted imaging and evaluating CTSK activity and inhibition in cells. The inhibitors completely abolished CTSK activity and blocked the probe from binding to the enzyme (Figure 8a). However, osteoclasts treated with the inhibitor (**GD20** and **GD38**) and then with the pan-cathepsin probe **GB123** had only a partial reduction in the fluorescence signal. As the inhibitors are specific to CTSK, they could not block the fluorescence signals of **GB123** associated with other cathepsins (B, L, S) (Figure 8b). Similar experiments with U2-OS and U-87 MG cell lines, both expressing active CTSK, show similar results in that **GD20** and **GD38** block CTSK activity (Figure 8c,d).

### Inhibition of Nuclear CTSK

**Figure 9:**
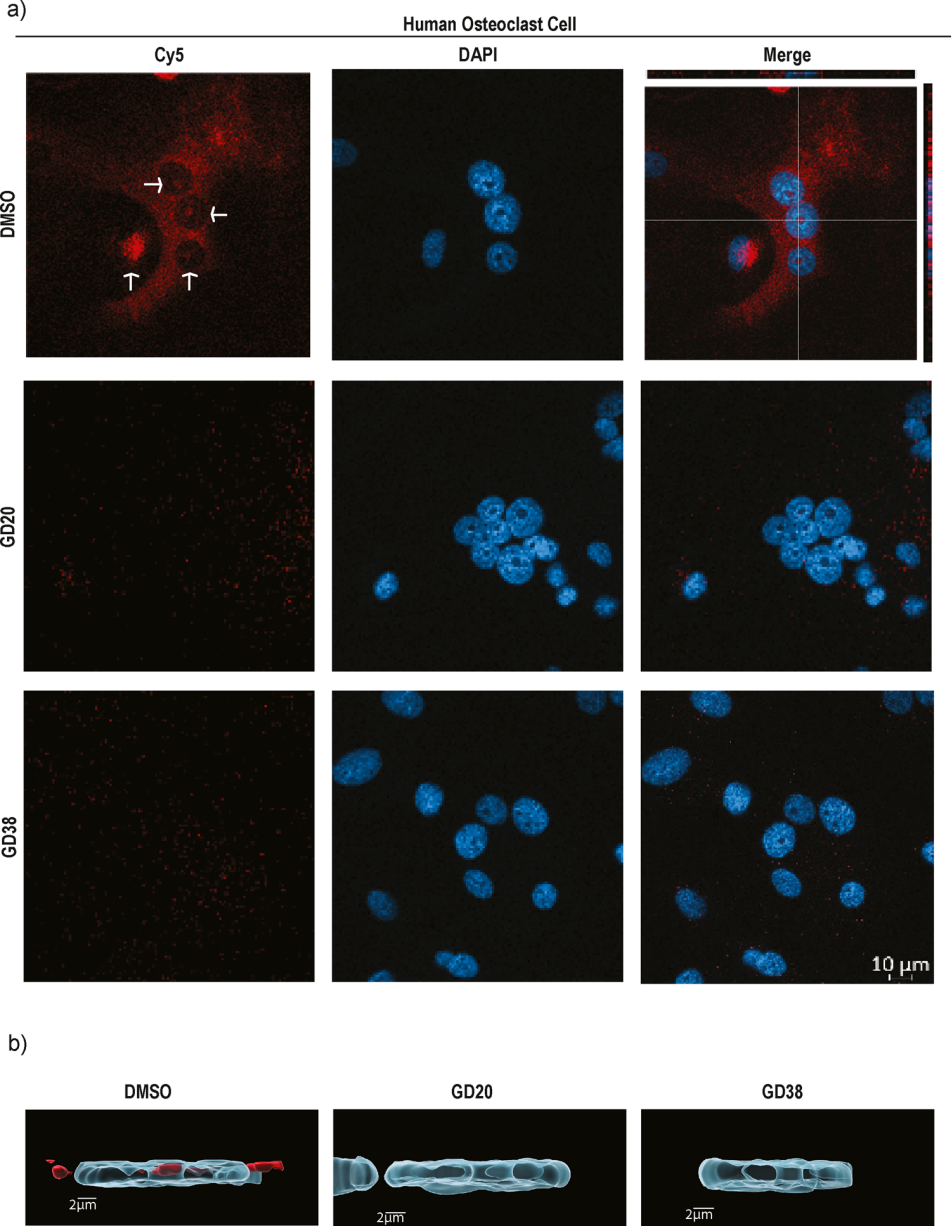
Detection of inhibition of endogenous cathepsin K in the nucleus of human osteoclast by fluorescence microscopy. (a) Osteoclast cells were treated with **GD20** or **GD38** inhibitors [3 μM] or DMSO vehicle in culture medium for 2 h, followed by incubation with **GD32** for 8 h, as shown in Figure 8a. Cells were then fixed and stained with DAPI (nucleus), and Z stack images were captured by confocal microscopy. The white arrows indicate the nucleus. The XZ and YZ portions provide evidence of CTSK activity in the cell’s nucleolus, and the inhibitors were found to block CTSK activity in the nucleolus. Red fluorescence is CTSK activity labeled by the ABP (**GD32**) and blue fluorescence (DAPI). (b) Microscopy image analysis software Imaris 10.0.1 was used to produce a three-dimensional (3D) surface rendering image of a cell nucleus (transparent blue) and CTSK inside it (opaque red) on top of the original image (blue, DAPI; red, **GD32**). The thresholds for the surfaces were chosen to emphasize CTSK inside the nucleus. One nucleus for each treatment was chosen from pictures in (a).

We have generated inhibitors that block nuclear CTSK activity for the first time. CTSK-specific inhibitors can penetrate the nucleus, as well as nucleoli, and selectively inhibit the activity of CTSK in the nuclear compartment of the cell (Figure 9). CTSK activity labeled by a specific fluorescence ABP, **GD32**, was detected in the nucleus and nucleoli of osteoclasts, as reported by Dey et al.^19^ The CTSK nuclear signal was completely eliminated in the presence of the inhibitors (Figure 9). These inhibitors can facilitate studying CTSK nuclear activity.

### Inhibition of CTSK Activity in Human Lung Cancer

**Figure 10:**
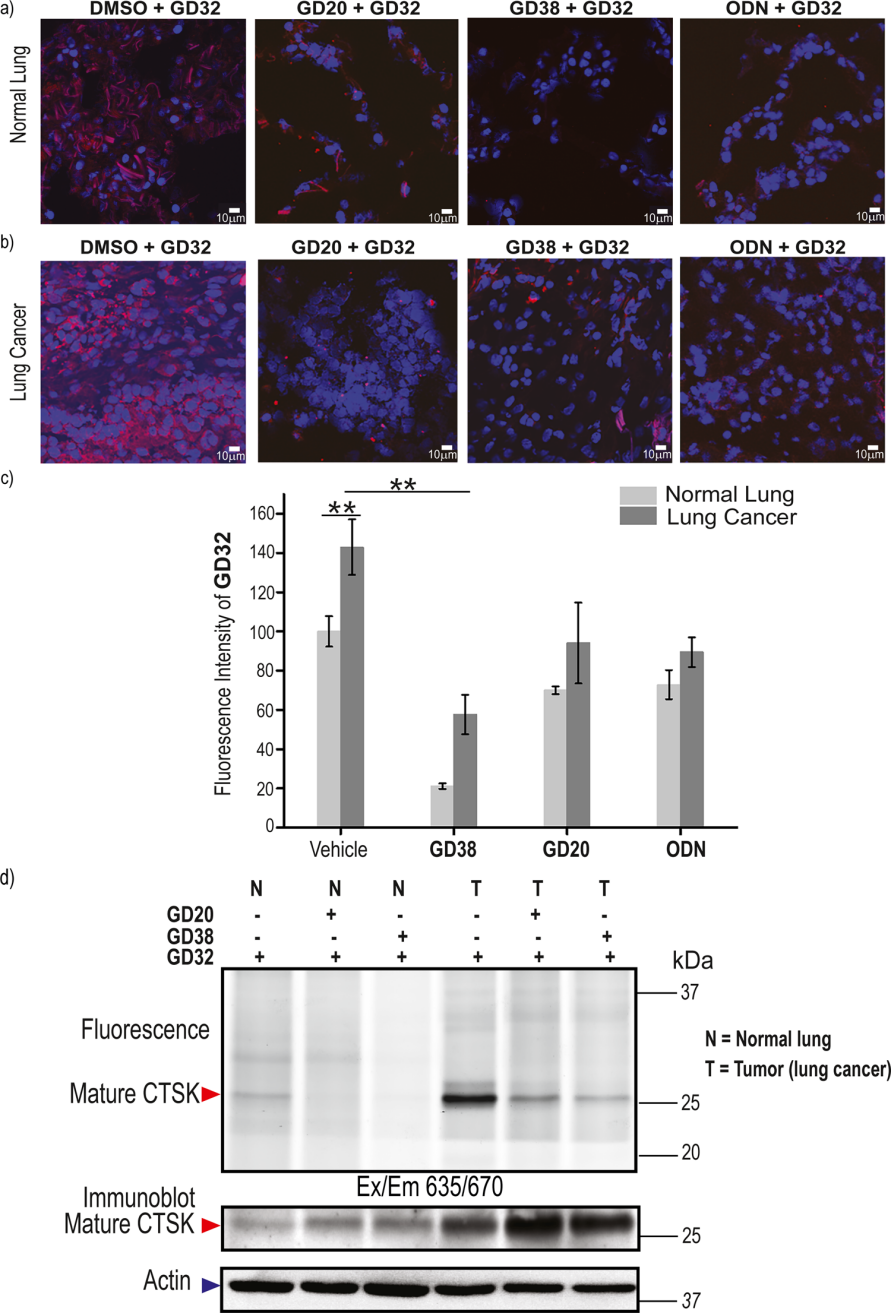
CTSK inhibition in human lung tissue. Human lung samples: (a) normal and (b) tumor lungs were treated with DMSO, **GD20**, **GD38**, or ODN (5 μM) for an hour before incubation with **GD32** (0.75 μM) for 4 h. The samples were fixed, stained with DAPI (nucleus), and subjected to fluorescence microscopy; indicative images are shown. (c) Quantification of the CTSK activity labeled by **GD32** (red) in the images of human lung tissues (a, b) normalized to the DAPI stain. (d) Human lung samples were treated with DMSO, **GD20**, or **GD38** (10 μM) for 3 h, followed by incubation with **GD32** (10 μM) for 17 h, before the cell lysis, SDS-PAGE separation, and fluorescent scanning of the gel using a Typhoon scanner at 635/670 nm. The gel then underwent Western blotting with an anti-CTSK antibody and anti-actin as a loading control. The position of the active mature CTSK is indicated.

Several researchers have set out to determine the specific involvement of CTSK in lung cancer. Therefore, we investigated whether our inhibitors can modulate CTSK activity in human lung cancer tissue as well as in normal human lung tissue. To this purpose, the inhibitors were applied to freshly resected human lung tissues, both normal and malignant tissues. Tissues were subsequently treated with the CTSK-specific probe **GD32**, and CTSK activity was visualized using fluorescence microscopy (Figure 10a,b). The fluorescent signal in the cells, marking CTSK activity, was quantified and normalized to the signal in the vehicle-treated normal lung tissue (Figure 10c). These images show that the cells within the lung cancer tissues have CTSK activity inherently stronger than that in the normal lung tissue cells. We noticed that collagen fibers are more pronounced in normal lung tissue and that the probe tends to bind them in a nonspecific manner. Importantly, in all cases, when the inhibitors were present, the signal strength dropped within the cells, indicating that the inhibitors permeated the tissue and reduced the CTSK activity.

To validate our findings, we lysed the treated tissues and used fluorescence SDS-PAGE to see the probe-labeled protein. The fluorescent band with a molecular weight of approximately 25 kDa, which represents the active mature form of CTSK, was enhanced in the tumor sample. The inhibitor treatment resulted in the elimination of this band, blocking the active site of CTSK from functioning and binding the probe (Figure 10d). Western blot also demonstrated the overexpression of CTSK in lung cancer (Figure 10d). This study shows that CTSK may play a role in lung cancer and that our inhibitors may modulate the activity associated with the illness. However, it should be highlighted that this trial offers only indirect evidence of the possible therapeutic use of CTSK inhibitors. More research is required to investigate the exact processes and consequences of CTSK activity in lung cancer and assess the inhibitors’ effectiveness in more complicated contexts, such as in vivo or clinical trials.

## Conclusions

Ultimately, we have demonstrated the creation of a distinctive category of covalent irreversible inhibitors for CTSK, with *K*_i_ values falling within the nanomolar range. These inhibitors are very effective and selective. The unique inhibitors combine a few innovative chemical approaches in their design: attaching peptide sequences in the prime binding pockets, including amino acids in the inverse orientation, combining small organic moiety in the molecule, and employing AOMK as a targeted warhead for cysteine residues that also serve as a bioisostere to the cleavage sequence. The SAR analysis demonstrated that the AOMK has significant reactivity toward the cysteine residue, and it can be employed as a specific warhead for a particular protease by integrating it into a specific molecular sequence, unlike PMK. The SAR investigation also revealed that the peptide sequences play a critical role in contact with the enzyme’s prime site, whereas the diphenyl component of the inhibitor is vital for the interaction with the nonprime site. The X-ray structure shows a covalent enzyme–inhibitor complex, which was refined at a resolution of 1.7 Å, and the covalent binding of the inhibitor’s AOMK to the enzymes’ active-site cysteine was proven. Importantly, despite the intricate cellular environment, the inhibitors show significant potential in specifically inhibiting the activity of CTSK. These findings were confirmed using fluorescence microscopic imaging and gel analyses. Our investigation validates that the bone loss caused by osteoclasts can be ceased by inhibiting CTSK’s activity permanently in a targeted manner, without adversely impacting cell survival. Furthermore, **GD20** suppressed pit formation on the bone surface nearly identical to that of odanacatib. Through meticulous examination of cathepsin activity in osteoclasts treated with inhibitors, it becomes evident that while the expression of CTSK is increased, the overall activity of the protease is inhibited, reflecting the actual cause of the decrement of pit formation. Moreover, the inhibitors can selectively block the activity of CTSK in lung cancer, where it is thought to play a vital role in the progression of malignancies.

Surprisingly, although the mouse and human CTSKs have different structural features, our inhibitors efficiently blocked both CTSK enzymes, suggesting potential applications in clinical and preclinical settings.

The main goal of inhibitor development is to improve the efficacy of therapeutics and their safety profile by reducing off-target effects. In comparison to other inhibitors, **GD34**, a variation of **GD20** that incorporates a Boc-protected amine, has shown good selectivity for CTSK (Figures 3 and S13). This inhibitor has a chemical handle that can be used to specifically target organs or cells in order to reduce toxicity in living subjects. These specific inhibitors can be employed to address inquiries regarding the cause of the negative effects produced by odanacatib, and notably, they may also function as possible secure treatments.

## Materials and Methods

The solvents (*n*-hexane, dichloromethane (DCM), methanol, and ethyl acetate), which were used in purifying compounds through column chromatography, were purchased from a commercial source and used without any distillation. All resins and reagents were purchased from commercial suppliers and used without further purification. Silica gel (120–200 μm, 60 Å) was used as the stationary phase for column chromatography. Merck silica gel 60-F-254 plates were used for thin-layer chromatography (TLC). Deionized water from a Milli-Q system was used in all experiments. All experiments used freshly prepared phosphate buffer saline (pH = 7.4, 100 mM) as a buffer system. All water-sensitive reactions were performed in anhydrous solvents under a positive pressure of argon. Cy5 succinimide ester (SE) was purchased from Lumiprobe. Reactions were characterized by liquid chromatography-mass spectrometry (LC-MS) (Thermo Scientific MSQ-Plus attached to an Accela UPLC system) using reversed-phase chromatography with a water/acetonitrile gradient. Recombinant human cathepsins B, K, L, and S were prepared as described.^50−52^ The Animal Ethics Committee of Hebrew University approved all animal procedures. Fluorescent gels were scanned with a Typhoon FLA 9500 flatbed laser scanner (GE Healthcare Bio-Sciences AB, Uppsala, Sweden).

### Instruments and Measurements

A Bruker Advance III 500 MHz spectrometer was used for measuring ^1^H and ^13^C NMR spectra of the compounds in CDCl_3_, CD_3_OD, and DMSO-*d*_6_. Bio-Rad ChemiDox XRS+ was used for Western blot analysis. Sciex Triple Quad TM 5500 was used for mass spectrometry analysis of compounds. A laser scanning confocal microscope (Nikon C2 plus) with NIS element software was used for confocal microscopy measurement.

### Recombinant Cathepsin Inhibition

Recombinant human cathepsin L (0.5 μM), cathepsin B (0.5 μM), cathepsin K (0.5 μM), and cathepsin S (0.5 μM) were pretreated with different concentrations of inhibitors (**GD** compounds, odanacatib) for 1 h in reaction buffer (50 mM acetate, 2 mM dithiothreitol (DTT), and 5 mM MgCl_2_, pH 5.5) at 37 °C. GB123 (1 μM) was added and incubated with samples for 60 min at 37 °C.^37,41^ The reaction was stopped by the addition of sample buffer 4× (40% glycerol, 0.2 M Tris/HCl pH 6.8, 20% β-mercaptoethanol, 12% SDS, and 0.4 mg/mL bromophenol blue). Samples were then boiled, separated on a 12.5% SDS gel, and scanned for fluorescence by a Typhoon scanner FLA 9500 at excitation and emission wavelengths of 635 and 670 nm for **GB123**.

### Kinetic Evaluation of Recombinant Cathepsin Inhibition

Cathepsin (0.4 nM, CatK) or 5 nM (CatS, CatB, and CatL) were each mixed with inhibitors before adding the fluorogenic substrate. The proteases were then reacted with their substrates, Z-RR-AMC (CatL) or Z-FR-AMC (CatS, CatB, and CatK), for 30 min at 37 °C in the phosphate buffer (100 mM Na_2_HPO_4_/NaH_2_PO_4_, 50 mM NaCl, 5 mM DTT, 0.1% PEG-6000, at pH 6). Reactions were done in 96-well black flat-bottom microplates (Greiner, Germany); fluorescence (Ex/Em 370 and 460 nm) was measured by a Tecan INFINITE M1000 pro plate reader (Tecan, Switzerland) in the presence of inhibitors or vehicle. For irreversible inhibitors, reaction data were analyzed by using the one-phase association formula in GraphPad Prism 9 software: *Y* = *Y*_0_ + (plateau – *Y*_0_) × (1 – exp(−*K* × *X*)), where *Y*_0_ and *Y* denote the fluorescence signals at times 0 and *t*, respectively, *K* represents the observed reaction rate *k*_obs_, and *X* signifies the inhibitor concentration. The inactivation rates at each inhibitor concentration were determined by subtracting the inactivation observed in the control sample: *k*_obs_ – *k*_ctrl_. The maximum inactivation rate (*k*_inact_) was derived as the asymptote from the equation *Y* = *k*_inact_ × *X*/(*K*_i_^app^ + *X*), where *X* represents the inhibitor concentration, *Y* is the observed inactivation rate for each *X*, and *K*_i_^app^ is the apparent inhibition constant. *K*_i_ values were derived from IC_50_ or *K*_i_^app^ using the equations *K*_i_ = *K*_i_^app^/(1 + *K*_m_/*S*) and IC_50_ = *E*_0_/2 + *K*_i_^app^, where *K*_m_ represents the Michaelis–Menten constant, *S* denotes substrate concentration, and *E*_0_ indicates cathepsin concentration. The maximum inhibitor dose in the experiment was 50 μM. Measurements were conducted in multiple independent duplicates. Average values are listed.

### Cell Culture

Human U-87 MG cells were cultured in Dulbecco’s modified Eagle’s medium (DMEM) containing 10% fetal bovine serum (FBS), 1% penicillin, and 1% streptomycin. Human bone osteosarcoma epithelial cells (U2-OS) were cultured in McCoy’s 5A medium containing 10% fetal bovine serum (FBS), 1% penicillin, and 1% streptomycin. For mice osteoclast preparation, bone marrow was extracted from the tibia and fibula of C57BL/6 mice and cultured in α MEM medium containing 10% fetal bovine serum (FBS), 1% penicillin, and 1% streptomycin with 30 ng/mL RANKL and 20 ng/mL M-CSF for 7–9 days until the multinucleated giant osteoclast cells have appeared. For macrophage preparation, the same procedure was maintained with M-CSF and IL-4 without RANKL. All cells were maintained in an incubator at 37 °C with a 5% CO_2_/air environment.

### Evaluation of the Permeability of Inhibitors to Intact Cells, Competition Assay

U-87 MG and U2-0S cells (5 × 10^5^ cells/well) were seeded in a six-well plate 1 day before treatment. Cells were treated with vehicle or different concentrations of inhibitors that were predissolved in 0.1% DMSO in a culture medium. After 2 h of inhibitor incubation, residual cathepsin activity was labeled with **GB123** (5 μM) (final DMSO concentration was maintained at 0.2%).^37,41^ Cells were washed with phosphate-buffered saline (PBS) and lysed with the addition of radio immunoprecipitation assay (RIPA) buffer, pH 7.4 (1% Tergitol-type NP-40 (nonyl phenoxypolyethoxylethanol), 0.1% SDS, 0.5% sodium deoxycholate). Proteins were quantified by bicinchoninic acid (BCA, Thermo Scientific); an equal quantity of proteins was prepared for all samples, and after the addition of sample buffer, the lysates were boiled for 10 min, centrifuged, and separated by 12.5% SDS-PAGE. Residual labeled proteases in cells were visualized by scanning the gel with a Typhon scanner, with excitation/emission wavelengths of 635/670 nm.

### Evaluation of Probes and Inhibitors in Mice Osteoclast Samples

Cells extracted from mice bone marrow (as mentioned above) were seeded in a six-well plate (5 × 10^6^ cells/well) and treated for 9 days with M-CSF and RANKL (R&D Systems).^53^ After that, the cells were incubated with different inhibitors, followed by incubation with pan-cathepsin probe **GB123**. Cells were lysed, and labeled proteases were visualized by scanning the gel with a Typhon scanner at excitation/emission wavelengths of 635/670 nm.^53^

### Evaluation of Inhibitors in Human Osteoclast on Bone Samples

#### Generation of Primary Human Osteoclasts

Peripheral blood mononuclear cells (PBMCs) were isolated from peripheral blood samples and differentiated to mature osteoclasts (OCs) as follows: whole blood samples were collected into CPT tubes, and monocytes were separated according to the manufacture protocol (BD Vacutainer, 362782). To induce RANK expression 2.5 × 10^7^ PBMCs were seeded in T75 culture flasks supplemented with 25 ng/mL M-CSF (R&D systems, 216-MC) for 2 days and then cells were detached from the flask using Accutase (Sigma, A6964), counted, and reseeded in 96-well plates for either differentiation or bone resorption assays (see below).

#### Bone Resorption Assays

##### Monocyte Isolation from Blood

Blood was taken in an aseptic nontouch technique from consented human volunteers that were bled from the median cubital vein using a 21G needle, by the Declaration of Helsinki. Blood was collected in vacutainers, BD Vacutainer (BD362782), and centrifuged according to the manufacture protocol. Monocytes, 2.5 × 10^7^, were seeded in a 75T flask in α-MEM media (Sigma, M8042) supplemented with 10% FBS, 1% penicillin, 1% glutamine, 1% streptomycin (Biological Industries) (full media), and 25 ng/mL M-CSF (R&D Systems) incubated at 37 °C and 5% CO_2_ for 3 days. Then, 1.8 × 10^5^ cells were seeded per well in 96-well plates (CorningR) on bone slice (BoneslicesR) cultured in α-MEM media in the presence of 25 ng/mL RANKL and 25 ng/mL M-CSF (R&D Systems). Every 2–3 days, the medium was changed with the required amount of M-CSF, RANKL, and different inhibitors: **GD20** (1 or 5 μM), ODN (1 or 5 μM), or vehicle. On the 10th day, cells were fixed with 4% paraformaldehyde (PFA) for 10 min at room temperature, washed three times with PBS and then washed thrice with PBS, and then stained using a TRAP staining kit (Sigma-Aldrich, 387A-1KT) according to the manufacturer’s protocol. At the end point of the resorption experiment, the bone slices were imaged using an Olympus 83× microscope. The whole bone slice was analyzed by generating a panoramic view of the slice using stitching of images taken with an 20× magnification. After the TRAP assay, cells were scraped off the bone slice, and the bone slices were stained with toluidine blue staining (Sigma, T3260), as was conducted by Mukai et al.^54^ And again, bone slice slices were imaged as was described before and counted using ImageJ software. The eroded surface perimeter was manually traced and divided by the total surface area of the slice. For each individual and treatment, 3–5 bone slices were used.

#### Osteoclast’s Differentiation Assay

To assess osteoclast differentiation, 7.5 × 10^4^ cells from PBMCs, cells preincubated with M-CSF, were cultured in 96-well plates. After 3 days, medium was replaced with differentiation medium (αMEM; M8042 sigma, 10% FBS, 5% Penstrep and l-Glu, BI) supplemented with 25 ng/mL M-CSF and 25 ng/mL RANKL (R&D systems, 390-TN) and different inhibitors: **GD20** (1 or 5 μM), ODN (1 or 5 μM), or vehicle. The differentiation medium was changed after 2 and 5 days. Cells were fixed on the 7th day with 4% PFA for 10 min at room temperature, they were washed three times with PBS, and then stained using a TRAP staining kit (Sigma-Aldrich, 387A-1KT) according to the manufacturer’s protocol with additional staining of the nuclei with DAPI. Osteoclast parameters were obtained by analysis of three wells from each- treatment. The osteoclasts were observed with a Nikon Ti microscope (Nikon DS-Fi2 color CCD camera). The total osteoclast surface area was determined using ImageJ software by manually tracing individual osteoclasts perimeters.

#### Evaluation of Inhibitors in Human Osteoclast Sample Using Gel-Based Assays

##### Monocyte Isolation from Blood

Blood was collected in lithium heparin-treated Vacutainers, and RosetteSep was added to enrich for monocytes, followed by density centrifugation with FicollPaque Plus density gradient media. The monocytes were then washed twice and resuspended in PBS, 2 mM ethylenediaminetetraacetic acid (EDTA) supplemented with fetal calf serum (FCS), and counted.

Cells were seeded 3 × 10^6^ per 24-well plate (Primaria, Corning) in 1 mL of RPMI 1640 medium supplemented with 10% FBS and 50 μg/mL gentamicin. The next day, the medium was removed, and the adherent cells were washed with α-MEM and cultured for 14 consecutive days in α-MEM supplemented with 10% FBS, 1% penicillin, 1% streptomycin (Biological Industries), 100 ng/mL RANKL (R&D Systems), and 25 ng/mL M-CSF (R&D Systems) (for osteoclast) or with 25 ng/mL M-CSF and IL-4 (macrophage). Every 3 days, the media was changed with the required amount of M-CSF and RANKL until the giant osteoclasts appeared or with M-CSF and IL-4. After 7 days, **GD20** or vehicle were added to the media and replaced every 3 days along with RANKL and M-CSF, incubated at 37 °C. On the 14th day, **GB123** (5 μM) was added for 2 h at 37 °C. Cells were then washed with PBS and lysed in RIPA buffer and boiled for 10 min and centrifuged. Equal amounts of proteins, 35 μg were separated by 12.5% SDS-PAGE. Labeled proteases were visualized by scanning the gel with a Typhon scanner at excitation/emission wavelengths of 635/670 nm.

#### Fluorescent Imaging of CTSK in Cells

Cells were seeded in 8-well chambers and then treated with an inhibitor or vehicle for 2 h. Cells were then labeled with the indicated probes for the indicated times and then washed with PBS, fixed with cold methanol, and mounted with DAPI-Fluoromount-G (SouthernBiotech, 0100-20) before visualization by confocal microscopy. Zeiss Confocal LSM 980 Microscope with Zen software was used with Airyscan (Germany).

#### Fluorescent Imaging of CTSK in Normal and Cancerous Lung Tissues

Normal and tumor human lung tissues were collected under Helsinki approval. Freshly resected lung cancer and normal lung tissues were embedded in the OCT, frozen in liquid nitrogen, and kept at −80 °C. Tissues were sectioned into 10 μm thick slices using a cryostat (Leica). The slides were defrosted and washed with 1× PBS. Then, the slides were pretreated with vehicle-DMSO, **GD20**, **GD38**, or ODN (5 μM) in buffer acetate for 1 h at 37 °C. Afterward, **GD32** (0.75 μM) or DMSO in reaction buffer (50 mM acetate, 4 mM DTT and 5 mM MgCl_2_, pH 5.5) were applied to the slides for 4 h at 37 °C. Three short washes with 1× PBS were done, and the slides were left in PBS overnight at 4 °C. The next day, slides were mounted with DAPI-Fluoromount-G (SouthernBiotech, 0100-20) before visualization by confocal microscopy, Olympus FV10i. The mean emission of **GD32** (Cy5 filter) intensity inside cells was quantified by NIS Elements software. The **GD32** signal was compared to the signal in normal tissue designated as a 100% ± standard error.

#### CTSK Labeling in Fresh Normal and Cancerous Lung Tissues

Freshl resected human lung samples were cut into small pieces, and the tissues were minced and incubated in a 24-well plate in 1 mL of DMEM supplemented with 10% FBS, 1% Pen Strep, and 1% Glutamine. Normal and tumor samples were each incubated in DMEM at 37 °C with vehicle (DMSO), **GD20**, or **GD38** (10 μM) for 3 h. Then, **GD32** (10 μM) was added to all samples for an additional 17 h. At the end of the incubation, samples were centrifuged at 14,000 rpm for 5 min, and the tissues were washed with PBS. Two volumes of cold RIPA were added to the tissues, and proteins were extracted using a bead homogenizer (Bullet Blender Storm-BBY24M, Next Advance) at speed 10 for 4 min at 4 °C. Proteins were quantified by BCA, Thermo Scientific; an equal quantity of proteins was prepared for all samples, and after the addition of sample buffer, the lysates were boiled for 10 min, centrifuged, and separated by 12.5% SDS-PAGE. Residual labeled proteases in the tissues were visualized by scanning the gel with a Typhon scanner, with excitation/emission wavelengths of 635/670 nm.

## Supporting Information Available

Synthetic procedures, NMR spectra, LC-MS data, and cellular and fluorescence bioimaging data (PDF)
