# Supplementary material for: Development and Application of Small Molecule–Peptide Conjugates as Cathepsin K-Specific Covalent Irreversible Inhibitors in Human Osteoclast and Lung Cancer
Source: JACS Au. 2025 Mar 3;5(3):1104–20. doi: 10.1021/jacsau.4c00840 (PMC11938014; doi:10.1021/jacsau.4c00840)
Supplement: Supplementary file 1 — au4c00840_si_001.pdf [file au4c00840_si_001.pdf]

Supporting Information

Development and Application of Small Molecule Peptide Conjugates as Cathepsin K Specific Covalent Irreversible Inhibitors in Human Osteoclast and Lung Cancer

Gourab Dey,† Evalyn Yakobovich,† Jure Loboda,‡ Reut Sinai-Turyansky,† Chen Abramovitch-Dahan,§ Emmanuelle Merquiol,† Nikhila Sridharan,ǁ Gal Itzhak,† Boris Turk,‡ Ori Wald,⊥ Dusan Turk,‡ Simon Yona,ǁ Noam Levaot,§ and Galia Blum *,†

†The Institute for Drug Research, The School of Pharmacy, The Faculty of Medicine, The Hebrew University, Jerusalem, 911200, Israel

‡Department of Biochemistry and Molecular Biology, J. Stefan Institute, Jamova 3, SI−1000, Ljubljana, Slovenia

§Department of Physiology and Cell Biology Faculty of Health Sciences, Ben−Gurion University of the Negev, Shderot Ben Gurion 1, Beer−Sheva 844394, Israel

ǁThe Institute of Biomedical and Oral Research, The Faculty of Dental Medicine, The Hebrew University of Jerusalem, Jerusalem, 911200, Israel

⊥Department of Cardiothoracic Surgery, Hadassah Hebrew University Medical Center, The Faculty of Medicine, The Hebrew University of Jerusalem, Jerusalem, 911200, Israel

*Email: galiabl@ekmd.huji.ac.il.

Crystal structure of GD20 bound to CTSK:

CTSK was crystallized in hexagonal space group P6122 with two molecules in the asymmetric unit. Both CTSK molecules are well resolved along the whole chain from A1 to M215, as indicated by the absence of Ramachandran plot outliers. The two molecules superimpose to each other with root-mean-square deviations (RMSD) of 0.33 Å over 215 CA atoms. Their positions are related by an approximately two-fold axis (175°and a screw of 1.28 Å). Inhibitors **GD20** binds to both molecules in the same way. In addition, the crystal structure contains a calcium ion that stabilizes the packing of the two molecules forming the asymmetric unit, two chloride ions, and 417 solvent molecules. The two active sites are at the 2-fold non-crystallographic symmetry axes, with Y67 rings positioned between the two inhibitor **GD20** molecules.

Inhibitor **GD20** binds in the non-primed site of the active site of CTSK (Figure 4c and S6) as other inhibitors (Figure S6) (Mons et al. 2019)[1]. Positions of all its non-hydrogen atoms were unambiguously resolved by the electron density maps (Figure S6). The atomic B-factors (mean 32.8 $Å^2$ of inhibitor **GD20** are slightly higher than those of CTSK residues in its vicinity (28.2 $Å^2$), indicating that the inhibitor bound to close to 100% of CTSK molecules in the crystal. The acyloxy moiety of inhibitor **GD20** binds in the S1 subsite of CTSK. The electron density map in Figure S6 shows the carbon atom of the acyloxy methyl group of inhibitor **GD20** is covalently attached to the SG atom of the reactive site Cys25 indicating the sp3 configuration of the C2 atom. The amide links acyloxy methyl moiety to the $S1^1$ subsite fragment. The amide stabilizes the binding by hydrogen bonds formed on both sides - with the carbonyl of N161 on the right and peptidyl nitrogen of G66 on the left. The hydrophobic part of the leucine fragment is bound into the S2 subsite of CTSK in a substrate-like manner. It packs against hydrophobic surfaces of Y67, M68, A134, A163, and L209. The amino protons form another hydrogen bond with the G66 carbonyl. The C11 is a chiral atom to which the trifluoromethyl group, which points into the solvent on the right, and the biphenyl group, which binds along the aromatic surface of two peptide bonds of G65-G66 and N60- D61, are attached. The phenyl rings of both inhibitor **GD20** structures are out of plane (29° and 37°). The terminal SO_2 methyl group faces the solvent and packs against the Y67 side chain from the other CTSK molecule in the asymmetric unit.

Table S1. Data collection and structure refinement statistics

Unit cell	A = 74.37Å, b = 74.37Å, c =336.42Å<br>α = β = 90.0˚, γ = 120.0˚
Space group	P61 2 2 (number 178)
molecules per au	2
wavelenght (Å)	0,97912
Resolution range (Å)	38.51 – 1.99
No. of uniqe reflections	38918

Completeness (last shell) (%)	99.99 (99,8)
Multiplicity	35.6
R meas (last shell) (%)	12.4(71.3)
I/σ	25.3 (5.4)
PDB ID	9G6A
Resolution range (Å)	38.52 – 1.99
No. of reflections in working set	38917
No. of reflections in test set	38917
R-work value	0.1804
R-kick value	0.2056
RMSD Bond lengths (Å)	0.014
RMSD Bond angles (°)	1.66
No. of atoms in au	3822
Protein atoms	3303
Ligands	2
Water molecules	431
PGE	3
Ca^{2+}	4
Mean *B* value (Å^2)	36.3
Ramachandran plot statistics	
Favored	417
Allowed	10
Outliers	0

Data collection, structure determination, and refinement for X-ray diffraction. Diffraction data was collected at the XRD2 beamline at Elettra synchrotron, Trieste[2] under cryogenic conditions. Data were first processed with XDS software[3] and the unmerged HKL file was used as an input for Pointless, Aimless and Ctruncate (CCP4 suite)[4] to obtain the merged MTZ file. The initial CTSK structure was taken from the model 6QBS[1] and refined and rebuilt using MAIN software[5] with maximum likelihood free kick target function.[6] Inhibitor was introduced in the model during refinement and fitted in the | Fo - Fc | map. The geometric restraints for

inhibitor were generated by PURY.[7] The established complex was then further refined. Figures were made with MAIN using RASTER 3D rendering software.[8]

Cell viability assay: U2-OS, U-87 MG, or enriched monocyte cells were grown at 37 ^{0}C with 5% of CO_2 in the respective medium as described previously. Cells were seeded (1500 cells/well in 96 wells plate) one day before the incubation with indicated inhibitors at various concentrations. After 48 h, cells were fixed, and viability was measured by quantitative colorimetric assay using methylene blue according to the procedure reported by Ben-Bassat et al.[9] The data were expressed as a percentage of control; data represent the mean of 2 experiments with triplicates for each treatment (± standard deviation).

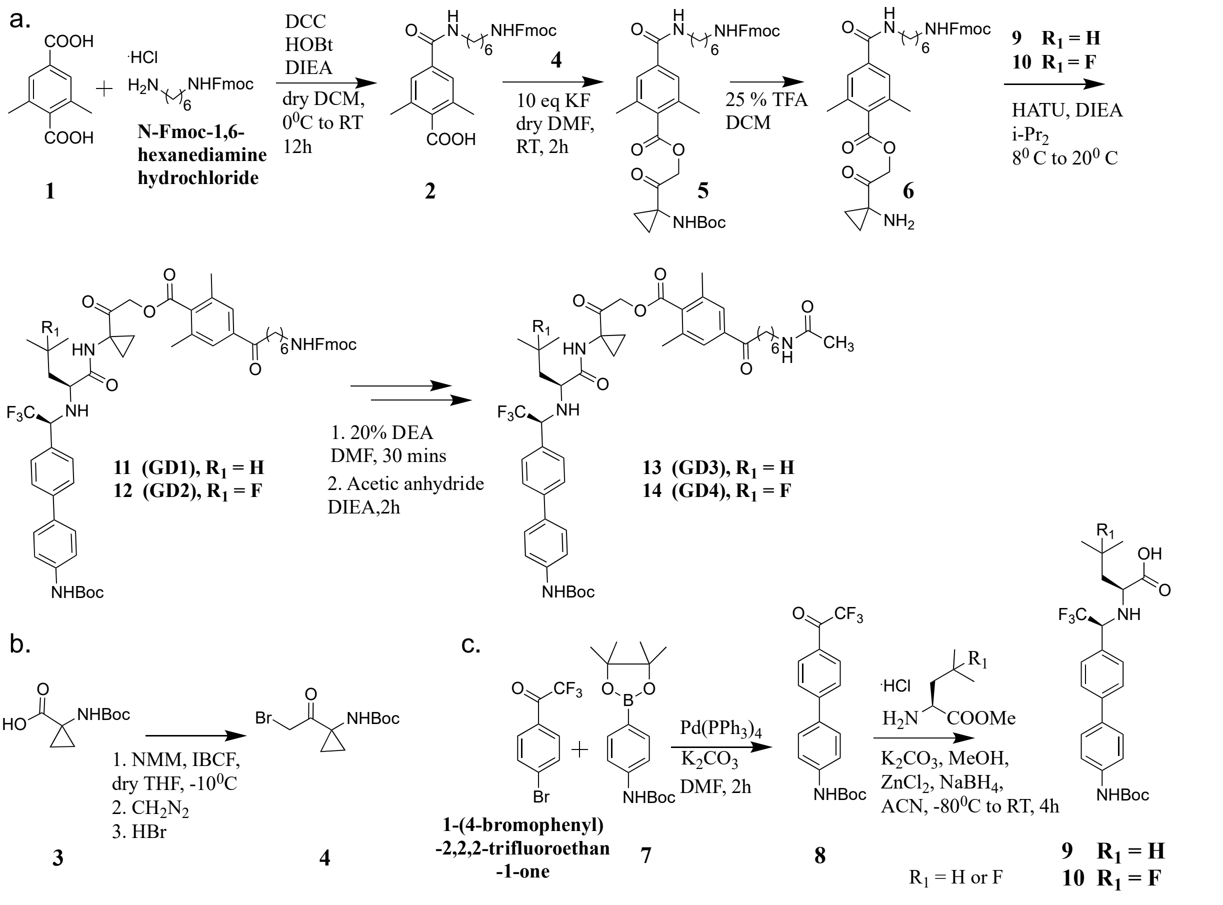

Scheme S1. Synthetic scheme of compound **GD1-GD4.**

(a) Compound **2** was generated by an amide coupling between commercial compounds: 2,6-dimethylterephthalic acid **1** and Fmoc-protected 1,6-hexanediamine chloride. Then **2** was reacted with **4** (which is described in panel (b) below) to generate **5**. The Boc protecting group was removed from **5,** and the free amine was coupled to compound **9** or **10** (described in (c) below) to form **11** and **12** (**GD1** and **GD2,** respectively). Fmoc removal followed by acetylation of **11** and **12** resulted in **13** and **14** (**GD3** and **GD4**, respectively). (b) Compound **4** was synthesized using the diazo coupling reaction of commercially available chemical **3**, followed by acidification with HBr. (c) Compound **8** was synthesized by a Suzuki coupling reaction using the commercially available compound 1-(4-bromophenyl)-2,2,2-trifluoroethan-1-one and **7**. Compound **8** was reacted with the acid chloride salt of leucine or fluoro leucine, followed by diastereospecific reduction using zinc borohydride to synthesize compounds **9** and **10**.

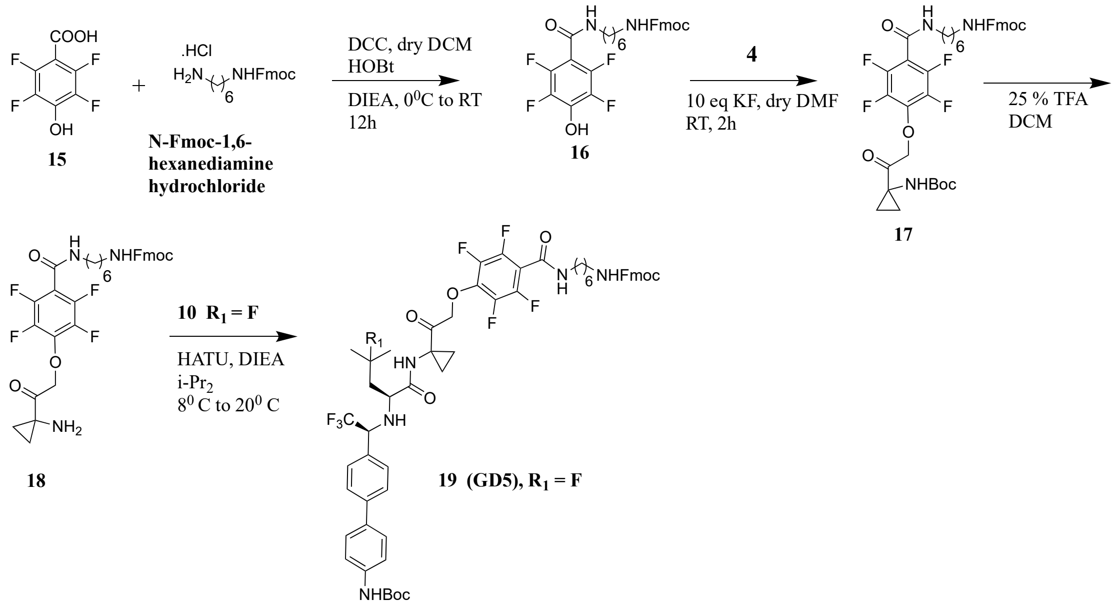

Scheme S2. Synthetic scheme of compound **GD5.**

The synthesis of **GD5** is similar to as described in Scheme S1 using commercial **15** (2,3,5,6 tetrafluoro 4–hydroxy benzoic acid) instead of commercial **1** (2,6-Dimethylterephthalic acid)**.**

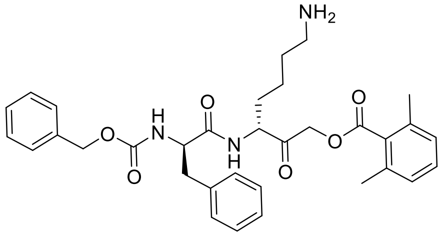

GB111-NH$_2$

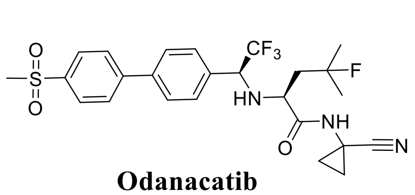

Odanacatib

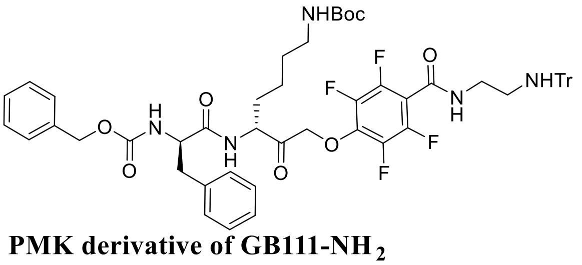

PMK derivative of GB111-NH$_2$

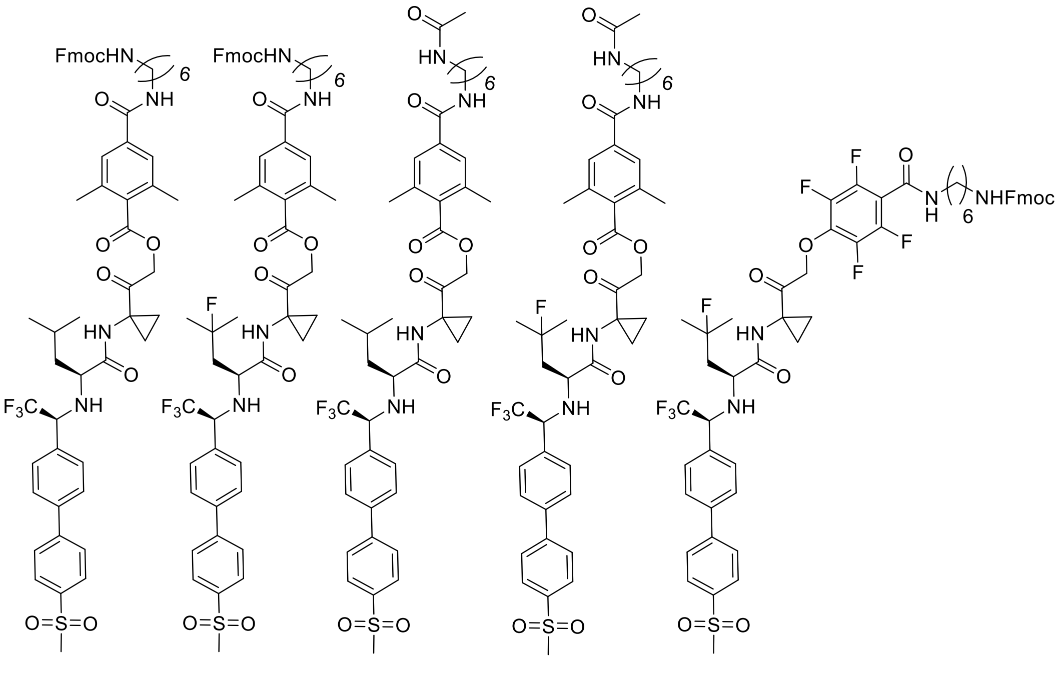

GD1 **GD2** **GD3** **GD4** **GD5**

		Activity		
**Compound**	**Name**	**CTSB**	**CTSL**	**CTSK**
11	**GD1**	>10 µM	>10 µM	>10 µM
12	**GD2**	>10 µM	>10 µM	>10 µM
13	**GD3**	>10 µM	>10 µM	>10 µM
14	**GD4**	>10 µM	>10 µM	>10 µM
19	**GD5**	>10 µM	>10 µM	>10 µM

Figure S1. Chemical structure of **GB111-NH$_2$**, ODN, and PMK derivative of **GB111-NH$_2$**, **GD1 – GD5** with their IC_{50} values towards CTSB, L, and K.

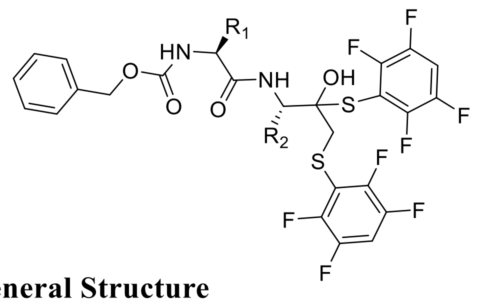

General Structure

Compound	Name	R1<br>(side chain)	R2<br>(side chain)	Activity<br>CTSB	CTSL	CTSK
42	**GD11**	Phe	Lys	5.2 ± 0.8 µM	> 5 µM	8.3 ± 0.9 µM

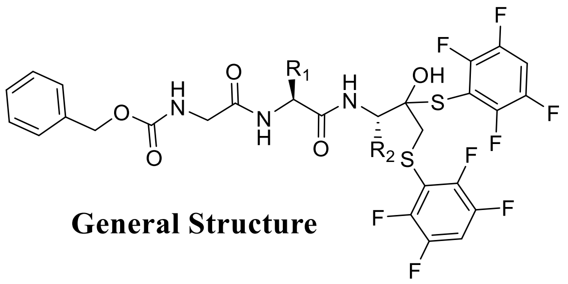

General Structure

Compound	Name	R1<br>(side chain)	R2<br>(side chain)	Activity<br>CTSB	CTSL	CTSK
55	**GD18**	Pro	Lys	> 5 µM	> 5 µM	2 ± 0.3 µM

Figure S2. Chemical structure of compounds **GD11 and GD18** with their potency towards CTSB, CTSL, and CTSK.

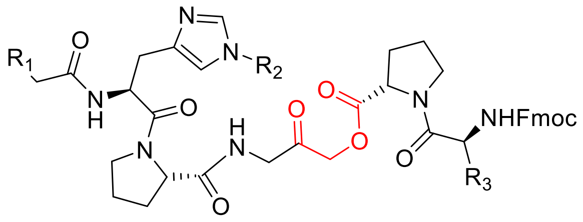

General Structure

**Compound**	**GD**	**R_1**	**R_2**	**R_3** (Side Chain)	**CTSB**	**CTSL**	**CTSK**
64	**19**	NH_2	H	Asp	6.6 ± 0.35 μM	> 5 μM	> 5 μM
66	**22**	NH_2	H	Gln	4 ± 0.3 μM	3.1 ± 0.35 μM	> 10 μM
70	**23**	NHBoc	Trt	Leu	> 5 μM	> 5 μM	> 10 μM
71	**24**	NH_2	H	Leu	0.69 ± 0.16 μM	0.69 ± 0.13 μM	> 5 μM

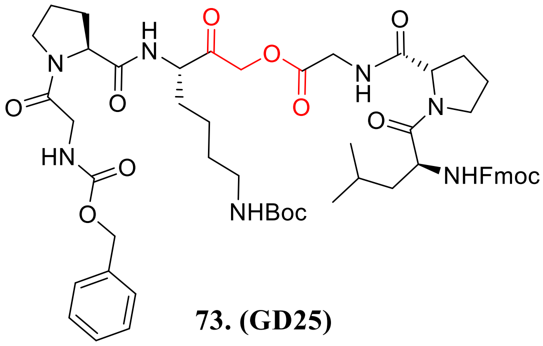

73. (GD25)

**Compound**	**GD**	**CTSB**	**CTSL**	**CTSK**
73	**25**	> 5 μM	> 5 μM	5.4 ± 0.1 μM

Figure S3. Chemical structure of compounds **GD19, GD22- GD25** with their potency towards CTSB, CTSL, and CTSK.

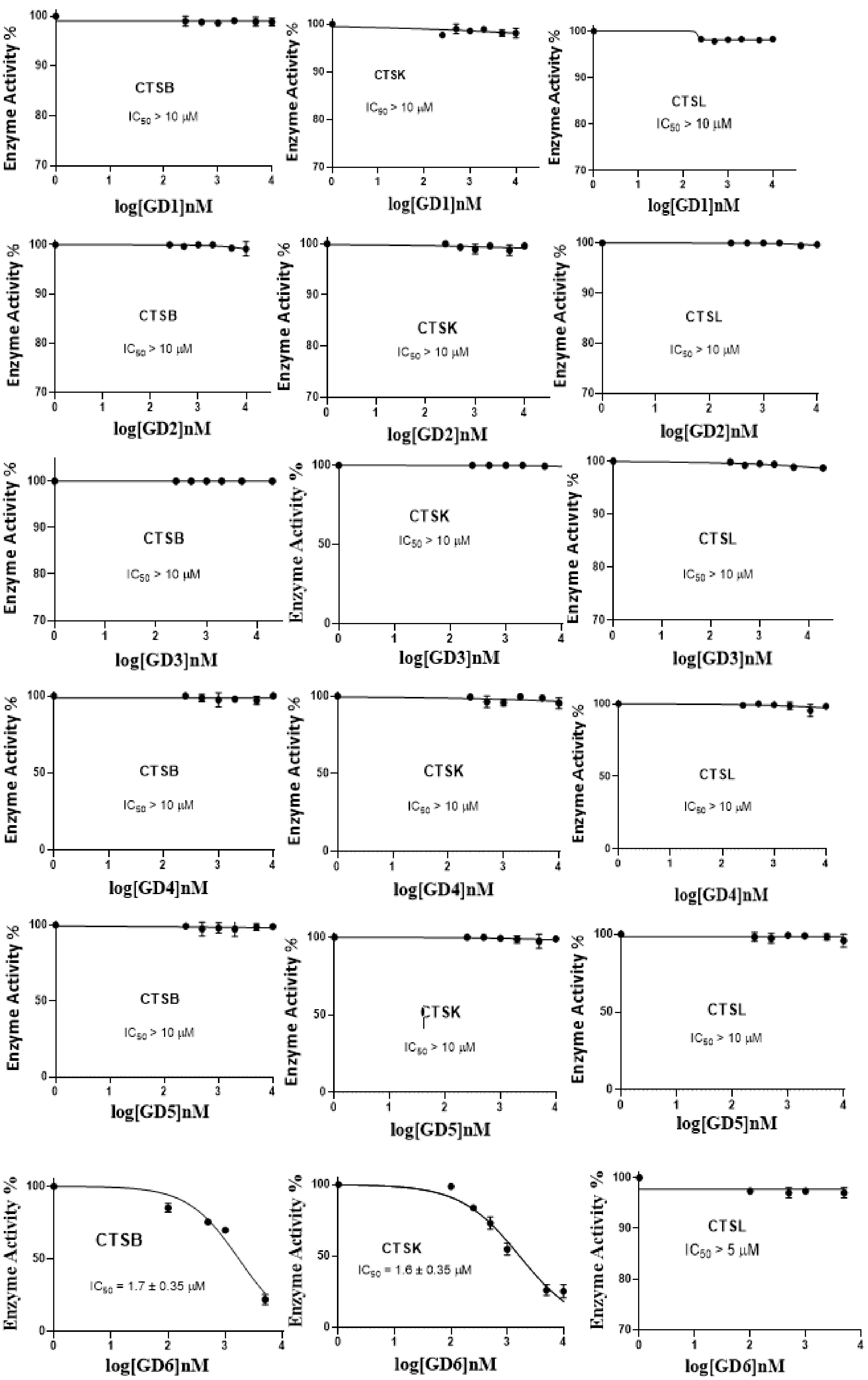

Enzyme Activity %
CTSB
IC50 > 10 μM
log[GD1]nM
CTSK
IC50 > 10 μM
log[GD1]nM
CTSL
IC50 > 10 μM
log[GD1]nM
CTSB
IC50 > 10 μM
log[GD2]nM
CTSK
IC50 > 10 μM
log[GD2]nM
CTSL
IC50 > 10 μM
log[GD2]nM
CTSB
IC50 > 10 μM
log[GD3]nM
CTSK
IC50 > 10 μM
log[GD3]nM
CTSL
IC50 > 10 μM
log[GD3]nM
CTSB
IC50 > 10 μM
log[GD4]nM
CTSK
IC50 > 10 μM
log[GD4]nM
CTSL
IC50 > 10 μM
log[GD4]nM
CTSB
IC50 > 10 μM
log[GD5]nM
CTSK
IC50 > 10 μM
log[GD5]nM
CTSL
IC50 > 10 μM
log[GD5]nM
CTSB
IC50 = 1.7 ± 0.35 μM
log[GD6]nM
CTSK
IC50 = 1.6 ± 0.35 μM
log[GD6]nM
CTSL
IC50 > 5 μM
log[GD6]nM

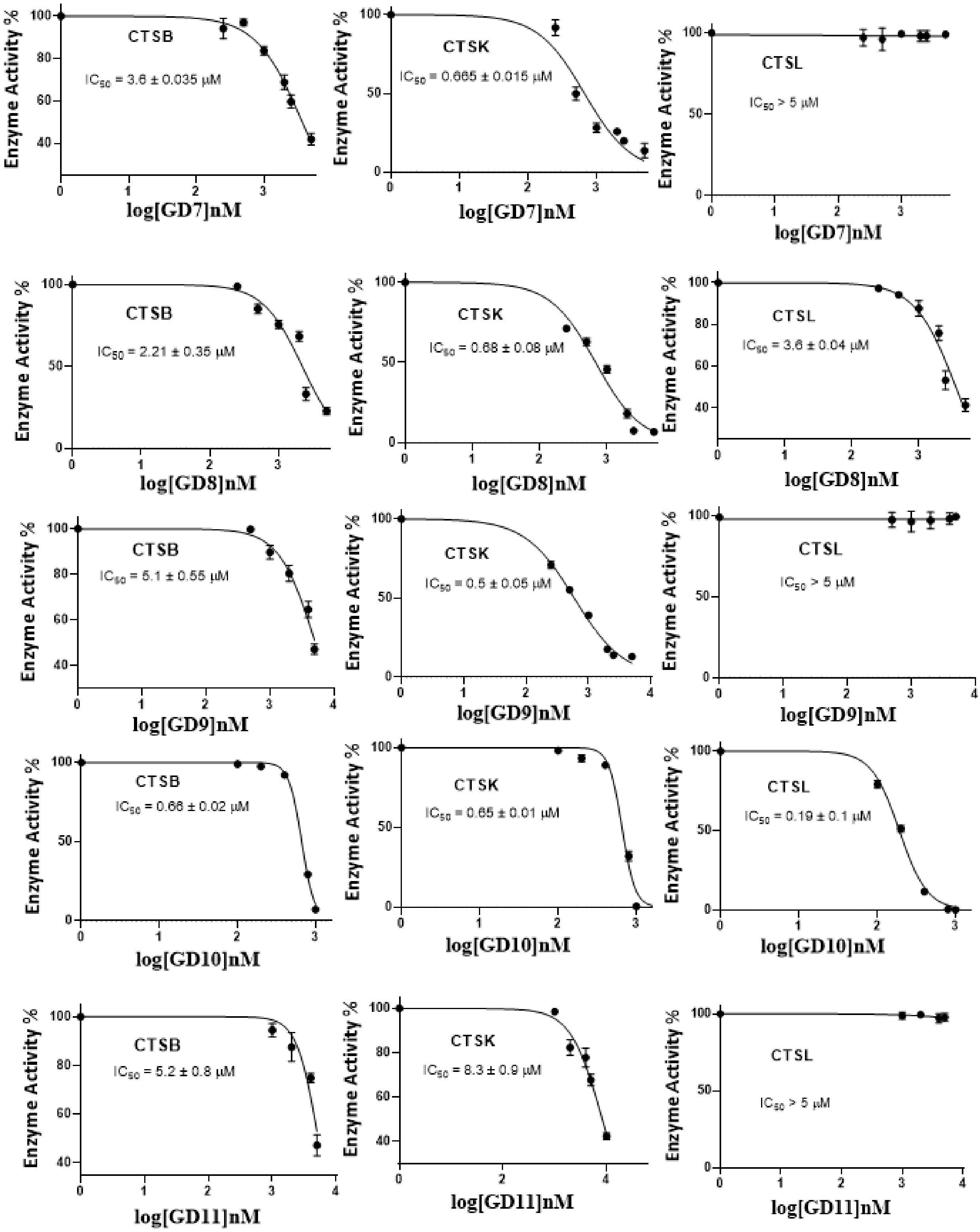

CTSB
IC50 = 3.6 ± 0.035 μM
log[GD7]nM
CTSK
IC50 = 0.665 ± 0.015 μM
log[GD7]nM
CTSL
IC50 > 5 μM
log[GD7]nM
CTSB
IC50 = 2.21 ± 0.35 μM
log[GD8]nM
CTSK
IC50 = 0.68 ± 0.08 μM
log[GD8]nM
CTSL
IC50 = 3.6 ± 0.04 μM
log[GD8]nM
CTSB
IC50 = 5.1 ± 0.55 μM
log[GD9]nM
CTSK
IC50 = 0.5 ± 0.05 μM
log[GD9]nM
CTSL
IC50 > 5 μM
log[GD9]nM
CTSB
IC50 = 0.66 ± 0.02 μM
log[GD10]nM
CTSK
IC50 = 0.65 ± 0.01 μM
log[GD10]nM
CTSL
IC50 = 0.19 ± 0.1 μM
log[GD10]nM
CTSB
IC50 = 5.2 ± 0.8 μM
log[GD11]nM
CTSK
IC50 = 8.3 ± 0.9 μM
log[GD11]nM
CTSL
IC50 > 5 μM
log[GD11]nM
Enzyme Activity %

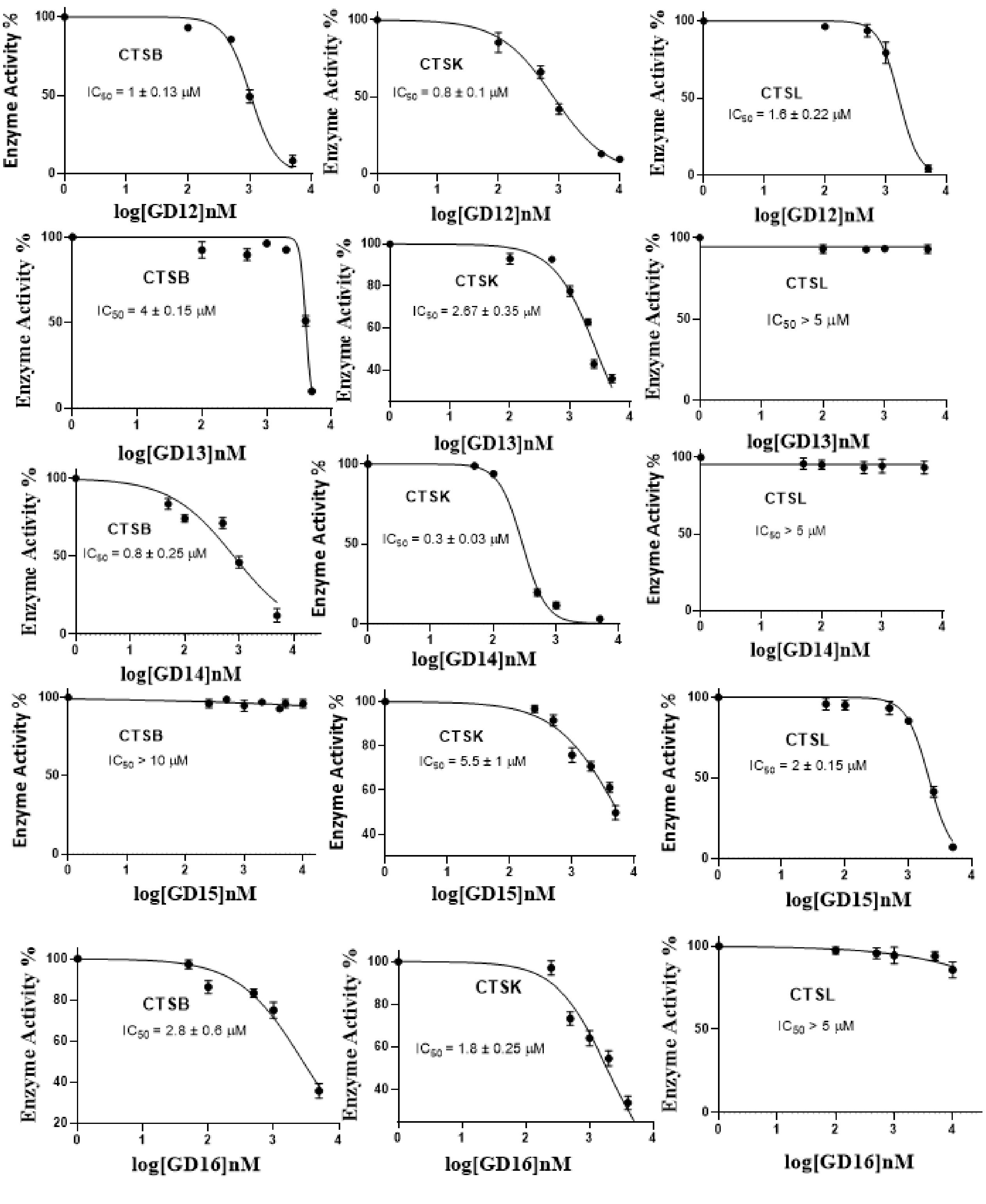
Enzyme Activity %
CTSB
IC_{50} = 1 ± 0.13 μM
log[GD12]nM
CTSK
IC_{50} = 0.8 ± 0.1 μM
log[GD12]nM
CTSL
IC_{50} = 1.6 ± 0.22 μM
log[GD12]nM
CTSB
IC_{50} = 4 ± 0.15 μM
log[GD13]nM
CTSK
IC_{50} = 2.67 ± 0.35 μM
log[GD13]nM
CTSL
IC_{50} > 5 μM
log[GD13]nM
CTSB
IC_{50} = 0.8 ± 0.25 μM
log[GD14]nM
CTSK
IC_{50} = 0.3 ± 0.03 μM
log[GD14]nM
CTSL
IC_{50} > 5 μM
log[GD14]nM
CTSB
IC_{50} > 10 μM
log[GD15]nM
CTSK
IC_{50} = 5.5 ± 1 μM
log[GD15]nM
CTSL
IC_{50} = 2 ± 0.15 μM
log[GD15]nM
CTSB
IC_{50} = 2.8 ± 0.6 μM
log[GD16]nM
CTSK
IC_{50} = 1.8 ± 0.25 μM
log[GD16]nM
CTSL
IC_{50} > 5 μM
log[GD16]nM

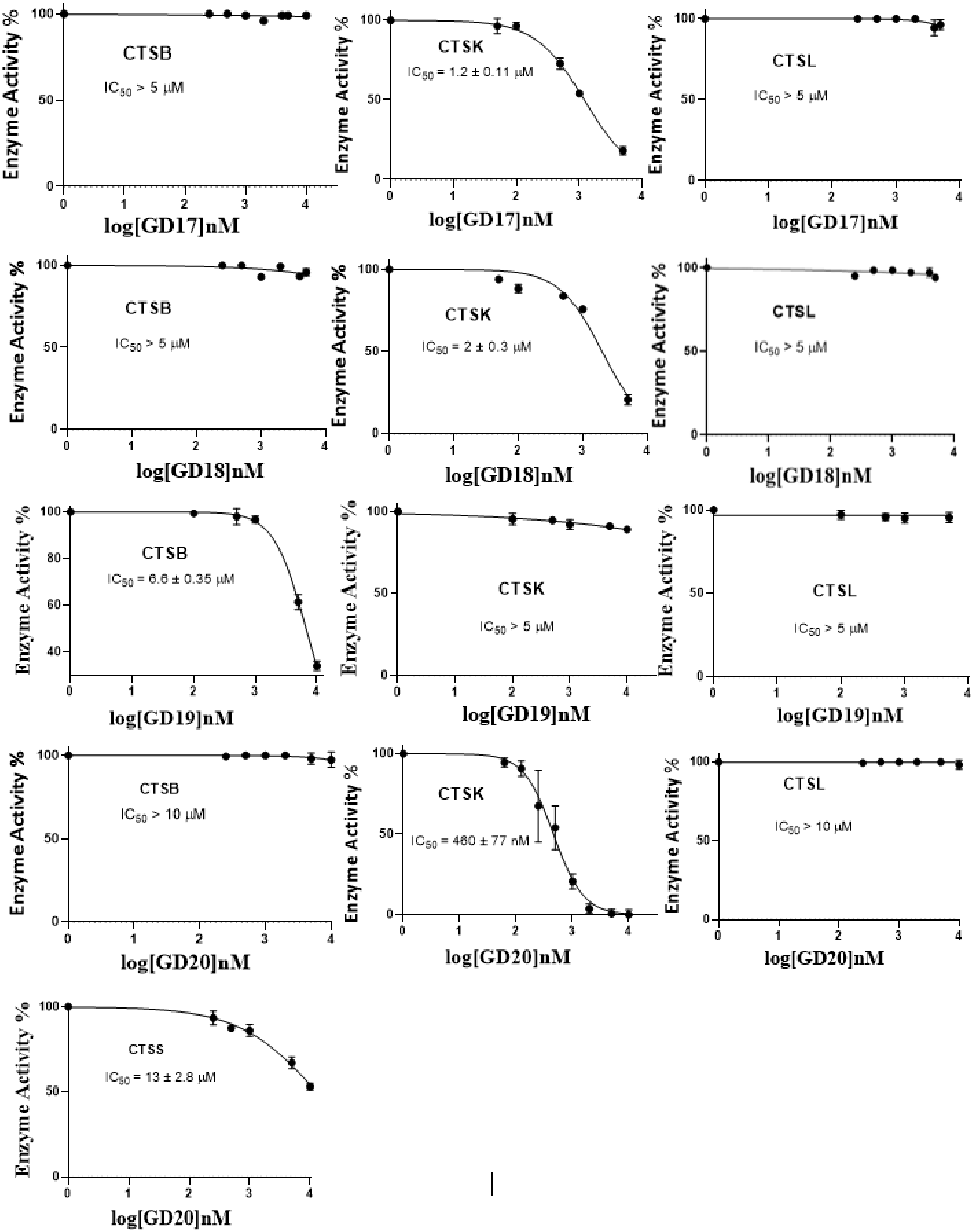

Enzyme Activity %
CTSB
IC50 > 5 μM
log[GD17]nM
CTSK
IC50 = 1.2 ± 0.11 μM
log[GD17]nM
CTSL
IC50 > 5 μM
log[GD17]nM
CTSB
IC50 > 5 μM
log[GD18]nM
CTSK
IC50 = 2 ± 0.3 μM
log[GD18]nM
CTSL
IC50 > 5 μM
log[GD18]nM
CTSB
IC50 = 6.6 ± 0.35 μM
log[GD19]nM
CTSK
IC50 > 5 μM
log[GD19]nM
CTSL
IC50 > 5 μM
log[GD19]nM
CTSB
IC50 > 10 μM
log[GD20]nM
CTSK
IC50 = 460 ± 77 nM
log[GD20]nM
CTSL
IC50 > 10 μM
log[GD20]nM
CTSS
IC50 = 13 ± 2.8 μM
log[GD20]nM

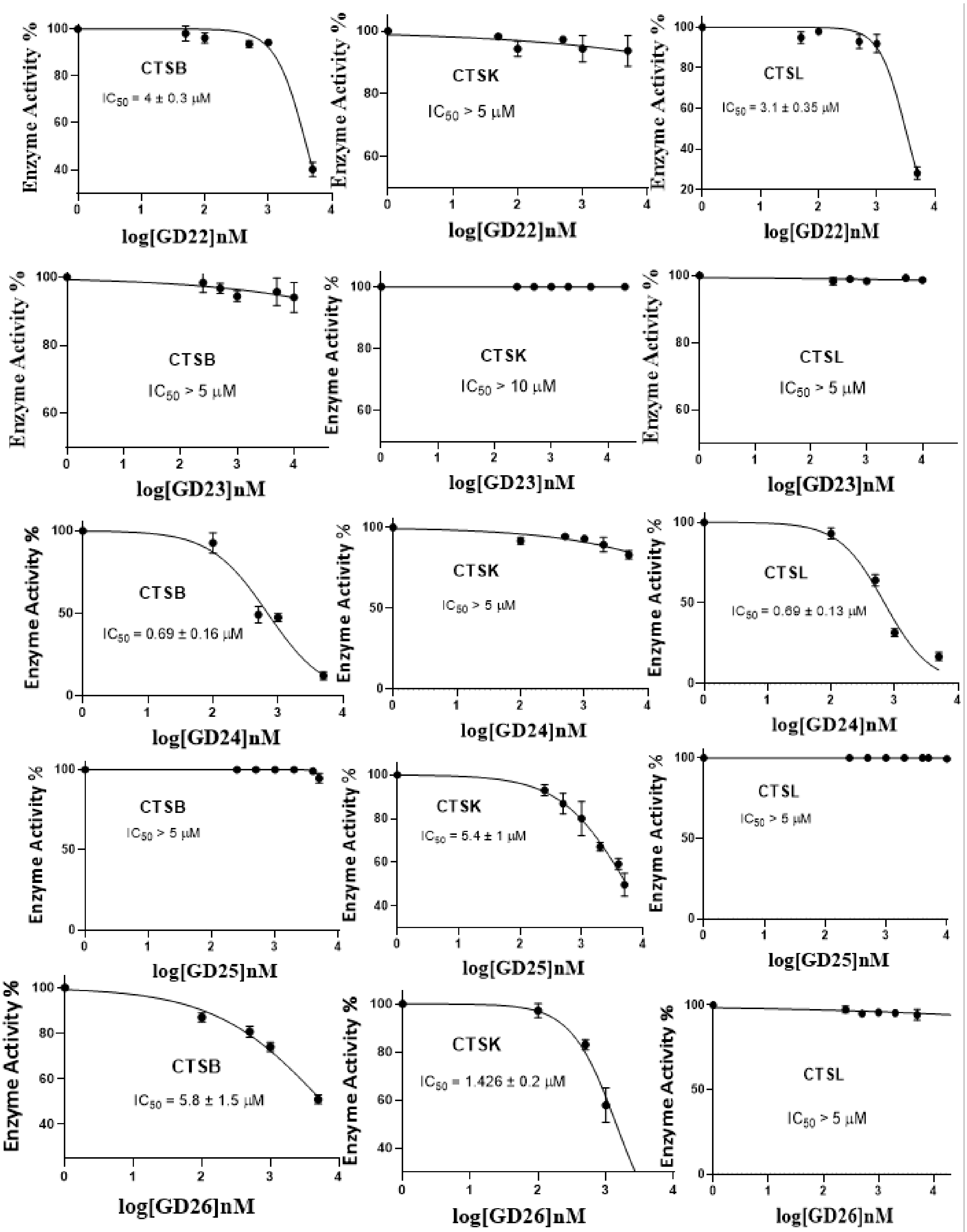
CTSB
IC50 = 4 ± 0.3 μM
CTSK
IC50 > 5 μM
CTSL
IC50 = 3.1 ± 0.35 μM
log[GD22]nM
CTSB
IC50 > 5 μM
CTSK
IC50 > 10 μM
CTSL
IC50 > 5 μM
log[GD23]nM
CTSB
IC50 = 0.69 ± 0.16 μM
CTSK
IC50 > 5 μM
CTSL
IC50 = 0.69 ± 0.13 μM
log[GD24]nM
CTSB
IC50 > 5 μM
CTSK
IC50 = 5.4 ± 1 μM
CTSL
IC50 > 5 μM
log[GD25]nM
CTSB
IC50 = 5.8 ± 1.5 μM
CTSK
IC50 = 1.426 ± 0.2 μM
CTSL
IC50 > 5 μM
log[GD26]nM
Enzyme Activity %

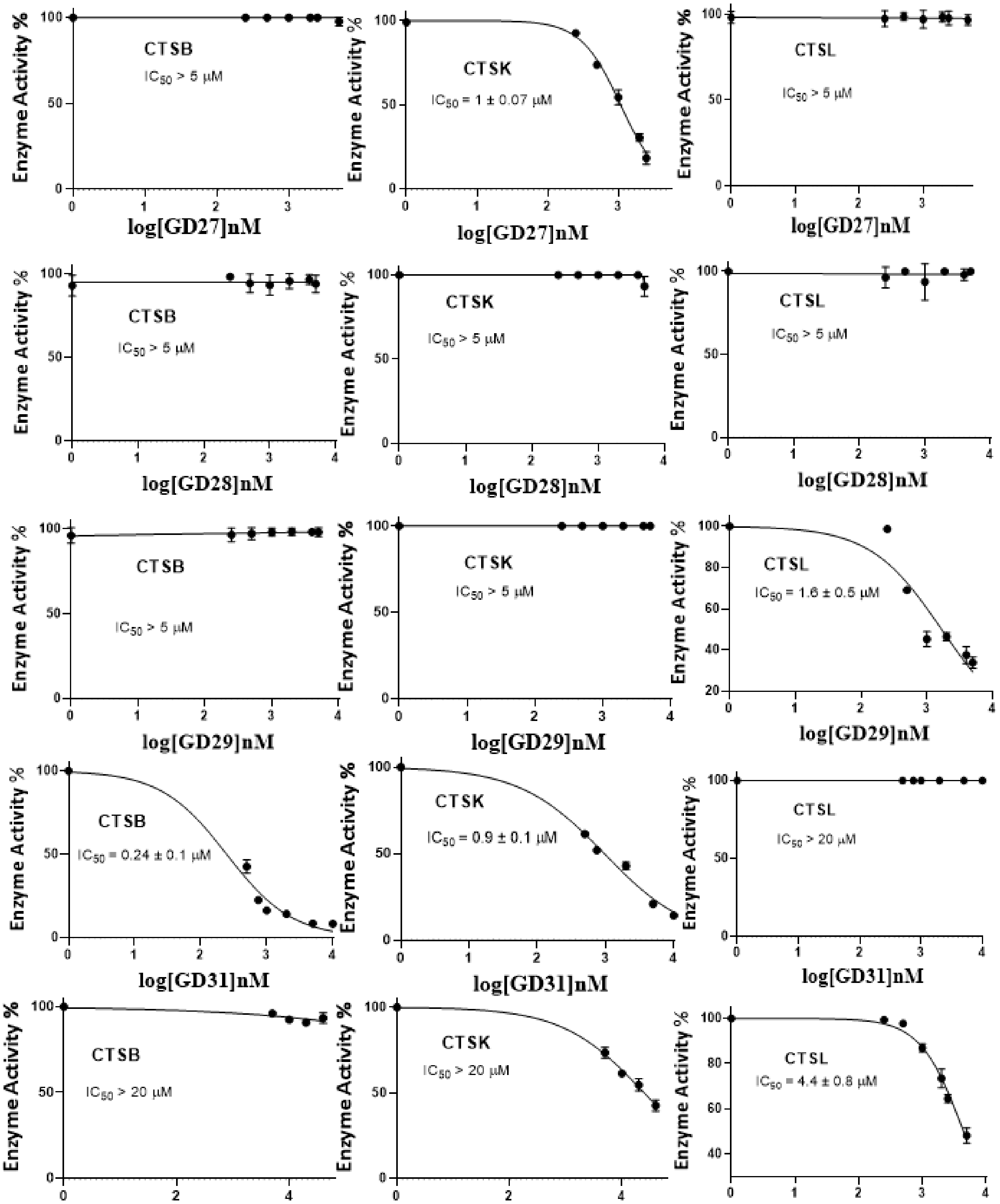
Enzyme Activity %
CTSB
IC50 > 5 μM
log[GD27]nM
CTSK
IC50 = 1 ± 0.07 μM
log[GD27]nM
CTSL
IC50 > 5 μM
log[GD27]nM
CTSB
IC50 > 5 μM
log[GD28]nM
CTSK
IC50 > 5 μM
log[GD28]nM
CTSL
IC50 > 5 μM
log[GD28]nM
CTSB
IC50 > 5 μM
log[GD29]nM
CTSK
IC50 > 5 μM
log[GD29]nM
CTSL
IC50 = 1.6 ± 0.5 μM
log[GD29]nM
CTSB
IC50 = 0.24 ± 0.1 μM
log[GD31]nM
CTSK
IC50 = 0.9 ± 0.1 μM
log[GD31]nM
CTSL
IC50 > 20 μM
log[GD31]nM
CTSB
IC50 > 20 μM
log[GD33]nM
CTSK
IC50 > 20 μM
log[GD33]nM
CTSL
IC50 = 4.4 ± 0.8 μM
log[GD33]nM

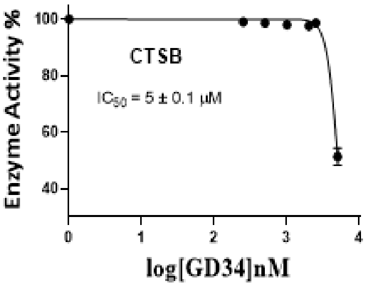
CTSB
IC_{50} = 5 ± 0.1 μM
Enzyme Activity %
log[GD34]nM

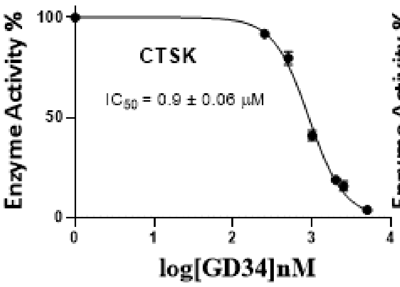
CTSK
IC_{50} = 0.9 ± 0.06 μM
Enzyme Activity %
log[GD34]nM

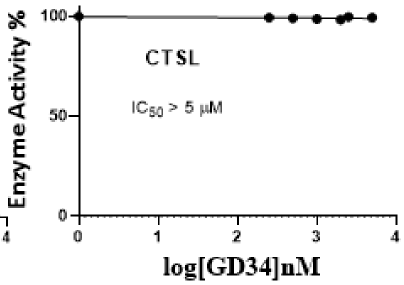
CTSL
IC_{50} > 5 μM
Enzyme Activity %
log[GD34]nM

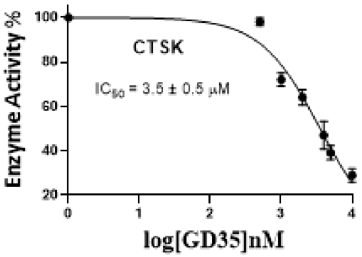
CTSK
IC_{50} = 3.5 ± 0.5 μM
Enzyme Activity %
log[GD35]nM

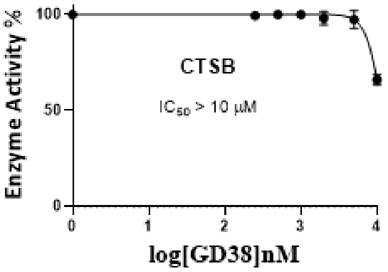
CTSB
IC_{50} > 10 μM
Enzyme Activity %
log[GD38]nM

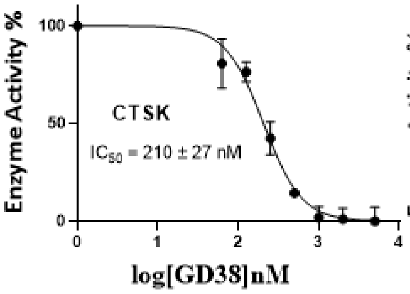
CTSK
IC_{50} = 210 ± 27 nM
Enzyme Activity %
log[GD38]nM

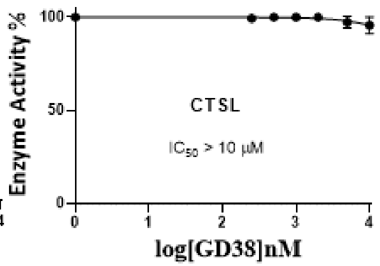
CTSL
IC_{50} > 10 μM
Enzyme Activity %
log[GD38]nM

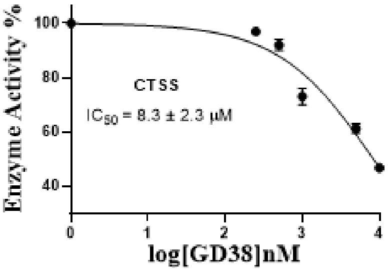
CTSS
IC_{50} = 8.3 ± 2.3 μM
Enzyme Activity %
log[GD38]nM

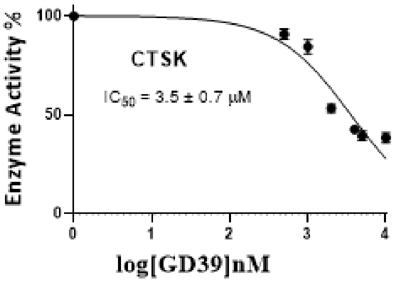
CTSK
IC_{50} = 3.5 ± 0.7 μM
Enzyme Activity %
log[GD39]nM

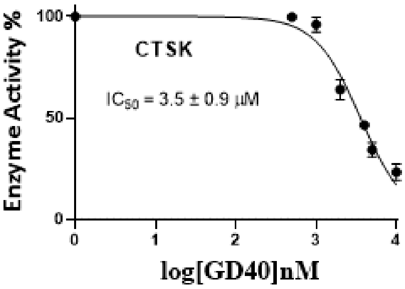
CTSK
IC_{50} = 3.5 ± 0.9 μM
Enzyme Activity %
log[GD40]nM

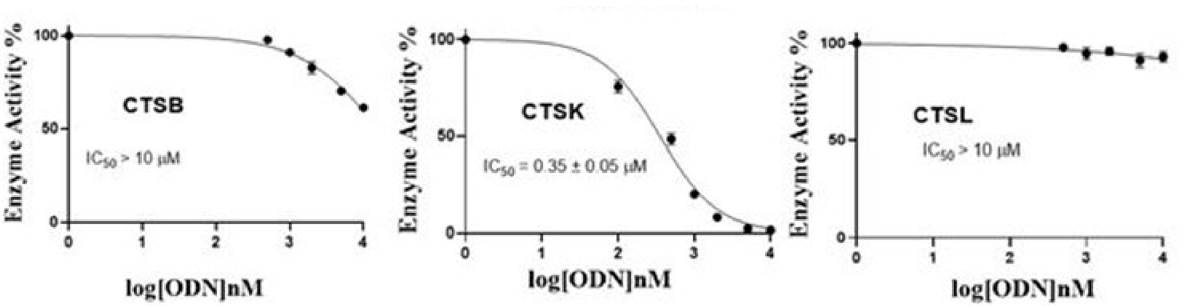

Figure S4. IC_{50} curves of compounds. The IC_{50} curves were generated from the results of three independent competition gel-based experiments. The spots represent an average ± SD. The graphs were generated in GraphPad Prism software.

Table S2. Structure and IC_{50} of compounds.

Compound	Structure	CTSB (IC_{50})	CTSL (IC_{50})	CTSK (IC_{50})
**ODN**	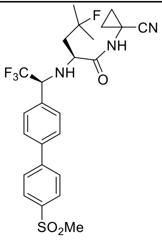	> 10 μM	> 10 μM	350 ± 0.05 nM
**GD1**	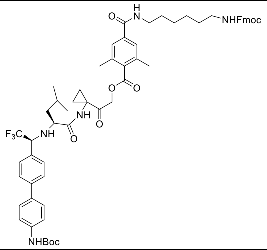	> 10 μM	> 10 μM	> 10 μM
**GD2**	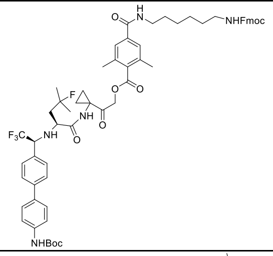	> 10 μM	> 10 μM	> 10 μM
**GD3**	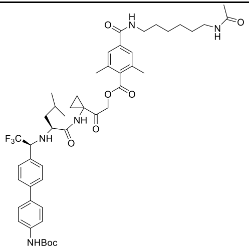	> 10 μM	> 10 μM	> 10 μM

**GD4**	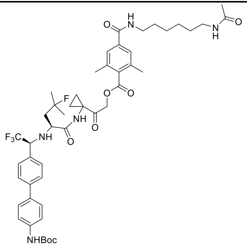	> 10 µM	> 10 µM	> 10 µM
**GD5**	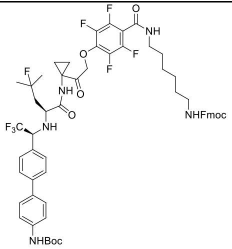	> 10 µM	> 10 µM	> 10 µM
**GD6**	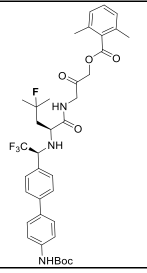	1.7 ± 0.35 µM	> 5 µM	1.6 ± 0.35 µM

**GD7**	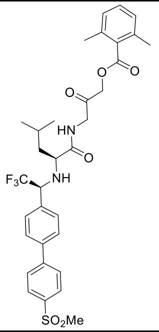	3.6 ± 0.03 µM	> 5 µM	0.66 ± 0.01 µM
**GD8**	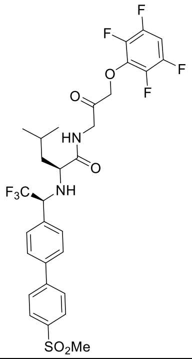	2.2 ± 0.35 µM	3.6 ± 0.04 µM	0.68 ± 0.08 µM

**GD9**	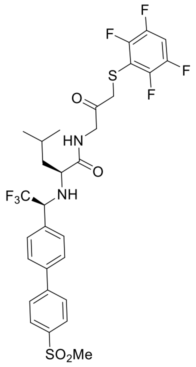	5.1 ± 0.55 µM	> 5 µM	0.5 ± 0.05 µM
**GD10**	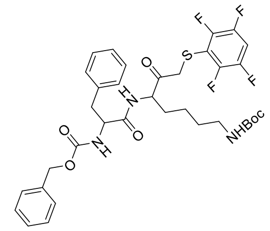	0.66 ± 0.02 µM	0.19 ± 0.1 µM	0.65 ± 0.01µM
**GD11**	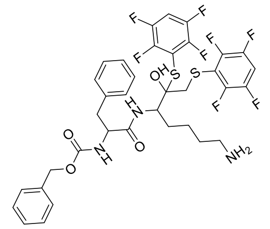	5.2 ± 0.8 µM	> 5 µM	8.3 ± 0.9 µM
**GD12**	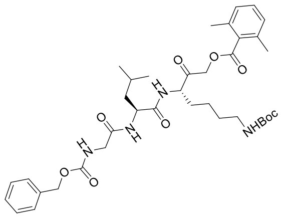	1 ± 0.13 µM	1.6 ± 0.22 µM	0.8 ± 0.1 µM

**GD13**	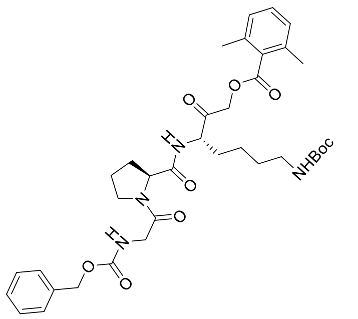	4.0 ± 0.15 µM	> 5 µM	2.7 ± 0.35 µM
**GD14**	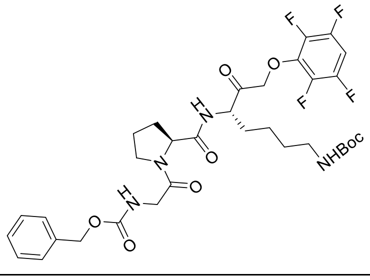	0.8 ± 0.25 µM	> 5 µM	0.3 ± 0.03 µM

**GD15**	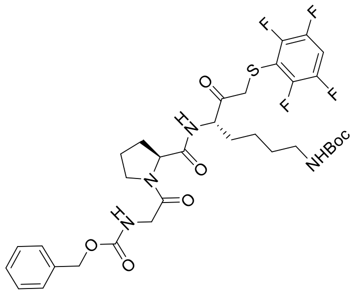	> 5 µM	2.0 ± 0.15 µM	5.5 ± 1 µM
**GD16**	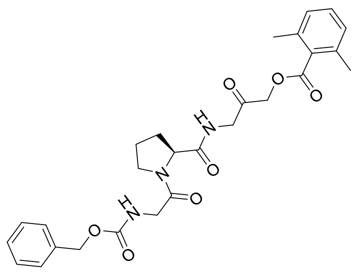	2.8 ± 0.6 µM	> 5 µM	1.8 ± 0.25 µM
**GD17**	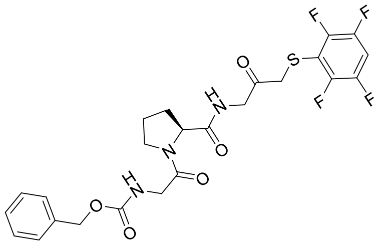	> 5 µM	> 5 µM	1.2 ± 0.11 µM

**GD18**	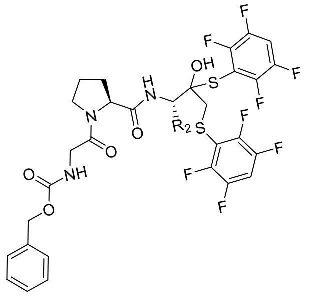	> 5 µM	> 5 µM	2 ± 0.3 µM
**GD19**	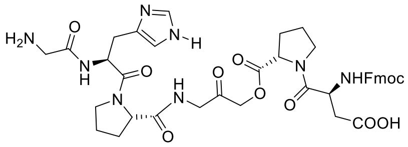	6.6 ± 0.35 µM	> 5 µM	> 5 µM
**GD20**	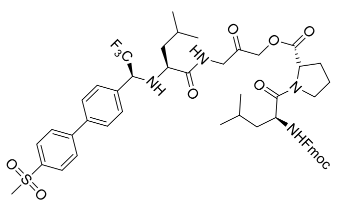	> 10 µM	> 10 µM	0.46 ± 0.07 µM
**GD22**	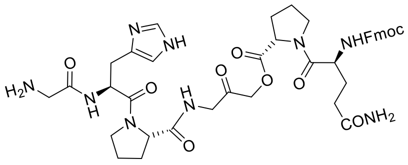	4 ± 0.3 µM	3.1 ± 0.35 µM	> 5 µM

**GD23**	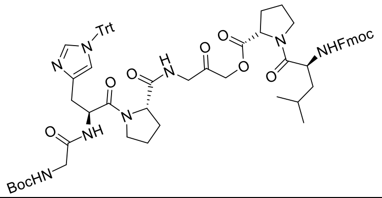	> 5 µM	> 5 µM	> 10 µM
**GD24**	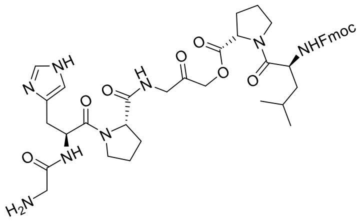	0.69 ± 0.16 µM	0.69 ± 0.1 µM	> 5 µM
**GD25**	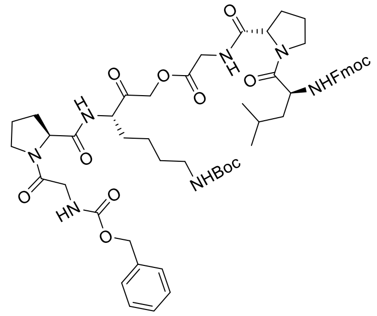	> 5 µM	> 5 µM	5.4 ± 0.1 µM
**GD26**	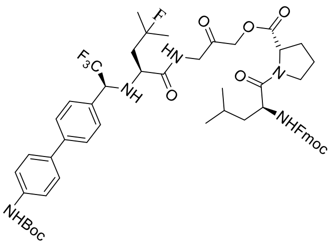	6 ± 1.5 µM	> 5 µM	1.4± 0.2 µM
**GD27**	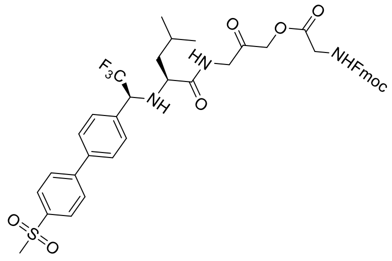	> 5 µM	> 5 µM	1 ± 0.07 µM
**GD28**	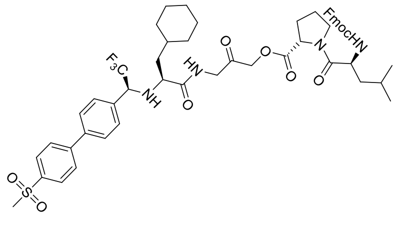	> 5 µM	> 5 µM	> 5 µM

**GD29**	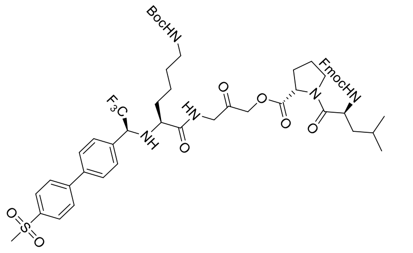	> 5 µM	1.6 ± 0.5 µM	> 5 µM
**GD31**	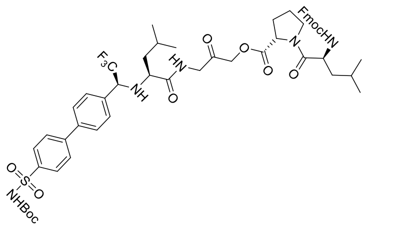	0.24 ± 0.1 µM	> 20 µM	0.9 ± 0.1 µM
**GD33**	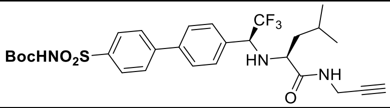	> 20 µM	4.4 ± 0.8 µM	> 20 µM
**GD34**	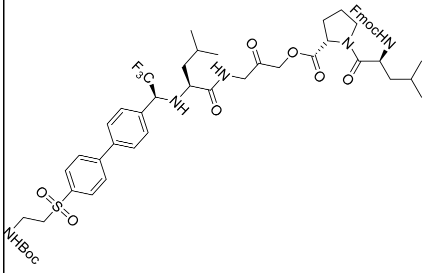	5 ± 0.1 µM	> 5 µM	0.9 ± 0.06 µM
**GD35**	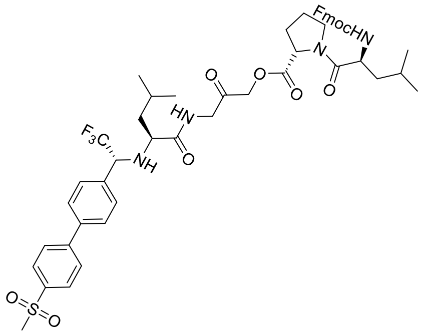			3.5 ± 0.5 µM

**GD38**	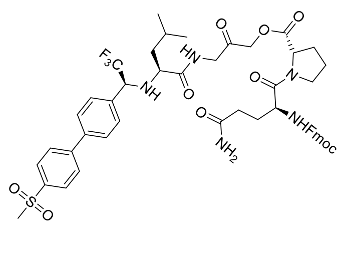	> 10 µM	> 10 µM	0.2 ± 0.02 µM
**GD39**	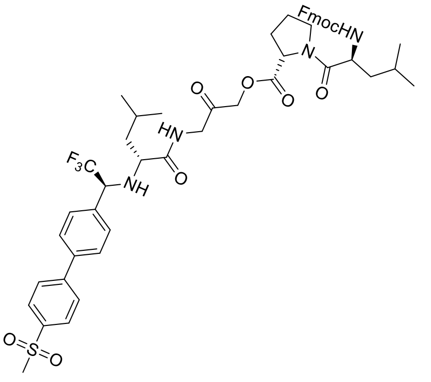			3.9 ± 0.7 µM
**GD40**	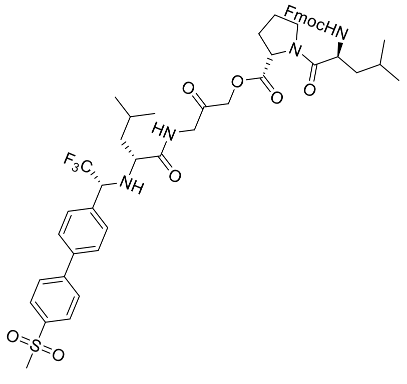			3.5 ± 0.9 µM
**GB111-NH$_2$**	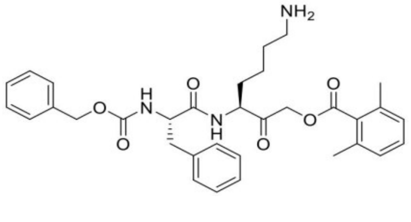	0.40 ± 0.06 µM	0.25 ± 0.04 µM	4.4 ± 0.9 µM

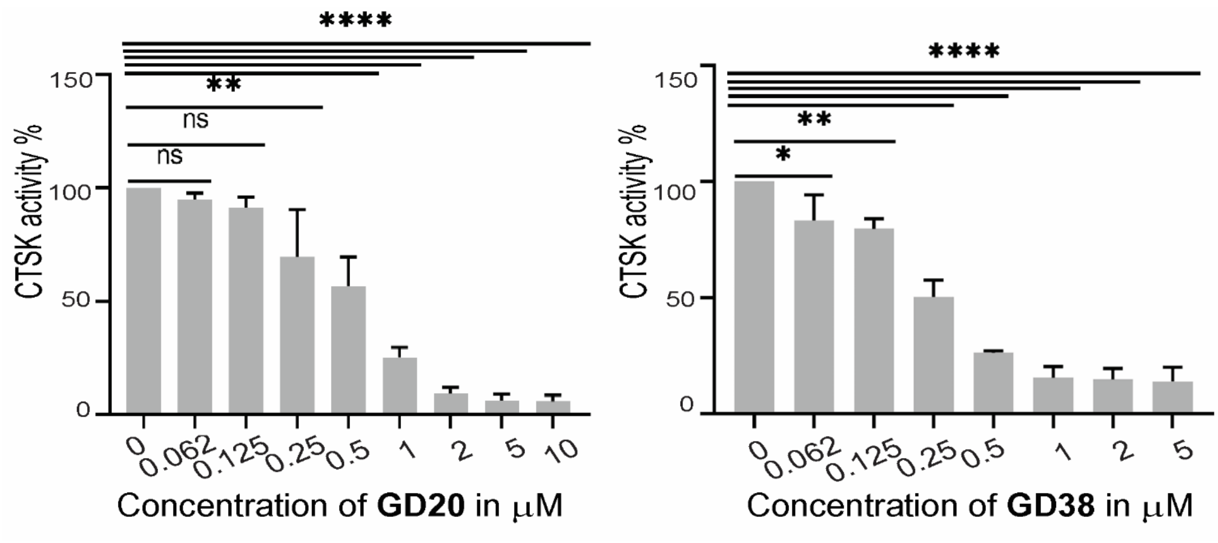

Figure S5. Quantitative analysis of CTSK inhibition by **GD20** and **GD38**. Data represent mean values ± standard deviation, n = 3.

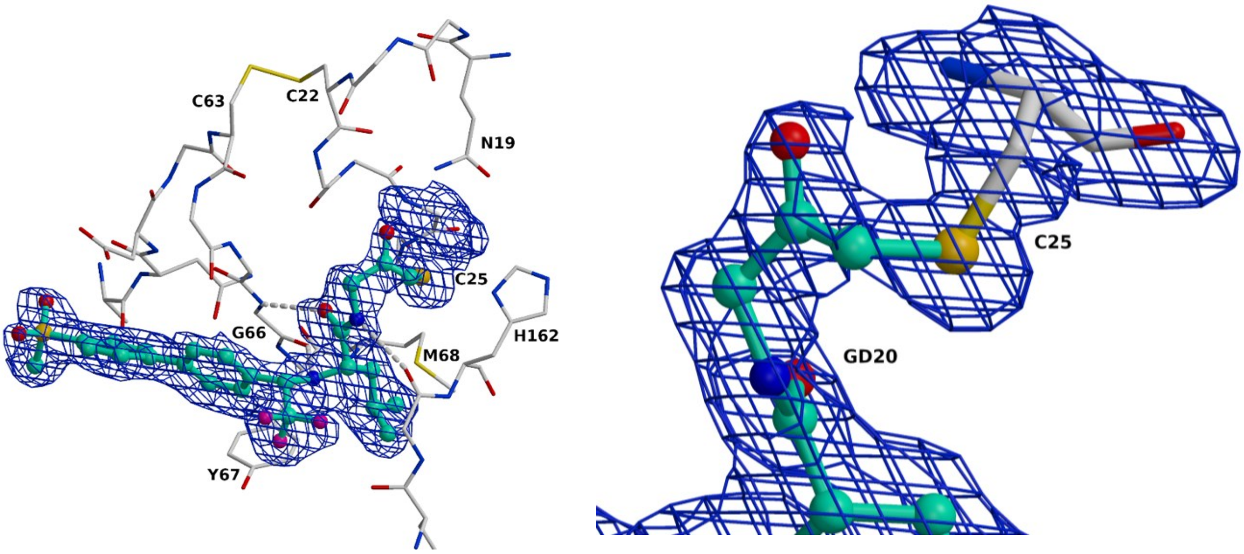

Figure S6. Structure and electron density of **GD20** in complex with CTSK bound to the active site Cysteine **25**.

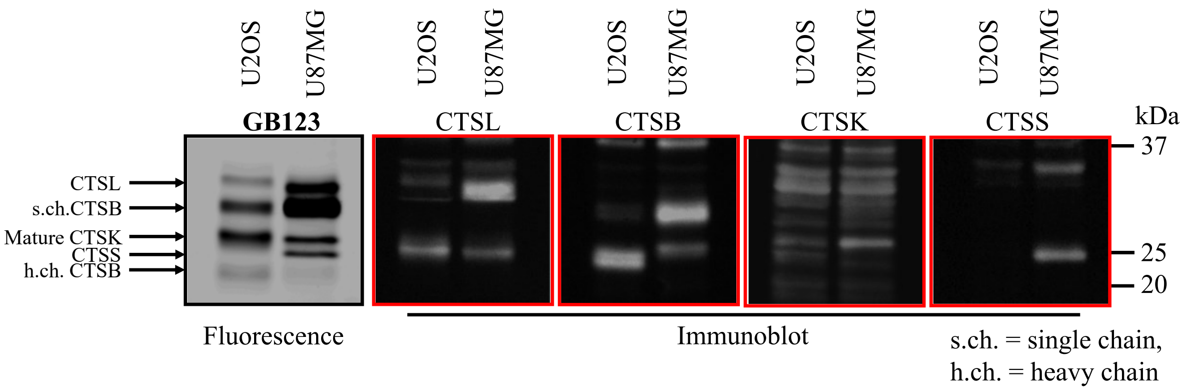

Figure S7. Identification of fluorescent band identity. Endogenous cathepsins in U2-OS and U87-MG cells were labeled by **GB123** (a fluorescent activity-based probe), and cell lysates were separated by SDS PAGE. Gels were scanned for fluorescence and then underwent immunoblotting with different cathepsin-specific antibodies. This experiment enabled identifying which bands originate from which cathepsin.

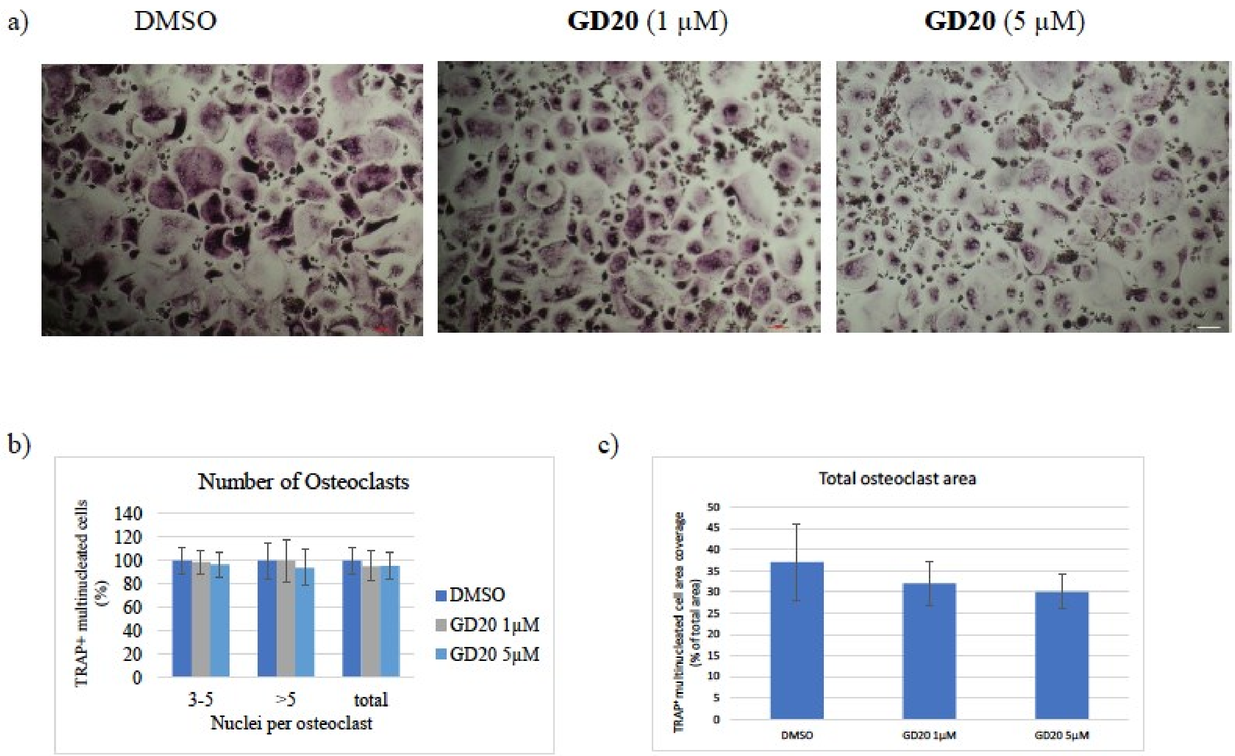

Figure S8. a) Representative pictures of Trap-strained human osteoclasts grown on plastic wells treated with indicated concentrations of **GD20** for 7 days. The scale bar is 100 µm. b)

Quantification of the total number of osteoclasts, and c) osteoclast-cell area in each treatment of the inhibitor.

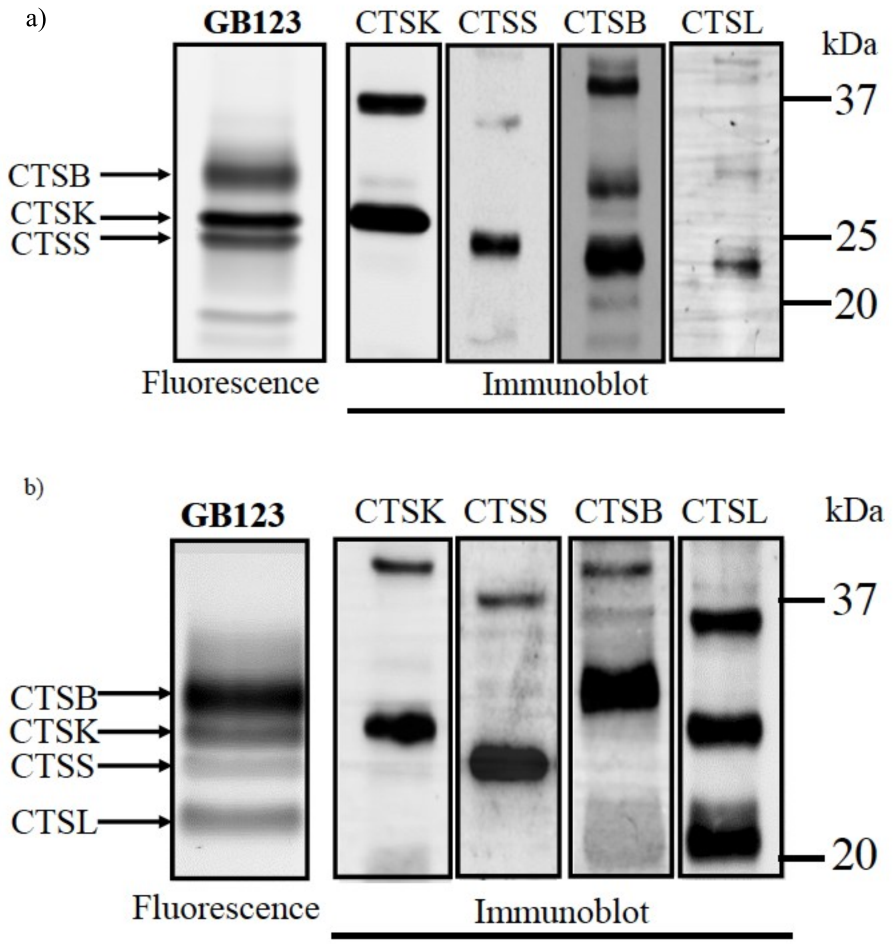

Figure S9. Detection of activity and expression of different cysteine-cathepsins in a) human osteoclasts, b) mice osteoclasts. Cathepsin activity was detected by labeling intact osteoclast with GB123, a pan-cathepsin activity-based probe. The samples then underwent immunoblotting using different cathepsin-specific antibodies, identifying the different cathepsins in the sample.

a)

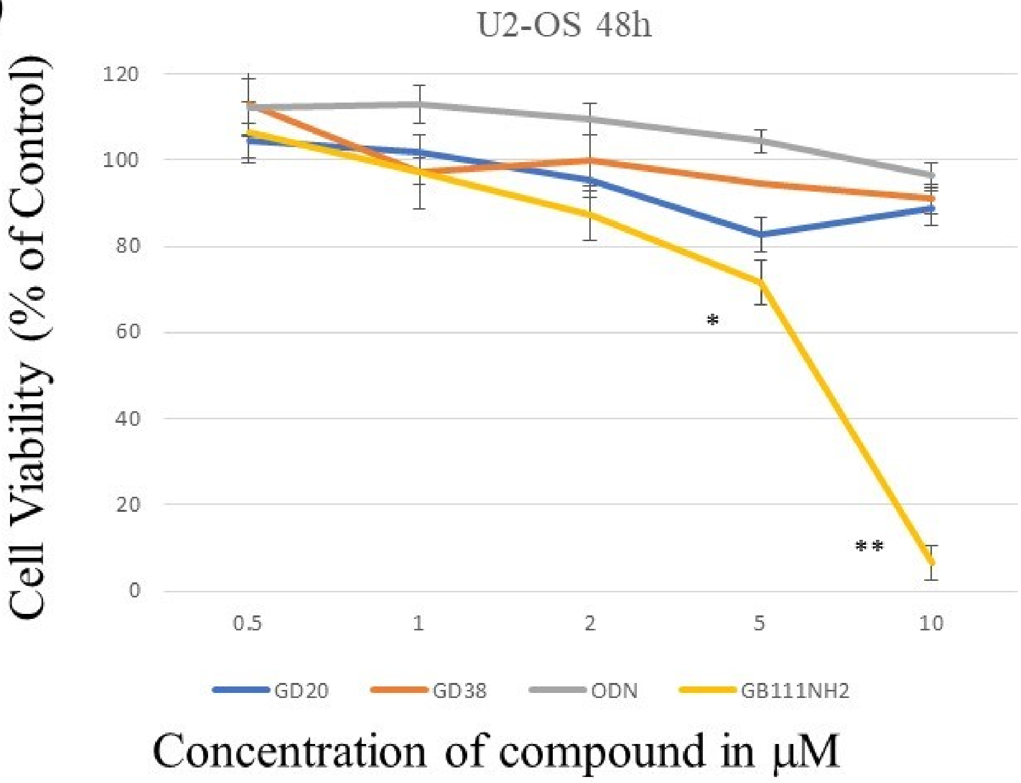
U2-OS 48h
Cell Viability (% of Control)
120
100
80
60
40
20
0
0.5
1
2
5
10
*
**
GD20
GD38
ODN
GB111NH2
Concentration of compound in μM

b)

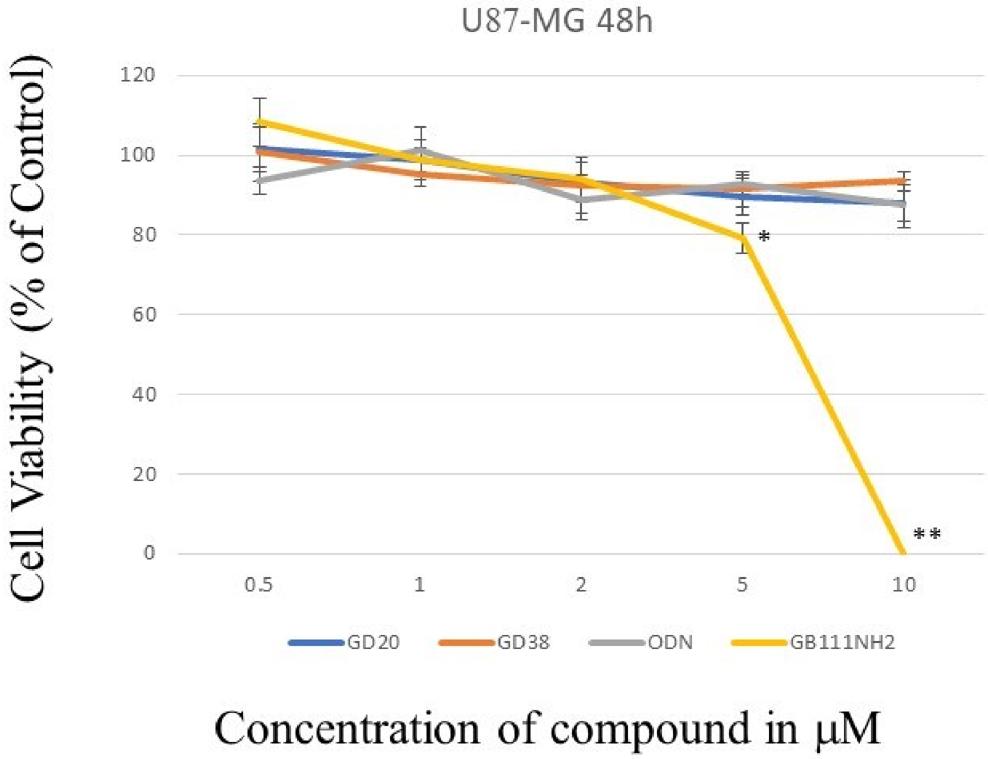
U87-MG 48h
Cell Viability (% of Control)
120
100
80
60
40
20
0
0.5
1
2
5
10
*
**
GD20
GD38
ODN
GB111NH2
Concentration of compound in μM

c)

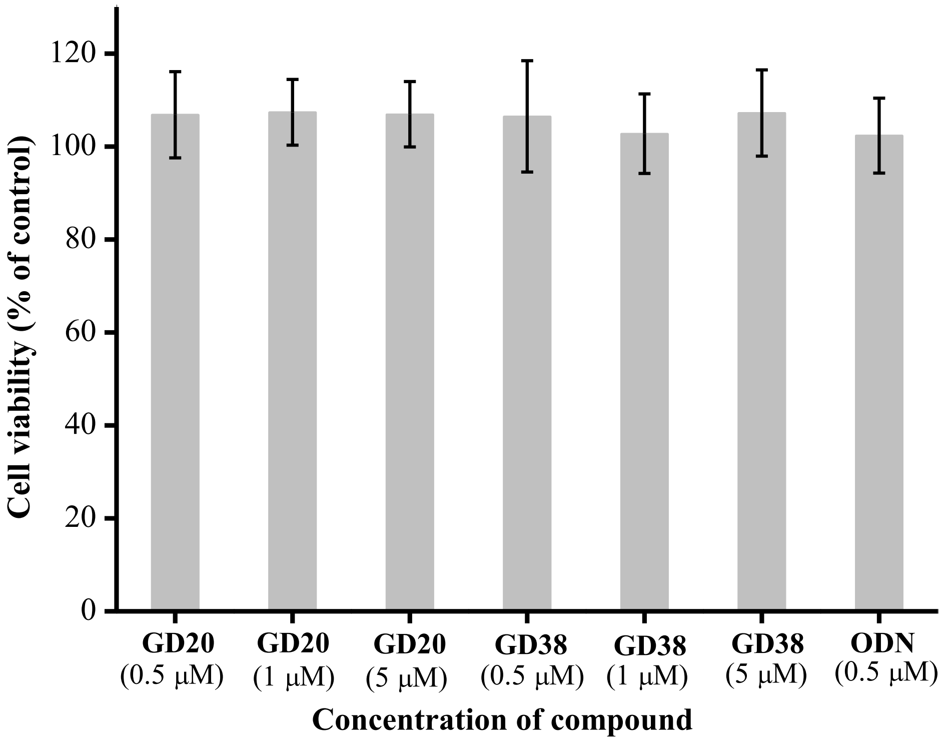

Figure S10. Cell viability assay. a) U2-OS and, b) U-87 MG were incubated with the different concentrations of indicated inhibitors. After a 48 h incubation cells were fixed, and viability was measured by methylene blue assay.The data were expressed as a percentage of control; data represent the mean of 2 experiments with triplicates for each treatment (± SD)**.** Statistical differences were evaluated by two-tailed Student's t-test from two independent experiments. Student's t-test was non-significant except for samples indicated with *, P <0.05 and **, P <0.01. (c) Human osteoclasts were incubated with the different concentrations of indicated inhibitors. After a 48 h incubation cells were fixed, and viability was measured by methylene blue assay. The results are displayed as mean ± SD of five biological replicates.The student's t-test was used and there were no significant differences between the treatments and the control.

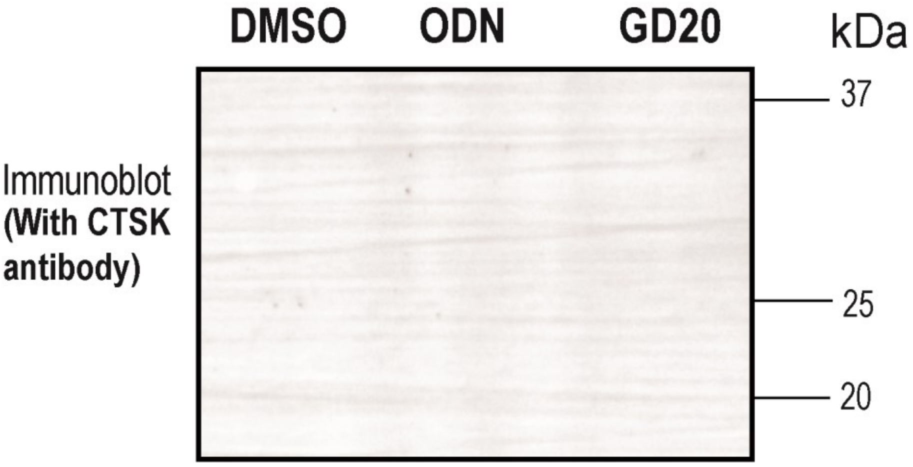

Figure S11. MCSF and IL-4 treated macrophage cells were treated for 2 hours with **GD20** (5 μM), ODN (5 μM) or DMSO in a culture medium, followed by 2 hours of labeling of residual enzyme activity by **GB123** (5 μM) for 2 hours. Samples were analyzed by SDS-PAGE and immuno blotted with a CTSK antibody.

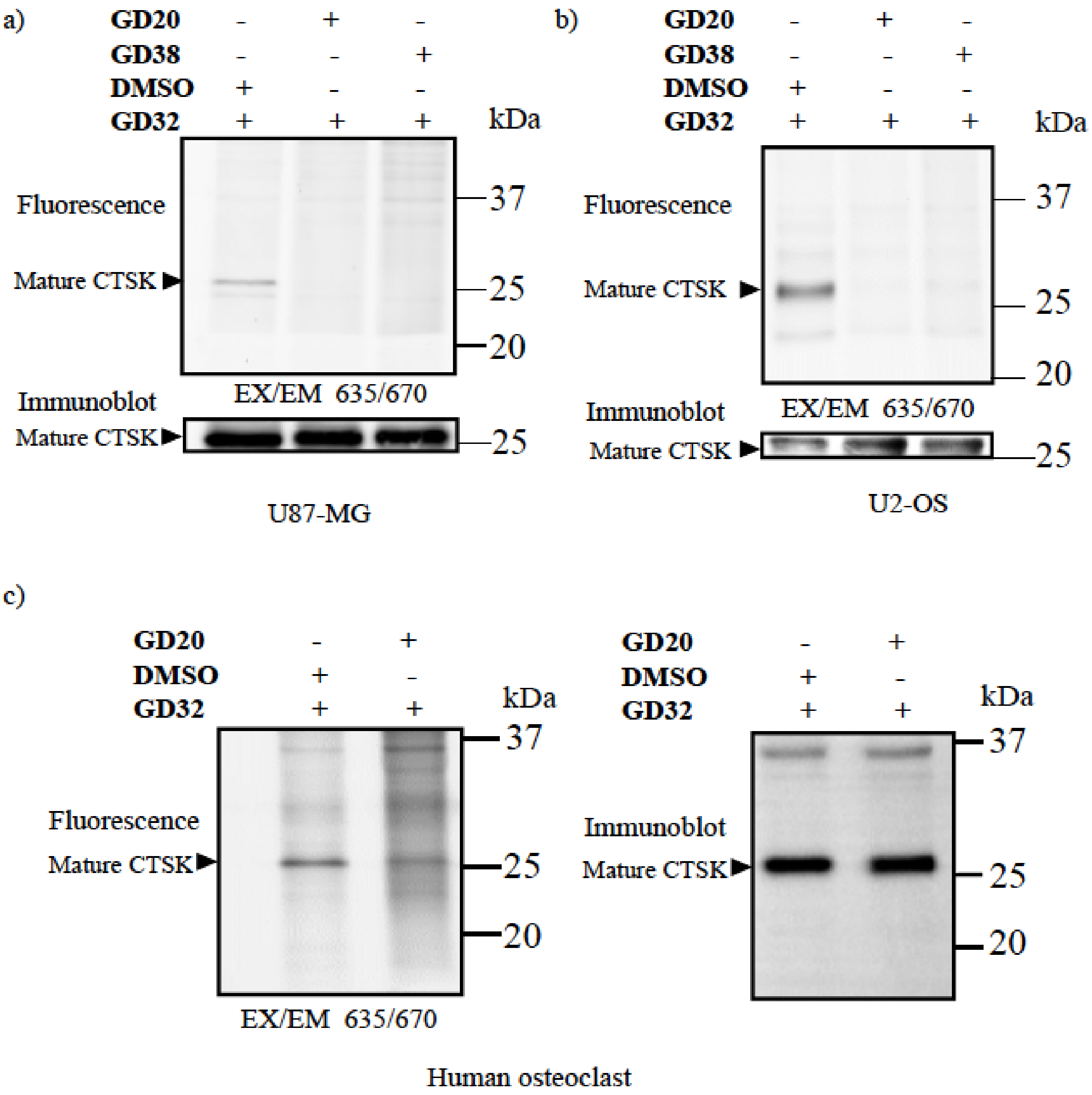

Figure S12. a) U87-MG, b) U2-OS cells were treated for 2 hours with **GD20** (5 µM), **GD38** (5 µM), or vehicle in the culture medium, followed by a) 2 hours, b) 24 hours of labeling of residual CTSK activity by **GD32** (5 µM). c) Human osteoclast cells grown on bones for 12 days) were treated for 2 hours with **GD20** (5 µM) or DMSO in a culture medium, followed by 6 hours of labeling of residual CTSK activity by **GD32** (5 µM). Samples were analyzed by SDS-PAGE and immune-blotted with CTSK antibody.

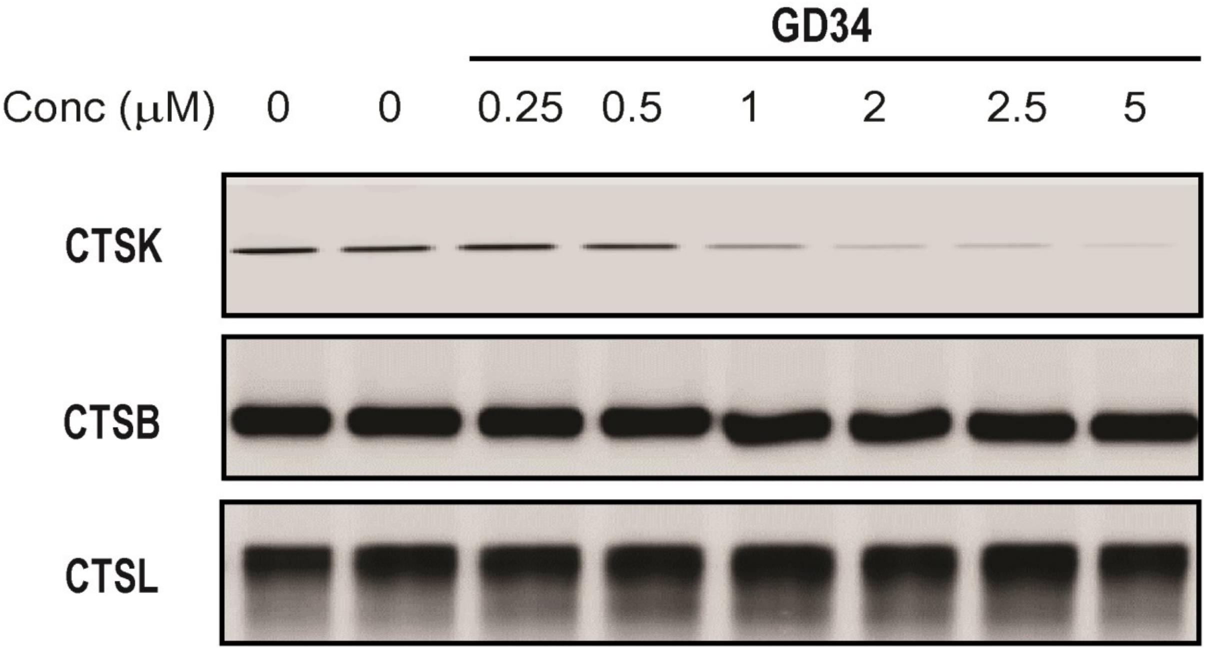

Figure S13. Inhibition of recombinant cathepsins by **GD34**. Recombinant cathepsins B, K, and L (0.5μM) have been treated with increasing concentrations of **GD34** followed by incubation with indicated concentrations of **GB123** (5μM). Samples were separated on SDS-PAGE. The gels were scanned for fluorescence by a Typhoon scanner at 635/670 nm.

Synthesis of compound (2)

Refer to Scheme-1

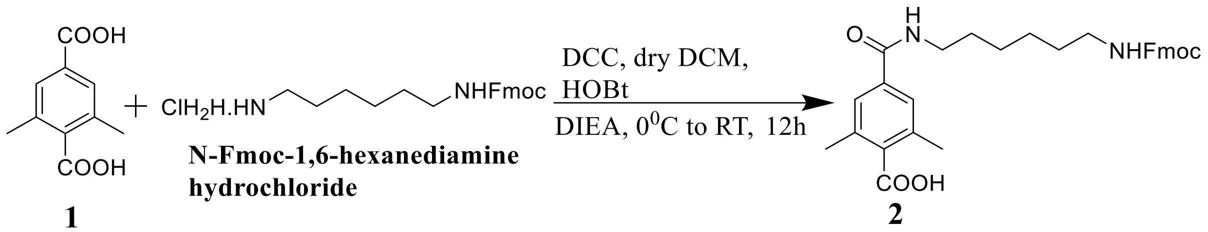

1 **2**

In a round-bottomed flask in a nitrogen atmosphere, commercially available compound **1** (1 equivalent, 500 mg, 2.57 mmol) and N-Fmoc-1,6-hexanediamine hydrochloride (4 equivalents) were added, followed by dry DCM, 3 equivalents of DCC (N, N′-Dicyclohexylcarbodiimide), 3 equivalents of HOBt (Hydroxybenzotriazole) and 5 equivalents of DIEA. The reaction mixture was allowed to stir at room temperature for 12 hours, and the progress of the reaction was monitored by TLC. After the reaction was finished, the reaction mixture was washed with 1N HCL solution, followed by separation of the organic layer and drying over sodium sulfate before concentrating in a rotavap. Finally, compound (**2**) was purified using column chromatography in a Hexane-Ethyl acetate system (800 mg, 1.55 mmol, 60.4% yield). The peak of the pure chemical was seen in the LCMS data. Calculated mass for [M + H] = 515.25, found in LCMS = 515.17.

Synthesis of compound (4)

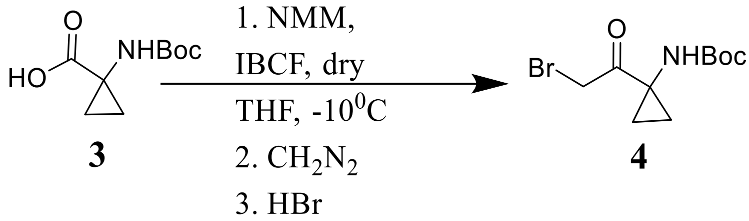

3 **4**

Compound **3** (1 equivalent, 400 mg, 1.98 mmol) was dissolved in dry THF at a temperature of -10° C in an inert atmosphere with argon. Isobutyl chloroformate (IBCF; 1.15 equivalent) and N-methyl morpholine (NMM; 1.25 equivalent) were added in turn. For two hours, the solution was left to swirl at -10° C. Then, at -10° C, excess ethereal diazomethane (14 eq.) that had been synthesized on-site was added to the anhydride. The resulting mixture was stirred at -10° C for one hour. For two hours, the temperature was increased to RT. The effervescence was halted by adding dropwise to the reaction mixture a solution of 1:9 water: 37% HBr at 0 °C. The organic layer was diluted in the solution by the addition of ethyl acetate. The liquid was then given a final water addition before being put into a funnel for organic layer separation. The organic layer was concentrated by a rotavapor and dried on sodium sulfate. Using an acetonitrile and water gradient, the resultant combination was purified using HPLC. The lyophilizer was used to freeze-dry the purified substance (331.75 mg, 1.19 mmol, 60% yield) before subjecting it to LCMS. Calculated mass for [M] = 277.03, found in LCMS = 277.58, calculated mass for [M – Boc + H] = 177.99, found in LCMS = 177.92.

Synthesis of compound (5)

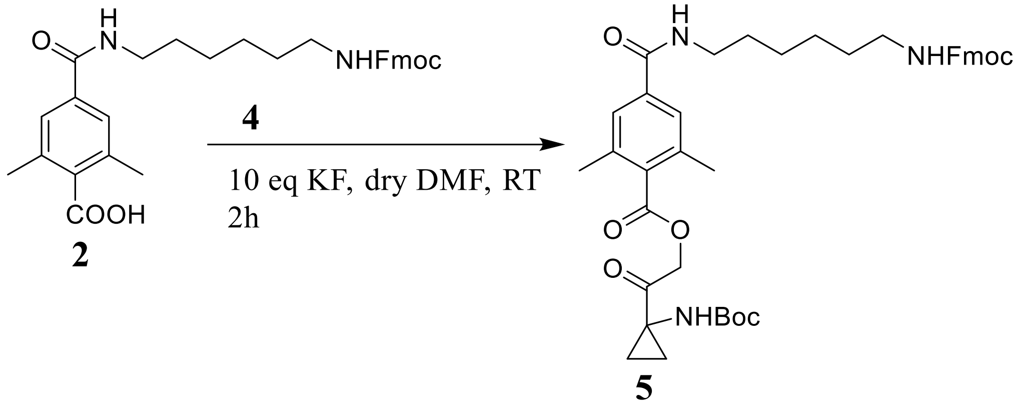

Compounds **2** (4 equivalents) and **4** (1 equivalent, 26.42 mg, 0.095 mmol) were dissolved in dry DMF at room temperature under an argon environment. KF (10 equivalents) was then added to the mixture and left to stir at room temperature for two hours. Following the workup

in a separating funnel using a water ethyl acetate combination, the light greenish-yellow tinted solution was put into ethyl acetate. Over sodium sulfate, the organic layer was recovered, dried, and evaporated using rotavapor. The resulting mixture was purified using HPLC using an acetonitrile and water gradient. The purified material was freeze-dried in a lyophilizer to receive (50 mg, .07 mmol, 74% yield). The peak of the pure chemical was detected by LCMS data. Calculated mass for [M + H] = 712.35, found in LCMS = 712.17.

Synthesis of compound (6)

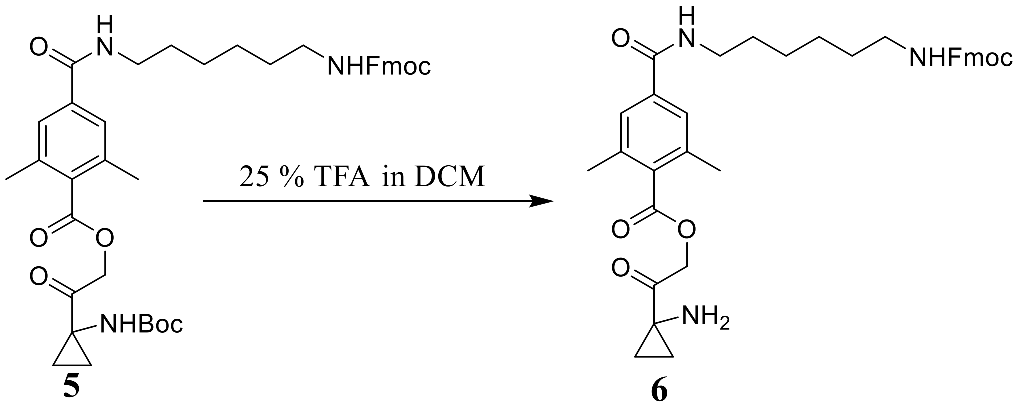

For 30 minutes at room temperature, compound **5** (50 mg, 0.07 mmol) was allowed to stir in a 25 percent trifluoroacetic solution in DCM. DCM was used to dilute the resultant solution before it was removed by a rotavap. Compound **6** was analyzed by LCMS, and was further used without purification (36 mg, 0.058 mmol, 83.71 % yield). The peak of the pure chemical was detected by LCMS. Calculated mass for [M + H] = 612.31, found in LCMS = 612.42.

Synthesis of compound (8)

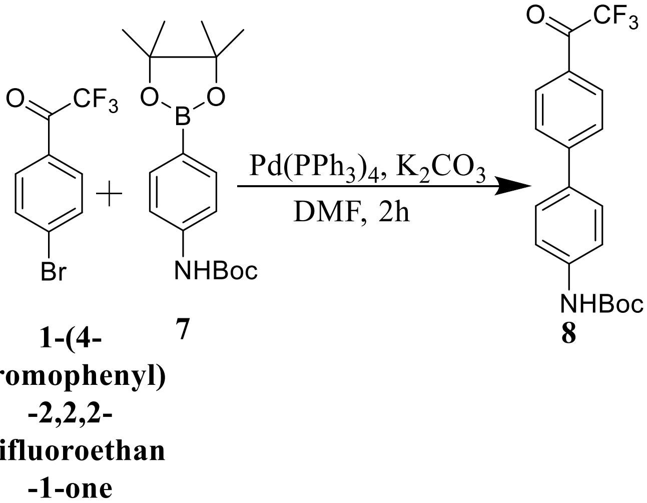

1-(4-bromophenyl)-2,2,2-trifluoroethan-1-one **7** **8**

Commercially available compound **1-(4-bromophenyl)-2,2,2-trifluoroethan-1-one** (1.25 equivalent) and compound **7** (1 equivalent, 400 mg, 1.25 mmol) were taken in a round-bottomed flask in a nitrogen atmosphere followed by the addition of dry DMF, 5 equivalents of palladium tetrakis and 2 M aqueous solution of potassium carbonate. The reaction mixture was allowed to reflux at 82° C for 2 hours, and the progress of the reaction was monitored by TLC. Once the reaction was completed, the solvent was evaporated using a rotavap. Then, the reaction mixture was dissolved in ethyl acetate before washing with 1N HCL solution, followed by separation of the organic layer and dried over sodium sulfate before concentrating by a rotavap. Finally, the compound was purified through column chromatography using Hexane – Ethyl acetate system (350 mg, 0.953 mmol, 76.25% yield). The compound was analyzed by NMR.

Synthesis of compound (9 and 10)

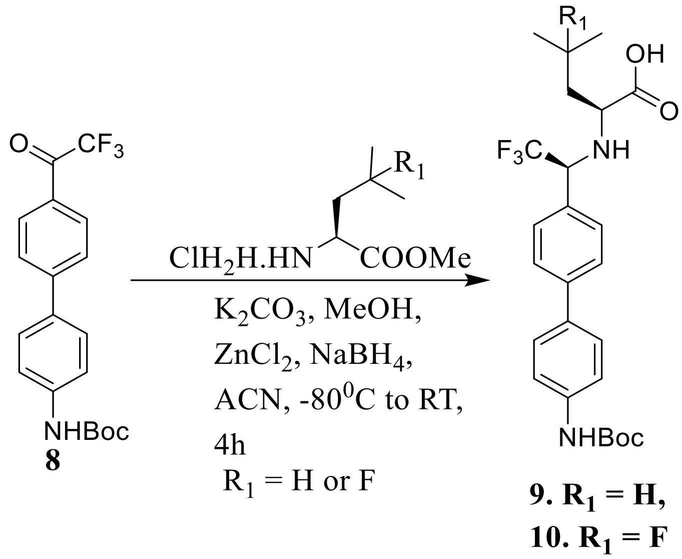

9. R_1 = H,
10. R_1 = F

With certain modifications, compounds **9** and **10** were synthesized using the method described in the literature. Anhydrous zinc chloride (2 equivalent) in dry DME was added to sodium borohydride (4 equivalent) at 0 °C and stirred at room temperature for 18 hours in a nitrogen environment. In the meanwhile, compound **8** (1 equivalent, 150 mg, 0.041 mmol)) was heated to 55°C for 12 hours in a nitrogen environment with methyl ester salt of L-leucine or L-fluoro leucine (1.25 equivalent), potassium carbonate (3 equivalent), and dry methanol. The methanol was then removed using a rotavap after the reaction mixture was allowed to cool to room temperature. While keeping the temperature at -44° C, dry acetonitrile was gradually added to the mixture and transferred to the flask with a round bottom holding the mixture of zinc chloride and sodium borohydride. The resultant reaction mixture was left to stir for 4 hours at this Temperature. To quench the reducing agent in the reaction mixture, acetone was added. The reaction mixture was then given an hour to reach room temperature. After adding 1(N) HCl, the solvent was evaporated with the aid of a rotavap. The crude was mixed with tertiary butyl methyl ether; then, water was used to extract the product. The ether layer was collected and dried. The product was purified by reverse-phase HPLC with a water/acetonitrile gradient. The

products were freeze-dried before further use (For **9**, 100 mg, 0.2 mmol, 50.68 % yield), (For **10**, 48 mg, 0.08 mmol, 22% yield). The purity and identity of the compounds were analyzed by LCMS and NMR. Calculated mass for **9** [M + H] = 481.23, found in LCMS = 481.22. Calculated mass for **10** [M - H] = 497.21, found in LCMS = 497.17.

Synthesis of compound (11 and 12)

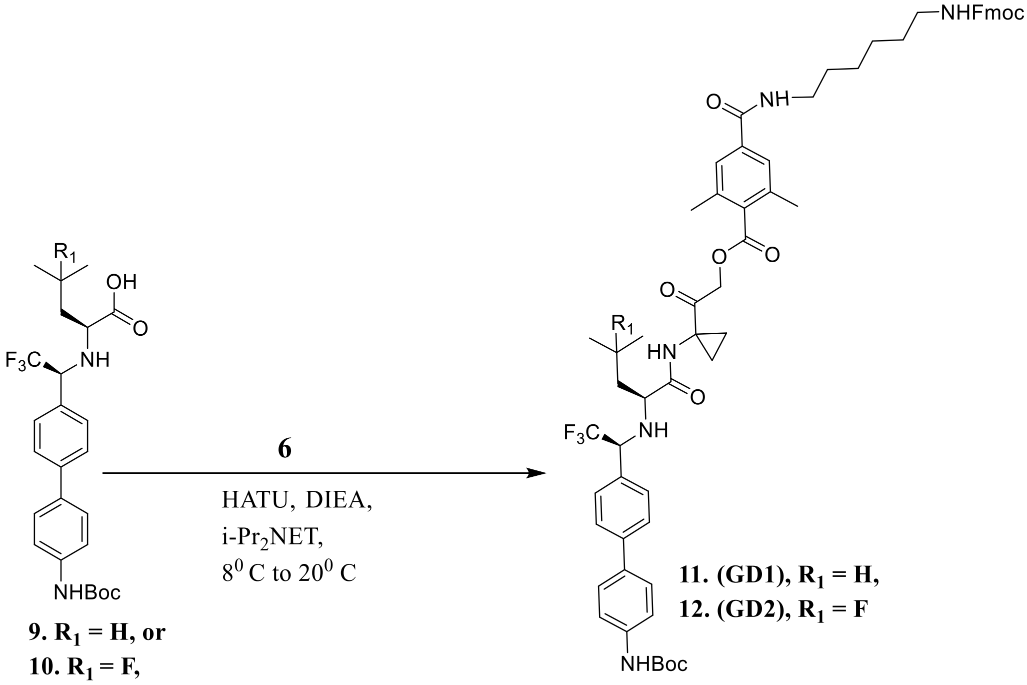

9. R_1 = H, or
10. R_1 = F,

11. (GD1), R_1 = H,
12. (GD2), R_1 = F

Compound **9 (**1 equivalent, 20 mg, 0.041 mmol**)** or **10 (**1 equivalent, 20 mg, 0.04 mmol**)** were put in ice-cooled dimethyl acetamide in a round-bottom flask. Compound **6** (3 equivalents) and HATU (4 equivalents) were added to the solution. After 10 mins of stirring at 0^0 C, DIEA (6 equivalents) was added to the solution. The reaction mixture was allowed to stir for another 2.5 hours, keeping the temperature around 8^0 C in a nitrogen atmosphere. Water was added to the reaction mixture and allowed to stir for another 1.5 hours at 20^0 C. The precipitate formation was observed at the bottom of the round-bottomed flask. The light-yellow colored precipitate was filtered and washed with a 1:1.2 DMF/water solution and water. Finally, the washed precipitate was then subjected to preparative reverse phase HPLC purification using a

water/acetonitrile gradient. The purified compounds were analyzed by LCMS **(11**, and **12)** and were freeze-dried before further used **(**For **11,** 5 mg, 0.0046 mmol, 11.3% yield**), (**For **12,** 4.8 mg, 0.0043 mmol, 10.95% yield**).** Calculated mass for **11** [M] = 1073.51, found in LCMS = 1074.08, Calculated mass for **12** [M] = 1091.5, found in LCMS = 1092.25.

Synthesis of compound (13 and 14)

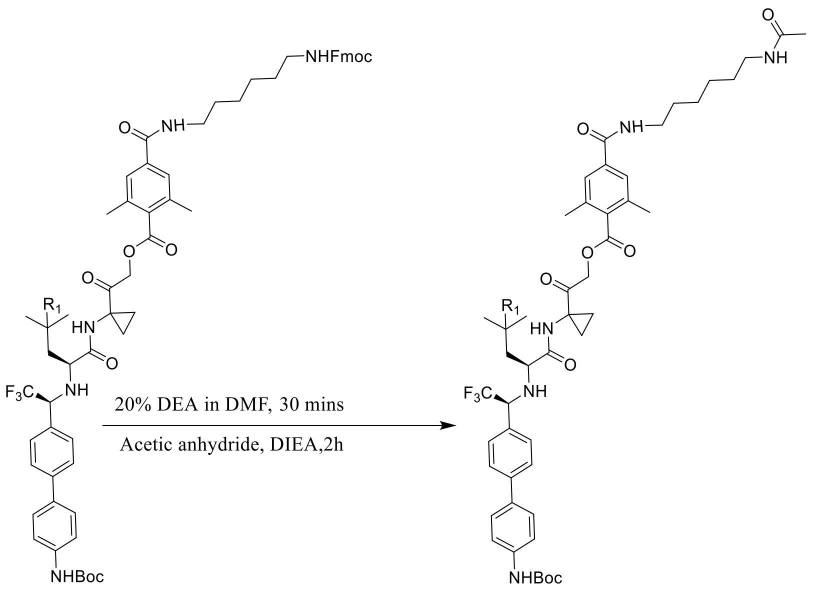

11. (GD1), R_1 = H,
12. (GD2), R_1 = F

13. (GD3), R_1 = H,
14. (GD4), R_1 = F

Compound **11** (2 mg, 0.001 mmol) or **12** (2 mg, 0.001 mmol) was taken in a round-bottom flask in the presence of 20% DEA/DMF solution (2 ml). The reaction mixture was stirred for 30 mins at RT. The progress of the reaction was monitored by LCMS. After the consumption of starting materials, DMF was removed. The resulting products were dissolved in dry DCM, 2 equivalents of acetic anhydride with 3 equivalents of DIEA were added. The reaction mixture was stirred in RT for 2 hours in an argon atmosphere. Next, after the evaporation of DCM, a water acetonitrile gradient was used for crude purification by reverse-phase HPLC. The chemicals were freeze-dried before further use **(**for **13,** 1.2 mg, 0.0013 mmol, 72.2% yield**)**, **(**for **14,** 0.8 mg, 0.0008 mmol, 47.9% yield**)**. The purity of the compounds was determined by

LCMS. Calculated mass for **13** [M] = 893.46, found in LCMS = 894.05, Calculated mass for **14** [M] = 911.45, found in LCMS = 912.25.

Synthesis of compound (16).

Refer to Scheme -2

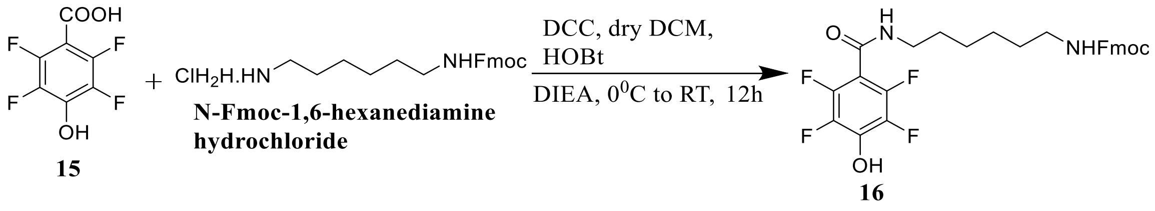

Commercially available compound **15** (1 equivalent, 500 mg, 2.38 mmol) and *N*-Fmoc-1,6-hexanediamine hydrochloride (4 equivalents) were put in a round-bottomed flask under a nitrogen atmosphere in dry DCM. DCC (N, N′-Dicyclohexylcarbodiimide), 3 equivalents, 3 equivalents of HOBt (Hydroxybenzotriazole), and 5 equivalents of DIEA were added. The reaction mixture was allowed to stir at room temperature for 12 hours, and the progress of the reaction was monitored by TLC. Once the reaction was complete, the reaction mixture was washed with 1N HCL solution, followed by separation of the organic layer, and dried over sodium sulfate before concentrating by a rotavap. Finally, compound **(16)** was purified through column chromatography using Hexane – Ethyl acetate system (800 mg, 1.5 mmol, 63.5% yield). Calculated mass for **16** [M + H] = 531.19, found in LCMS = 531.17.

Synthesis of compound (17).

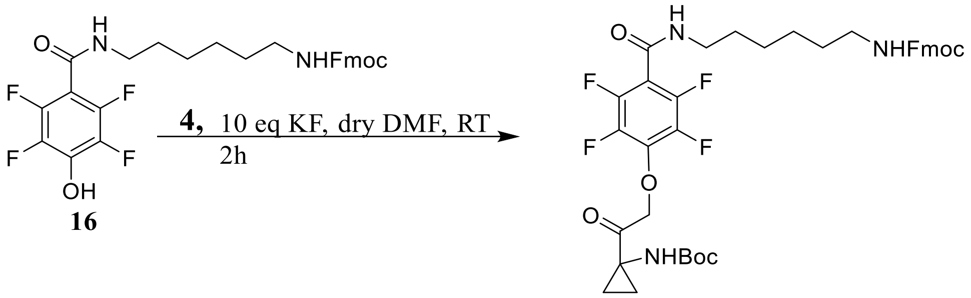

Compounds **16** (4 equivalent) and **4** (1 equivalent, 40 mg, 0.143 mmol) were dissolved in dry DMF at room temperature in an argon environment. KF (10 equivalents) was then added to the mixture and left to stir at room temperature for two hours. Following the workup in a separating funnel using a water ethyl acetate combination, the light greenish-yellow tinted solution was put into ethyl acetate. Over sodium sulfate, the organic layer was recovered, dried, and evaporated using a rotavap. The resulting mixture was purified using HPLC using an acetonitrile and water gradient. The purified material was freeze-dried in the lyophilizer before being subjected to LCMS (60 mg, 0.082 mmol, 57.33% yield). The LCM data showed the peak of the pure chemical. Calculated mass for **17** [M] = 727.29, found in LCMS = 728.

Synthesis of compound (18).

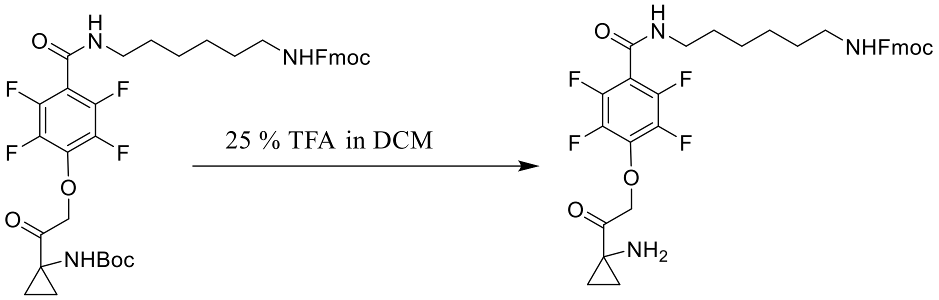

For 30 minutes at room temperature, compound **17** (55 mg, 0.0755 mmol) was allowed to stir in a 25 percent trifluoroacetic solution of DCM. DCM was used to dilute the resultant solution before it was removed by a rotavap. Compound **17** was analyzed by LCMS and was further

used without purification (38 mg, 0.06 mmol, 80% yield). Calculated mass for **18** [M] = 627.24, found in LCMS = 627.64.

Synthesis of compound (19).

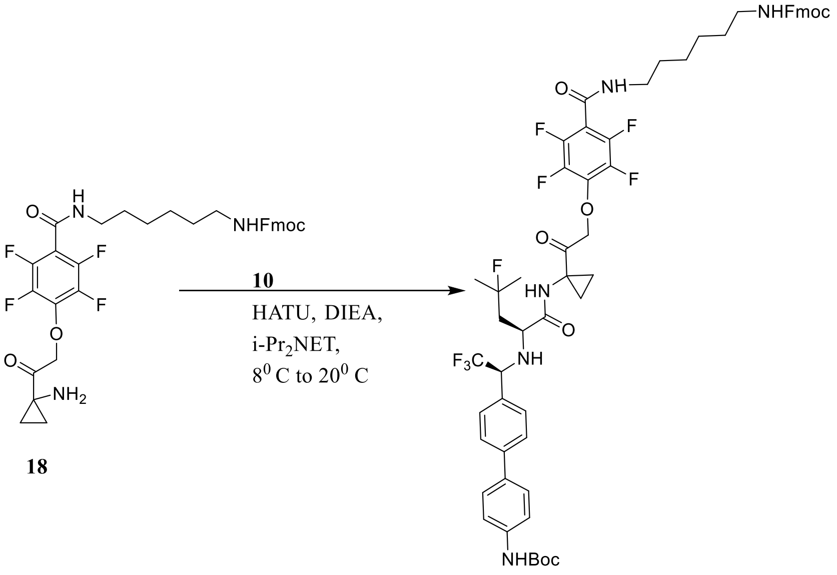

18

19. (GD5)

Compound **10** (1 equivalent, 20 mg, 0.04 mmol) was put in ice-cooled dimethyl acetamide in a round-bottomed flask. Compound **18** (3 equivalents) and HATU (4 equivalents) were added to the solution. After 10 mins of stirring at 0^0 C, DIEA (6 equivalents) was added to the solution. The reaction mixture was allowed to stir for another 2.5 hours keeping the temperature around 8^0 C. A required amount of water was added to the reaction mixture and allowed to stir for another 1.5 hours at 20^0 C. The precipitate formation was observed at the bottom of the round-bottom flask. The light-yellow colored precipitate was filtered and washed with a 1:1.2 DMF/water solution and water. Finally, the washed precipitate was subjected to preparative reverse phase HPLC purification using a water acetonitrile gradient. The purified compound **(GD5)** was finally freeze-dried before it was used for experimental purposes (5 mg, 0.004 mmol, 11.2% yield). The LCMS data showed the peak of the pure chemicals. Calculated mass for **GD5** [M] = 1108.14, found in LCMS = 1108.25.

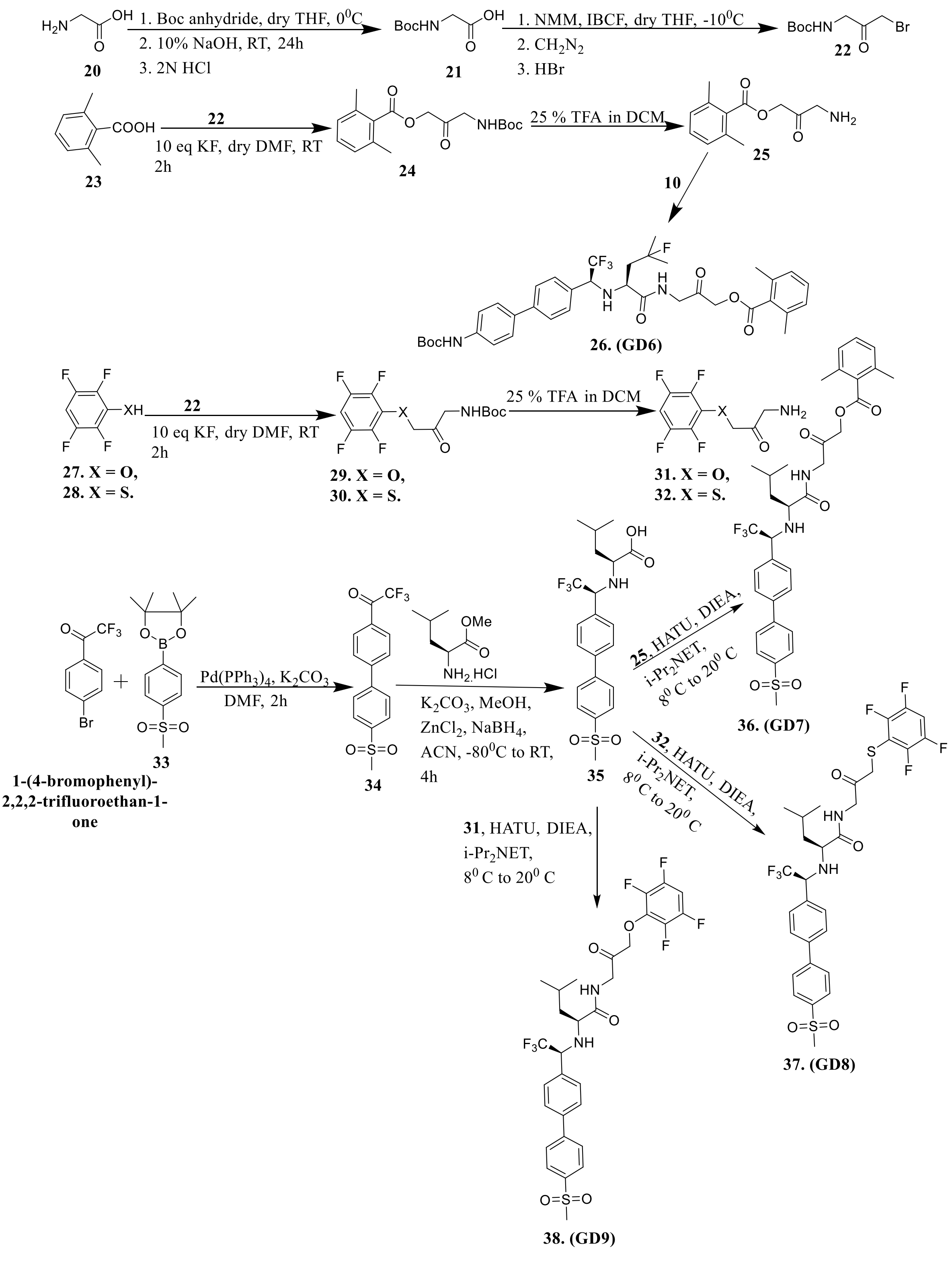

Scheme S3. Synthetic scheme of compound **GD6 - GD9.**

Synthesis of compound (21).

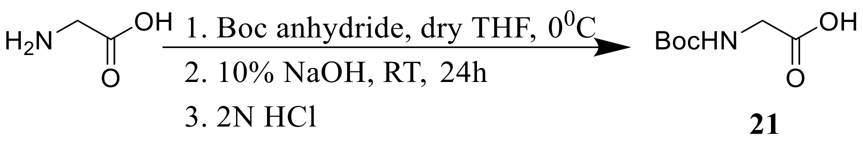

Glycine (1 equivalent, 1 gram, 13.3 mmol) was dissolved in a 10% sodium hydroxide solution in tetrahydrofuran (THF) at 0 degrees Celsius. Then, 1.5 equivalents of BOC-anhydride were added to the solution. The solution was stirred for 24 hours at room temperature. The solvent was dried by a rotavap and then neutralized using a 2% hydrochloric acid solution. Subsequently, the solution was obtained by employing ethyl acetate and then subjected to evaporation using a rotavapor after being treated with sodium sulfate. A white compound (**21**) was obtained (1.8 gr, 10.2 mmol, 77.2% yield). This product was employed in the next stage without undergoing any additional purification.

Synthesis of compound (22).

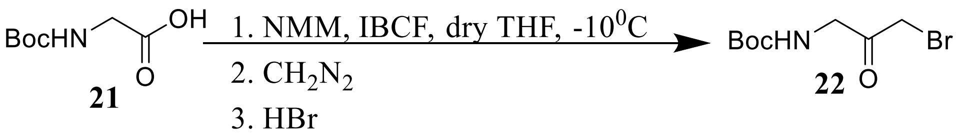

Compound **21** (1 equivalent, 1.6 gr, 9.13 mmol) was dissolved in dry THF at a temperature of -10 °C in an inert atmosphere under argon. Isobutyl chloroformate (IBCF; 1.15 equivalents) and N-methyl morpholine (NMM; 1.25 equivalents) were added in turn. For two hours, the solution was left to swirl at -10° C. Then, at -10° C, excess ethereal diazomethane (14 equivalents) that had been synthesized on-site was added to the anhydride. The resulting mixture was stirred at -10° C for one hour. For two hours, the temperature was allowed to reach RT. The effervescence was halted by adding a solution of 1:9 (water: 37% HBr) dropwise at 0° C. The organic layer was diluted in the solution by the addition of ethyl acetate. The liquid was then given a final water addition before being put into a funnel for organic layer separation. The organic layer was concentrated by a rotavap and dried on sodium sulfate. The reaction

mixture was purified by HPLC using an acetonitrile and water gradient receiving (1.8 gr, 7.13 mmol, 78.2% yield). The product was lyophilized before LCMS analysis. The peak of the pure chemical was detected by LCMS. Calculated mass for **22** [M] = 251.0157, found in LCMS = 251.67.

Synthesis of compound (24).

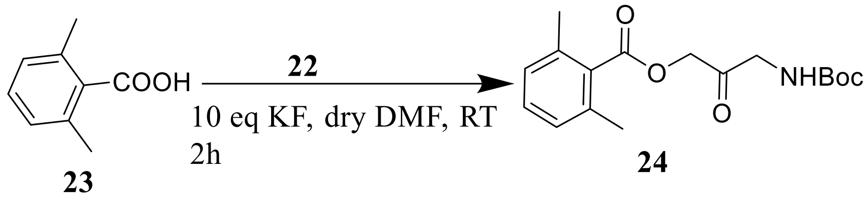

23 **24**

Compounds **23** (4 equivalents) and **22** (1 equivalent, 100 mg, 0.39 mmol) were dissolved in dry DMF at room temperature in an dry environment. KF (10 equivalents) was then added to the mixture and left to stir at room temperature for two hours. Following the workup in a separating funnel using a water ethyl acetate combination, the light greenish-yellow tinted solution was put into ethyl acetate. Over sodium sulfate, the organic layer was recovered, dried, and evaporated using a rotavap. The resulting mixture was purified using HPLC using an acetonitrile and water gradient. The purified material was freeze-dried in the lyophilizer before being subjected to LCMS (81 mg, 0.252 mmol, 63.5% yield). The LCMS data showed the peak of the pure chemical. Calculated mass for **24** [M-Boc] = 221.11, found in LCMS = 222.

Synthesis of compound (25).

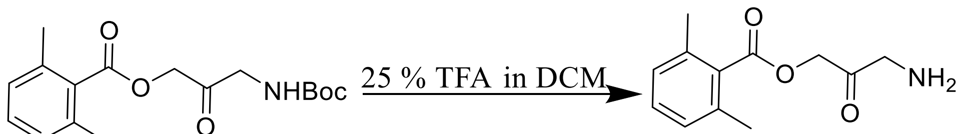

24 **25**

Compound **24** (80 mg, 0.248 mmol) was allowed to stir in a 25% trifluoroacetic/DCM solution for 30 minutes at room temperature. DCM was used to dilute the resultant solution before it

was evaporated by a rotavap. In LCMS, compound **25** was verified, and it was employed directly in the next step without purification (48 mg, 0.216 mmol, 87% yield). Calculated mass for **25** [M + H] = 222.11, found in LCMS = 222.83.

Synthesis of compound (26).

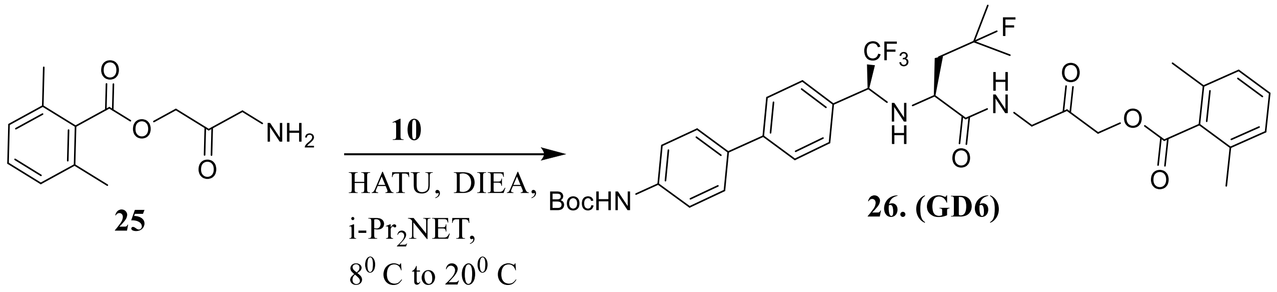

Compound **10** (1 equivalent, 7 mg, .014 mmol) was put in ice-cooled dimethyl acetamide in a round-bottomed flask. Compound **25** (3 equivalents) and HATU (4 equivalents) were added tothe solution. After 10 mins of stirring at 0^0 C, DIEA (6 equivalents) was added to the solution. The reaction mixture was allowed to stir for another 2.5 hours, keeping the temperature around 8^0 C. Water was added to the reaction mixture and was stirred for another 1.5 hours at 20^0 C. The precipitate formation was observed at the bottom of the round-bottomed flask. The light-yellow colored precipitate was filtered and washed with a 1:1.2 DMF/water solution and water. Finally, the washed precipitate was then subjected to preparative reverse phase HPLC purification using a water acetonitrile gradient. The purified compound **26 (GD6)** was finally freeze-dried before it was used for experimental purposes receiving (1.2 mg, 0.0017 mmol, 12.1% yield). The LCMS data showed the peak of the pure compound. Calculated mass for **25** [M+H] = 702.32, found in LCMS = 702.42.

Synthesis of compounds (29) and (30).

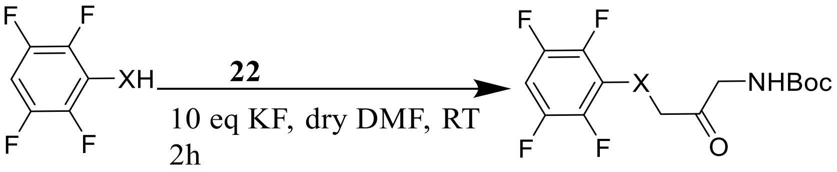

27. X = O,
28. X = S.

29. X = O,
30. X = S.

Compounds **27** (4 equivalents) or **28** (4 equivalents) and **22** (1 equivalent, 100 mg, 0.39 mmol) were dissolved in dry DMF at room temperature under argon. KF (10 equivalents) was then added to the mixture and left to stir at room temperature for two hours. Following the workup in a separating funnel using water and ethyl acetate, the light orange solution was put into ethyl acetate. The organic layer was recovered, dried, over sodium sulphate and evaporated using rotavap. The resulting mixture was purified using HPLC with an acetonitrile/water gradient. The purified material was freeze-dried in the lyophilizer before being subjected to LCMS (for **30,** 88 mg, 0.261 mmol, 66.9% yield), (for **29**, 74 mg, 0.0216 mmol, 55.53% yield). The LCMS data showed the peak of the pure chemicals. Calculated mass for **29** [M] = 337.09, found in LCMS = 337.08. Calculated mass for **30** [M-Boc+H] = 253.02, found in LCMS = 253.92.

Synthesis of compound (34)

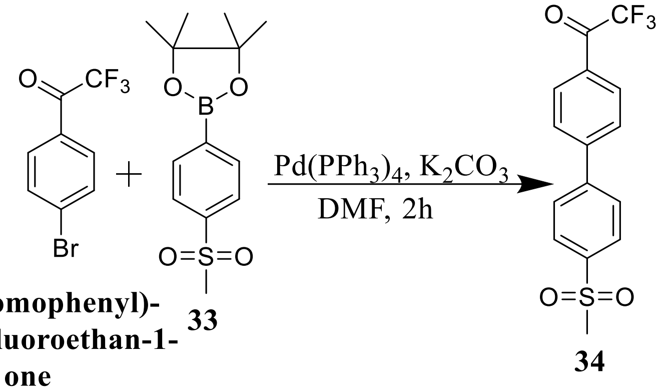

1-(4-bromophenyl)-2,2,2-trifluoroethan-1-one

33

34

Commercially available compound **1-(4-bromophenyl)-2,2,2-trifluoroethan-1-one** (1.25 equivalent) and compound **33** (1 equivalent, 600 mg, 2.12 mmol) were taken in a round-bottomed flask in a nitrogen atmosphere followed by the addition of dry DMF, 5 equivalent of

palladium tetrakis and 2 M aqueous solution of potassium carbonate. The reaction mixture was allowed to reflux at 82° C for 2 hours and the progress of the reaction was monitored by TLC. Once the reaction was completed, the solvent was removed using a rotavap. The reaction mixture was dissolved in ethyl acetate before washing with 1N HCL solution, followed by separation of the organic layer and drying over sodium sulfate before concentrating using a rotavap. Finally, the compound was purified through column chromatography using a hexane–ethyl acetate system (510 mg, 1.55 mmol, 73% yield). The compound **34** was analyzed by NMR.

Synthesis of compound (35)

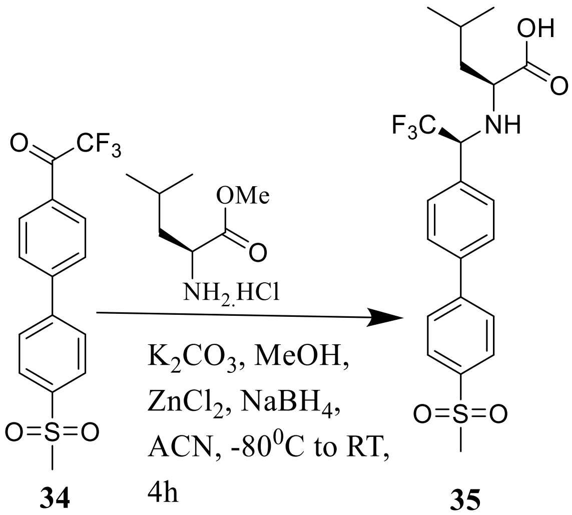

34 **35**

Anhydrous zinc chloride (2 equivalent) in dry DME was added to sodium borohydride (4 equivalent) at 0 °C and stirred at room temperature for 18 hours in a nitrogen environment. In the meanwhile, compound **34** (1 equivalent, 500 mg, 1.52 mmol) was heated to 55°C for 12 hours in a nitrogen environment with methyl ester salt of L-leucine (1.25 equivalent), potassium carbonate (3 equivalent), and dry methanol. The methanol was then removed using a rotavap, after the reaction mixture was allowed to reach room temperature. While keeping

the temperature at -44°C, dry acetonitrile was gradually added to the mixture that was then transferred to a flask with a mixture of zinc chloride and sodium borohydride. At this temperature, the reaction mixture was left to stir for 4 hours. To quench the reducing agent present in the reaction mixture, acetone was added. The reaction mixture was then given an hour to reach room temperature. After adding 1(N) HCl, the solvent was evaporated with the aid of a rotavap. The crude compound was mixed with tertiary butyl methyl ether, then water was added for workup. The ether layer was collected and dried by a rotavap. Reverse-phase HPLC purification with a water/acetonitrile gradient was used to purify the product. The chemical was freeze-dried giving (280 mg, 0.631 mmol, 41% yield). The compound was analysed by LCMS and NMR. Calculated mass for **35** [M] = 443.13, found in LCMS = 444.08.

Synthesis of compound (36), (37), and (38).

See the synthetic route in **Scheme S3** above.

Compound **35** (1 equivalent, 20 mg, 0.045 mmol) was put in ice-cooled dimethyl acetamide in a round bottom flask. Compounds **25** or **31** or **32** (3 equivalent) and HATU (4 equivalent) were mixed in the solution. After 10 mins of stirring at 0^0 C, DIEA (6 equivalent) was added to the solution. The reaction mixture was allowed to stir for another 2.5 hours, keeping the temperature around 8^0 C. Required amount of water was added to the reaction mixture and allowed to stir for another 1.5 hours at 20^0 C. The precipitate formation was observed at the bottom of the round-bottomed flask. The light-yellow colored precipitate was filtered and washed with a 1:1.2 DMF/water solution and water. Finally, the washed precipitates were subjected to preparative reverse phase HPLC purification using a water acetonitrile gradient. The purified compounds **(36, 37, and 38)** were freeze-dried before they were used for experimental purposes and analyzed by LCMS (for **36**, 4 mg, 0.006 mmol, 13.7% yield), (for **37**, 6.1 mg, 0.0089 mmol, 20% yield), (for **38**, 1.2 mg, 0.0018 mmol, 4% yield). Calculated mass for **36** [M] = 646.23, found in LCMS = 646.92. Calculated mass for **37** [M] = 678.68, found in LCMS = 679. Calculated mass for **38** [M] = 662.17, found in LCMS = 662.75.

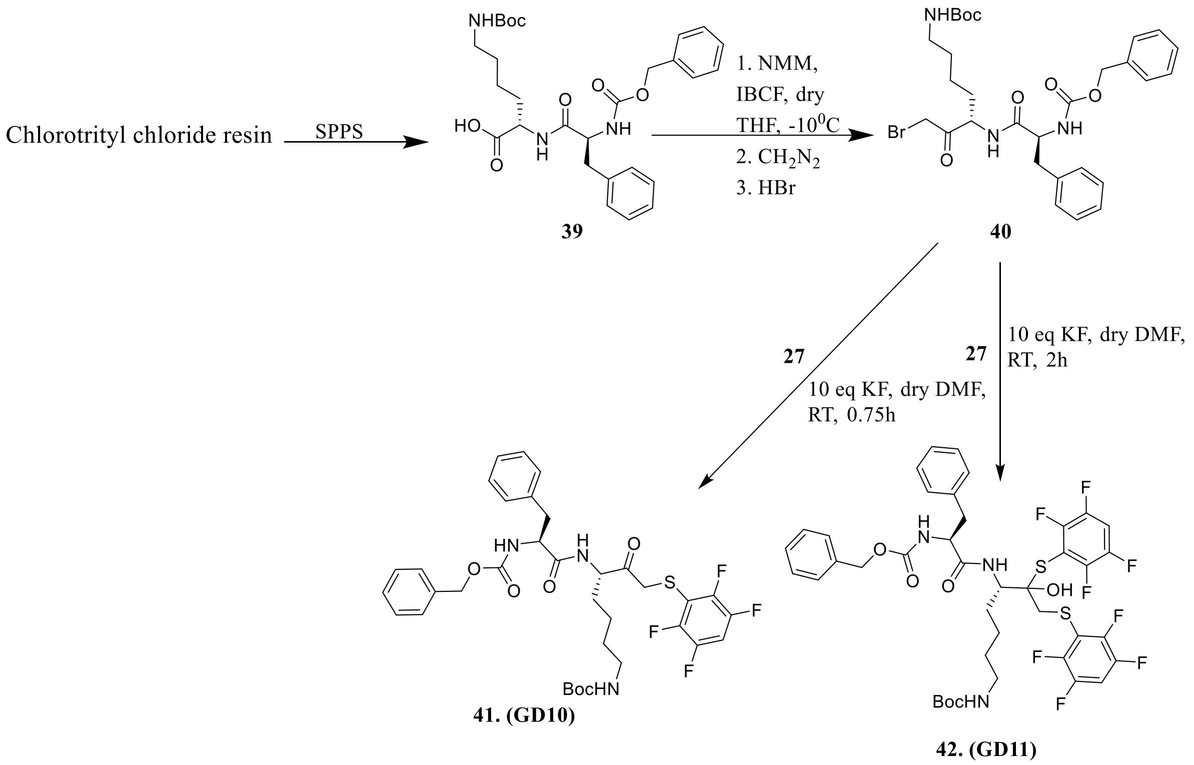

Scheme S4. Synthetic scheme of compound **GD10 - GD11.**

Solid phase peptide synthesis of peptide (39).

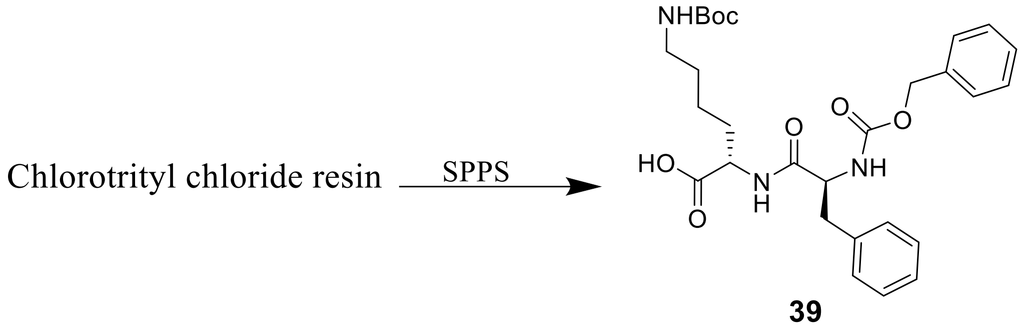

39

Chlorotityl chloride resin was loaded with Fmoc-Lysine (Boc)-OH (1.5 equivalents, 2 gr, 4.26 mmol) and diisopropylethylamine (DIEA) for 2 hours with vigorous shaking using dry DCM as the solvent. Methanol (1 ml/gr) was added to the reaction mixture and agitated for another 30 minutes before being rinsed multiple times with DMF and DCM. To remove the Fmoc protective group of lysine, 20% piperidine/DMF (v/v) was added to the resin and stired for 40 minutes. After that, the resin was washed multiple times with DMF and DCM before the

peptide was elongated ba adding a solution of N -benzyloxycarbonyl-phenyalanine (3 equivalents), DIPEA (3 equivalents), hydroxybenzotriazole (HOBT; 3 equivalents), and HATU (3 equivalents) in DMF for 6 hours. The resin was washed numerous times with DMF and DCM. The final product was cleaved from the resin with a 5% TFA in DCM solution. Following rotavapor evaporation, the final product was purified by HPLC using an acetonitrile-water gradient (74% yield relative to resin loading). The peptide's mass was determined using LCMS. Calculated mass for **39** [M] = 678.68, found in LCMS = 679.

Synthesis of compound (40).

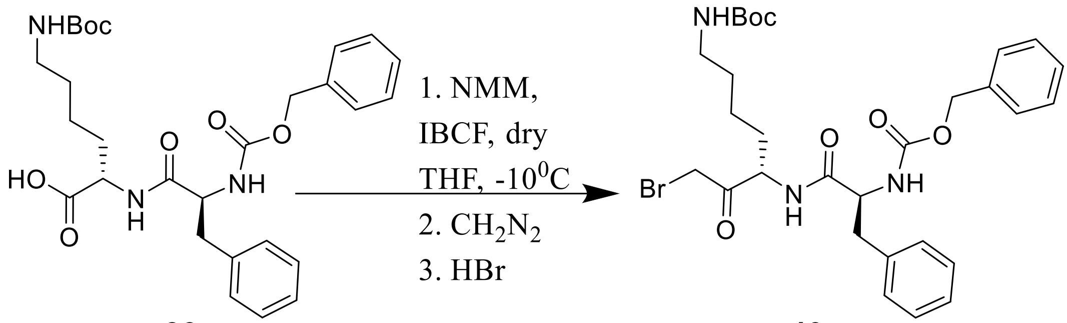

39 **40**

Compound **39** (1 equivalent, 200 mg, 0.294 mmol) was dissolved in dry THF at -10 °C in an inert atmosphere under argon. Isobutyl chloroformate (IBCF; 1.15 equivalents) and N-methyl morpholine (NMM; 1.25 equivalents) were added in turn. For two hours, the solution was left to swirl at -10° C. Then, at -10° C, excess ethereal diazomethane (14 equivalents) that was synthesized on-site was added to the anhydride. The resulting mixture was stirred at -10° C for one hour. The temperature was increased to RT over two hours. A solution of 1:1 water: 37% HBr was added dropwise to the reaction mixture at 0° C, stopping the bubbling. The organic layer was diluted in the solution by the addition of ethyl acetate. Water was added, and the organic layer was collected using a separation funnel. The organic layer was concentrated using a rotavap and dried on sodium sulfate. The reaction mixture was purified using an HPLC using an acetonitrile/water gradient. The purified product was freeze-dried with a lyophilizer before

subjecting it to LCMS (118 mg, 0.195 mmol, 66.3% yield). The peak of the pure compound was detected by the LCMS. Calculated mass for **40** [M] = 603.19, found in LCMS = 604.

Synthesis of compound (41).

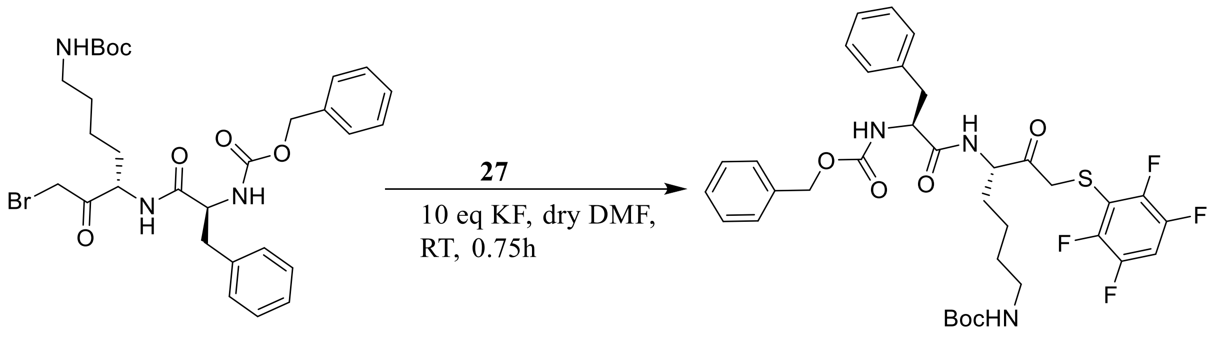

40 **41. (GD10)**

Compounds **40** (1 equivalent, 10 mg, 0.0165 mmol) and **27** (1 equivalent) were dissolved in dry DMF under argon at room temperature. KF (10 equivalents) was then added to the mixture and left to stir at room temperature for 0.75 hours. Following the workup in a separating funnel using a water-ethyl acetate combination, the light orange-colored solution was put into ethyl acetate and dried over sodium sulfate, and evaporated using rotavap. The resulting mixture was purified using HPLC using an acetonitrile/water gradient. The purified material was freeze-dried in the lyophilizer and subjected to LCMS (2 mg, 0.00284 mmol, 17.13% yield). The LCMS data showed the peak of the pure chemical. Calculated mass for **GD10** [M-Boc+2H] = 606.2, found in LCMS = 606.42.

Synthesis of compound (42).

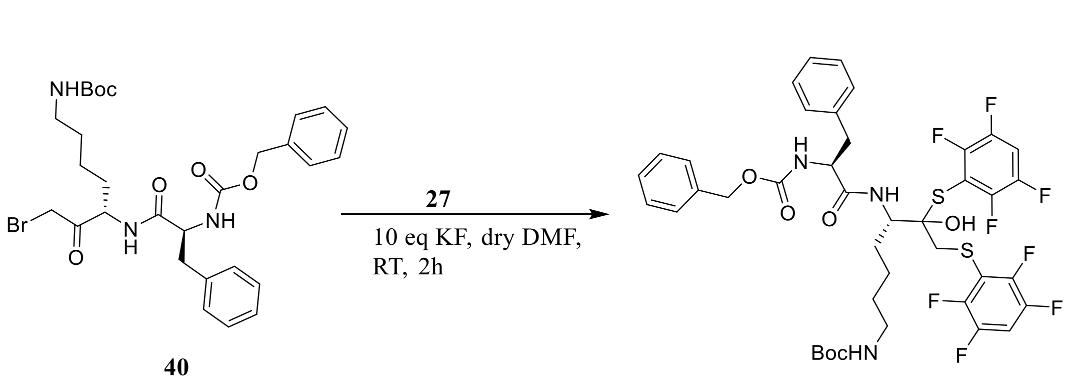

40

42. (GD11)

Compounds **40** (1 equivalent, 10 mg, 0.0165 mmol) and **27** (5 equivalent) were dissolved in dry DMF under argon at room temperature. KF (10 equivalents) was then added to the mixture and left to stir at room temperature for 2 hours. Following the workup in a separating funnel using a water ethyl acetate combination, the light yellow colored solution was put into ethyl acetate and dried over sodium sulfate, then evaporated using rotavapor. The resulting mixture was purified by HPLC using an acetonitrile/water gradient (3 mg, 0.00337 mmol, 20.4% yield). The purified material was freeze-dried and subjected to LCMS. The LCMS data showed the peak of the pure chemical. Calculated mass for **GD11** [M-Boc+H] = 787.18, found in LCMS = 786.5.

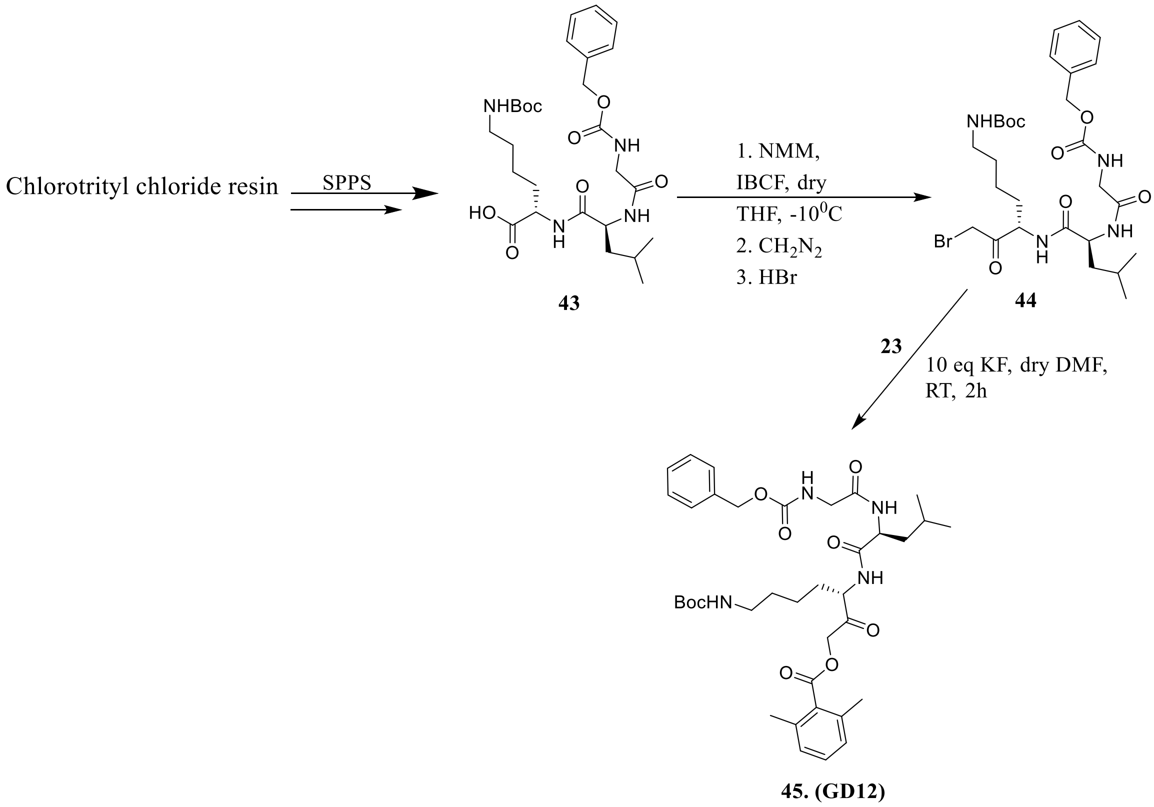

Scheme S5. Synthetic scheme of compound **GD12.**

Synthesis of peptide (43).

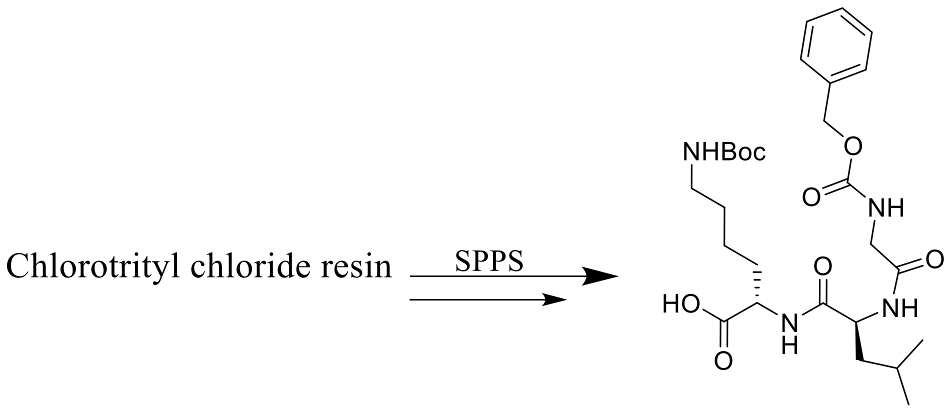

43

Chlorotrityl chloride resin was loaded with Fmoc-Lysine (Boc)-OH (1.5 equivalents, 800 mg, 1.45 mmol) and diisopropylethylamine (DIEA) for 2 hours with vigorous shaking using dry dichloromethane as the solvent. Methanol (1 ml/gr) was added to the reaction mixture and agitated for another 30 minutes before being rinsed multiple times with DMF and DCM. To remove the Fmoc protective group of lysine, 20% piperidine/DMF (v/v) was added to the resin and incubated for 40 minutes. After that, the resin was washed multiple times with DMF and DCM and the peptide was elongated by adding a solution of Fmoc-Leucine-OH (3 equivalents), DIPEA (3 equivalents), hydroxybenzotriazole (HOBT; 3 equivalents), and HATU (3 equivalents) in DMF for 6 hours. The resin was washed numerous times with DMF and DCM. Fmoc was then deprotected by using 20% piperidine/DMF (v/v) followed by peptide elongation with a solution of N -benzyloxycarbonyl-glycine (3 equivalents), DIPEA (3 equivalents), hydroxybenzotriazole (HOBT; 3 equivalents), and HATU (3 equivalents). The final product was cleaved from the resin using 5% TFA in DCM. Following solvent evaporation, the final product was purified by HPLC using an acetonitrile/water gradient. The overall yield is 64% relative to resin loading. The peptide's purity was determined using LCMS. Calculated mass for **43** [M + H] = 551.31, found in LCMS = 551.8.

Synthesis of compound (44).

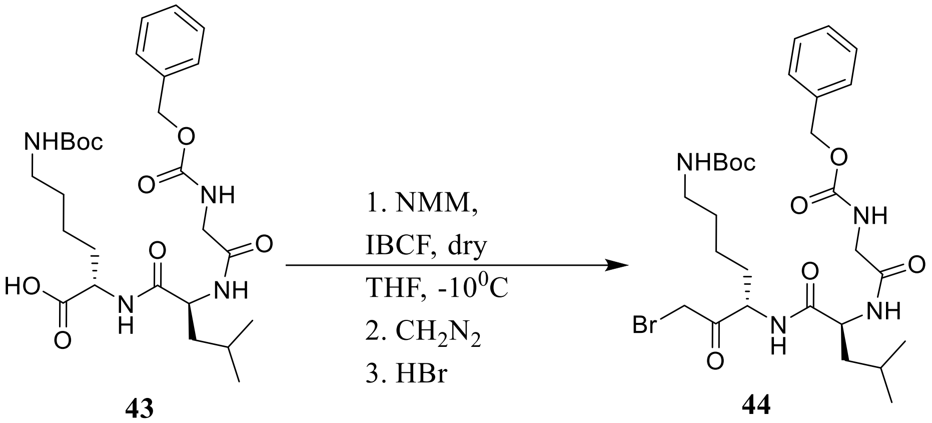

43

44

Compound **43** (1 equivalent, 200 mg, 0.363 mmol) was dissolved in dry THF at a temperature of -10 °C in an inert atmosphere with argon. Isobutyl chloroformate (IBCF; 1.15 equivalent) and N-methyl morpholine (NMM; 1.25 equivalent) were added in turn. For two hours, the solution was left to swirl at -10° C. Then, at -10° C, excess ethereal diazomethane (14 eq.) that was synthesized on-site was added to the anhydride. The resulting mixture was stirred at -10° C for one hour. The temperature was increased to Rt over 2 hours. A solution of 1:1 water: 37% HBr was added dropwise to the reaction mixture at 0° C. The organic layer was diluted by the addition of ethyl acetate. The liquid was then given a final water addition before being put into a separating funnel to collect the organic layer. The organic layer was concentrated by rotavap and dried over sodium sulfate. The product was purified using HPLC with an acetonitrile/water gradient (140 mg, 0.223 mmol, 61.4% yield). The product was freeze-dried before analysis by LCMS. The peak of the pure chemical was seen in the LCMS data. Calculated mass for **44** [M] = 626.23, found in LCMS = 626.92.

Synthesis of peptide (45).

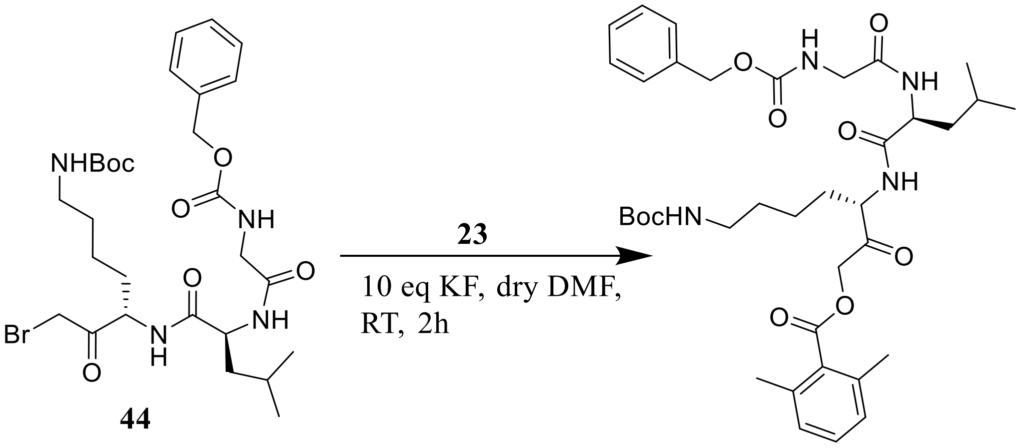

44

45. (GD12)

Compounds **44** (1 equivalent, 15 mg, 0.0239 mmol) and **23** (1 equivalent) were dissolved in dry DMF at room temperature under argon environment. KF (10 equivalents) was then added to the mixture and left to stir at room temperature for 2 hours. Following the workup in a separating funnel using a water ethyl acetate combination, the yellowish-green colored solution was put into ethyl acetate. The organic layer was recovered and dried over sodium sulfate and then evaporated using rotavap. The reaction mixture was purified using HPLC using an acetonitrile/water gradient. The product was freeze-dried before analysis by LCMS (4 mg, 0.00574 mmol, 24% yield). The LCMS data showed the peak of the pure chemical. Calculated mass for **GD12** [M] = 696.37, found in LCMS = 697.17.

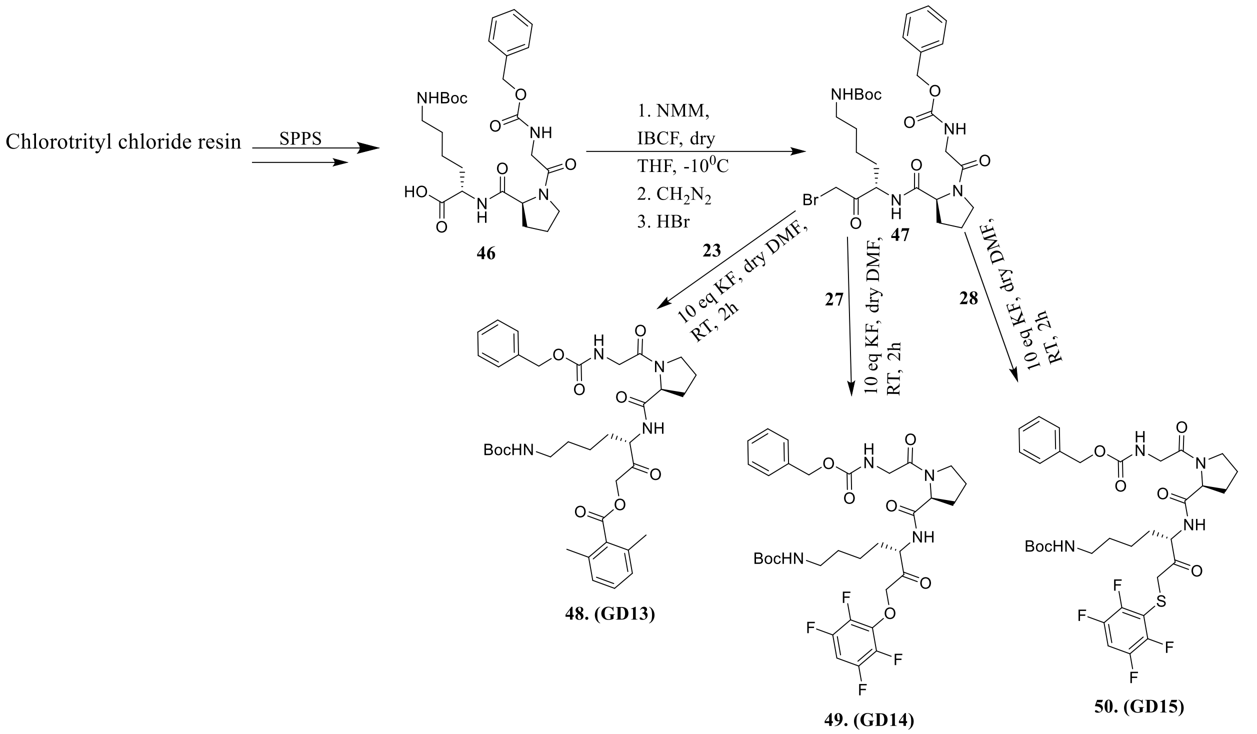

Scheme S6. Synthetic scheme of compounds **GD13 – GD15.**

Synthesis of compound (46)

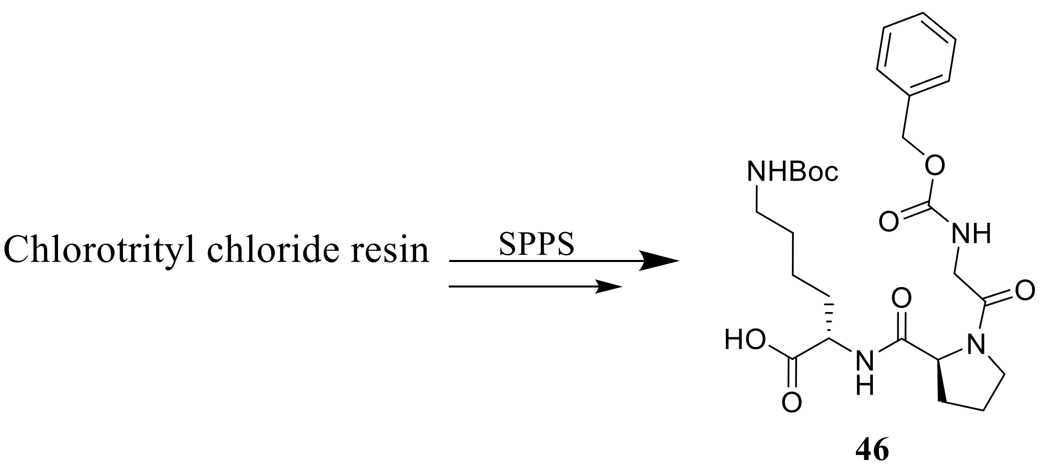

46

Chlorotrityl chloride resin was loaded with Fmoc-Lysine (Boc)-OH (1.5 equivalents, 2 gr, 4.26 mmol) and diisopropylethylamine (DIEA, 3 equivalents) for 2 hours with vigorous shaking using dry DCM as the solvent. Methanol (1 ml/gr) was added to the reaction mixture and agitated for another 30 minutes before being rinsed multiple times with DMF and DCM. To remove the Fmoc protective group of lysine, 20% piperidine/DMF (v/v) was added to the resin

and incubated for 40 minutes. After that, the resin was washed multiple times with DMF and DCM before the peptide was elongated by adding a solution of Fmoc-Proline -OH (3 equivalents), DIPEA (3 equivalents), hydroxybenzotriazole (HOBT; 3 equivalents), and HATU (3 equivalents) for 6 hours in DMF. The resin was washed numerous times with DMF and DCM. Then Fmoc was deprotected by using 20% piperidine/DMF (v/v) followed by washing and adding a solution N -benzyloxycarbonyl-glycine (3 equivalents), DIPEA (3 equivalents), hydroxybenzotriazole (HOBT; 3 equivalents), and HATU (3 equivalents). The product was cleaved from the resin by a 5% TFA in DCM solution. Following rotavapor evaporation, the final product was purified by HPLC using an acetonitrile-water gradient. The peptide's purity was determined using LCMS. The overall yield is 64% relative to the resin loading. Calculated mass for **46** [M] = 534.27, found in LCMS = 535.08.

Synthesis of compound (47)

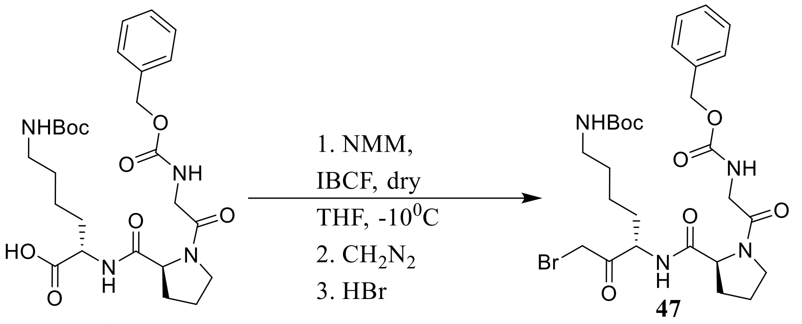

46 47

Compound **46** (1 equivalent, 240 mg, 0.449 mmol) was dissolved in dry THF at a temperature of -10 °C in an inert atmosphere with argon. Isobutyl chloroformate (IBCF; 1.15 equivalents) and N-methyl morpholine (NMM; 1.25 equivalents) were added in turn. The solution was left to swirl at -10° C for two hours; then, excess ethereal diazomethane (14 equivalents) that had been synthesized on-site was added to the anhydride. The resulting mixture was stirred at -10° C for an additional hour. The temperature reached RT over 2 hours. A solution of 1:1 water: 37% HBr was added dropwise to the reaction mixture at 0° C. The organic layer was diluted

by the addition of ethyl acetate. Water was added and mixed, the product was extracted to the organic layer that was separated using a sep-funnel. The organic layer was dried over sodium sulfate and concentrated in with a rotavap. The product was purified using HPLC with an acetonitrile/water gradient. The product was freeze-dried, and the purified compound was analyzed by LCMS (178.2 mg, 0.292 mmol, 65% yield). The peak of the pure chemical was detected by the LCMS. Calculated mass for **47** [M] = 610.2, found in LCMS = 611.

Synthesis of compounds (48), (49), and (50).

For the synthetic route, see **Scheme S6** above.

Compounds **47** (1 equivalent, 20 mg, 0.0327 mmol) and **23** (5 equivalents) or **27** (5 equivalents) or **28** (1 equivalent) were dissolved in dry DMF at room temperature under argon environment. KF (10 equivalents) was then added to the mixture and left to stir at room temperature for 2 hours. Following the workup in a separating funnel using water and ethyl acetate, the yellowish green colored (for **48**) or orange colored (for **49**, and **50**) solution was put into ethyl acetate. The organic layer was recovered, dried over sodium sulfate, and evaporated using a rotavap. The resulting mixtures were purified using HPLC using an acetonitrile/water gradient. The purified materials were freeze-dried before being analysis by LCMS (for **48**, 5.57 mg, 0.00818 mmol, 25% yield), (for **49**, 3 mg, 0.0043 mmol, 12.2% yield), (for **50**, 6 mg, 0.000842 mmol, 25% yield). The LCMS data showed the peaks of the pure compounds. Calculated mass for **GD13** [M] = 680.34, found in LCMS = 681.17. Calculated mass for **GD14** [M] = 696.27, found in LCMS = 697.08. Calculated mass for **GD15** [M] = 712.25, found in LCMS = 713.08.

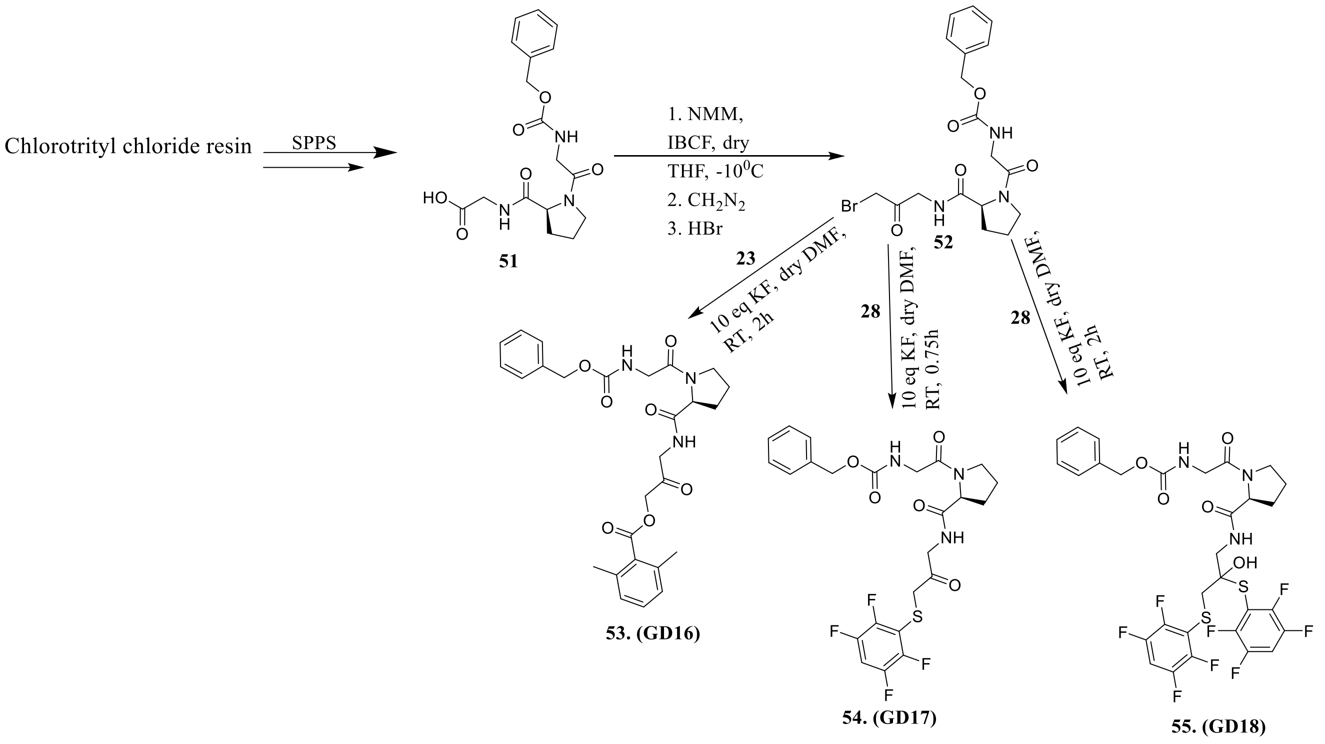

Scheme S7. Synthetic scheme of compounds **GD16 – GD18.**

Synthesis of peptide (51)

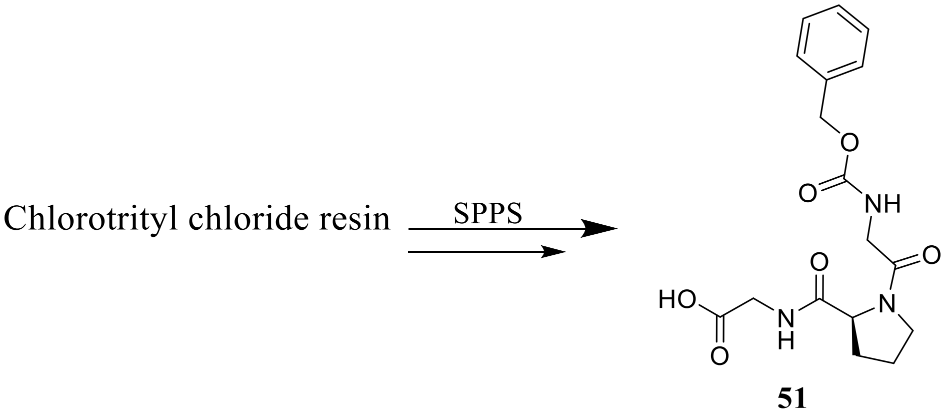

51

Chlorotrityl chloride resin was loaded with Fmoc-Glycine-OH (1.5 equivalent, 2 gr, 6.72 mmol), and SPPS was followed as described above method for compound **46**, coupling Fmoc Proline and then N-benzyloxcarbonyl-Glycine. The final product was purified in HPLC using an acetonitrile/water gradient. The overall yield is 65% relative to the resin loading. The peptide's exact mass was determined using LCMS. Calculated mass for **51** [M] = 363.14, found in LCMS = 364.08.

Synthesis of compound (52)

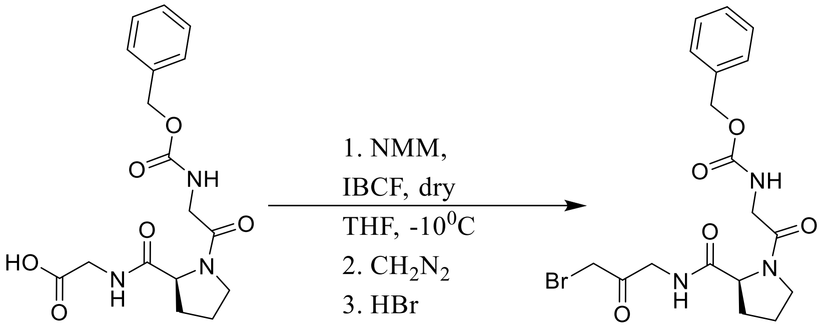

51 52

Compound **51** (1 equivalent, 230 mg, 0.633mmol) was dissolved in dry THF at a temperature of -10 °C in an inert atmosphere under argon. Isobutyl chloroformate (IBCF; 1.15 equivalents) and N-methyl morpholine (NMM; 1.25 equivalents) were added in turn. The same procedure was used as for the generation of compounds **44** and **47**. The product was purified using HPLC with an acetonitrile/water gradient. The product was freeze-dried, and the purified compound was analyzed by LCMS (120 mg, 0.272 mmol, 43% yield). The peak of the pure compound was seen in the LCMS data. Calculated mass for **52** [M] = 440.28, found in LCMS = 440.08.

Synthesis of compounds (53), (54), and (55).

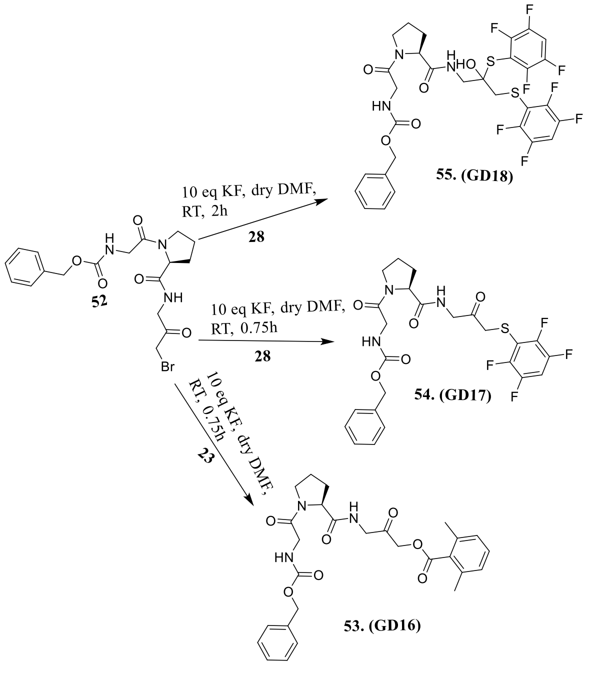

Compounds **52** (1 equivalent, 20 mg, 0.045 mmol) and **23** (5 equivalents), or **28** (5 equivalents) or **28** (1 equivalent) were dissolved in dry DMF at room temperature under an argon. KF (10 equivalents) was then added to the mixture and left to stir at room temperature for 2 hours (for **53** and **55**), and 0.75 hours for **54**. The procedure was followed as mentioned above for **50**. The product was purified using HPLC with an acetonitrile/water gradient. The purified materials were freeze-dried and analyzed by LCMS (for **53**, 15 mg, 0.0294 mmol, 65% yield), (for **54**, 11 mg, 0.0203 mmol, 45% yield), (for **55**, 6 mg, 0.00829 mmol, 18.4% yield). The LCMS data showed the peaks of the pure chemicals. Calculated mass for **GD16** [M+H] = 510.22, found in LCMS = 510.25. Calculated mass for **GD17** [M+H] = 542.12, found in LCMS = 542.17. Calculated mass for **GD18** [M] = 723.11, found in LCMS = 723.08.

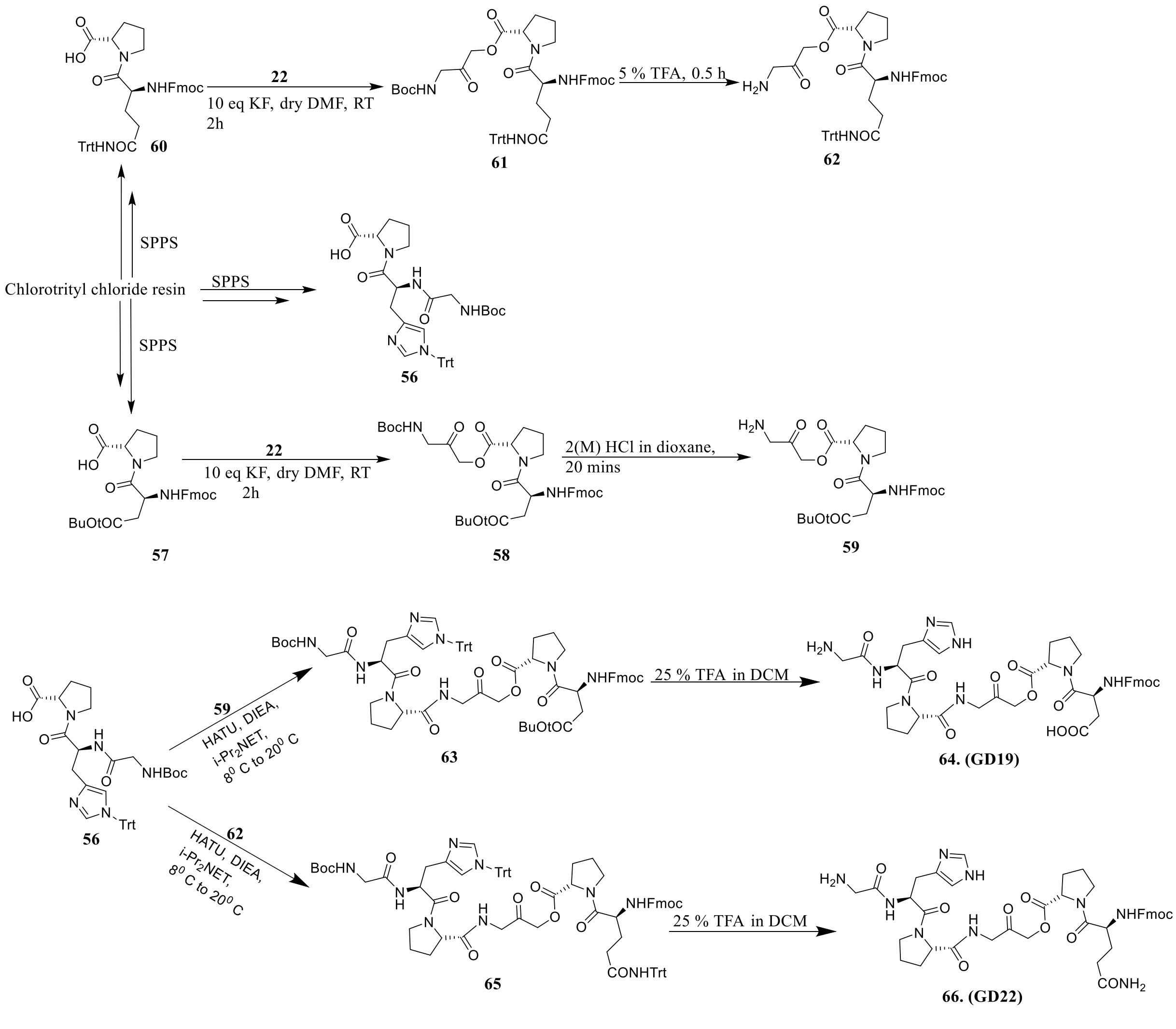

Scheme S8. Synthesis scheme of compounds **GD19**, and **GD22**.

Synthesis of peptide (56).

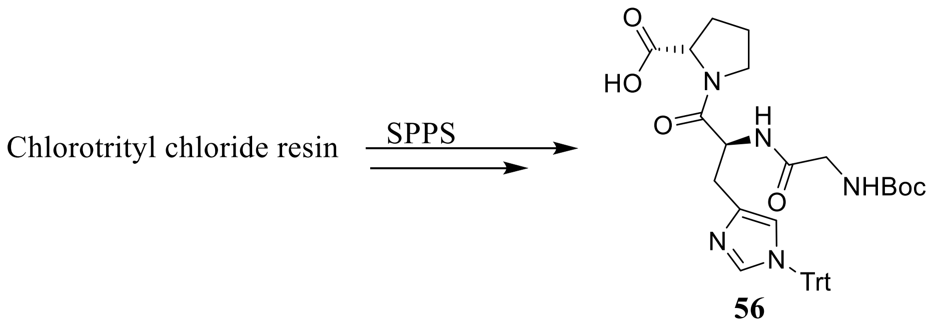

56

Chlorotrityl chloride resin was loaded with Fmoc-Proline-OH (1.5 equivalent, 2 gr, 5.92 mmol) and SPPS was followed as described above for compound **46**, coupling with Fmoc-Histidine(Trt) and NHBoc-Glycine. The final product was purified by HPLC using an acetonitrile/water gradient. The overall yield is 55% relative to the resin loading. The peptide's exact mass was determined using LCMS. Calculated mass for **56** [M+H] = 652.31, found in LCMS = 652.33.

Synthesis of peptide (57).

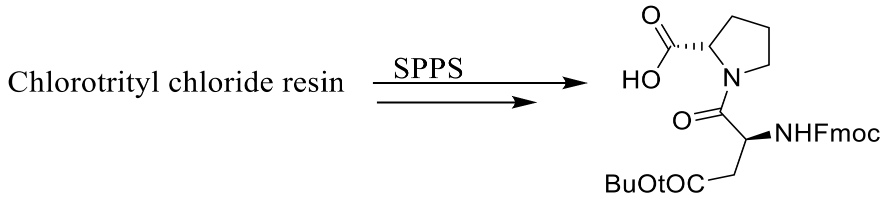

57

Chlorotrityl chloride resin was loaded with Fmoc-Proline-OH (1.5 equivalent, 2 gr, 5.92 mmol), and SPPS was followed as described above for compound **46**, coupling with Fmoc-Glu(OtBu). The final product was purified in HPLC using an acetonitrile/water gradient. The

overall yield is 45% relative to the resin loading. The peptide's exact mass was determined using LCMS. Calculated mass for **57** [M] = 508.22, found in LCMS = 509.

Synthesis of compound (58).

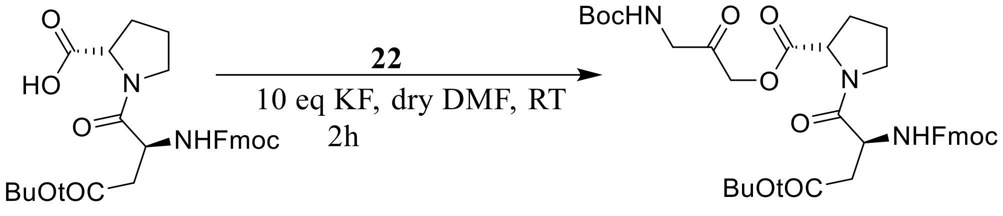

57 **58**

Compounds **22** (1 equivalent, 20 mg, 0.079 mmol) and **57** (5 equivalent) were dissolved in dry DMF at room temperature under argon. KF (10 equivalents) was then added to the mixture and left to stir at room temperature for 2 hours. The procedure was followed as mentioned above for compound **50**. The resulting mixture was purified by HPLC using an acetonitrile/water gradient. The purified materials were freeze-dried and analyzed by LCMS (15 mg, 0.022 mmol, 28% yield). The LCM data showed the peak of the pure chemical. Calculated mass for **58** [M] = 679.31, found in LCMS = 680.08.

Synthesis of compound (59).

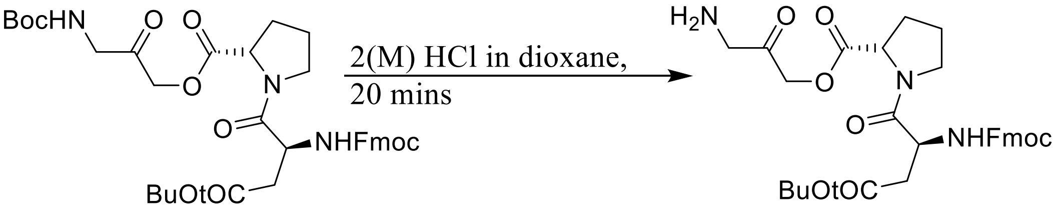

58 **59**

The Boc group was removed by stirring the **58** (13 mg, 0.01913 mmol) in 2 (M) HCl solution in dioxane for 20 mins at RT. The progress of the reaction was monitored by LCMS. The crude was used in next step without further purification (4.4 mg, 0.0076 mmol, 40% yield). Calculated mass for **59** [M] = 580.26, found in LCMS = 580.25.

Synthesis of peptide (60).

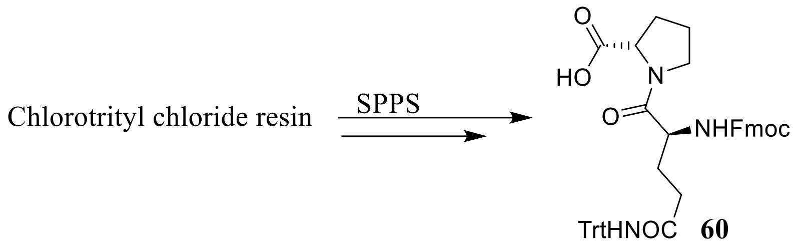

60

The chlorotrityl chloride resin was loaded with Fmoc-Proline-OH (1.5 equivalents, 2 gr, 5.92 mmol) and SPPS was followed as described above for compound **46**, coupling Fmoc-Gln(Trt). The final product was purified by HPLC using an acetonitrile/water gradient. The overall yield is 72% relative to the resin loading. The peptide's exact mass was determined by LCMS. Calculated mass for **60** [M] = 707.3, found in LCMS = 708.08.

Synthesis of compound (61).

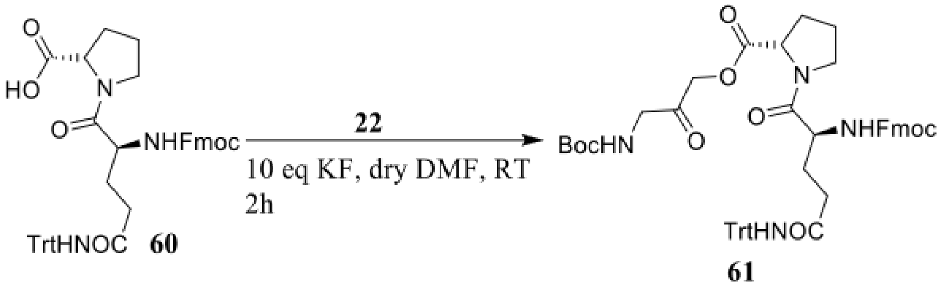

61

Compounds **22** (1 equivalent, 10 mg, 0.039 mmol) and **60** (3 equivalents) were dissolved in dry DMF at room temperature under an argon environment. KF (10 equivalents) was then added to the mixture and left to stir at room temperature for 2 hours. The procedure was followed as mentioned above for compound **50**. The resulting mixture was purified by HPLC using an acetonitrile/water gradient. The purified materials were freeze-dried and analyzed by LCMS (18 mg, 0.02049 mmol, 52% yield). The LCMS data showed the peak of the pure chemical. Calculated mass for **61** [M] = 878.39, found in LCMS = 879.17.

Synthesis of compound (62).

The Boc group was removed by stirring the **61** (18 mg, 0.02049 mmol) in 5% TFA solution in DCM for 30 mins at RT. The progress of the reaction was monitored by LCMS. The crude was collected after evaporating the solvent and used in the next step without further purification (13.5 mg, 0.0173 mmol, 84% yield). Calculated mass for **62** [M+H] = 779.34, found in LCMS = 779.42.

Synthesis of compound (63).

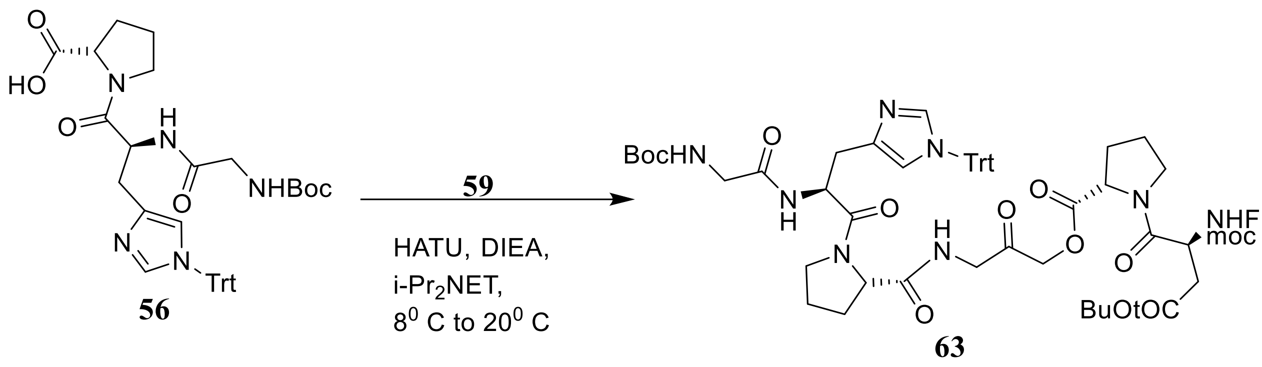

Compound **56** (1 equivalent, 4.5 mg, 0.0069 mmol) was put in ice-cooled dimethyl acetamide in a round-bottom flask. Compound **59** (1.3 equivalent) and HATU (4 equivalent) were added tothe solution. The procedure was followed as described before (for compounds **36**, **37**, and **38**). The compound was purified by HPLC using a water/acetonitrile gradient. The purified compound **(63)** was freeze-dried before being analyzed by LCMS, and was used for the next step (3 mg, 0.00247 mmol, 35.7% yield). Calculated mass for **63** [M+H] = 1213.56, found in LCMS = 1213.75.

Synthesis of compound (64).

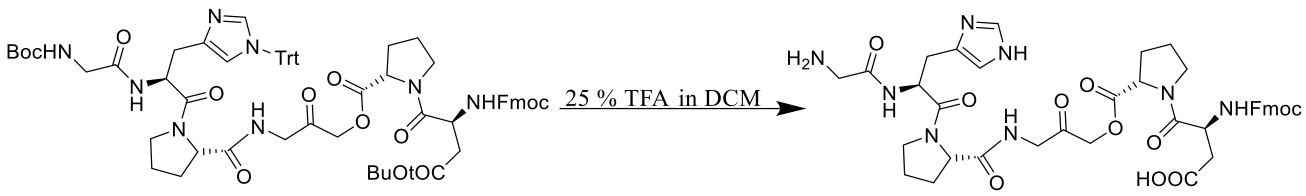

63

64. (GD 19)

The Boc, trityl, and tertiary butyl protecting groups were removed by stirring **63** (2 mg, 0.001646 mmol) in 25% TFA solution in DCM for 30 mins at RT. The progress of the reaction was monitored by LCMS. The crude was collected after evaporating the solvent and purified by HPLC using an acetonitrile/water gradient. The purified material was freeze-dried and analyzed by LCMS (0.75 mg, 0.000921 mmol, 55.9% yield). Calculated mass for **GD19** [M+H] = 815.33, found in LCMS = 815.42.

Synthesis of compound (65).

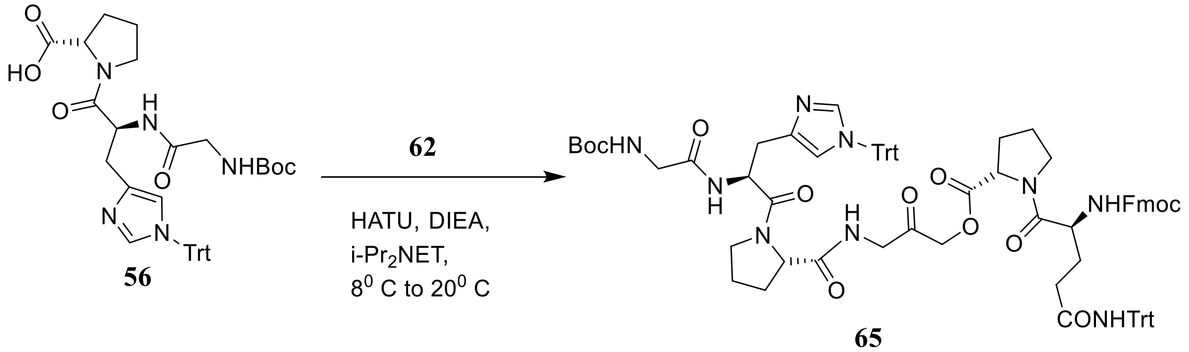

56

65

Compound **56** (1 equivalent, 7.72 mg, 0.0118 mmol) was put in ice-cooled dimethyl acetamide in a round-bottom flask. Compound **62** (1.3 equivalent) and HATU (4 equivalent) were added tothe solution. The procedure was followed as described before (for compounds **36**, **37**, and **38**). The compound was purified by HPLC using a water/acetonitrile gradient. The purified compound **(65)** was finally freeze-dried before analysis by LCMS and use for the next step (7.4 mg, 0.0052 mmol, 44% yield). Calculated mass for **65** [M+H] = 1412.64, found in LCMS = 1413.25.

Synthesis of compound (66).

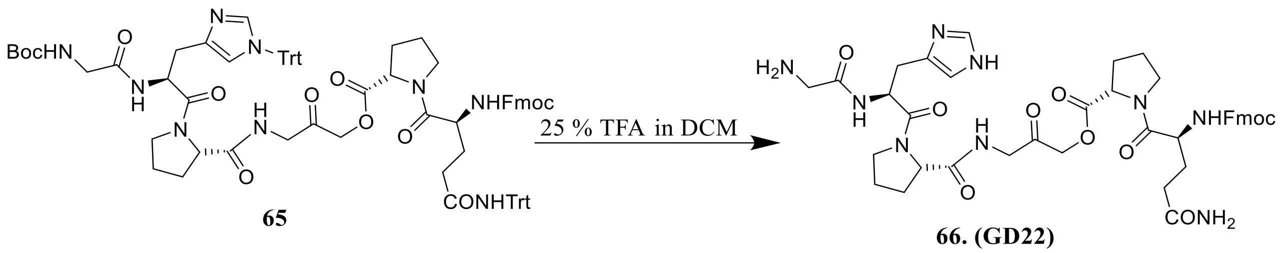

65 **66. (GD22)**

The Boc and trityl, groups were removed by stirring the **65** (6 mg, 0.00425 mmol) in 25% TFA solution in DCM for 30 mins at RT. The progress of the reaction was monitored by LCMS. The compound (**66**) was purified by HPLC using an acetonitrile/water gradient. The purified materials were freeze-dried and analyzed by LCMS (2 mg, 0.00241 mmol, 57% yield). Calculated mass for **GD22** [M+H] = 828.37, found in LCMS = 828.42.

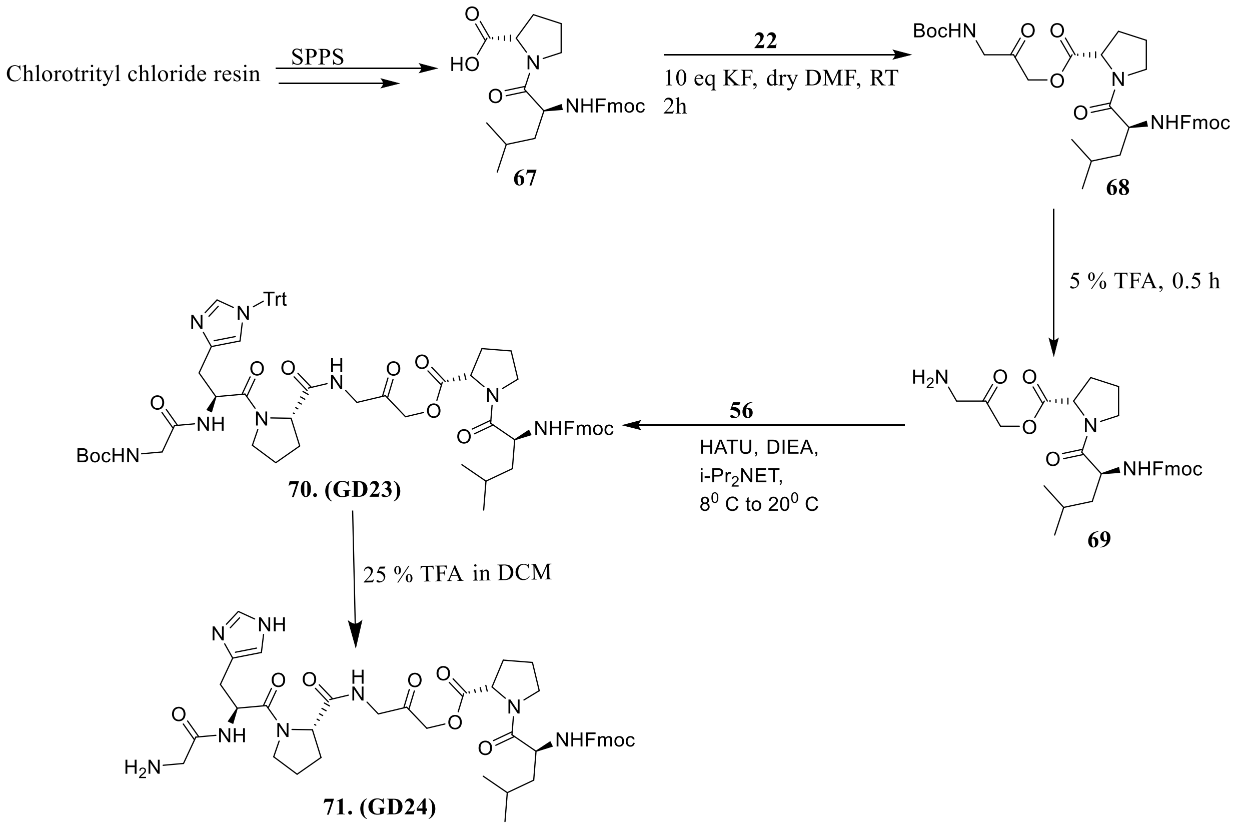

67 **68** **69** **70. (GD23)** **71. (GD24)**

Scheme S9a. Synthesis scheme of compounds **GD23**, and **GD24**.

Synthesis of peptide (67).

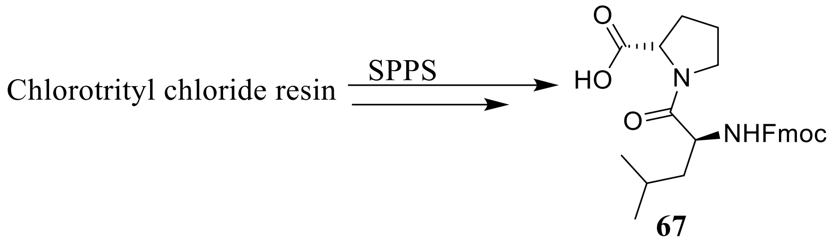

67

Chlorotrityl chloride resin was loaded with Fmoc-Proline-OH (1.5 equivalent, 2 gr, 5.92 mmol) and SPPS was followed as described above for **46**, coupling with Fmoc-Leucine. The final product was purified by HPLC using an acetonitrile/water gradient. The overall yield was 80% relative to the resin loading. The peptide's exact mass was determined using LCMS. Calculated mass for **67** [M+H] = 451.2, found in LCMS = 451.21.

Synthesis of compound (68).

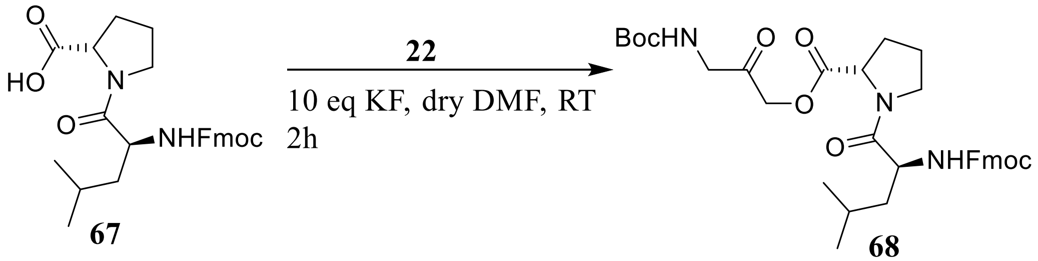

67 **68**

Compounds **22** (1 equivalent, 80 mg, 0.318 mmol) and **67** (3 equivalents) were dissolved in dry DMF at room temperature under an argon environment. KF (10 equivalents) was then added to the mixture and left to stir at room temperature for 2 hours. The procedure was followed as mentioned above for compound **50**. The resulting mixture was purified by HPLC using an acetonitrile/water gradient. The purified material was freeze-dried and analyzed by LCMS (128 mg, 0.2056 mmol, 64.6% yield). The LCM data showed the peak of the pure chemical. Calculated mass for **68** [M] = 622.31, found in LCMS = 622.08.

Synthesis of compound (69).

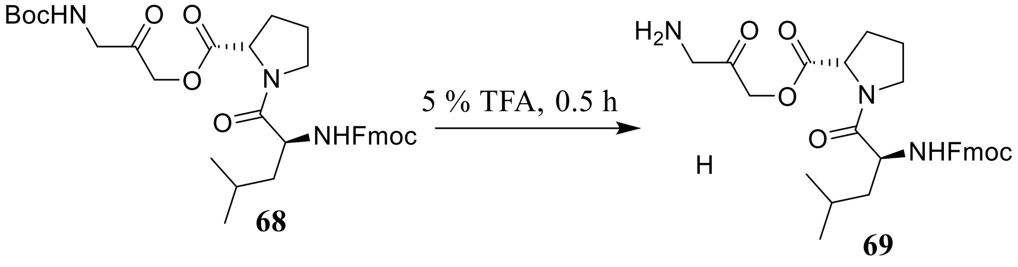

The Boc protecting group was removed by stirring the **68** (128 mg, 0.2056 mmol) in 5% TFA solution in DCM for 30 mins at RT. The progress of the reaction was monitored by LCMS. The crude was collected after evaporating the solvent and purified by HPLC using an acetonitrile/water gradient. The purified materials were freeze-dried and analyzed by LCMS

(80 mg, 0.152 mmol, 74% yield). Calculated mass for **69** [M+H] = 522.26, found in LCMS = 522.33.

Synthesis of compound (70).

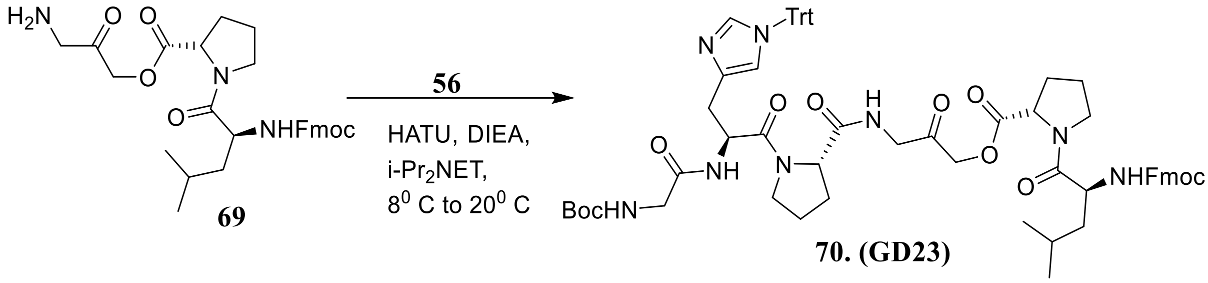

Compound **56** (1 equivalent, 9.53 mg, 0.014 mmol) was put in ice-cooled dimethyl acetamide in a round-bottom flask. Compound **69** (1.3 equivalents) and HATU (4 equivalents) were added to the solution. The procedure was followed as described before (for compounds **36**, **37**, and **38**). The compound was purified by HPLC using a water/acetonitrile gradient. The purified compound **(70)** was analyzed by LCMS, freeze dried before further use (7.6 mg, 0.006582 mmol, 47% yield). Calculated mass for **GD23** [M+H] = 1155.56, found in LCMS = 1155.75.

Synthesis of compound (71).

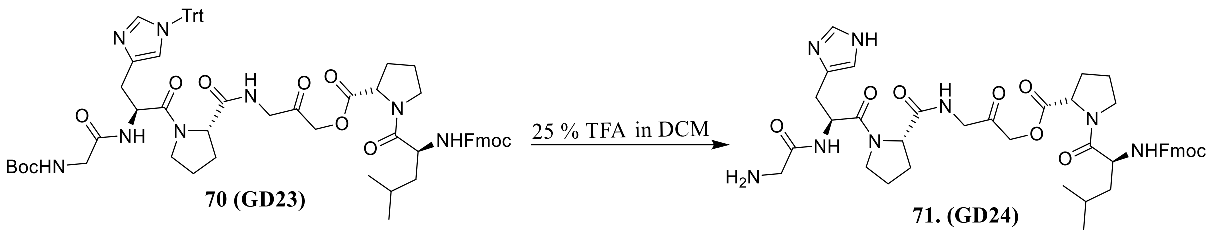

The Boc and trityl groups were removed by stirring the **70** (4.5 mg, 0.00389 mmol) in 25% TFA solution in DCM for 30 mins at RT. The progress of the reaction was monitored by LCMS. The crude was collected after evaporating the solvent and purified by HPLC using an acetonitrile/water gradient. The purified materials were freeze-dried and analyzed by LCMS (1.2 mg, 0.00147 mmol, 38% yield). Calculated mass for **GD24** [M+H] = 813.39, found in LCMS = 813.58.

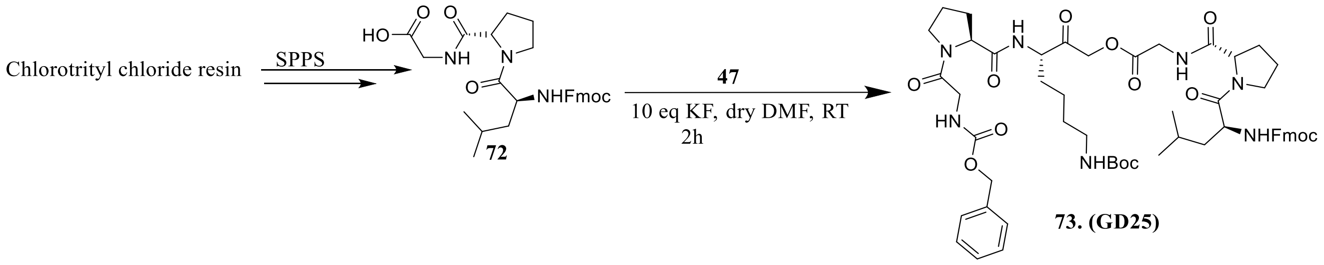

Scheme S9b. Synthesis scheme of compounds **GD25**.

Synthesis of peptide (72)

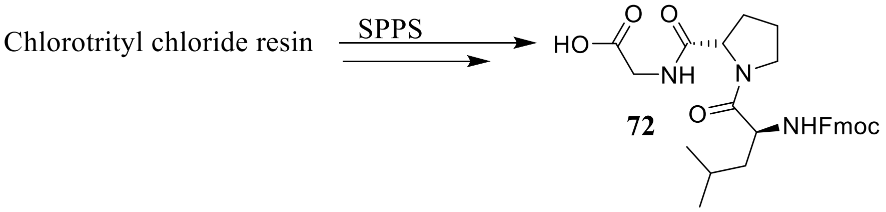

Chlorotrityl chloride resin was loaded with Fmoc-Glycine-OH (1.5 equivalent, 2 gr, 6.72 mmol) and SPPS was followed as described above for compound **46**, coupling with Fmoc-Proline and Fmoc-Leucine. The final product was purified by HPLC using an acetonitrile/water gradient. The overall yield is 66% relative to the resin loading. The peptide's exact mass was determined using LCMS. Calculated mass for **72** [M+H] = 508.24, found in LCMS = 508.25.

Synthesis of Compound (73)

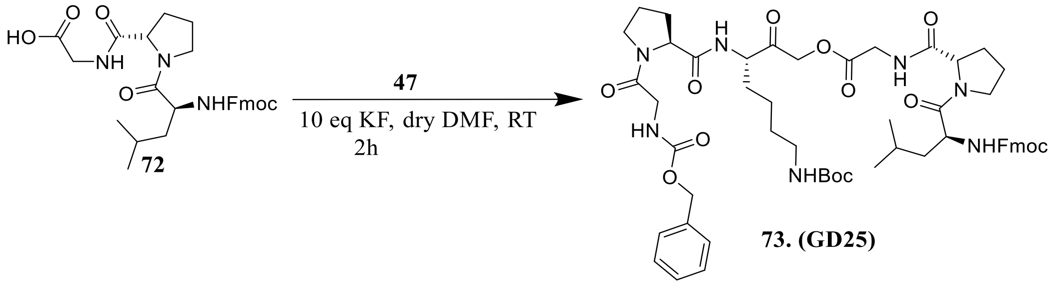

Compounds **47** (1 equivalent, 20 mg, 0.0327 mmol) and **72** (3 equivalents) were dissolved in dry DMF at room temperature under an argon environment. KF (10 equivalents) was then added to the mixture and left to stir at room temperature for 2 hours The procedure was followed as mentioned above for compound **50**. The resulting mixture was purified by HPLC using an acetonitrile/water gradient. The purified materials were freeze-dried and analyzed by LCMS (7 mg, 0.00674 mmol, 20.6% yield). The LCMS data showed the peak of the pure chemical. Calculated mass for **GD25** [M] = 1037.51, found in LCMS = 1038.33.

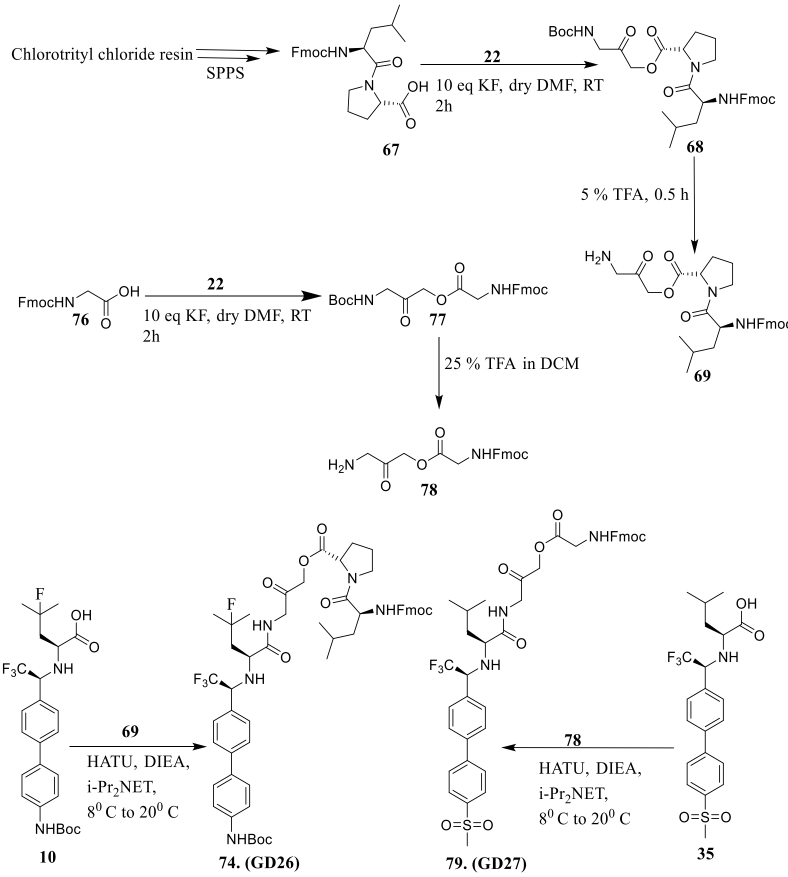

Scheme S10. Synthesis scheme of compounds **GD26 and GD27**.

Synthesis of compound (74).

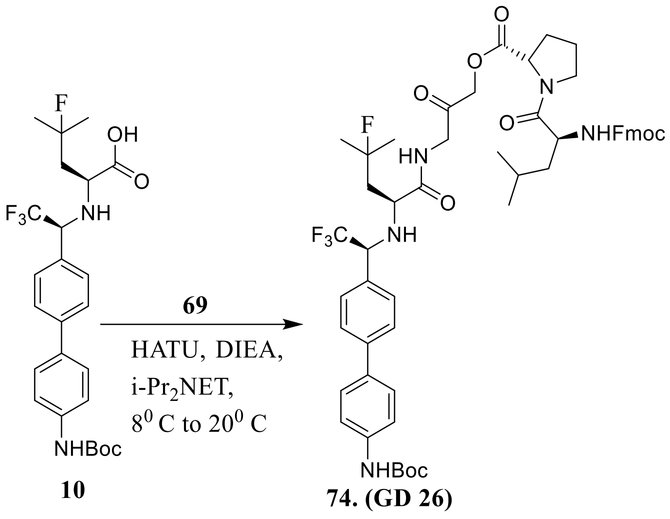

Compound **10** (1 equivalent, 10 mg, 0.02 mmol) was put in ice-cooled dimethyl acetamide in a round-bottom flask. Compound **69** (1.3 equivalents) and HATU (4 equivalents) were added to the solution. The procedure was followed as described before compound **70**. The compound was purified by HPLC using a water/acetonitrile gradient. The purified compound **(74)** was freeze-dried before it was analyzed by LCMS and used for the next step (4 mg, 0.0039 mmol, 20% yield Calculated mass for **GD 26** [M+H] = 1002.46, found in LCMS = 1002.58.

Synthesis of Compound (75)

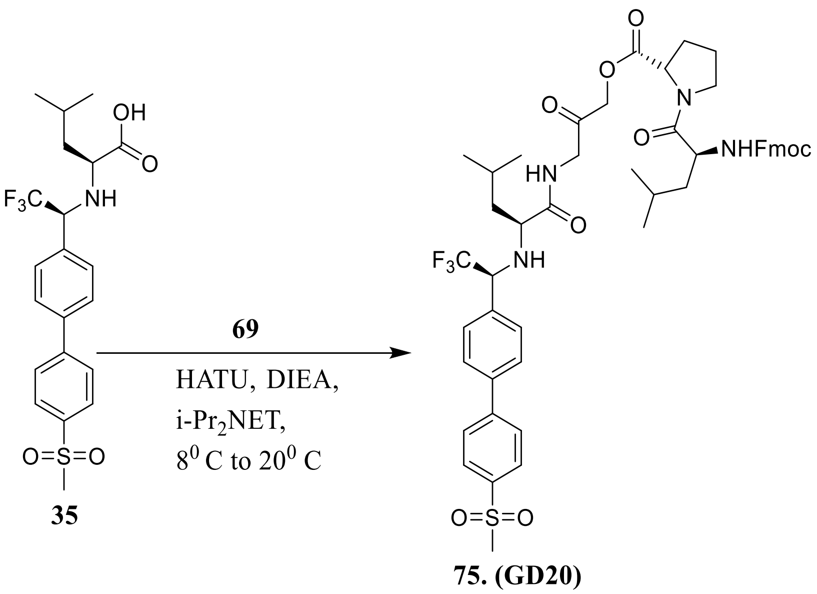

Compound **35** (1 equivalent, 40 mg, 0.09 mmol) was put in ice-cooled dimethyl acetamide in a round-bottom flask. Compound **69** (3 equivalents) and HATU (4 equivalents) were added to the solution. The procedure was followed as described before (for compounds **36**, **37**, and **38**). The compound was purified by HPLC using a water/acetonitrile gradient. The purified compound **(75)** was finally freeze-dried before it was used for the next step and analyzed by LCMS (19 mg, 0.02 mmol, 22.2% yield). Calculated mass for **GD20** [M+H] = 947.39, found in LCMS = 947.5.

Synthesis of compound (77).

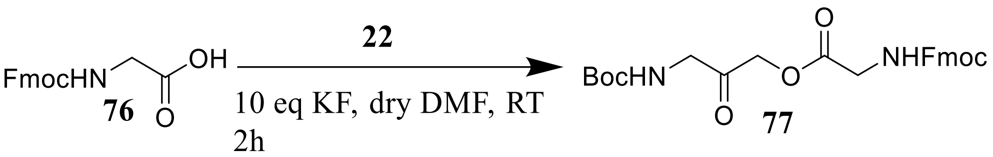

Compounds **22** (1 equivalent, 30 mg, 0.119 mmol) and **76** (5 equivalent) were dissolved in dry DMF at room temperature under argon. KF (10 equivalents) was then added to the mixture and left to stir at room temperature for 2 hours. The procedure was followed as mentioned for compound **50**. The resulting mixture was purified by HPLC using an acetonitrile/water gradient. The purified materials were freeze-dried and analyzed by LCMS (18 mg, 0.0384 mmol, 32.2% yield). The LCMS data showed the peak of the pure chemical. Calculated mass for **77** [M+H_2O] = 486.2, found in LCMS = 486.08.

Synthesis of compound (78).

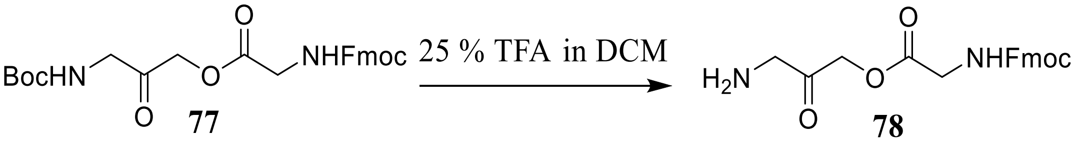

The Boc group was removed by stirring **77** (18 mg, 0.0384 mmol) in 25% TFA solution in DCM for 30 mins at RT. The progress of the reaction was monitored in LCMS. The crude was

collected after evaporating the solvent it was purified by HPLC using acetonitrile/water. The purified materials were freeze-dried and analyzed by LCMS (8 mg, 0.0217 mmol, 56.5% yield). Calculated mass for **78** [M] = 368.39, found in LCMS = 369.

Synthesis of compound (79).

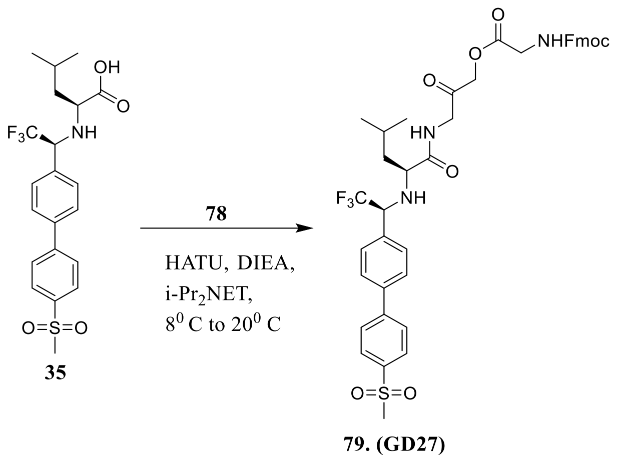

Compound **35** (1 equivalent, 7.5 mg, 0.0169 mmol) was taken in an ice-cooled solution of dimethyl acetamide in a round-bottom flask. Compound **78** (1.3 equivalents) and HATU (4 equivalents) were added to the solution. The procedure was followed as described before (for compounds **36**, **37**, and **38**). The compound was purified by HPLC using a water/acetonitrile gradient. The purified compound **(79)** was analyzed by LCMS and freeze-dried before it was used for next step (3 mg, 0.00377 mmol, 22.3% yield). Calculated mass for **GD27** [M] = 793.86, found in LCMS = 794.

Synthesis of Peptide (80)

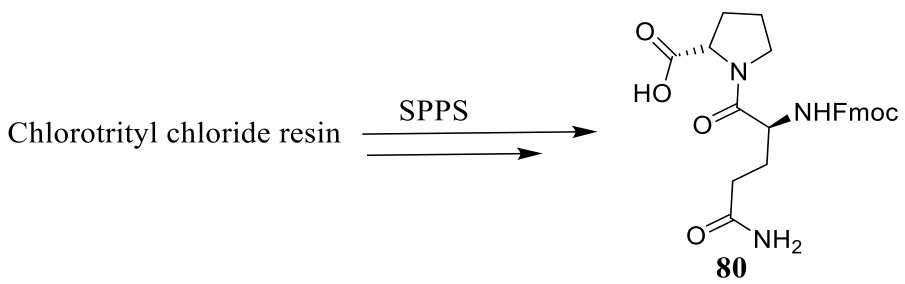

Chlorotrityl chloride resin was loaded with Fmoc-Proline-OH (1.5 equivalents, 2 gr, 5.92 mmol), and SPPS was followed as described above for compound **46**, coupling Fmoc-Gln (Trt). The final product was purified by HPLC using an acetonitrile/water gradient. The overall yield is 77% relative to the resin loading. The peptide's exact mass was determined using LCMS. Calculated mass for **80** [M] = 465.19, found in LCMS = 465.92.

Synthesis of Compound (81)

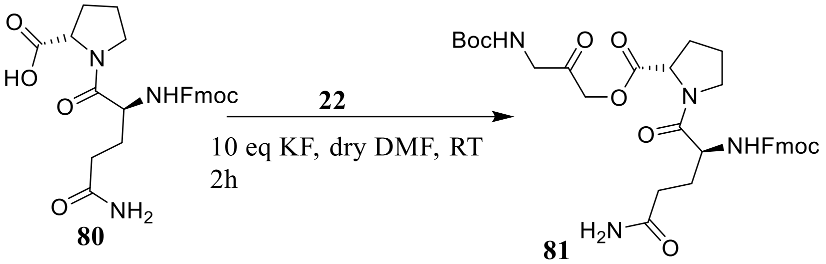

Compounds **22** (1 equivalent, 40 mg, 0.159 mmol) and **80** (3 equivalents) were dissolved in dry DMF at room temperature under an argon environment. KF (10 equivalents) was then added to the mixture and left to stir at room temperature for 2 hours. The procedure was followed as mentioned above for compound **50**. The resulting mixture was purified by HPLC using an acetonitrile/water gradient. The purified materials were freeze-dried and analyzed by LCMS (44 mg, 0.069 mmol, 44.3% yield). Calculated mass for **81** [M] = 636.28, found in LCMS = 636.75.

Synthesis of Compound (82)

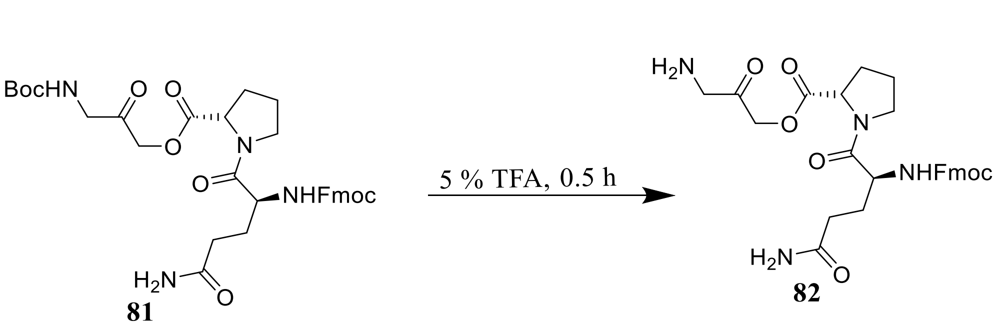

The Boc group was removed by stirring **81** (44 mg, 0.069 mmol) in a 5% TFA solution in DCM for 30 mins at RT. The progress of the reaction was monitored in LCMS. The crude was collected after evaporating the solvent and purified by HPLC using an acetonitrile/water gradient. The purified materials were freeze-dried and analyzed by LCMS (30 mg, 0.0559 mmol, 81% yield). Calculated mass for **82** [M] = 536.23, found in LCMS = 537.

Synthesis of Compound (83)

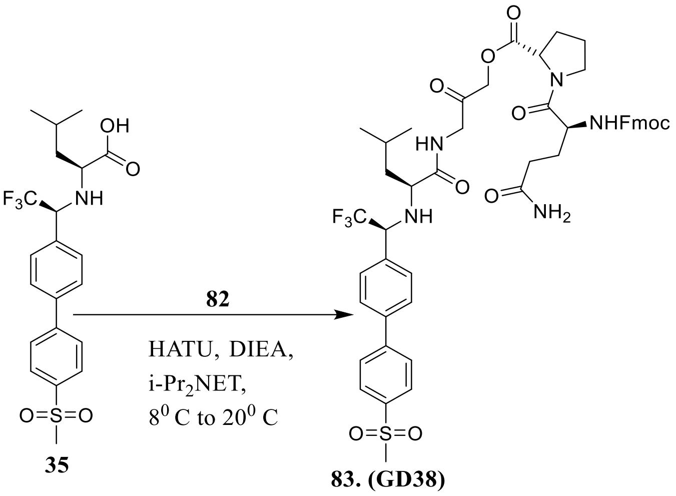

35 **83. (GD38)**

Compound **35** (1 equivalent, 19 mg, 0.0428 mmol) was put in ice-cooled dimethyl acetamide in a round-bottom flask. Compound **82** (1.3 equivalents) and HATU (4 equivalents) were added to the solution. The procedure was followed as described before (for compounds **36**, **37**, and **38**). The compound was purified by HPLC using a water/acetonitrile gradient. The purified compound **(83)** was analyzed by LCMS and freeze-dried before it was used for next step (22 mg, 0.0228 mmol, 53.28% yield). Calculated mass for **GD38** [M] = 961.35, found in LCMS = 962.

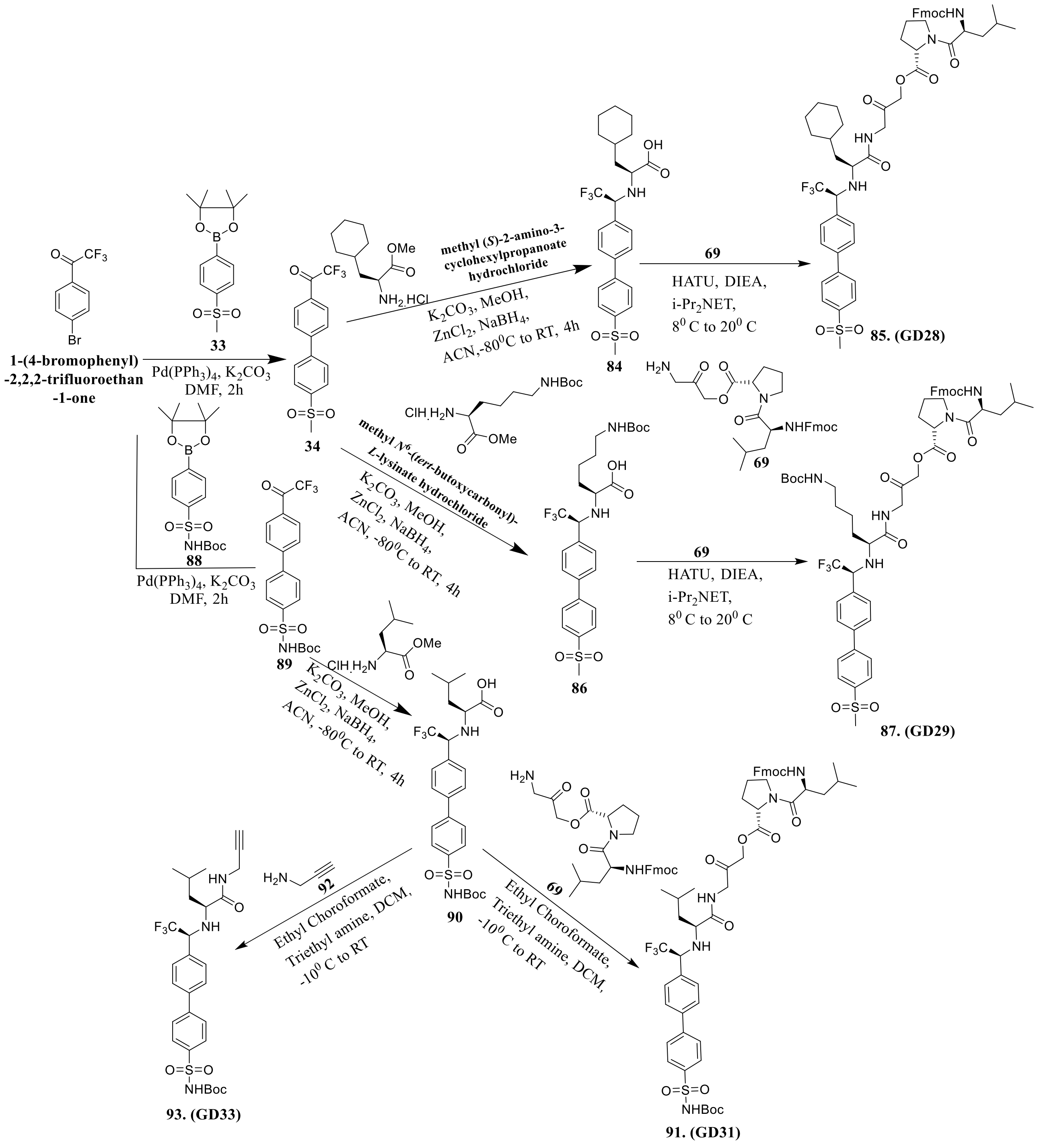

Scheme S11. Synthesis scheme of compounds **GD28, GD29, GD31, and GD33**.

Synthesis of compound (84).

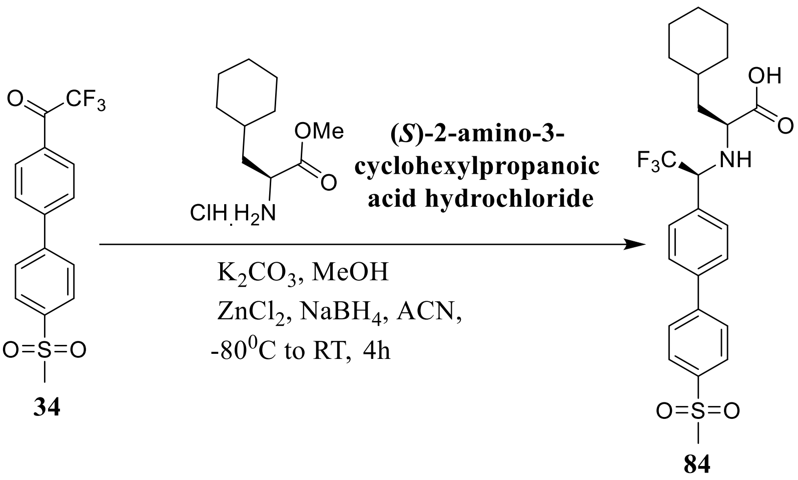

Anhydrous zinc chloride (2 equivalents) in dry DME was added to sodium borohydride (4 equivalents) at 0 °C and stirred at room temperature for 18 hours in a nitrogen environment. In the meanwhile, compound **34** (1 equivalent, 80 mg, 0.243 mmol) was heated to 55°C for 12 hours in a nitrogen environment with methyl ester salt of L-Cyclohexyl propionic acid (1.25 equivalents), potassium carbonate (3 equivalents), and dry methanol. The procedure was followed as mentioned before (for compound **9**). The compound (**84**) was purified by HPLC using a water/acetonitrile gradient. The compound was analyzed by LCMS before it could be utilized in experiments, (37.7 mg, 0.078 mmol, 32% yield). Calculated mass for **84** [M+H] = 484.18, found in LCMS = 484.25.

Synthesis of compound (85).

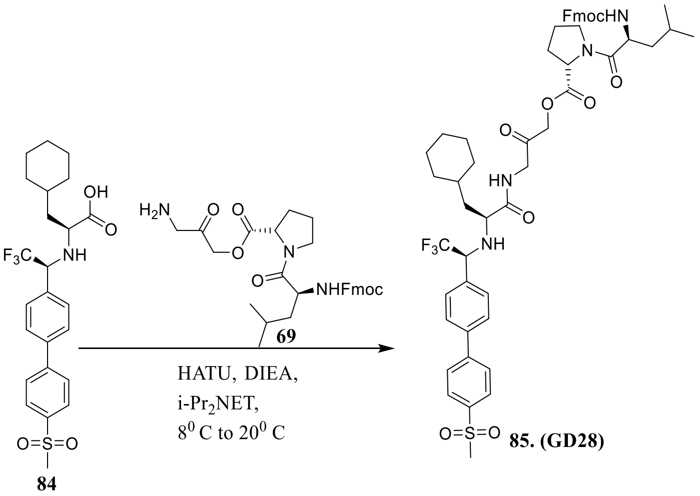

Compound **84** (1 equivalent, 10 mg, 0.0206 mmol) was put in ice-cooled dimethyl acetamide in a round-bottom flask. Compound **69** (1.3 equivalents) and HATU (4 equivalents) were added to the solution. The procedure was followed as described before (for compound **83**). The compound was purified by HPLC using a water/acetonitrile gradient. The purified compound **(85)** was analyzed by LCMS and freeze-dried before it was used (4 mg, 0.00405 mmol, 19.6% yield). Calculated mass for **GD28** [M+H] = 987.42, found in LCMS = 987.42.

Synthesis of compound (86).

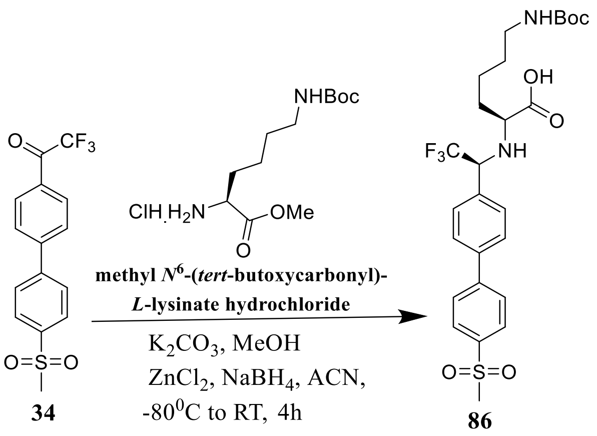

Anhydrous zinc chloride (2 equivalents) in dry DME was added to sodium borohydride (4 equivalents) at 0 °C and stirred at room temperature for 18 hours in a nitrogen environment. In the meanwhile, compound **34** (1 equivalent, 80 mg, 0.243 mmol) was heated at 55°C for 12 hours in a nitrogen environment with methyl ester salt of L-Lysine (1.25 equivalents), potassium carbonate (3 equivalents), and dry methanol. The procedure was followed as mentioned before (for compound **9**). The compound was purified by HPLC using a water/acetonitrile gradient. The compound was analyzed by LCMS before it was used in experiments (24 mg, 0.0429 mmol, 17.6% yield). Calculated mass for **86** [M] = 558.2, found in LCMS = 559.

Synthesis of compound (87).

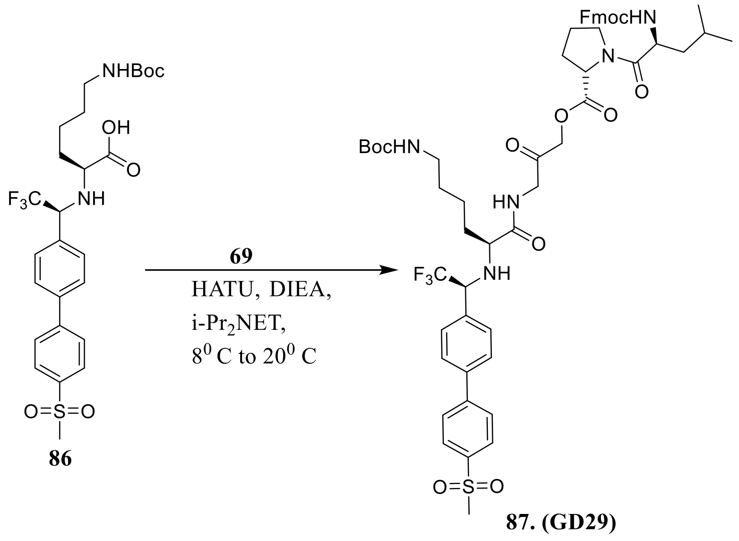

Compound **86** (1 equivalent, 12 mg, 0.02145 mmol) was put in ice-cooled dimethyl acetamide in a round-bottom flask. Compound **69** (3 equivalents) and HATU (4 equivalents) were added to the solution. The procedure was followed as described before (for compound **83**). The compound was purified by HPLC using a water/acetonitrile gradient. The purified compound **(87)** was analyzed by LCMS and freeze-dried before further use (4 mg, 0.00376 mmol, 17.52% yield). Calculated mass for **GD29** [M] = 1061.44, found in LCMS = 1062.25.

Synthesis of compound (89).

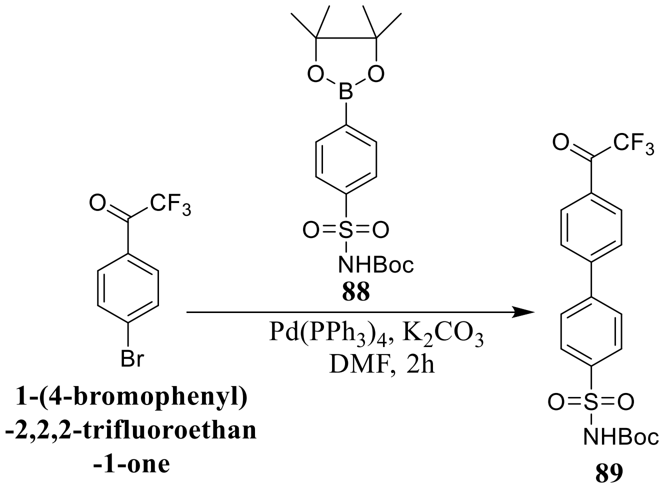

Commercially available compound **1-(4-bromophenyl)-2,2,2-trifluoroethan-1-one** (1.25 equivalent) and compound **88** (1 equivalent, 200 mg, 0.521 mmol) were put in a round-bottom flask under nitrogen followed by the addition of dry DMF, 5 equivalents of palladium tetrakis and 2 M aqueous solution of potassium carbonate. The procedure was followed as described before (for compound **34**). The compound was purified using column chromatography with hexane–ethyl acetate (160 mg, 0.372 mmol, 71% yield). The purity of compound **89** was analyzed by NMR.

Synthesis of compound (90).

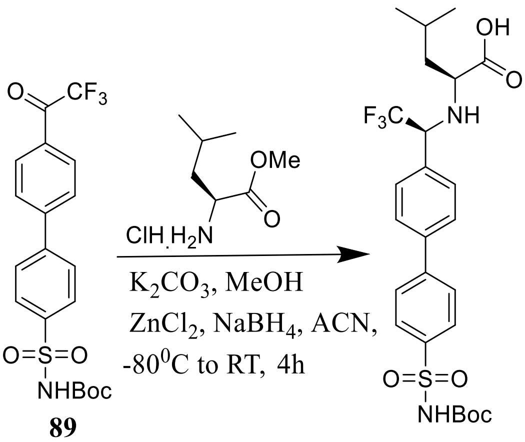

89 **90**

Anhydrous zinc chloride (2 equivalents) in dry dimethoxyethane was added to sodium borohydride (4 equivalents) at 0 °C and stirred at room temperature for 18 hours in a nitrogen environment. In the meanwhile, compound **89** (1 equivalent, 70 mg, 0.163 mmol) was heated to 55°C for 12 hours in a nitrogen environment with methyl ester salt of L-Leucine (1.25 equivalents), potassium carbonate (3 equivalents), and dry methanol. The procedure was followed as mentioned before (for compound **9**). The compound was purified by HPLC using a water/acetonitrile gradient. The compound was analyzed by LCMS before it was used further, (22 mg, 0.04 mmol, 24.7% yield). Calculated mass for **90** [M+H] = 545.19, found in LCMS = 545.17.

Synthesis of compound (91).

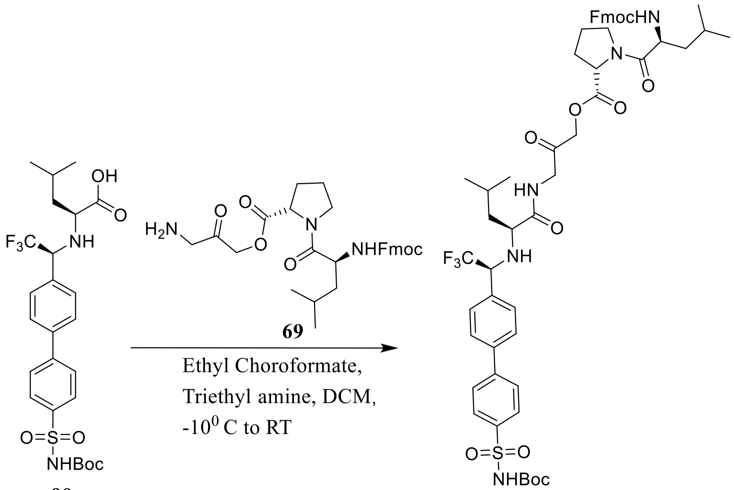

90

91. (GD31)

Compound **90** (1 equivalent, 20 mg, 0.0367 mmol) was put in ice-cooled dry DCM in round-bottom flask, ethyl chloroformate (2 equivalents) with triethyl amine (2 equivalents) were added to the reaction mixture. The reaction mixture was allowed to stir at 0° C for 15 mins. Compound **69** (1.3 equivalents) in the requisite amount of DCM was added inertly with some more equivalents of triethyl amine. The ice bath was removed, and the temperature of the reaction was allowed to rise to 6° C. The Reaction was quenched by adding water, and the DCM layer was collected. The organic solvent was removed and product was purified by a preparative reverse phase HPLC using a water/acetonitrile gradient. The purified compound **(91)** was analyzed by LCMS and freeze-dried (7 mg, 0.00668 mmol, 18.17% yield). Calculated mass for **GD31** [M] = 1047.42, found in LCMS = 1048.25.

Synthesis of compound (93).

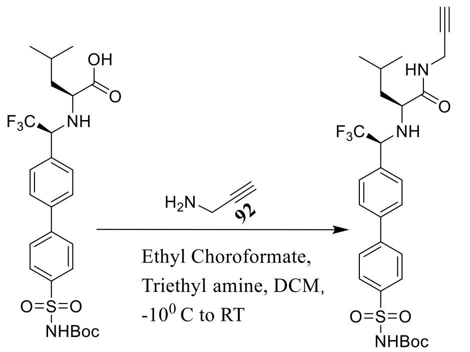

90

93. (GD33)

Compound **90** (1 equivalent, 20 mg, 0.0367 mmol) was put in ice-cooled dry DCM in a round-bottom flask. Ethyl chloroformate (2 equivalents) and tri ethyl amine (3 equivalents) were added. The reaction mixture was allowed to stir at 0° C for 15 mins. Compound **92** (3 equivalents) in DCM was added inertly with some more equivalents of triethyl amine. The ice bath was removed, and the temperature of the reaction was allowed to rise up to 6° C. The reaction was quenched by adding water, and the DCM layer was collected. The organic solvent was removed and product was purified by a preparative reverse phase HPLC using a water/acetonitrile gradient. The purified compound **(93)** was finally freeze-dried and analyzed by LCMS (3.2 mg, 0.0055 mmol, 15% yield). Calculated mass for **GD33** [M+H] = 582.22, found in LCMS = 582.25.

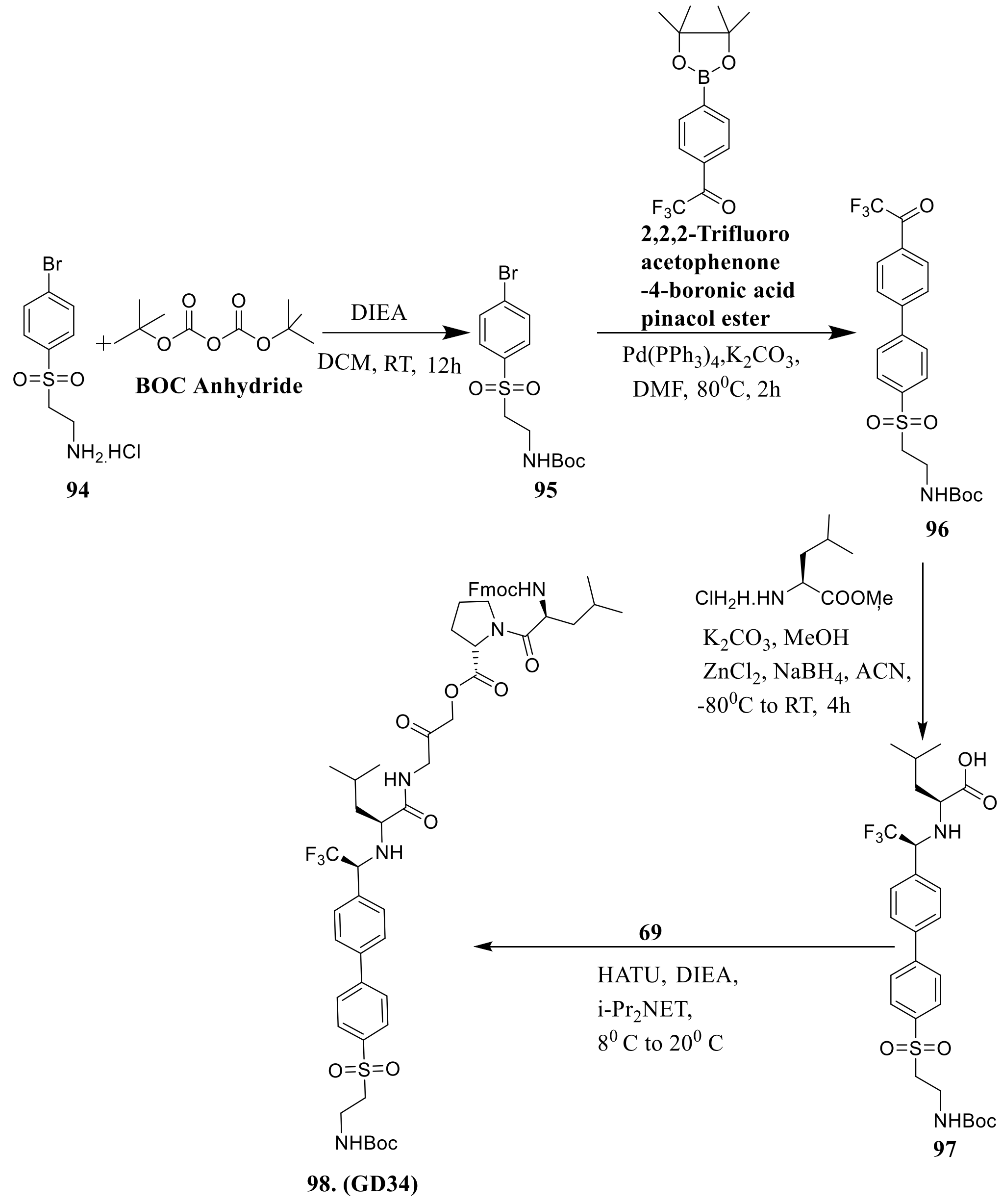

Scheme S12. Synthesis scheme of compound **GD34**.

Synthesis of compound (94).

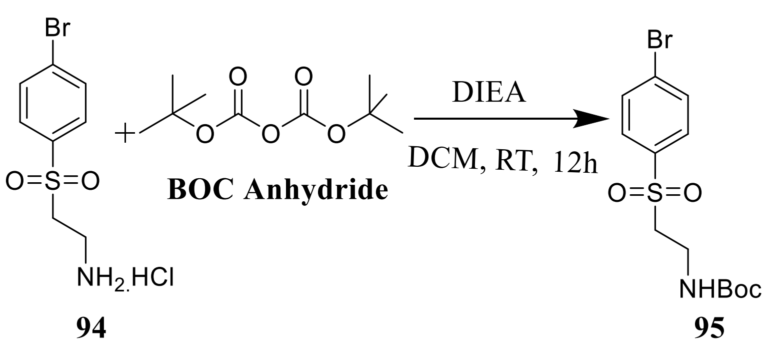

Commercially available compound **94** (1 equivalent, 200 mg, 0.665 mmol) and BOC Anhydride (6 equivalents) were put in a round-bottom flask in a nitrogen atmosphere, followed by the addition of 5 equivalent of DIEA and dry DCM. The reaction mixture was allowed to stir at room temperature for 12 hours, and the progress of the reaction was monitored by TLC. Once the reaction was complete, the reaction mixture was washed with 1N HCL solution, followed by separation of the organic layer that was dried over sodium sulfate before concentrating by a rotavap. Compound **(95)** was purified by column chromatography using a hexane – ethyl acetate system (216 mg, 0.59 mmol, 89.3% yield). Calculated mass for **95** [M + Na] = 386, found in MALDI = 386.63.

Synthesis of compound (95).

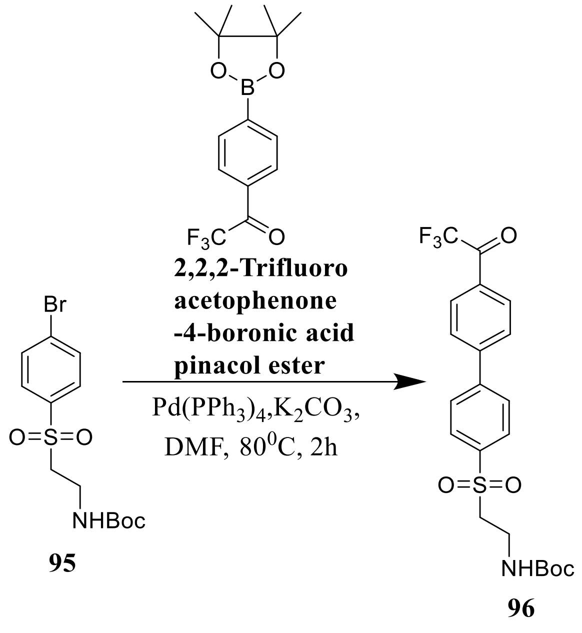

Commercially available compound **2,2,2-Trifluoro acetophenone-4-boronic acid pinacol ester** (1.25 equivalents) and compound **95** (1 equivalent, 206 mg, 0.565 mmol) were put in a round-bottom flask in a nitrogen atmosphere followed by the addition of dry DMF, 5 equivalent of palladium tetrakis and 2 M aqueous solution of potassium carbonate. The procedure was followed as described before (for compound **34**). The compound was purified through column

chromatography using a hexane–ethyl acetate (130 mg, 0.283 mmol, 50% yield). The compound was analyzed by NMR. Calculated mass for **96** [M + Na] = 480.11, found by MALDI = 480.23.

Synthesis of compound (97).

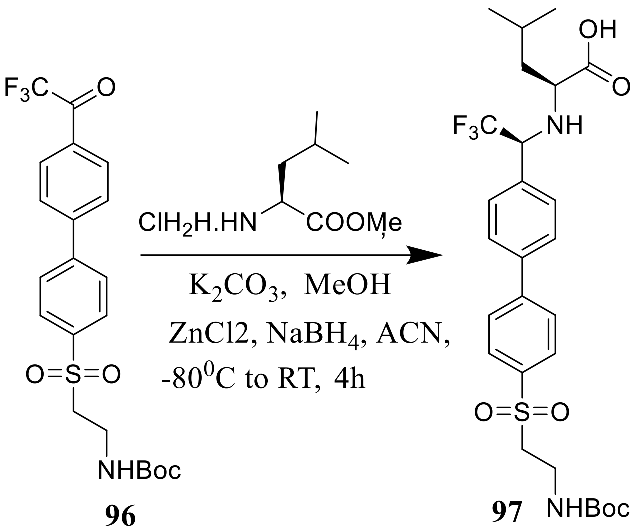

96 **97**

Anhydrous zinc chloride (2 equivalents) in dry DME was added to sodium borohydride (4 equivalents) at 0 °C and stirred at room temperature for 18 hours in a nitrogen environment. In the meanwhile, compound **96** (1 equivalent, 100 mg, 0.218 mmol)) was heated to 55°C for 12 hours in a nitrogen environment with methyl ester salt of L-leucine or L-fluoro leucine (1.25 equivalents), potassium carbonate (3 equivalents), and dry methanol. The procedure was followed as mentioned before (for compound **9**). The compound was purified by HPLC using a water/acetonitrile gradient. The compound was analyzed by LCMS and NMR before use (50 mg, 0.087 mmol, 40% yield). Calculated mass for **97** [M+H] = 572.22, found in LCMS = 572.83.

Synthesis of compound (98).

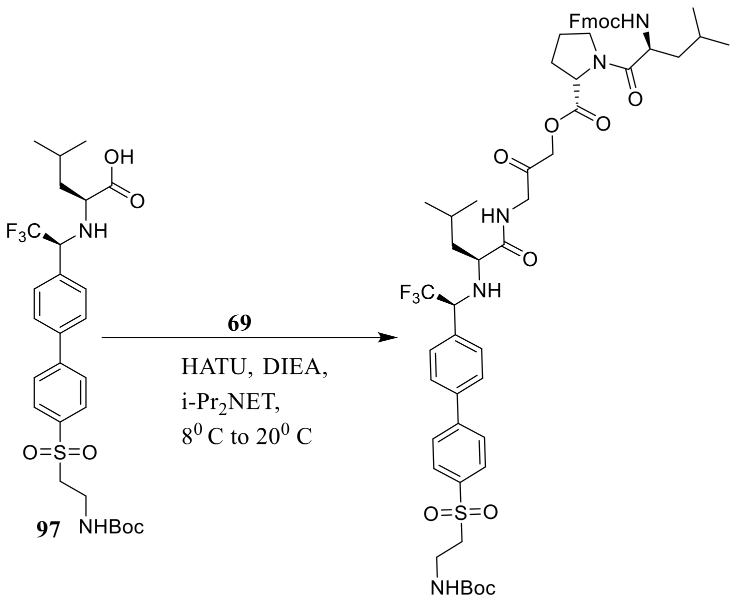

98. (GD34)

Compound **97** (1 equivalent, 40 mg, 0.07 mmol)) was put in ice-cooled dimethyl acetamide in a round-bottom flask. Compound **69** (3 equivalents) and HATU (4 equivalents) were added to the solution. The procedure was followed as described before (for compound **83**). The compound was purified by HPLC using a water/acetonitrile gradient. The purified compound **(98)** was analyzed by LCMS and freeze-dried before further use (14.5 mg, 0.01347 mmol, 19.2% yield). Calculated mass for **GD34** [M] = 1075.46, found in LCMS = 1076.25.

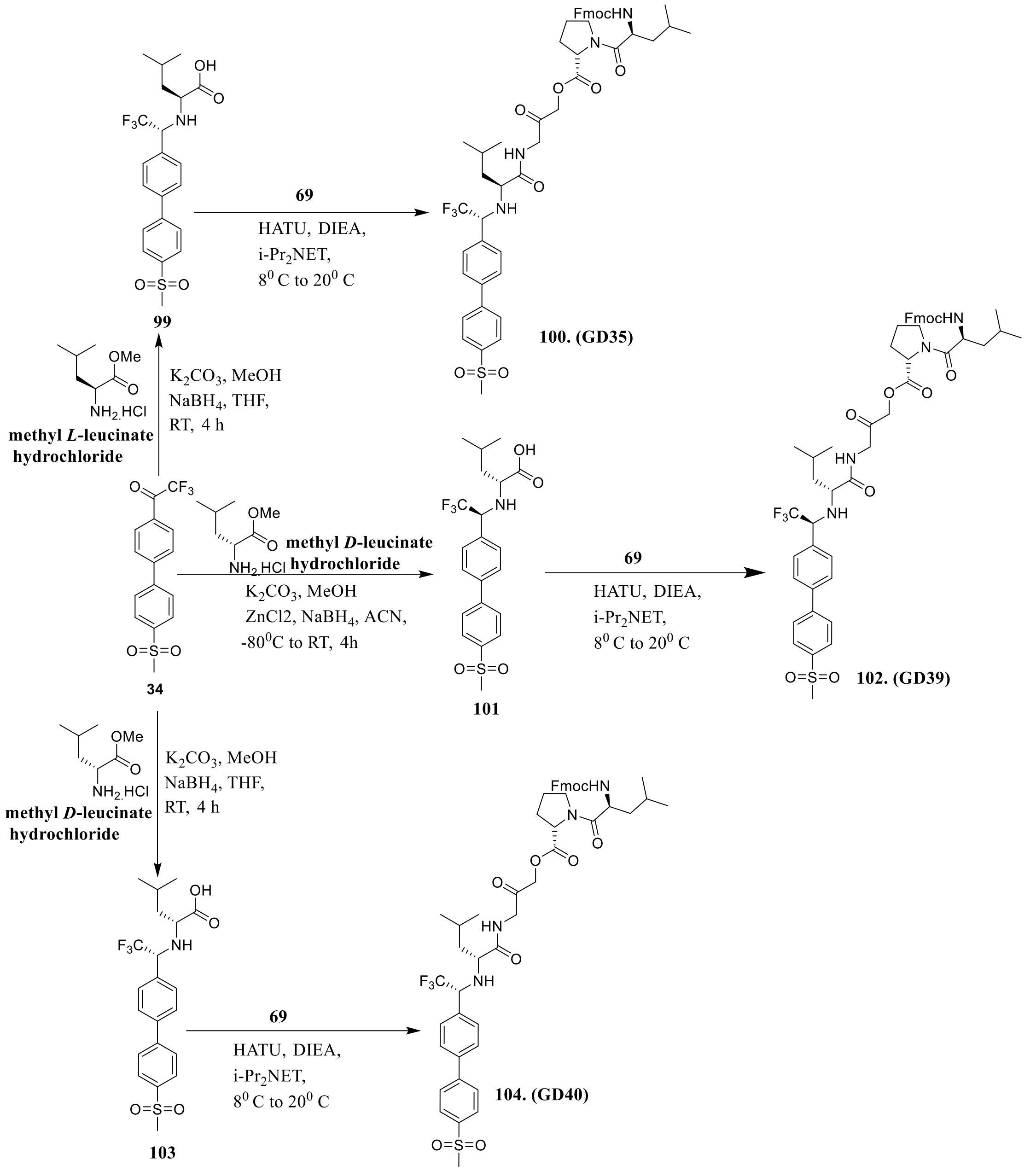

Scheme S13. Synthesis scheme of compound **GD35, GD39, and GD40**.

Synthesis of compound (99).

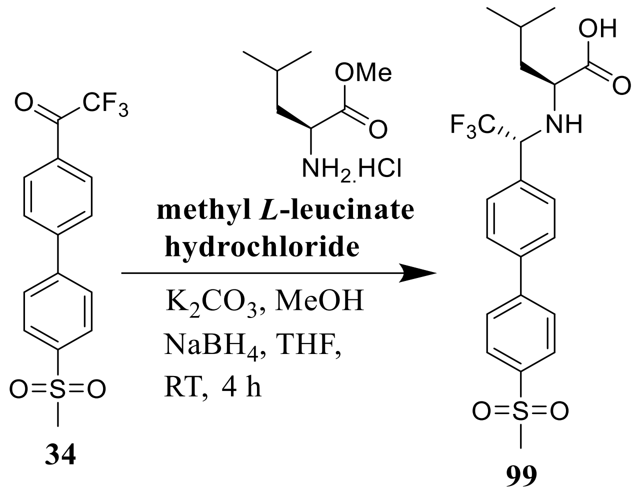

((*R*)-2,2,2-trifluoro-1-(4'-(methylsulfonyl)-[1,1'-biphenyl]-4-yl)ethyl)-*L*-leucine

At first, compound **34** (1 equivalent, 60 mg, 0.182 mmol) was heated to 55° C for 12 hours in a nitrogen environment with methyl ester salt of L-leucine (1.25 equivalents), potassium carbonate (3 equivalents), and dry methanol. The methanol was then removed using a rotavap after the reaction mixture had been allowed to reach room temperature. While at room temperature, a subsequent amount of THF was gradually added to the mixture and transferred to a round-bottom flask containing the solution of sodium borohydride (4 equivalent) in THF. At this temperature, the resultant reaction mixture was left to stir for 4 hours. During this time, a 20% (v/v) solution of water/THF was added dropwise. After adding 1(N) HCl, the solvent was evaporated by a rotavap. The reaction mixture was mixed with tertiary butyl methyl ether; then, water was added for workup. The ether layer was collected and dried by a rotavap. The compound was purified by reverse-phase HPLC with a water/acetonitrile gradient (20.2 mg, 0.045 mmol, 24% yield). The compound was analyzed by LCMS and NMR. Calculated mass for **99** [M] = 443.14, found in LCMS = 443.92.

Synthesis of compound (100).

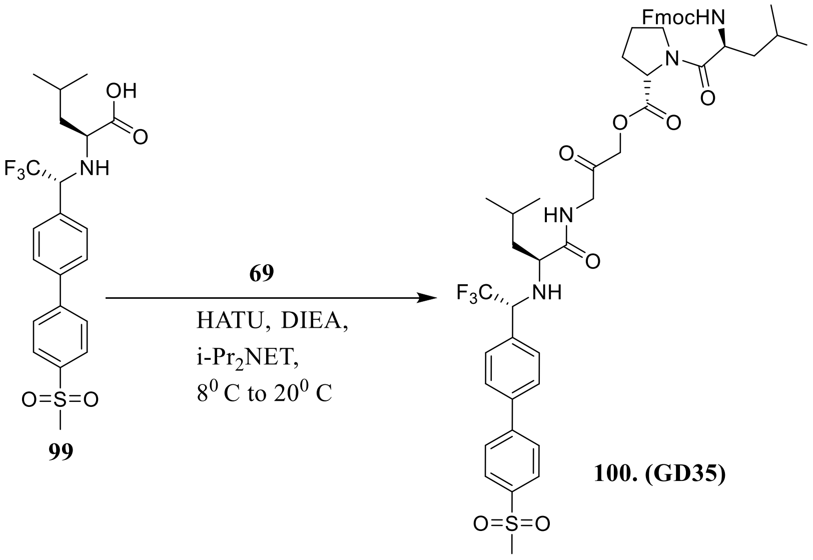

Compound **99** (1 equivalent, 20.2 mg, 0.045 mmol) was put in ice-cooled dimethyl acetamide in a round-bottom flask. Compound **69** (1.3 equivalents) and HATU (4 equivalents) were added to the solution. The procedure was followed as described before (for compound **83**). The compound was purified by HPLC using a water/acetonitrile gradient. The purified compound **(100)** was analyzed by LCMS and freeze-dried before further use (7 mg, 0.0074 mmol, 16.4% yield). The purity of the compounds was checked in analytical HPLC and mass was calculated in MALDI. Calculated mass for **GD35** [M + H] = 947.39, found in MALDI = 947.95.

Synthesis of compound (101).

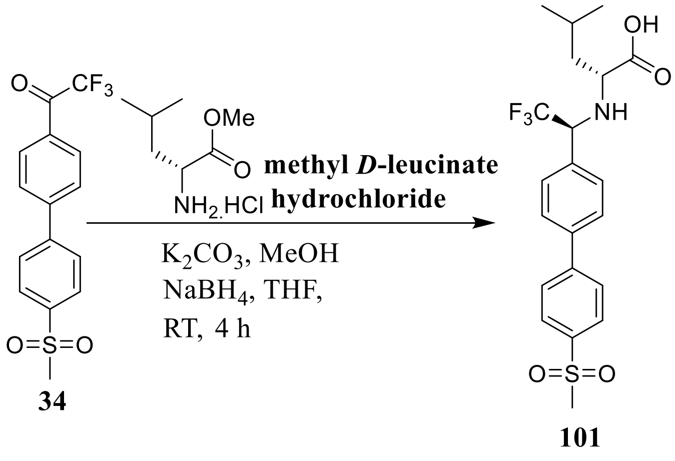

Compound **34** (1 equivalent, 60 mg, 0.182 mmol)) was heated to 55°C for 12 hours in a nitrogen environment with methyl ester salt of D-leucine (1.25 equivalents), potassium carbonate (3 equivalents), and dry methanol. The methanol was then removed using a rotavap after the reaction mixture was allowed to reach room temperature. The procedure was as mentioned before (for compound **99**). Reverse-phase HPLC purification with a water/acetonitrile gradient was used to purify the product (17 mg, 0.038 mmol, 21% yield). The compound was analyzed by LCMS, and NMR. Calculated mass for **101** [M] = 443.14, found in MALDI = 443.92.

Synthesis of compound (102).

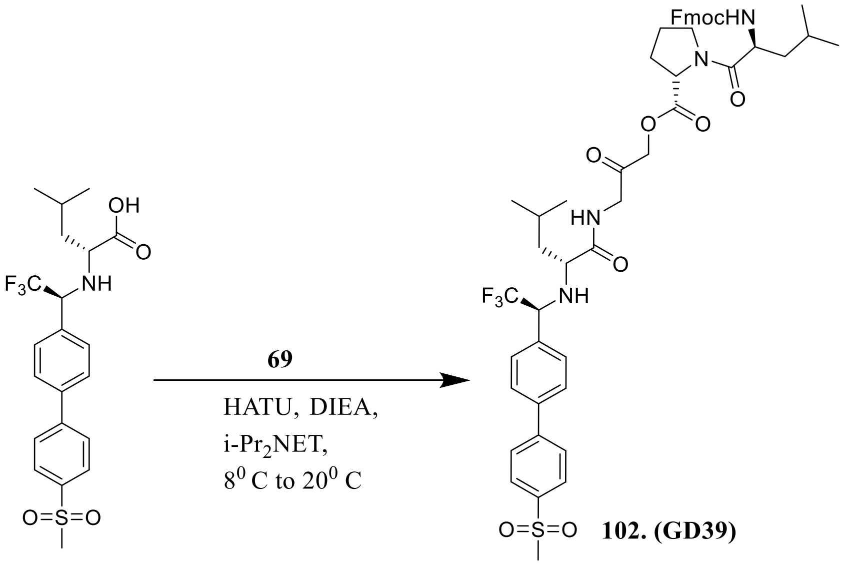

101

102. (GD39)

Compound **101** (1 equivalent, 17 mg, 0.038 mmol,) was put in ice-cooled dimethyl acetamide in a round-bottom flask. Compound **69** (1.3 equivalent) and HATU (4 equivalents) were added to the solution. The procedure was followed as described before (for compound **83**). The compound was purified by HPLC using a water/acetonitrile gradient. The purified compound **(102)** was analyzed by LCMS and freeze-dried before further use (6 mg, 0.006 mmol, 16.5% yield). Calculated mass for **GD39** [M + H] = 947.39, found in MALDI = 947.95.

Synthesis of compound (103).

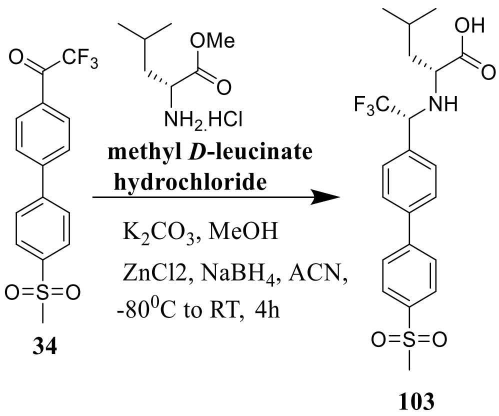

Anhydrous zinc chloride (2 equivalents) in dry DME was added to sodium borohydride (4 equivalents) at 0 °C, reaction was brought to room temperature and stirred for 18 hours in a nitrogen environment. In the meanwhile, compound **34** (1 equivalent, 60 mg, 0.182 mmol) was heated to 55°C for 12 hours in a nitrogen environment with methyl ester salt of D-leucine (1.25 equivalents), potassium carbonate (3 equivalents), and dry methanol. The procedure was followed as mentioned before (for compound **9**). The compound was purified by HPLC using a water/acetonitrile gradient and analysed by LCMS and NMR before further use, (28.33 mg, 0.0639 mmol, 35% yield). Calculated mass for **103** [M + H] = 443.14, found in LCMS = 443.92.

Synthesis of compound (104).

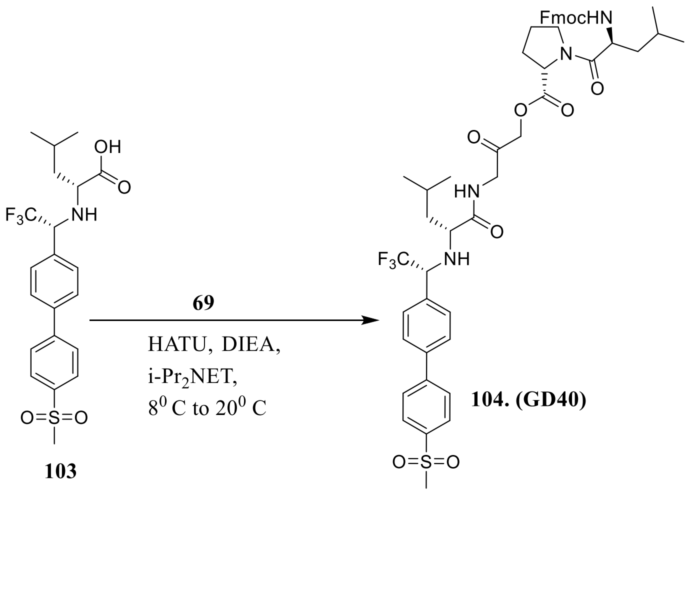

Compound **103** (1 equivalent, 28.33 mg, 0.0639 mmol) was put in ice-cooled dimethyl acetamide in a round-bottom flask. Compound **69** (3 equivalents) and HATU (4 equivalents) were added to the solution. The procedure was followed as described before (for compound **83**). The compound was purified by HPLC using a water/acetonitrile gradient. The purified compound **(104)** was analyzed by LCMS, freeze-dried before further use (6.6 mg, 0.007 mmol, 11% yield). Calculated mass for **GD40** [M+H] = 948.4, found in LCMS = 948.92.

These reactions were repeated multiple times using equal amounts of reactants to obtain the desired quantity of compounds. The overall yield has been determined by utilizing the mean value.

Spectroscopic details of the compound tert-butyl (4'-(2,2,2-trifluoroacetyl)-[1,1'-biphenyl]-4-yl)carbamate (**8**)**:**

^{1}H NMR (500 MHz, CDCl$_3$): δ 8.14 (d, *J* = 5 Hz, 2H),7.75 (d, *J* = 5 Hz, 2H), 7.61 (d, *J* = 10 Hz, 2H), 7.52 (d, *J* = 5 Hz, 2H), 6.65 (s, 1H), 1.57 (s, 9H).

^{13}C NMR (125 MHz, CDCl$_3$): δ 180.1, 152.5, 145.95, 147.5, 139.3, 133.4, 130.8, 127.9, 127, 118.8, 115.6, 81, 28.1.

Spectroscopic details of the compound. ((S)-1-(4'-((tert-butoxycarbonyl)amino)-[1,1'-biphenyl]-4-yl)-2,2,2-trifluoroethyl)-L-leucine **(9):**

^{1}H NMR (500 MHz, CDCl$_3$): δ 7.52 (d, *J* = 5 Hz, 2H),7.47 (d, *J* = 5 Hz, 2H), 7.37-7.43 (m, 4H), 4.08-4.12 (m, 1H), 3.58 (q, *J* = 5 Hz, 1H), 1.9-2 (m, 1H), 1.56 (s, 9H), 1.43-1.52 (m, 2H), 0.95 (t, *J* = 7.5 Hz, 6H).

^{13}C NMR (125 MHz, CDCl$_3$): δ 180.3, 141.3, 137.8, 135, 133.3, 128.7, 127.5, 127, 124.5, 119, 63.5, 58.8, 42.6, 28.3, 24.7, 22.9, 21.7.

Spectroscopic details of the compound. (S)-2-(((S)-1-(4'-((tert-butoxycarbonyl)amino)-[1,1'-biphenyl]-4-yl)-2,2,2-trifluoroethyl)amino)-4-fluoro-4-methylpentanoic acid (**10**):

^{1}H NMR (500 MHz, CDCl$_3$): δ 7.53 (d, J = 10 Hz, 2H), 7.47 (d, J = 10 Hz, 2H), 7.26-7.43 (m, 4H), 5.64 (b, 2H), 4.19-4.24 (m, 1H), 3.68 (q, J = 5 Hz, 1H), 2.13-2.22 (m, 1H), 1.91-1.99 (m, 1H), 1.55 (s, 9H), 1.44 (dd, J = 10 Hz, 6H).

^{13}C NMR (125 MHz, CDCl$_3$): 177.3, 141.6, 138, 134.8, 132.2, 128, 127.5, 127.1, 124.2, 119.1, 94.7 (d, J = 40 Hz), 62.9, 56.8, 43.5 (d, J = 38.75 Hz), 28.37, 26.93 (d, J = 26.2 Hz).

Spectroscopic details of the compound. 2,2,2-trifluoro-1-(4'-(methylsulfonyl)-[1,1'-biphenyl]-4-yl)ethan-1-one (**34**):

^{1}H NMR (500 MHz, CD$_3$COCD$_3$): δ 8.03 (d, J = 10 Hz, 2H),7.91 (d, J = 10 Hz, 2H), 7.72 - 7.82 (m, 4H), 3.16 (s, 3H).

^{13}C NMR (125 MHz, CD$_3$COCD$_3$): δ 147.8, 142.2, 141.8, 137.2, 130.8, 129.8, 129.7, 128.8, 128.4, 117.5, 44.8.

Spectroscopic details of the compound. ((S)-2,2,2-trifluoro-1-(4'-(methylsulfonyl)-[1,1'-biphenyl]-4-yl)ethyl)-L-leucine **(35):**

^{1}H NMR (500 MHz, CDCl$_3$): δ 7.99 (d, J = 10 Hz, 2H), 7.74 (d, J = 5 Hz, 2H), 7.60 (d, J = 5 Hz, 2H), 7.51 (d, J = 10 Hz, 2H), 6.22 (s, 2H), 4.15 (q, J = 5 Hz, 1H), 3.57 (dd, J = 8.8, 5.2 Hz, 1H), 3.09 (s, 3H), 1.89 – 1.94 (m, 1H), 1.47 – 1.58 (m, 2H), 0.94 (dd, J = 9.2, 6.7 Hz, 6H).

^{13}C NMR (125 MHz, CDCl$_3$): 180.4, 145.9, 140.2, 139.6, 135.2, 129.3, 128.1, 127.9, 126.6, 124.4, 63, 58.6, 44.7, 42.7, 24.9, 23, 21.8.

Spectroscopic details of the compound. tert-butyl ((4'-(2,2,2-trifluoroacetyl)-[1,1'-biphenyl]-4-yl)sulfonyl)carbamate (**89**):

^{1}H NMR (500 MHz, CD_3COCD_3): δ 8.26 (d, *J* = 10 Hz, 2H), 8.18 (d, *J* = 10 Hz, 2H), 8.08 (d, J=7.5 Hz, 4H), 1.41 (s, 9H).

^{13}C NMR (125 MHz, CD_3COCD_3): 179.9, 150, 146.3, 144, 140, 131, 129, 128.6, 128.3, 127.1, 115.6, 82.9, 27.5, 24.7.

Spectroscopic details of the compound. ((S)-1-(4'-(N-(tert-butoxycarbonyl)sulfamoyl)-[1,1'-biphenyl]-4-yl)-2,2,2-trifluoroethyl)-L-leucine **(90):**

^{1}H NMR (500 MHz, $CDCl_3$): δ 8.05 (d, *J* = 5 Hz, 2H), 7.71 (d, *J* = 10 Hz, 2H), 7.61 (d, *J* = 10 Hz, 2H), 7.52 (d, *J* = 10 Hz, 2H), 5.49 (s, 3H), 4.17-4.21 (m, 1H), 3.58 (q, *J* = 5 Hz, 1H), 1.9 – 1.96 (m, 1H), 1.48 – 1.61 (m, 2H), 1.39 (s, 9H), 0.94 (dd, *J* = 7.5 Hz, 6H).

^{13}C NMR (125 MHz, $CDCl_3$): 179.4, 149.5, 145.6, 139.9, 137.8, 134.9, 129.1, 128.8, 127.7, 127.4, 126.4, 84.6, 63.05 (d, *J* = 28.75 Hz), 58.4, 42.5, 27.8, 24.7, 22.8, 21.7.

Spectroscopic details of the compound. ((R)-2,2,2-trifluoro-1-(4'-(methylsulfonyl)-[1,1'-biphenyl]-4-yl)ethyl)-L-leucine **(99):**

^{1}H NMR (500 MHz, $CDCl_3$): δ 8.02 (d, *J* = 10 Hz, 2H), 7.78 (d, *J* = 5 Hz, 2H), 7.64 (d, *J* = 10 Hz, 2H), 7.55 (d, *J* = 10 Hz, 2H), 4.27 (q, *J* = 10 Hz, 1H), 3.14 (dd, *J* = 7.5 Hz, 1H), 3.11 (s, 3H), 1.83 – 1.91 (m, 1H), 1.54 (t, *J* = 5 Hz, 2H), 0.91 (d, *J* = 10 Hz, 3H), 0.75 (d, *J* = 5 Hz, 3H).

^{13}C NMR (125 MHz, $CDCl_3$): 176.7, 145.9, 140, 137.9, 134.3. 129.2, 128.5, 128.1, 127.9, 124.1 (q, *J* = 258.7 Hz), 94.7 (q, *J* = 163.7 Hz), 62.3 (q, *J* = 28.75 Hz), 56 (d, *J* = 65 Hz), 43.8, 34.6, 28.3, 27.1 (d, *J* = 25 Hz), 26.8 (d, *J* = 25 Hz).

Spectroscopic details of the compound. ((S)-2,2,2-trifluoro-1-(4'-(methylsulfonyl)-[1,1'-biphenyl]-4-yl)ethyl)-D-leucine **(101):**

^{1}H NMR (500 MHz, $CDCl_3$): δ 8.02 (d, *J* = 5 Hz, 2H), 7.77 (d, *J* = 10 Hz, 2H), 7.63 (d, *J* = 10 Hz, 2H), 7.56 (d, *J* = 10 Hz, 2H), 5.87 (s, 2H), 4.3 – 4.34 (m, 1H), 3.13 (t, *J* = 7.5 Hz, 1H),

3.11 (s, 3H), 1.85 – 1.95 (m, 1H), 1.53 (t, *J* = 7.5 Hz, 2H), 0.91 (d, *J* = 5 Hz, 3H), 0.75 (d, *J* = 10 Hz, 3H).

^{13}C NMR (125 MHz, $CDCl_3$): 181.1, 145.5, 140.2, 139.3, 133.2, 129.6, 128, 127.9, 127.7, 123.6, 99.9, 62.7 (q, *J* = 110 Hz), 56.2, 44.7, 42.7, 24.2, 23.1, 21.

Spectroscopic details of the compound. ((R)-2,2,2-trifluoro-1-(4'-(methylsulfonyl)-[1,1'-biphenyl]-4-yl)ethyl)-D-leucine (**103**):

^{1}H NMR (500 MHz, $CDCl_3$): δ 8 (d, *J* = 10 Hz, 2H), 7.74 (d, *J* = 10 Hz, 2H), 7.60 (d, *J* = 10 Hz, 2H), 7.51 (d, *J* = 10 Hz, 2H), 5.73 (s, 2H), 4.16 – 4.2 (m, 1H), 3.57 – 3.6 (q, *J* = 5 Hz, 1H), 3 (s, 3H), 1.90 – 1.95 (m, 1H), 1.46 – 1.6 (m, 2H), 0.93 (dd, *J* = 10 Hz, 6H).

^{13}C NMR (125 MHz, $CDCl_3$): 180, 145.7, 140, 139.4, 135, 129.8, 129.1, 128, 127.8, 122 (q, *J* = 280 Hz), 62.6 (q, *J* = 30 Hz), 58.4, 44.5, 42.5, 24.7, 22.8, 21.7.

NMR spectra

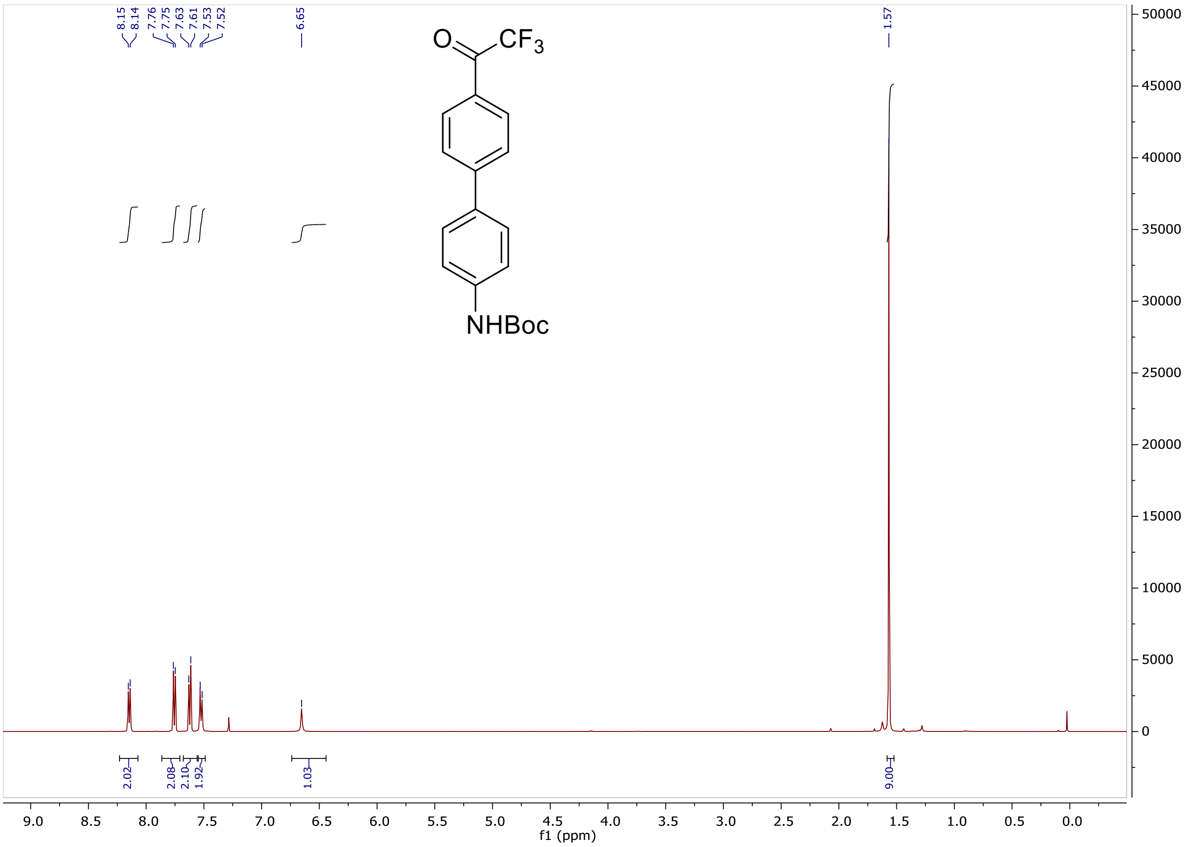

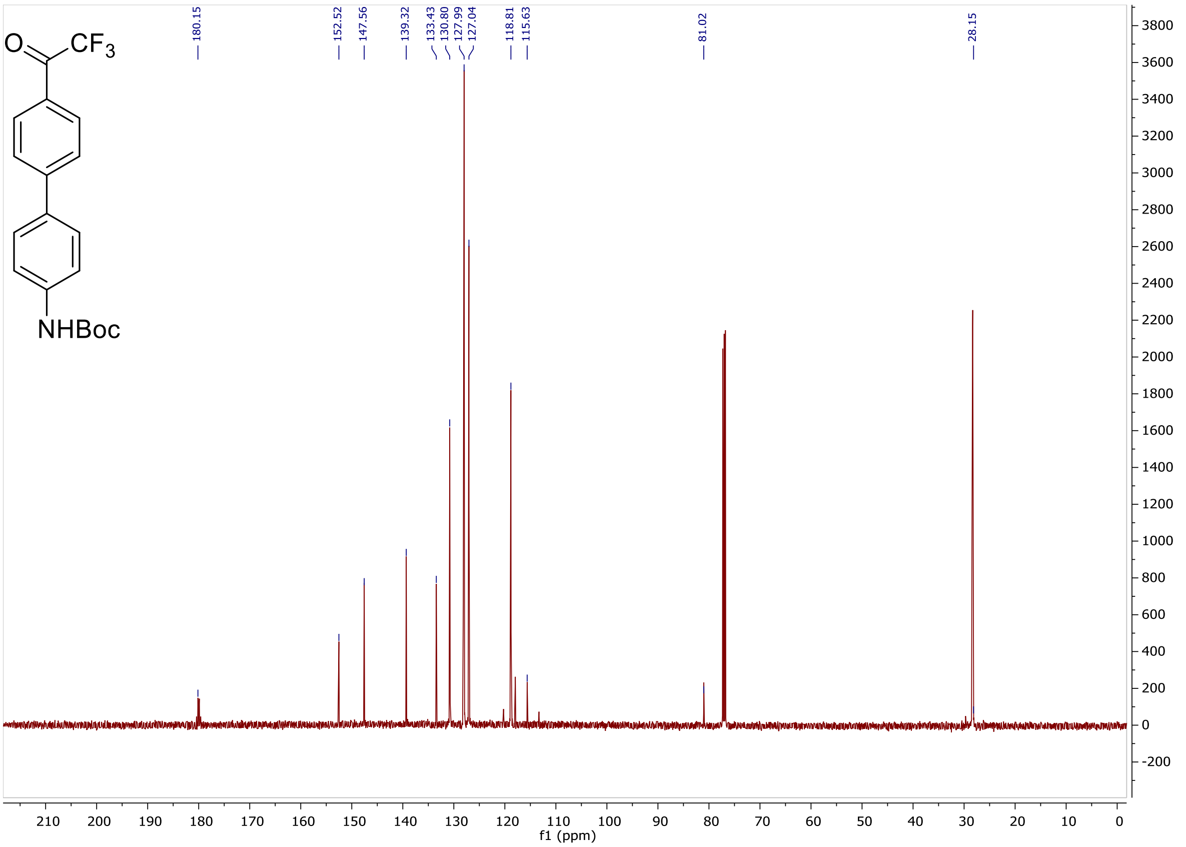

Figure S14. ^{1}H and ^{13}C NMR of Compound **8**.

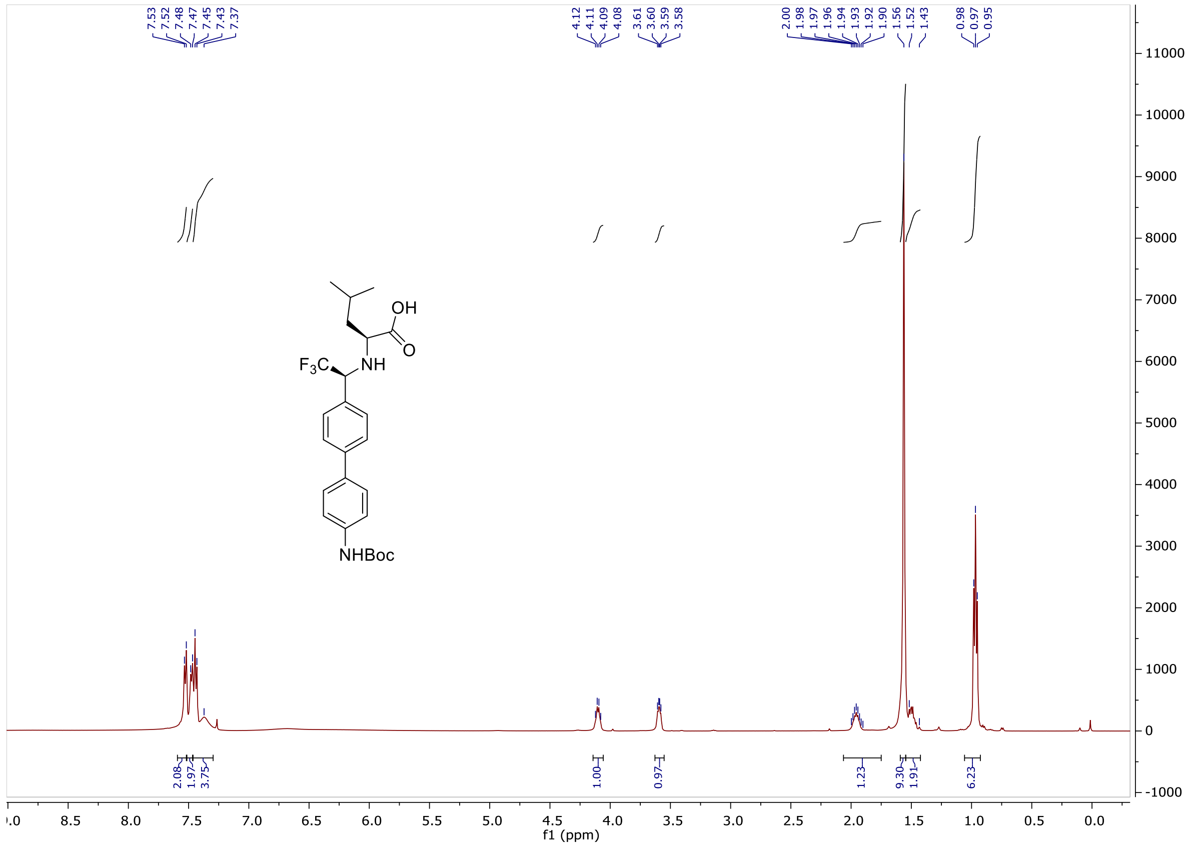

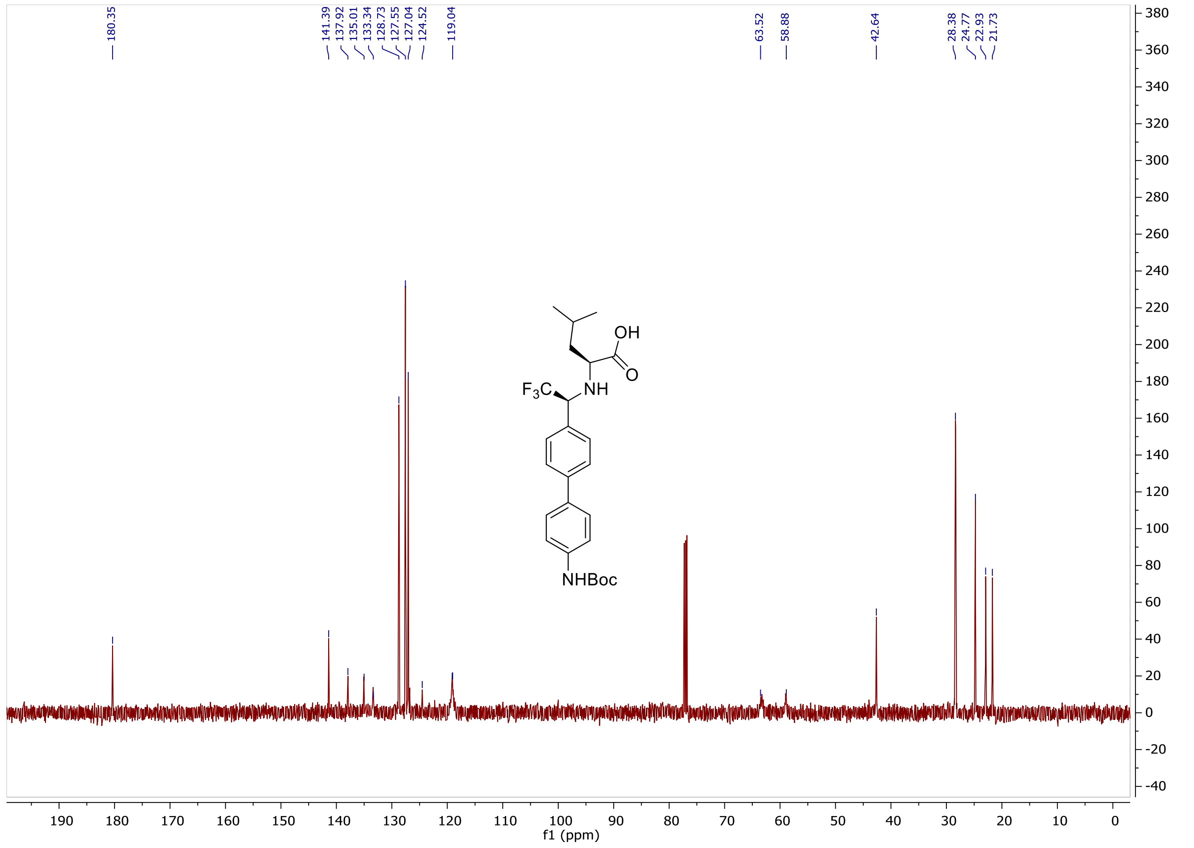

Figure S15. ^{1}H and ^{13}C NMR of Compound **9**.

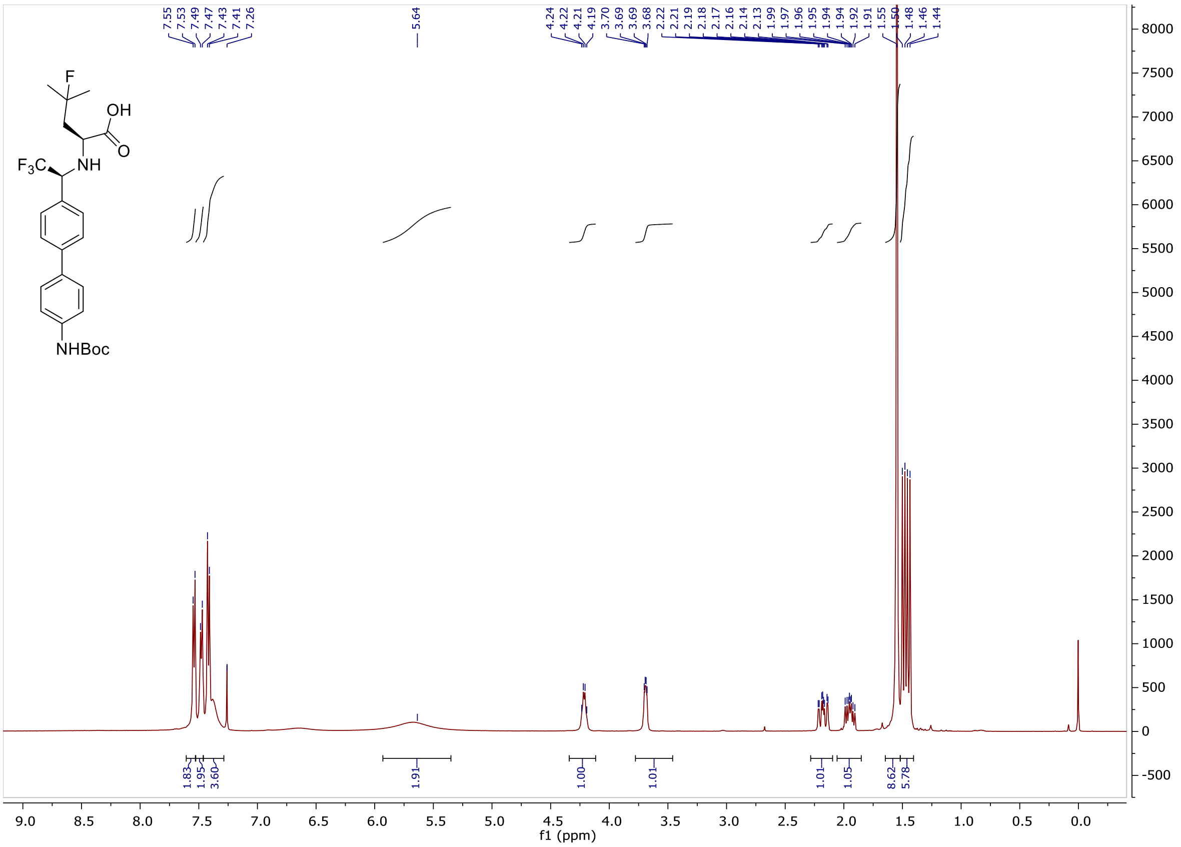

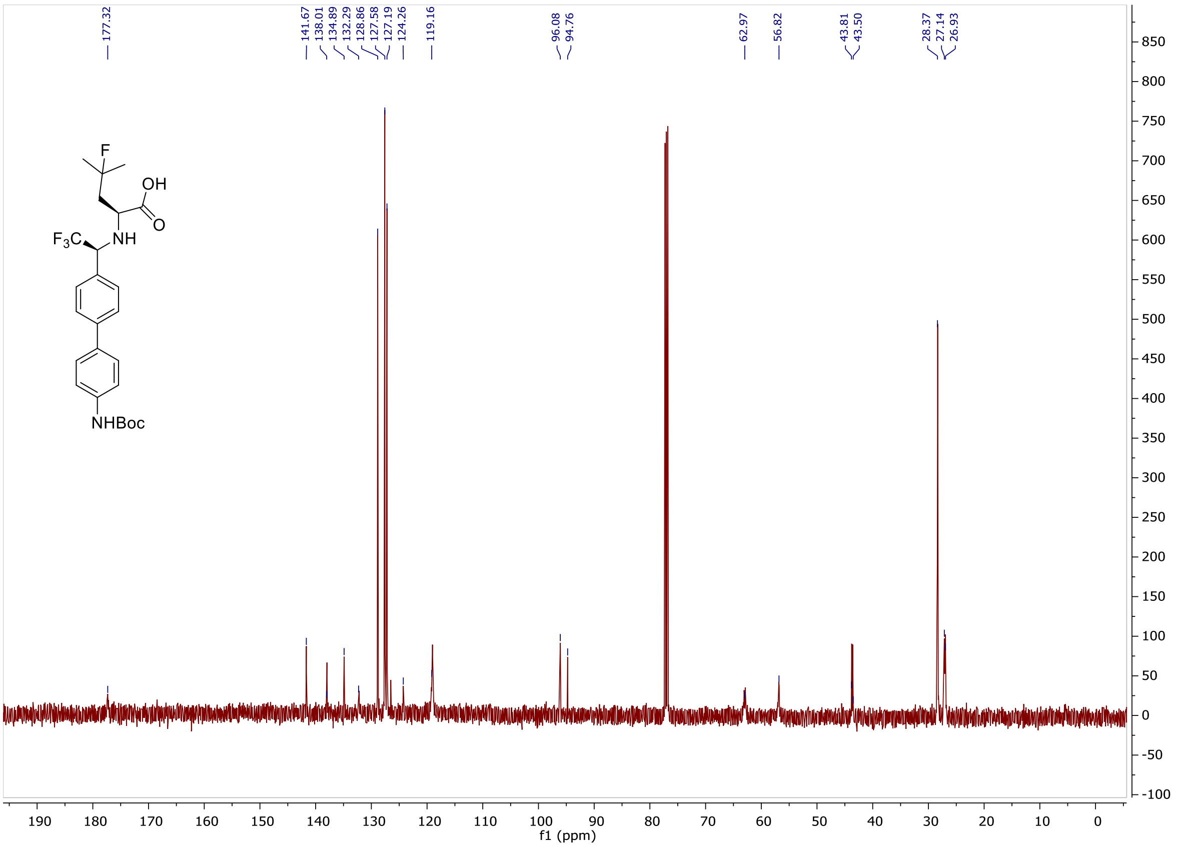

Figure S16. ^{1}H and ^{13}C NMR of Compound **10**.

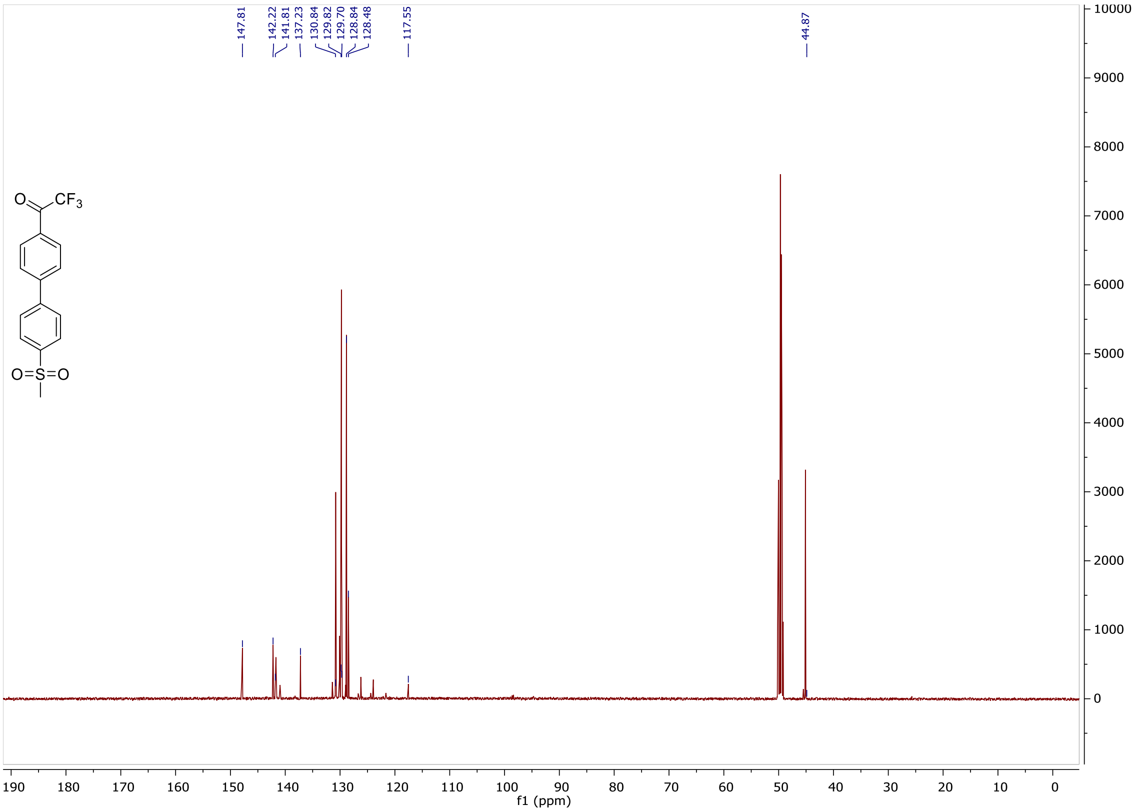

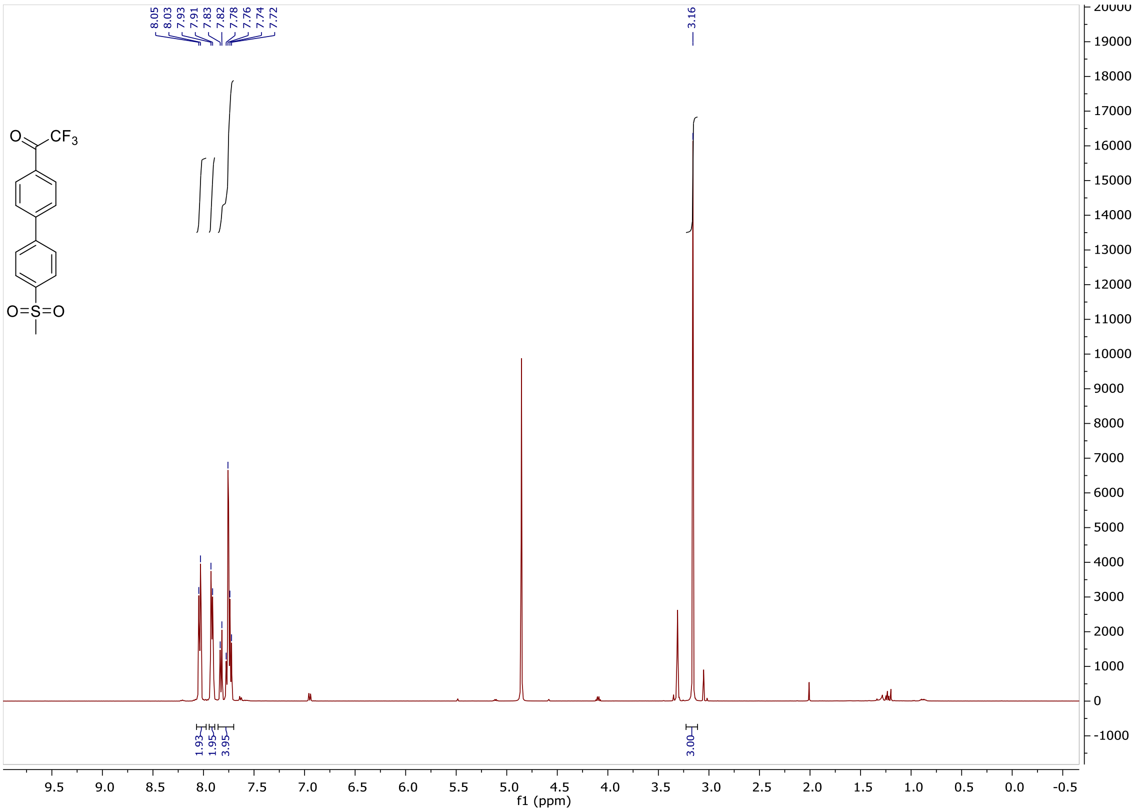

Figure S17. ^{1}H and ^{13}C NMR of Compound **34**.

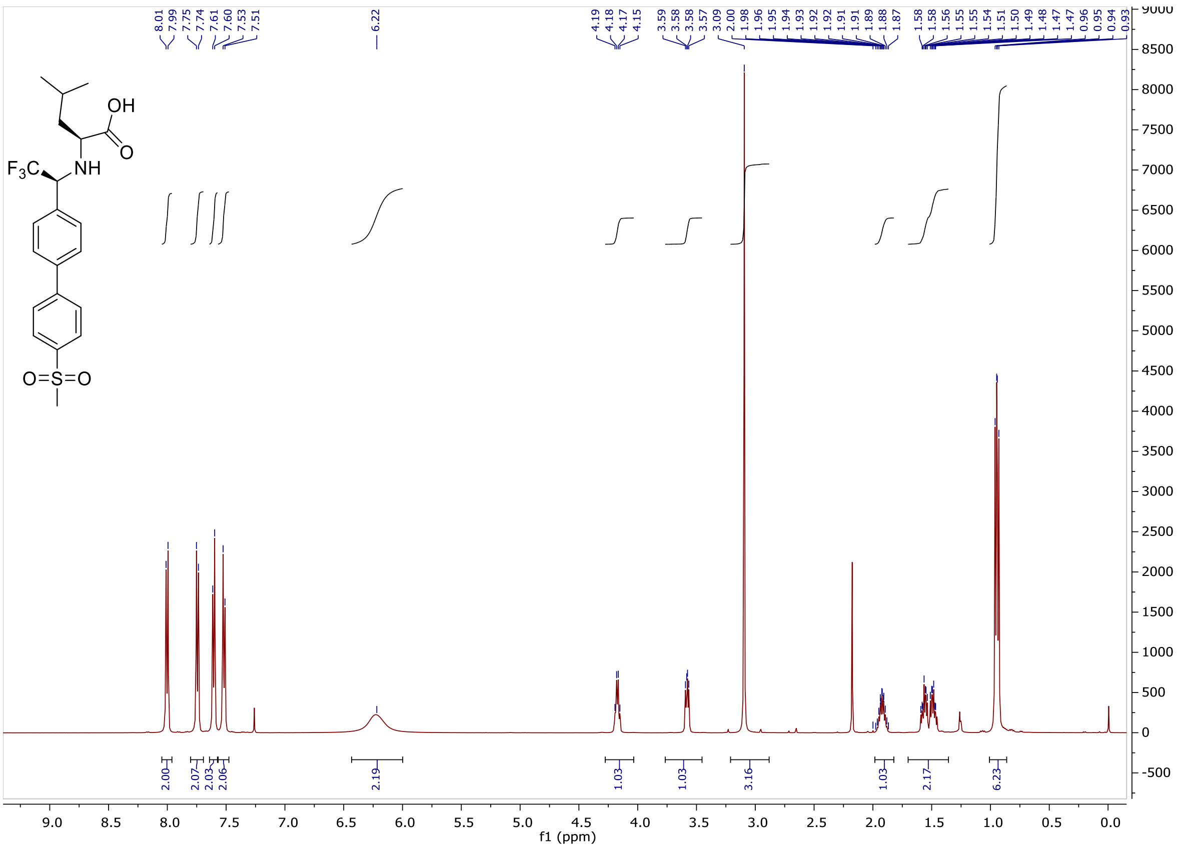

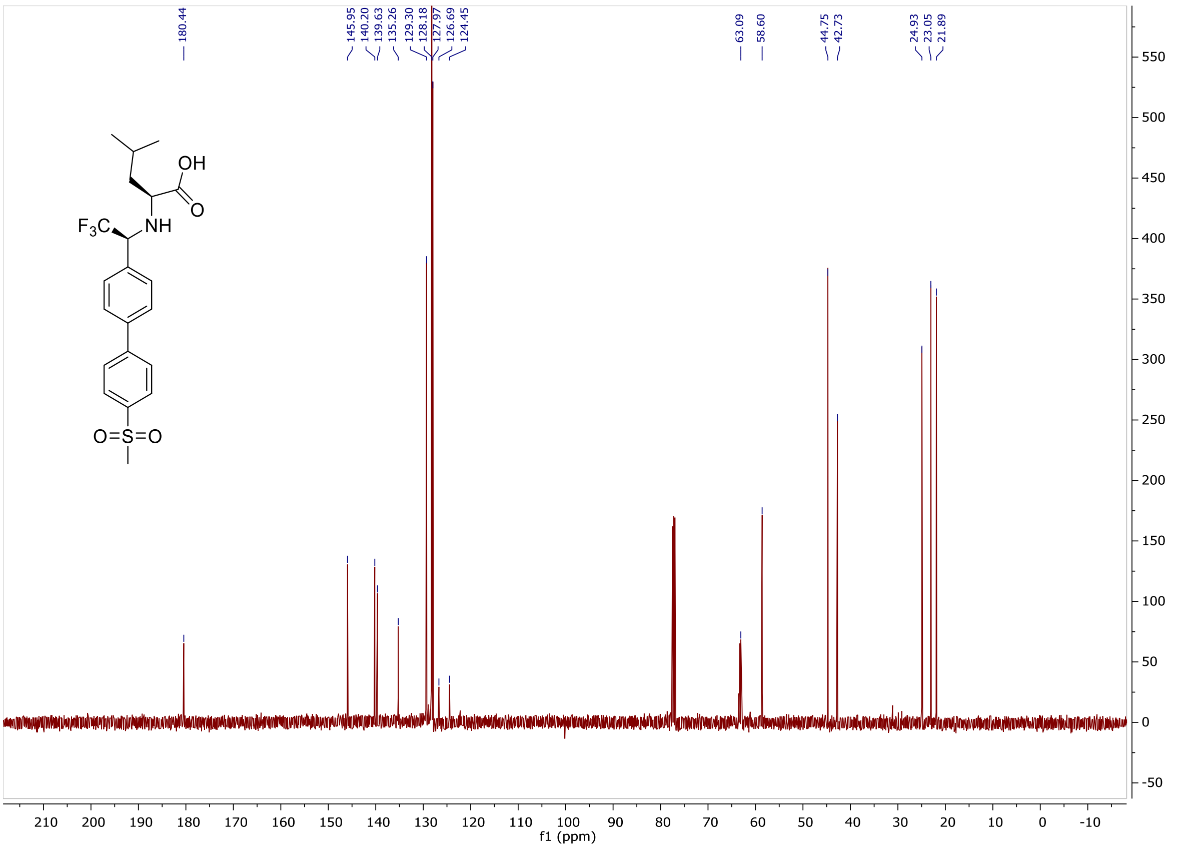

Figure S18. ^{1}H and ^{13}C NMR of Compound **35**.

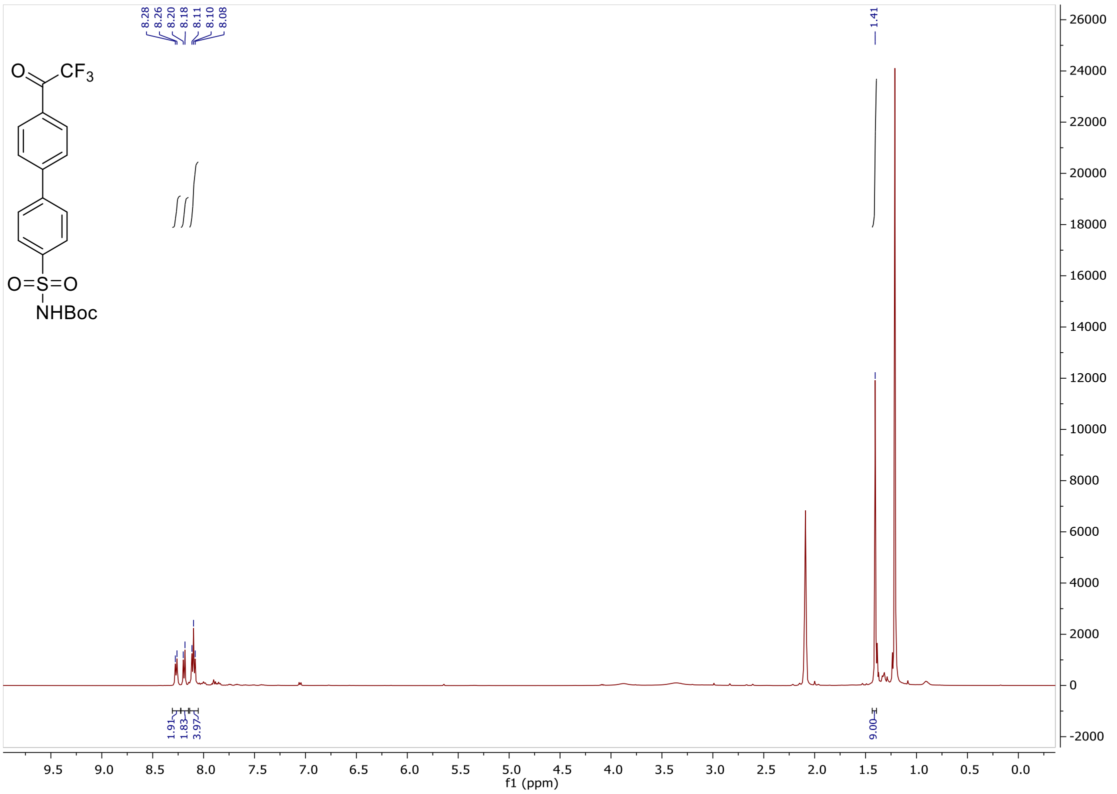

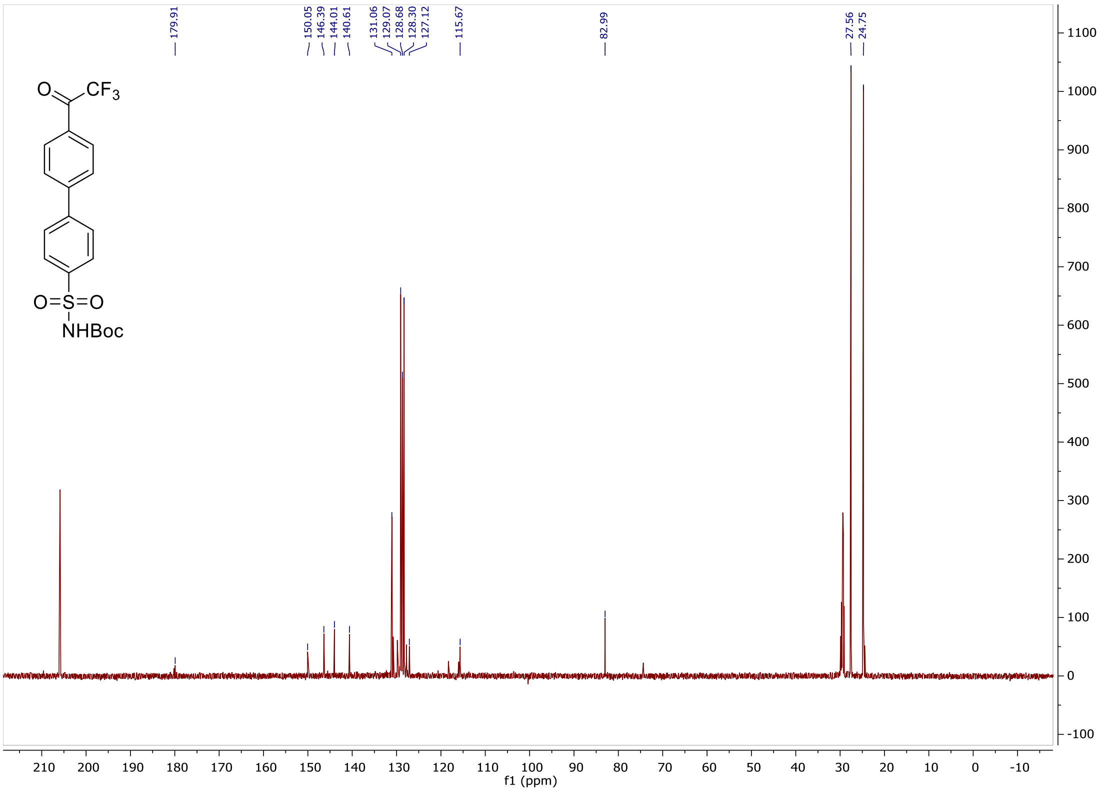

Figure S19. ^{1}H and ^{13}C NMR of Compound **89**.

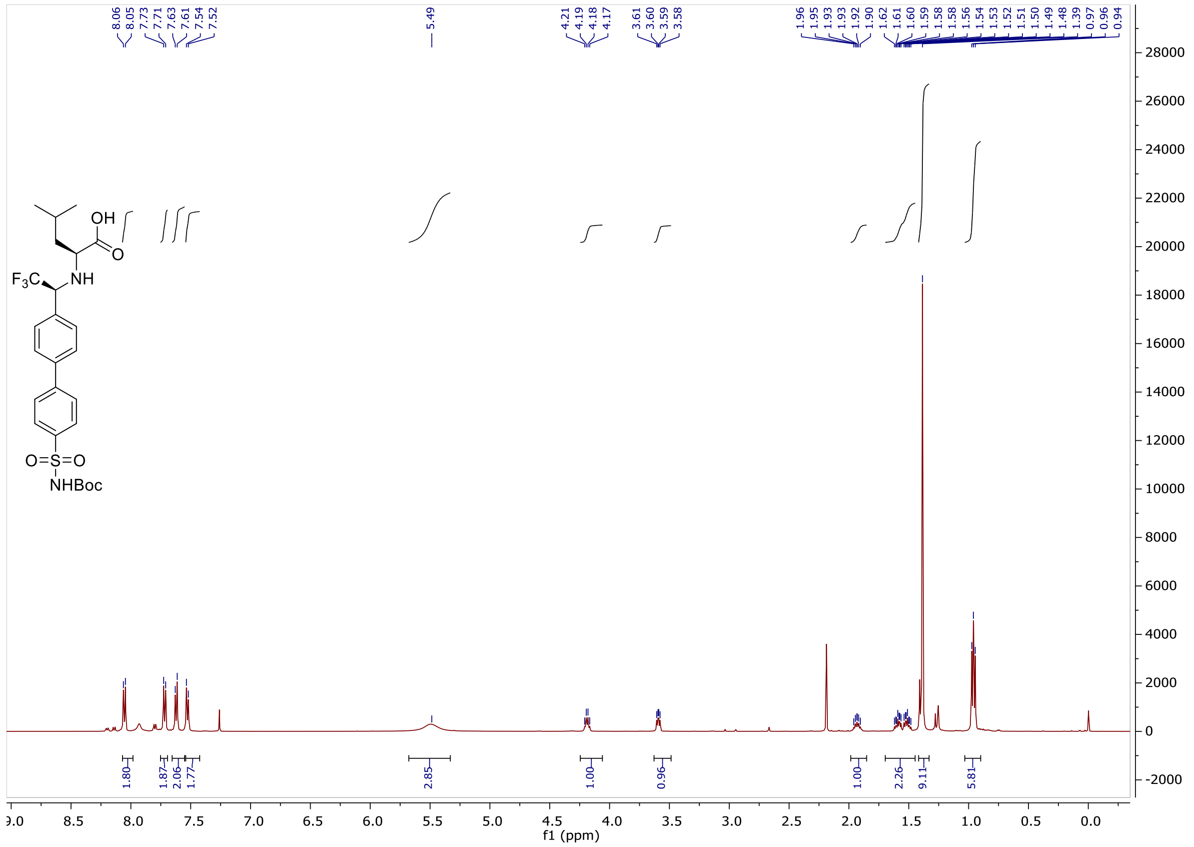

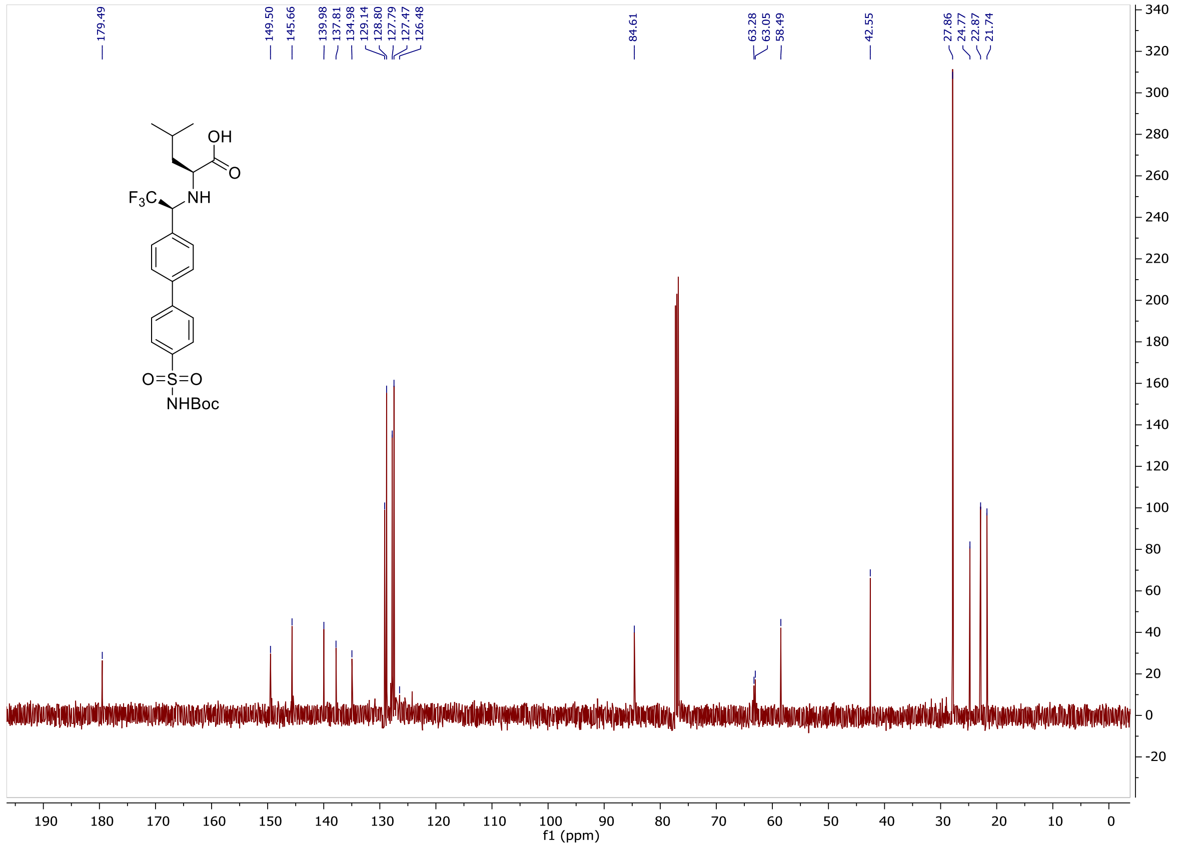

Figure S20. ^{1}H and ^{13}C NMR of Compound **90**.

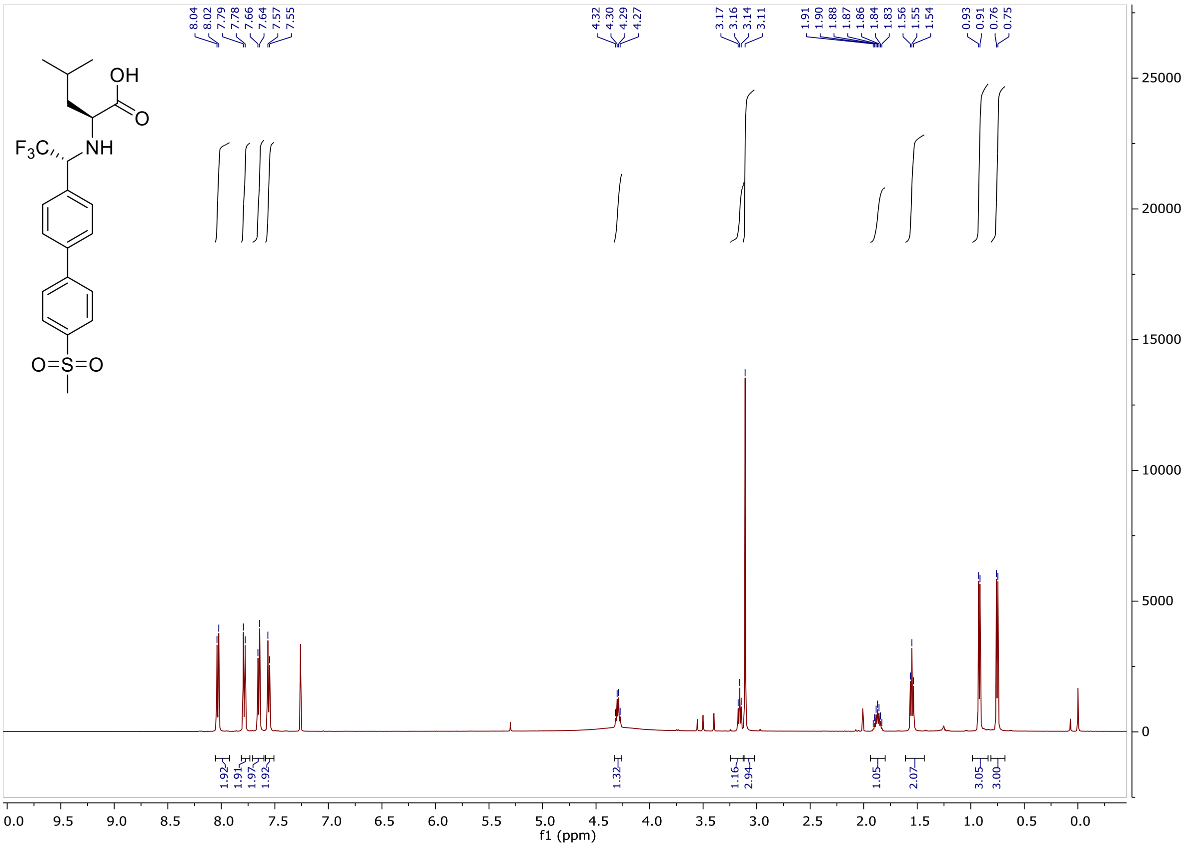

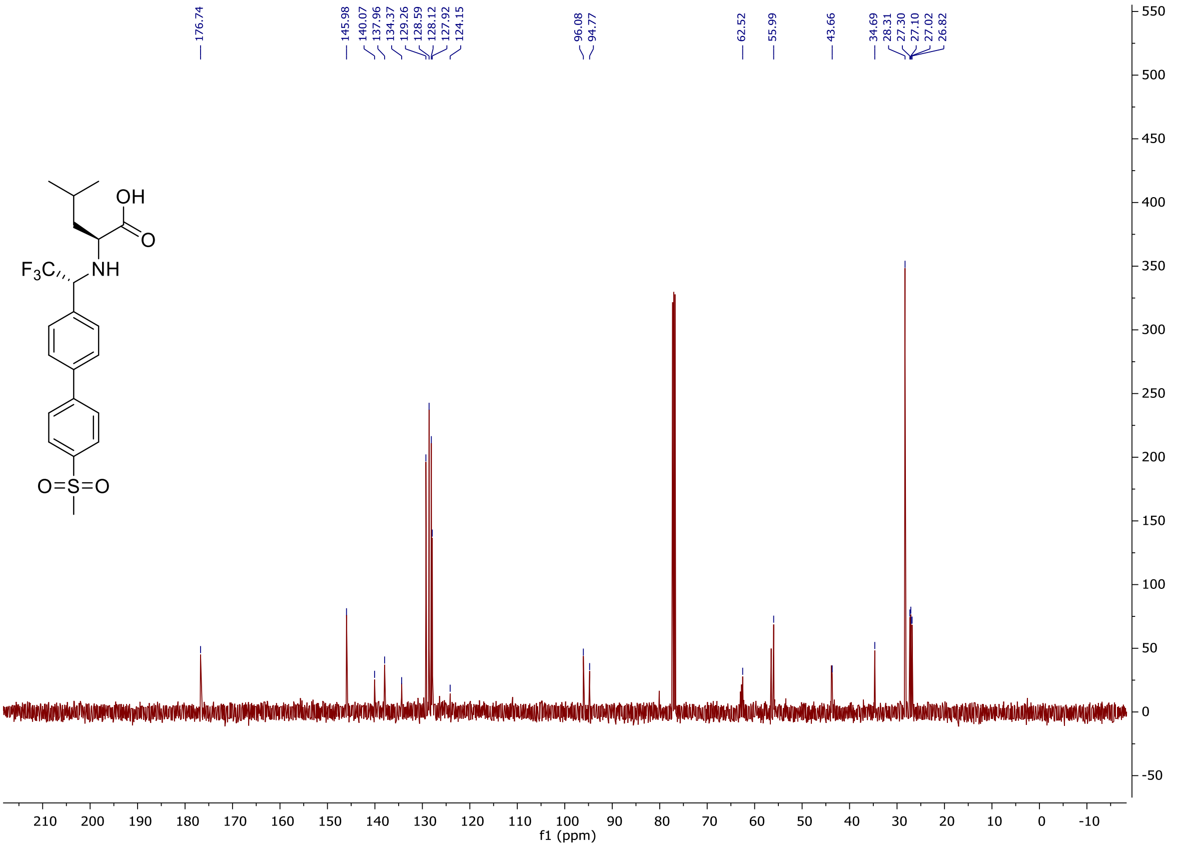

Figure S21. ^{1}H and ^{13}C NMR of Compound **99**.

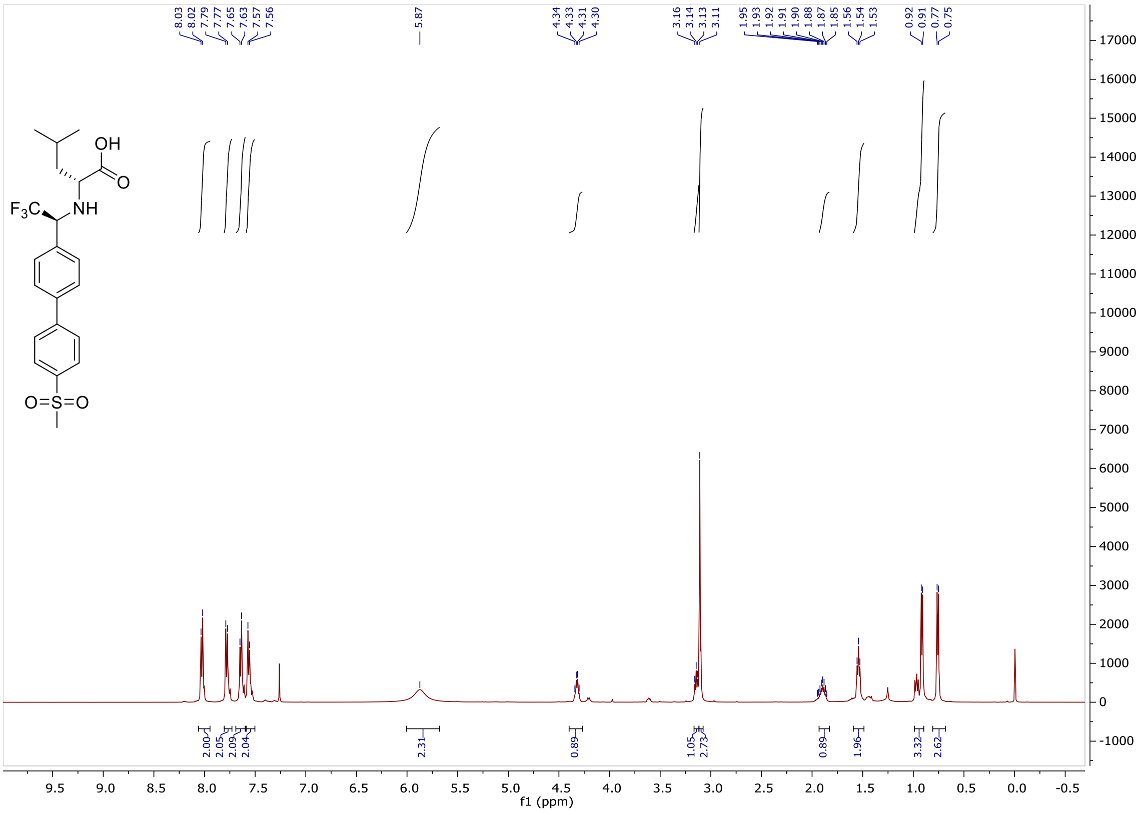

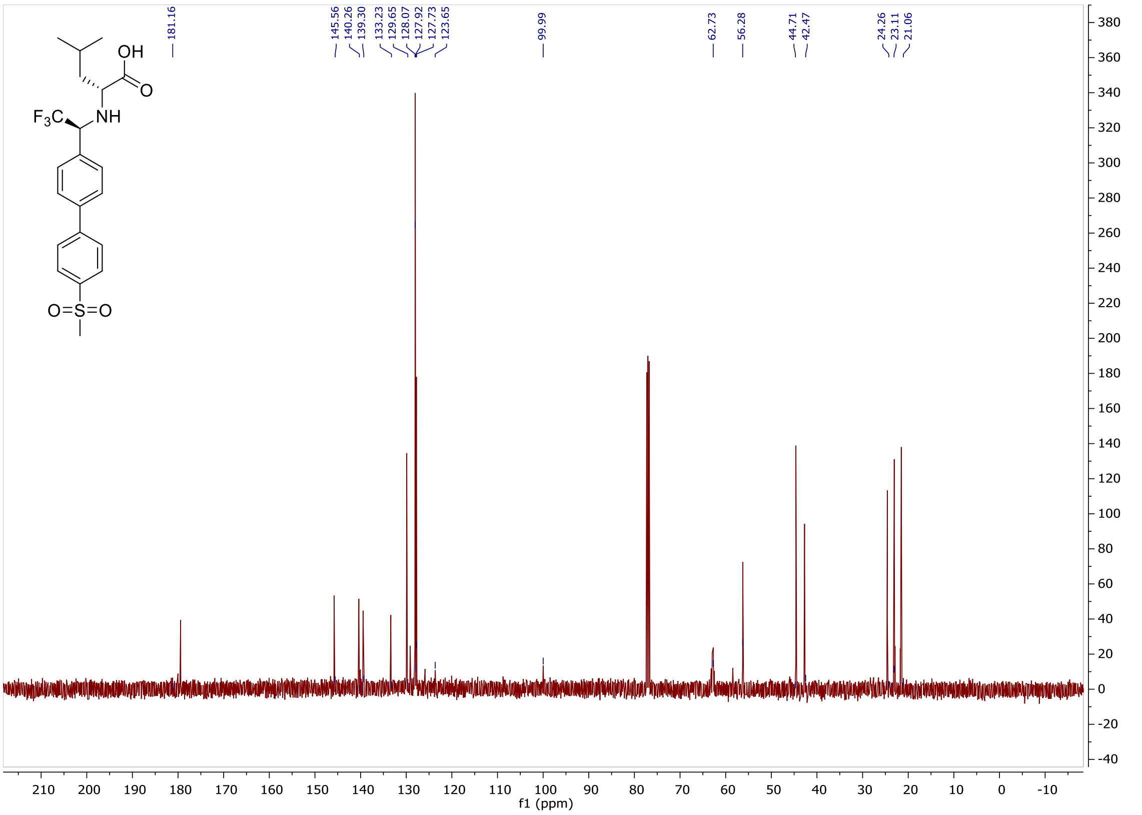

Figure S22. ^{1}H and ^{13}C NMR of Compound **101.**

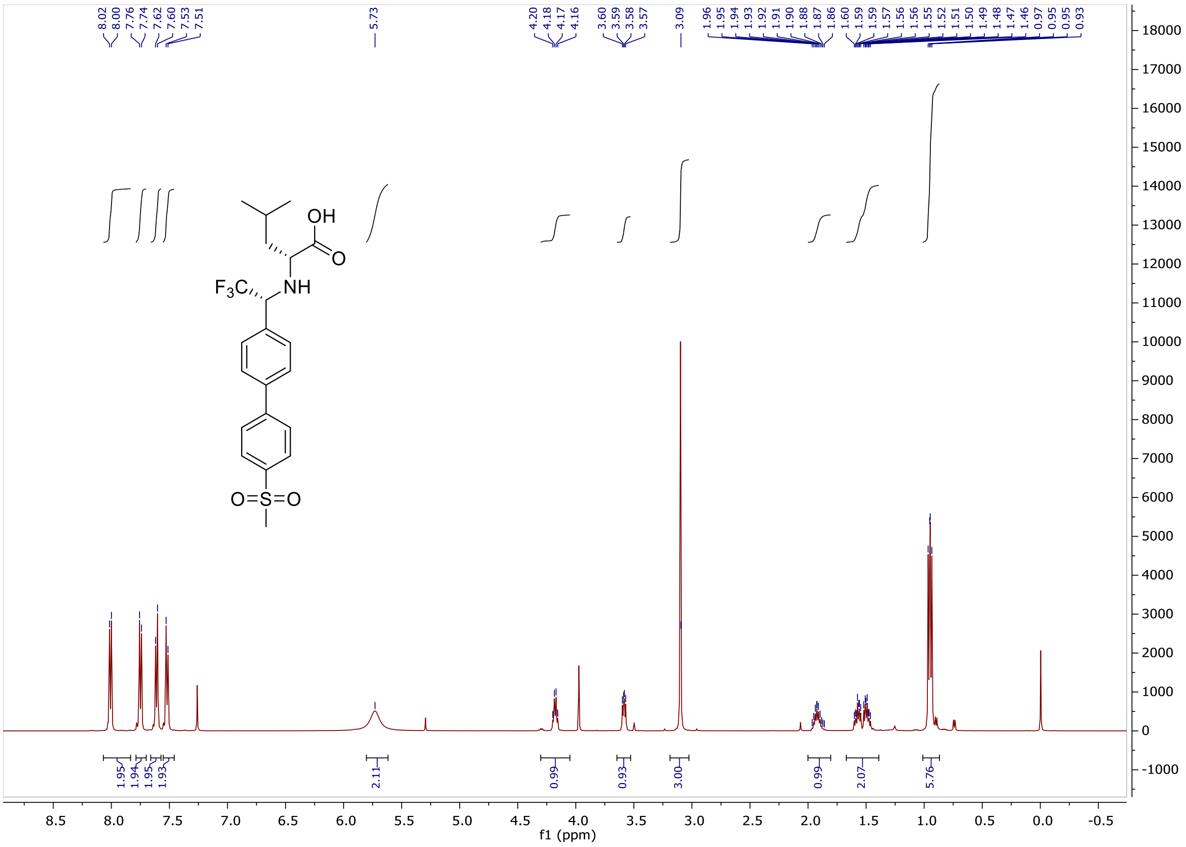

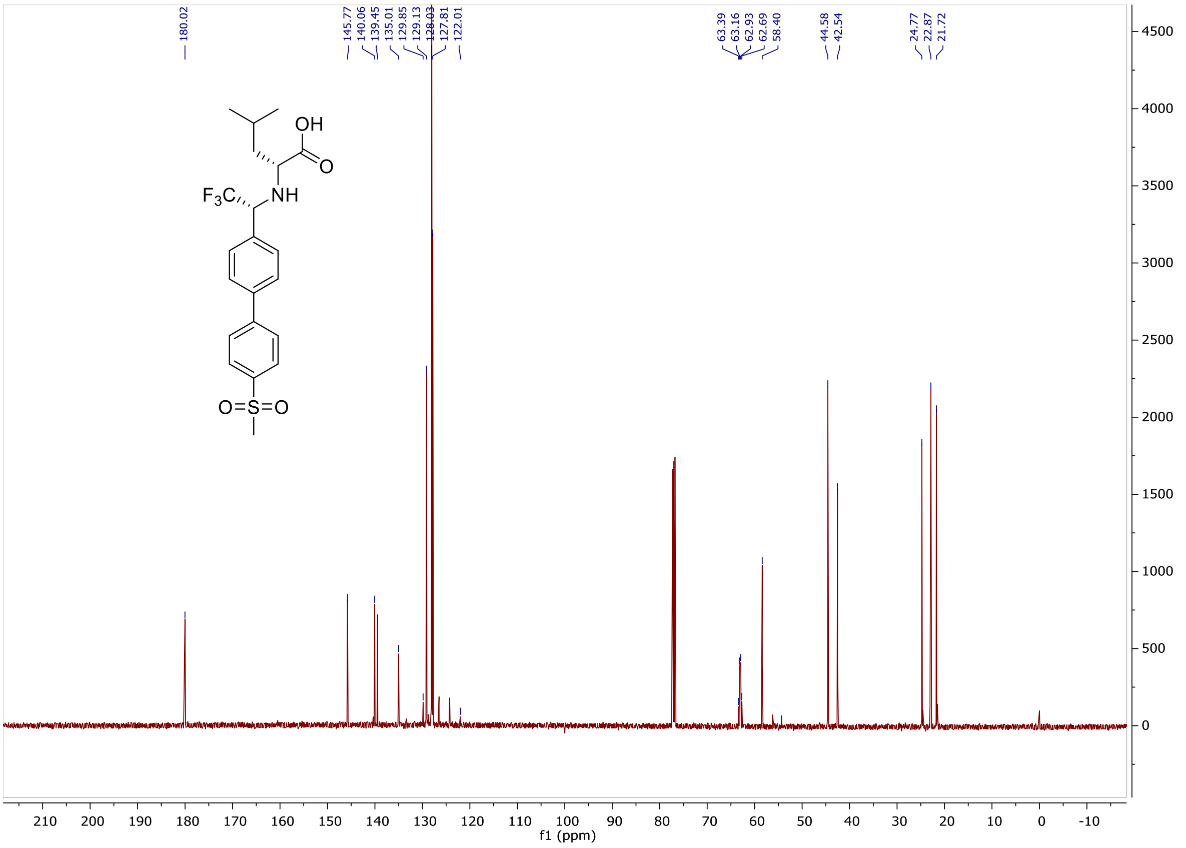

Figure S23. ^{1}H and ^{13}C NMR of Compound **103**.

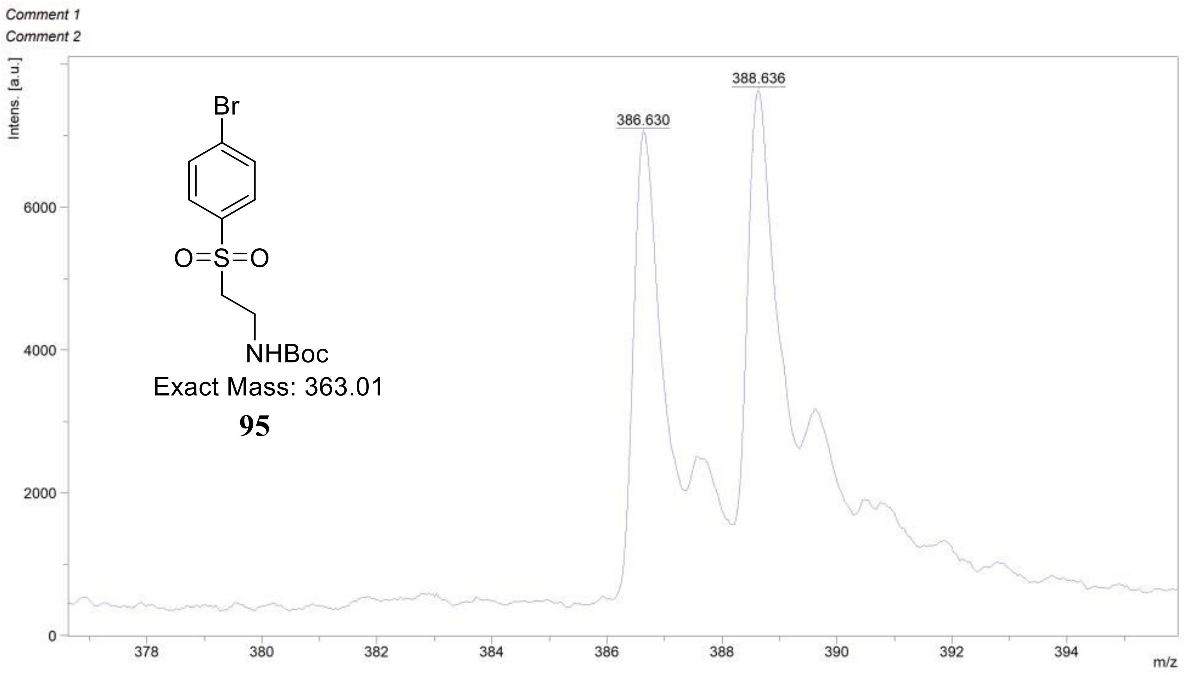

Figure S24. MALDI of Compound **95**.

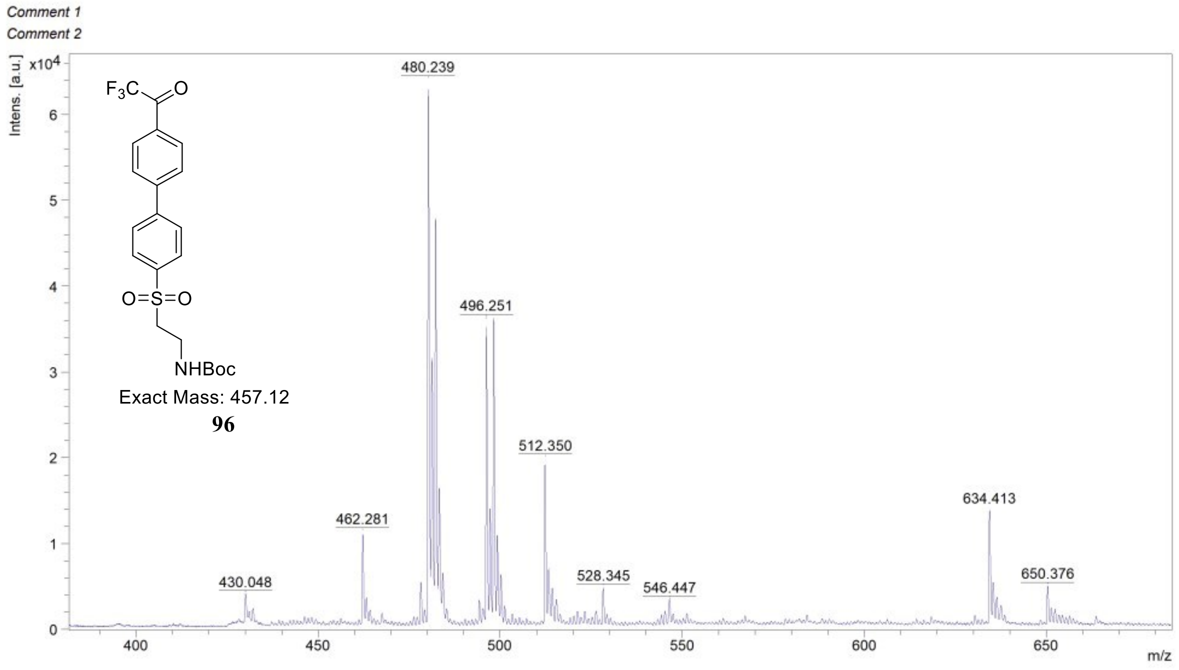

Figure S25. MALDI of Compound **96**.

LCMS of Compounds

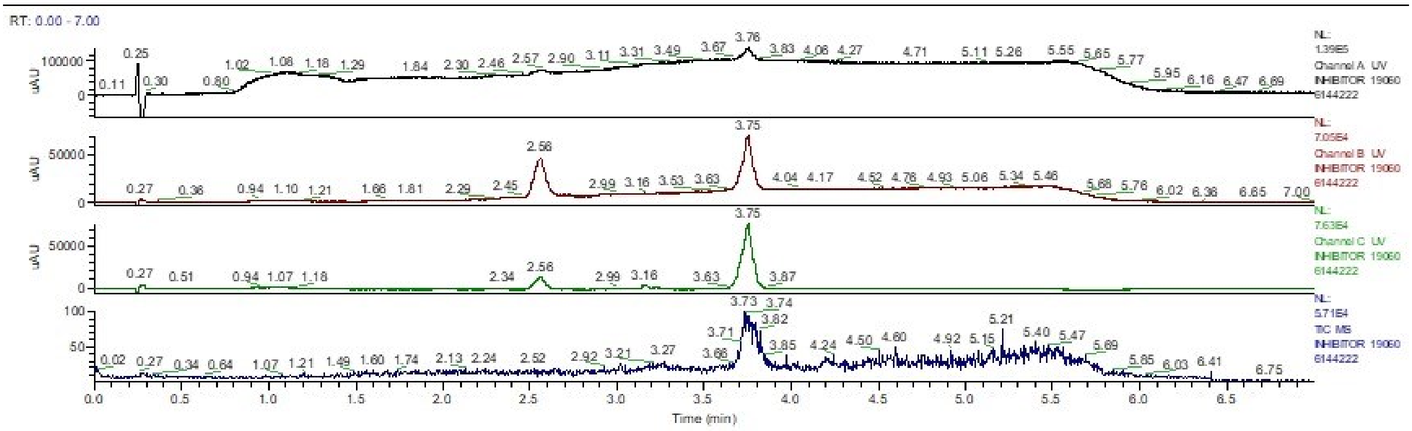

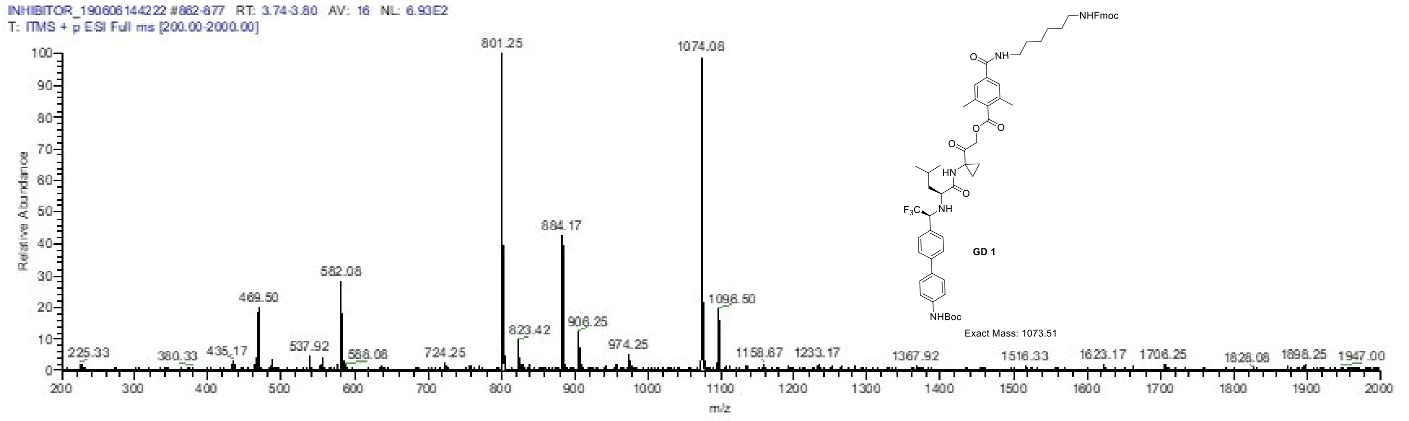

Figure S26. LCMS of Compound **GD1**.

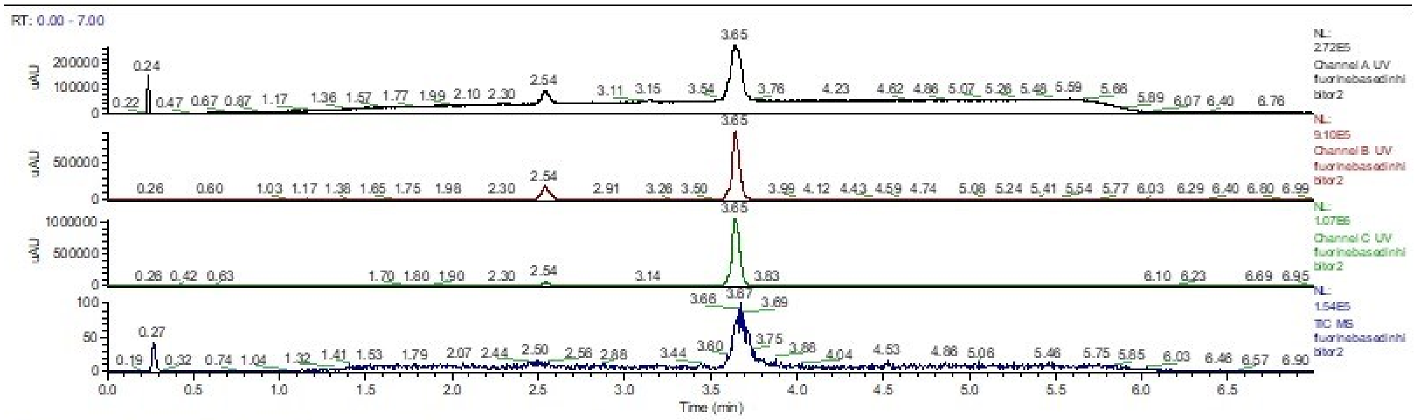

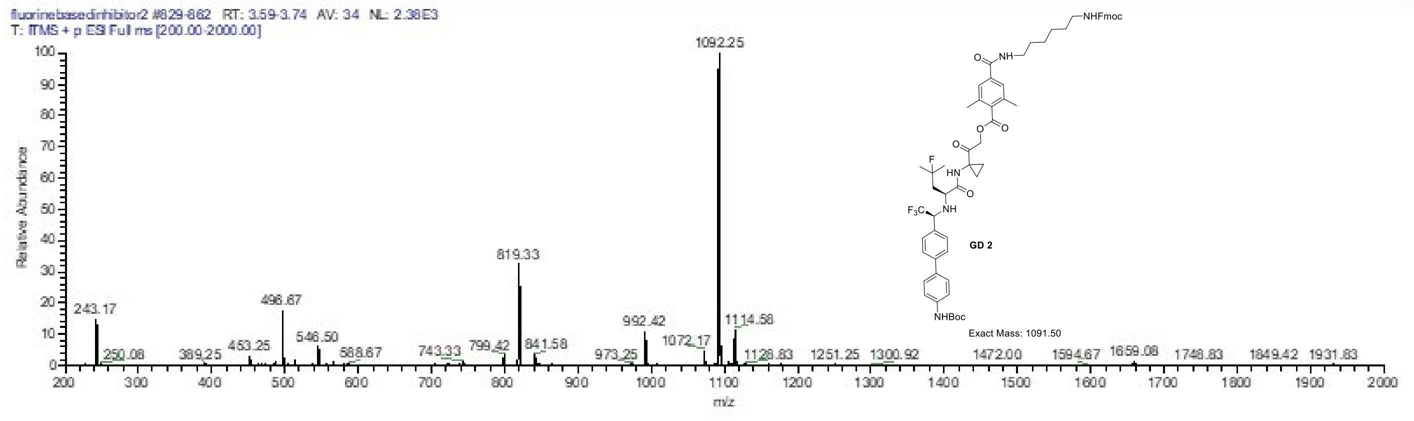

Figure S27. LCMS of Compound **GD2**.

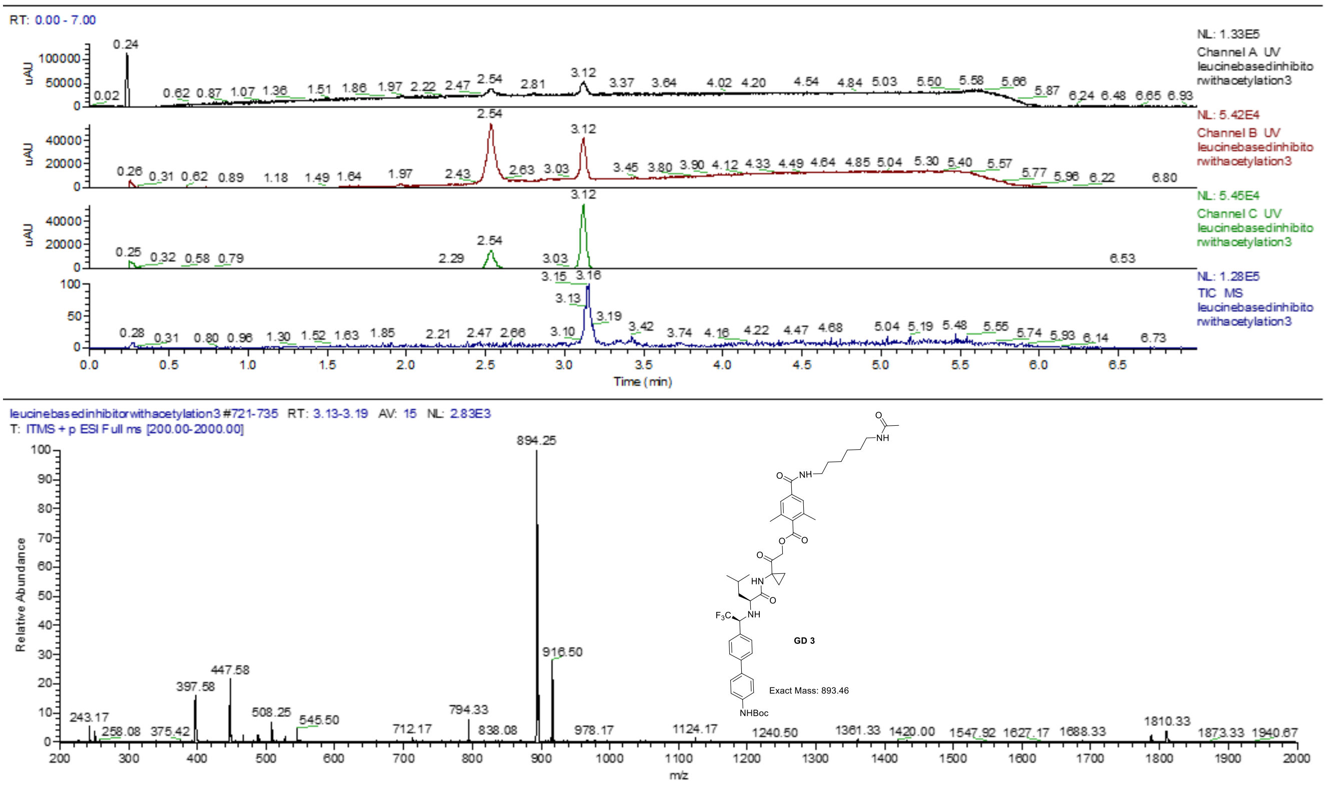

Figure S28. LCMS of Compound **GD3**.

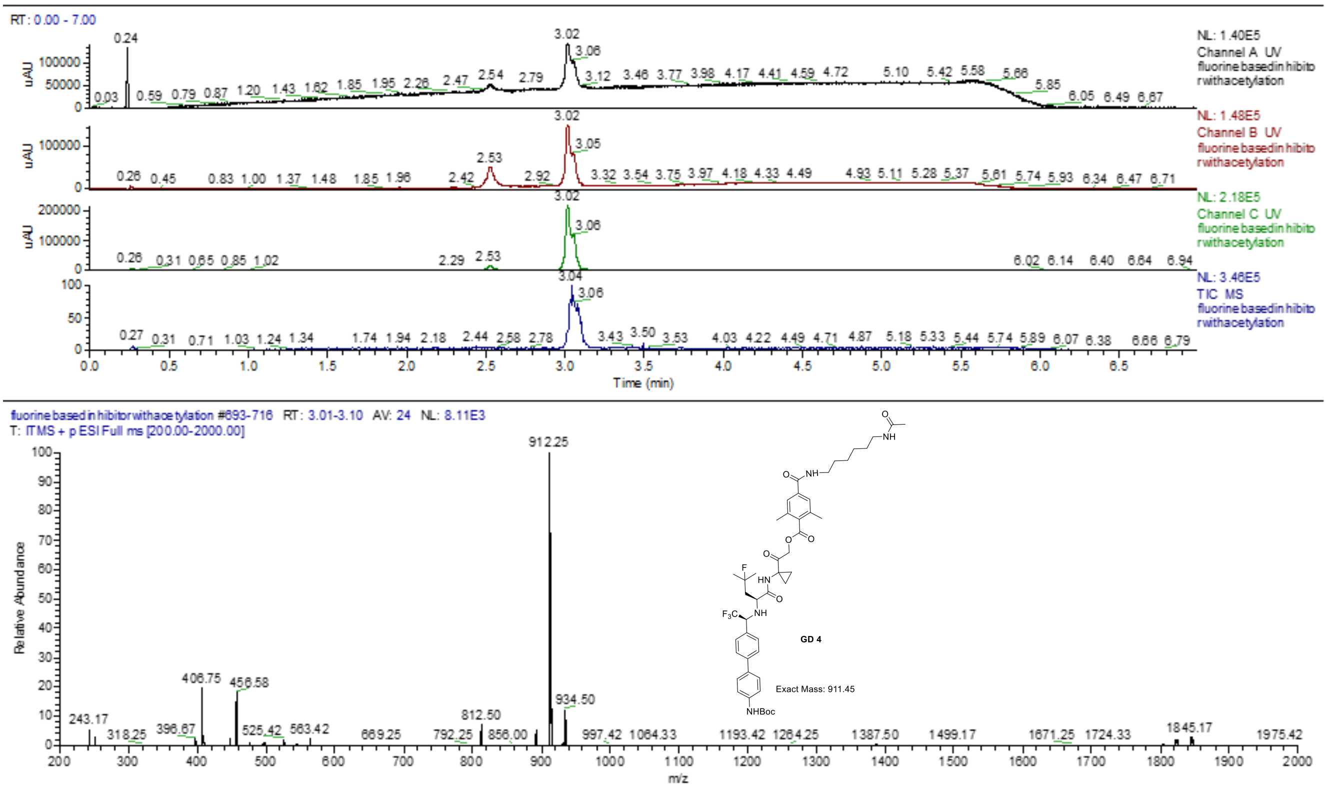

Figure S29. LCMS of Compound **GD4**.

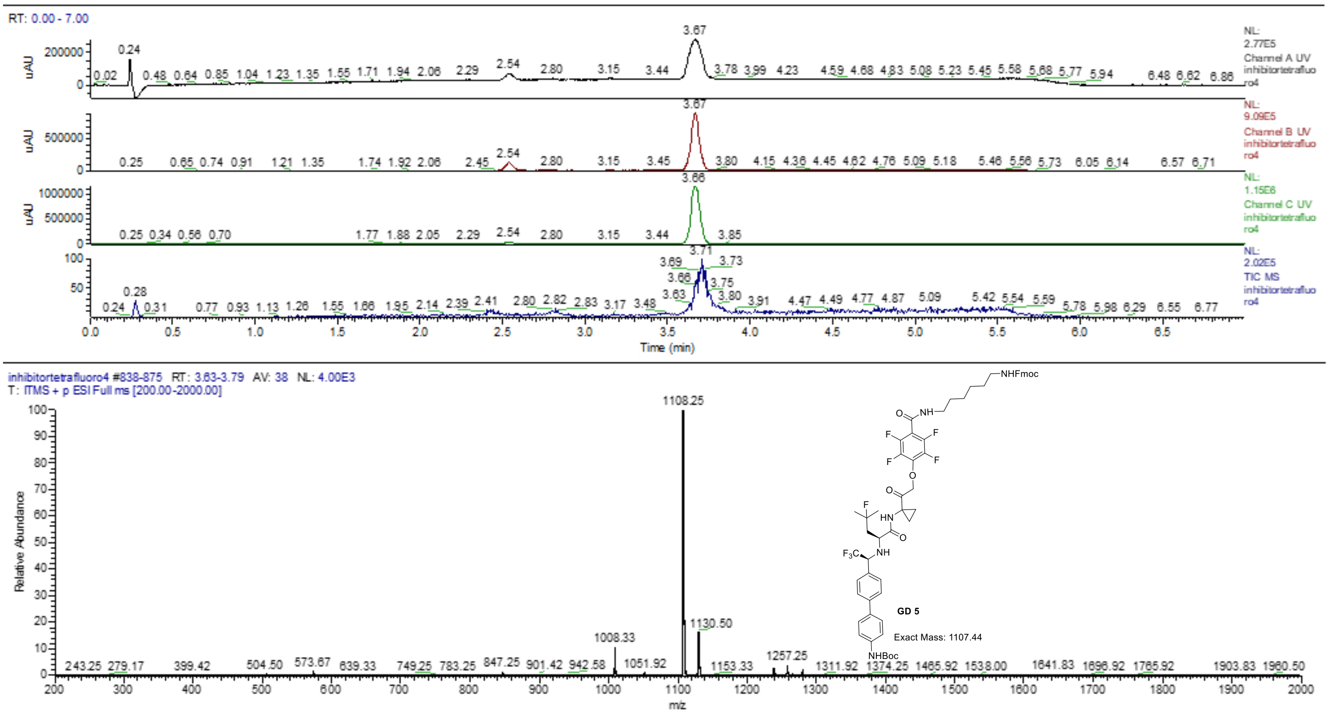

Figure S30. LCMS of Compound **GD5**.

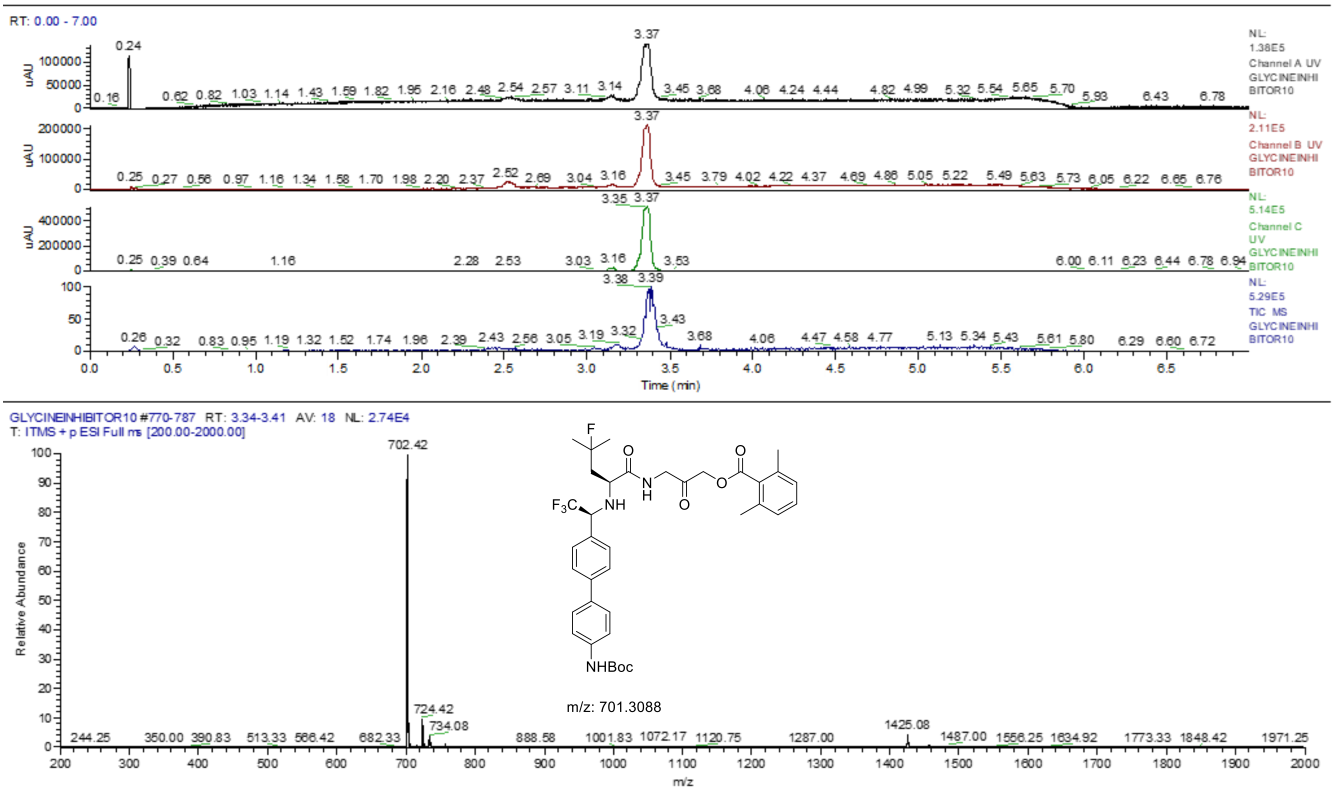

Figure S31. LCMS of Compound **GD6**.

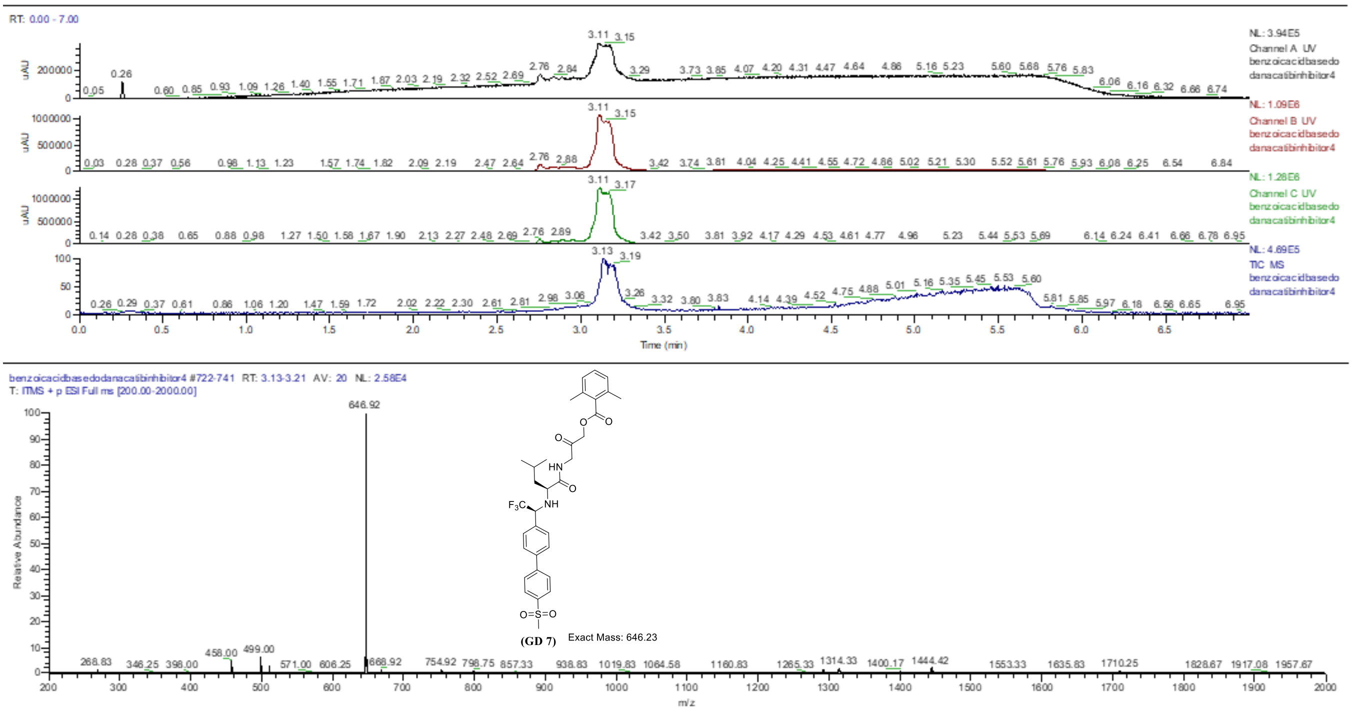

Figure S32. LCMS of Compound **GD7**.

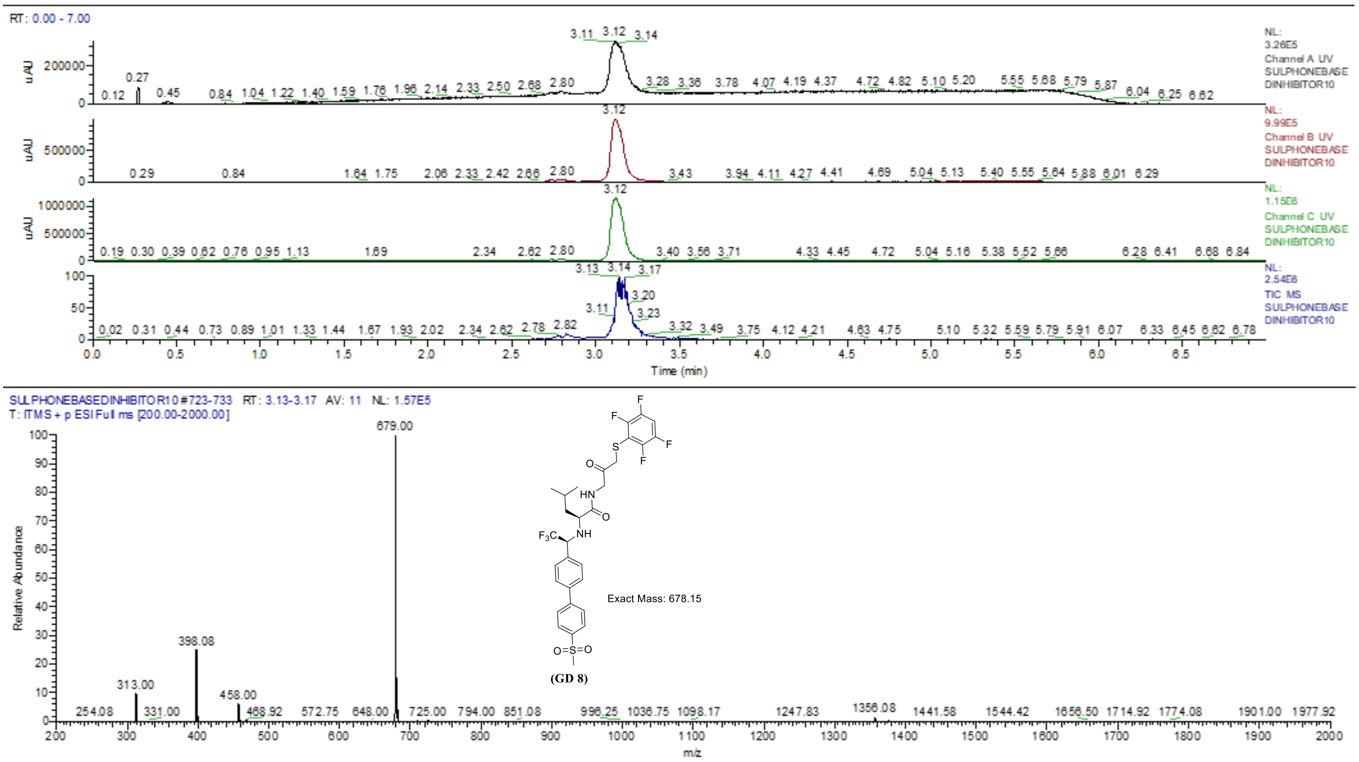

Figure S33. LCMS of Compound **GD8**.

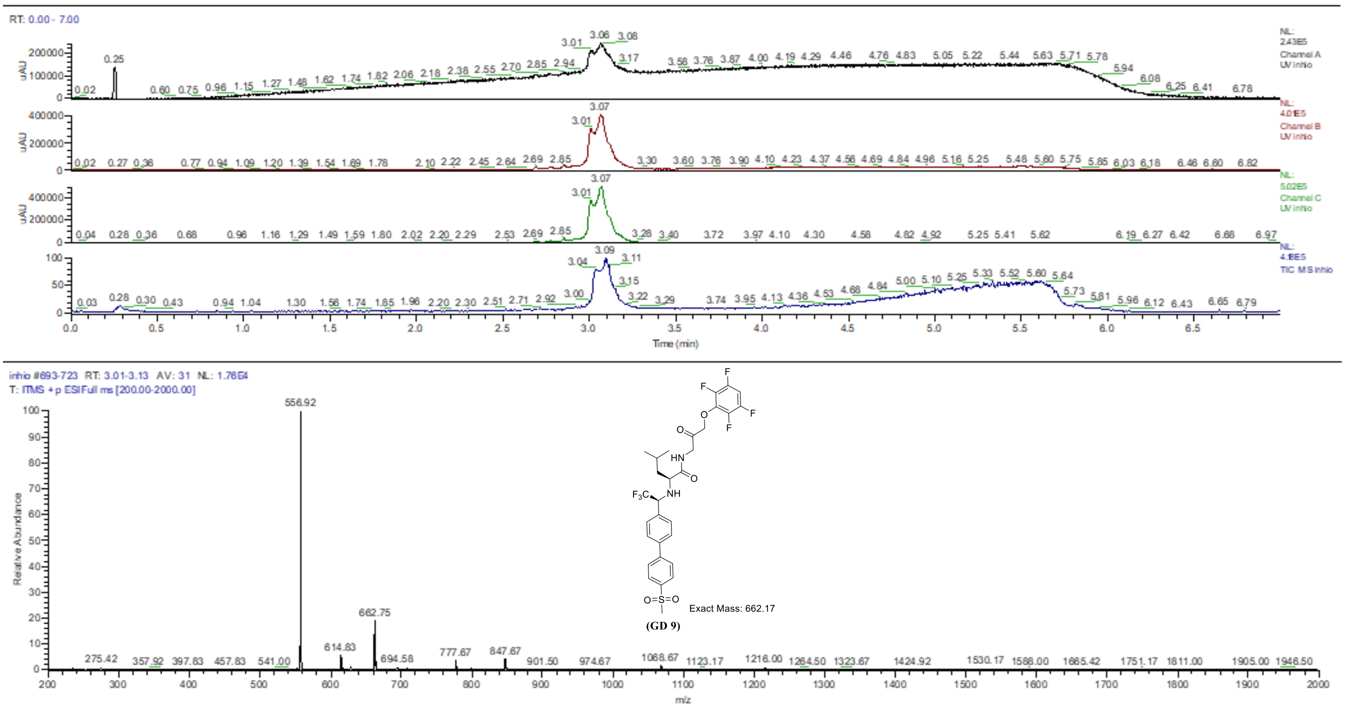

Figure S34. LCMS of Compound **GD9**.

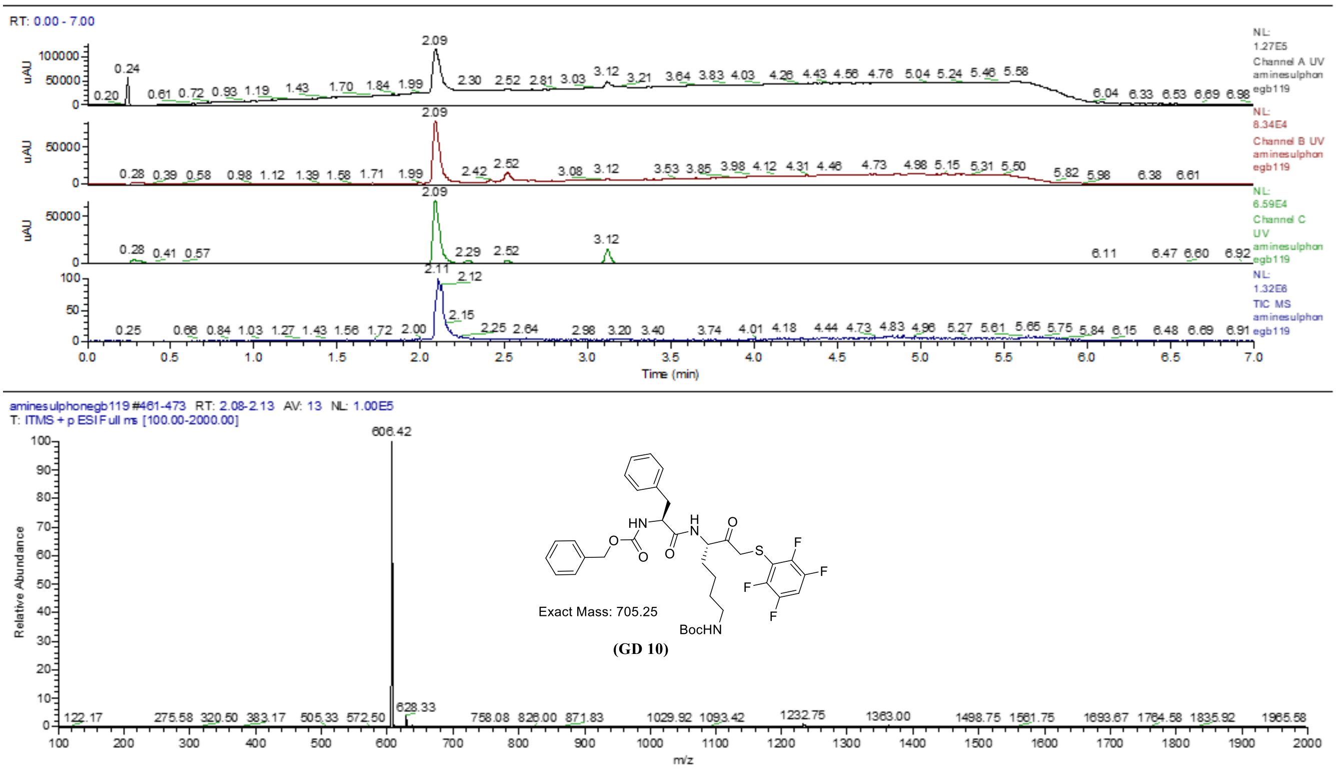

Figure S35. LCMS of Compound **GD10**.

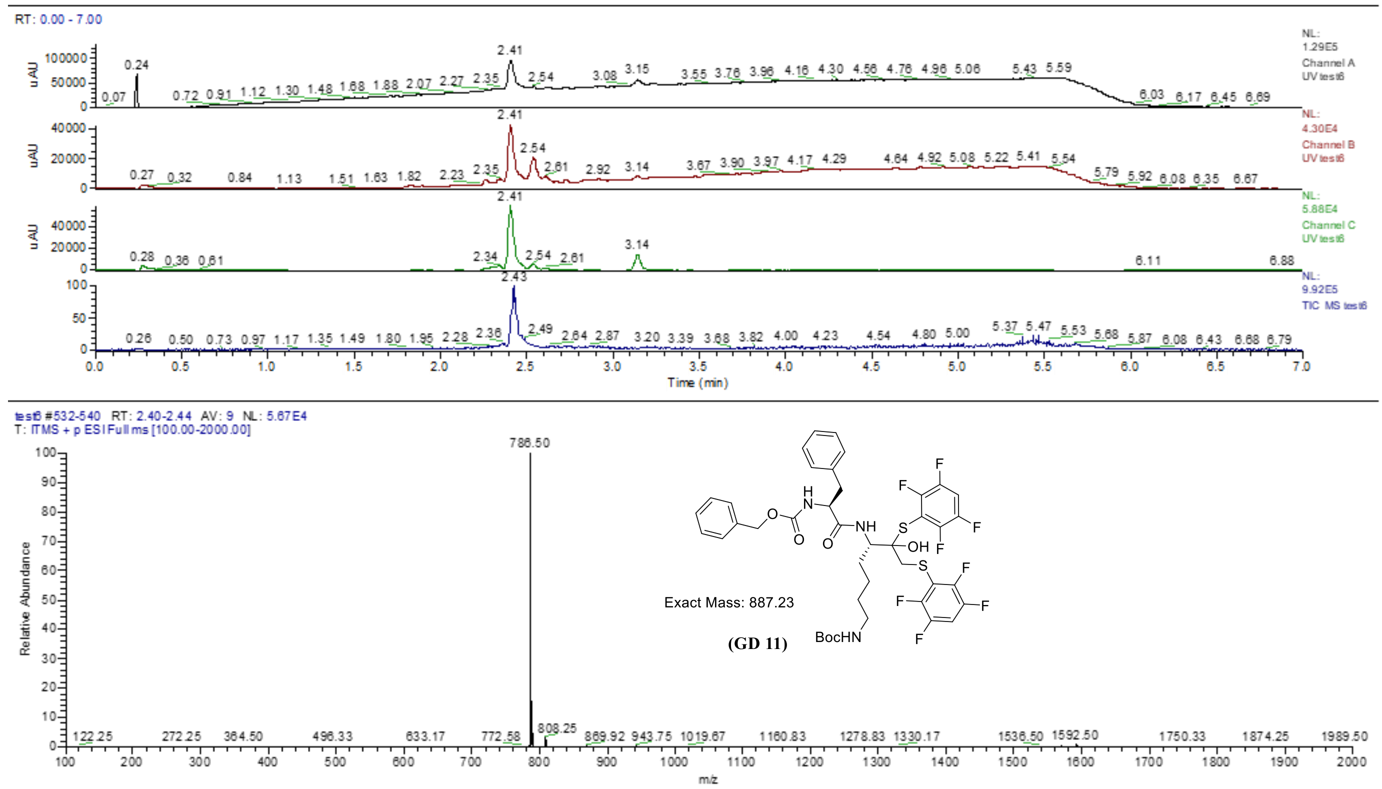

Figure S36. LCMS of Compound **GD11**.

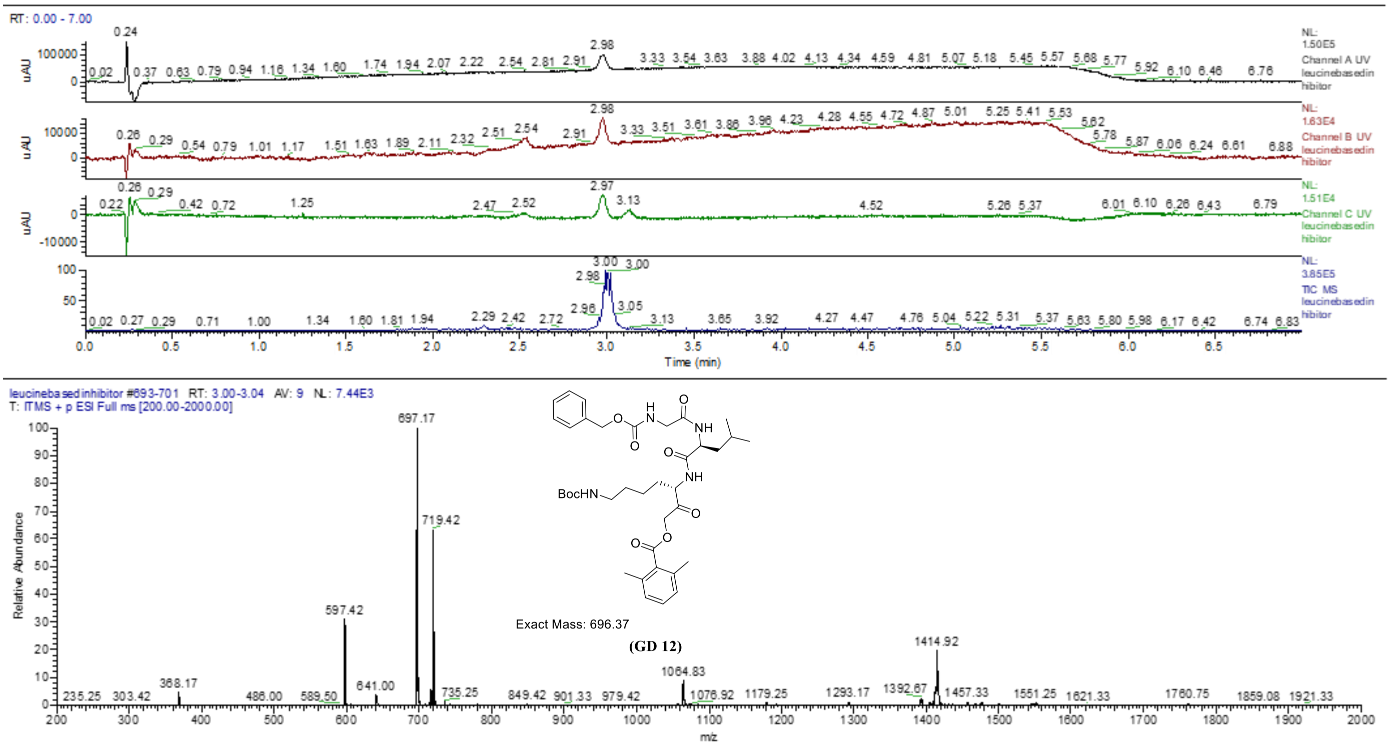

Figure S37. LCMS of Compound **GD12**.

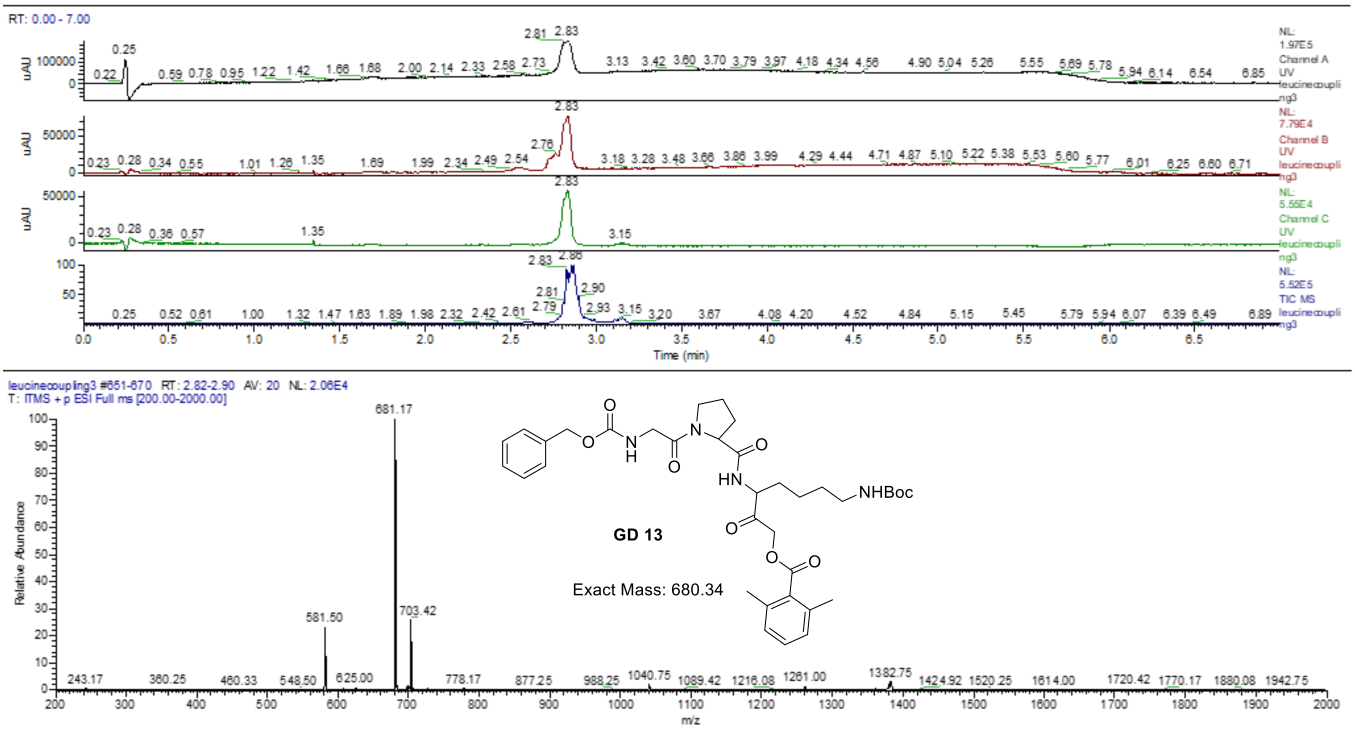

Figure S38. LCMS of Compound **GD13**.

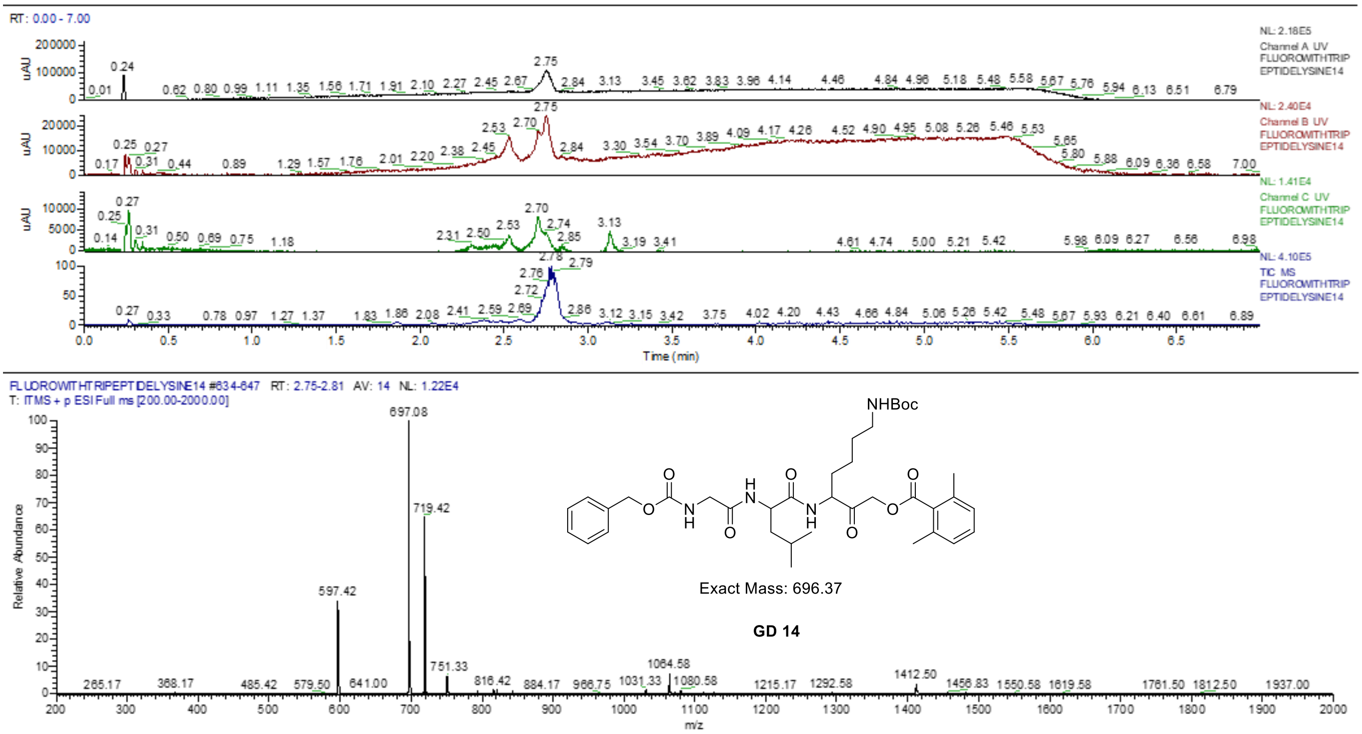

Figure S39. LCMS of Compound **GD14**.

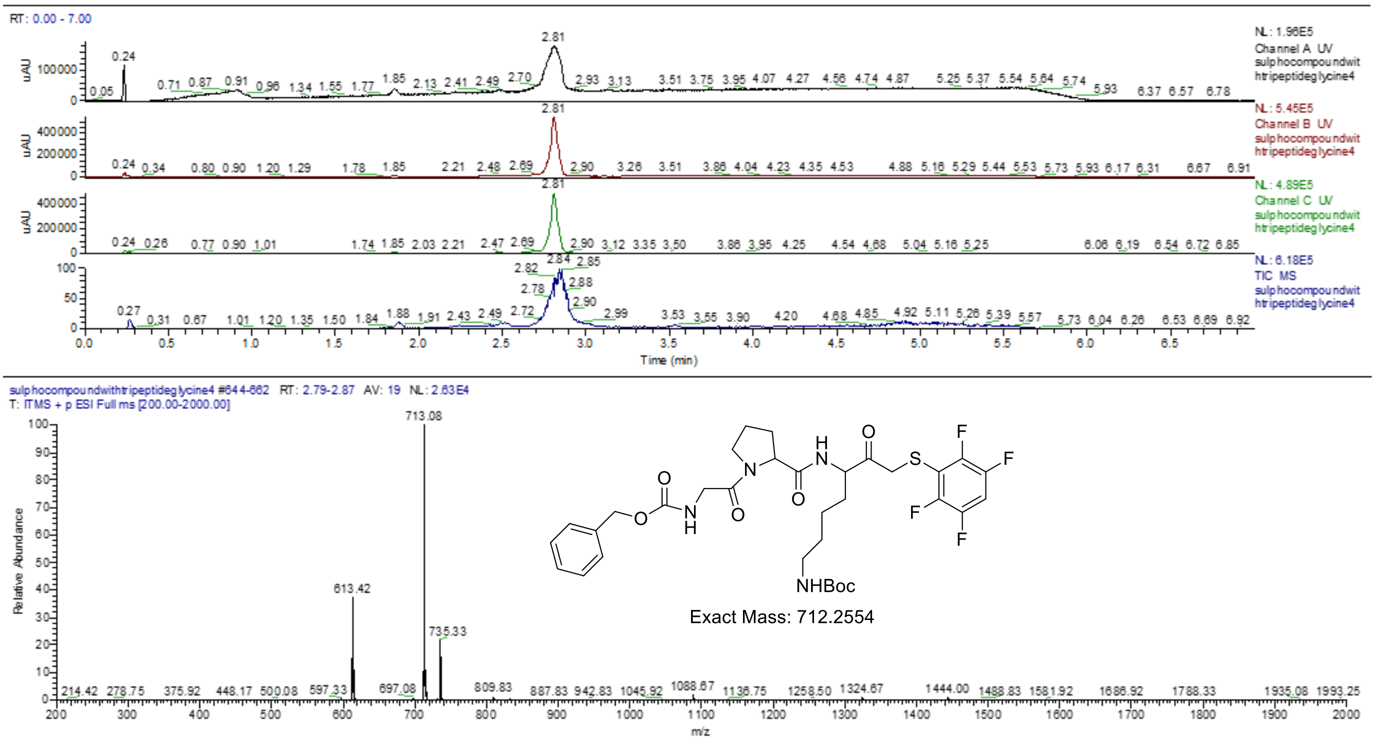

Figure S40. LCMS of Compound **GD15**.

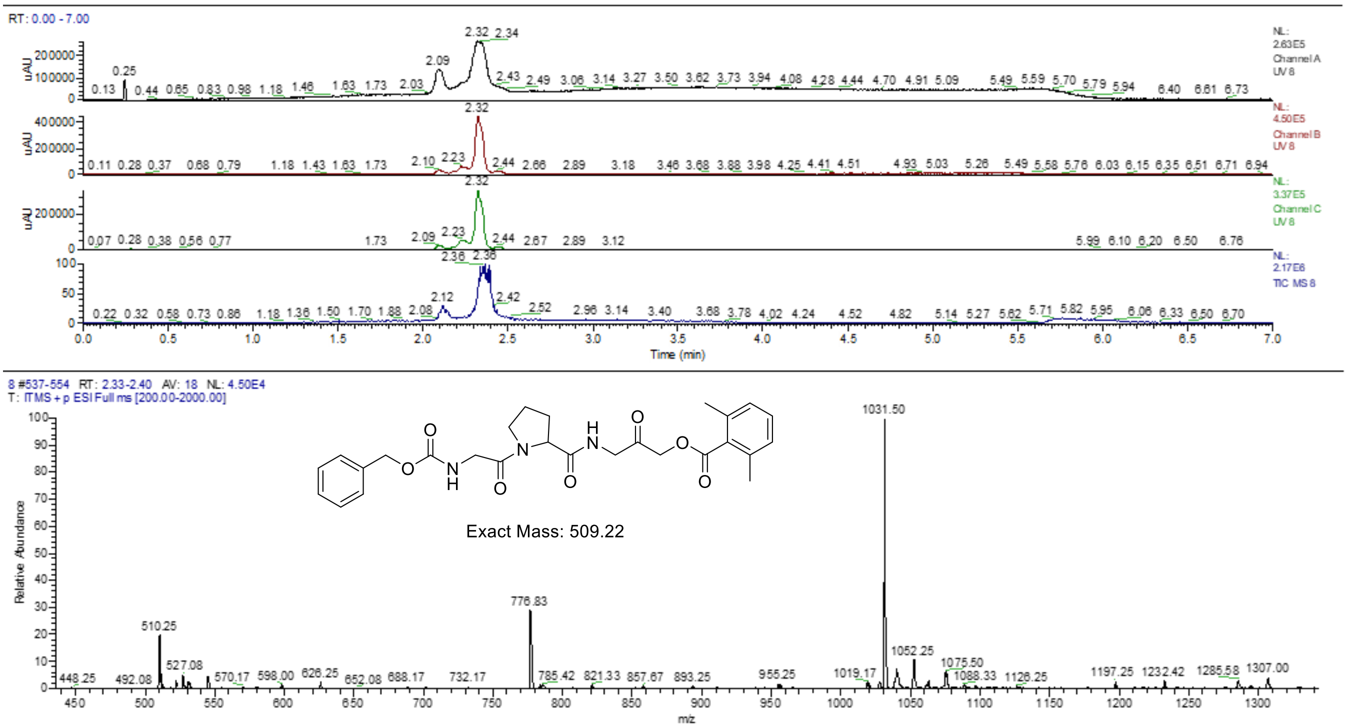

Figure S41. LCMS of Compound **GD16**.

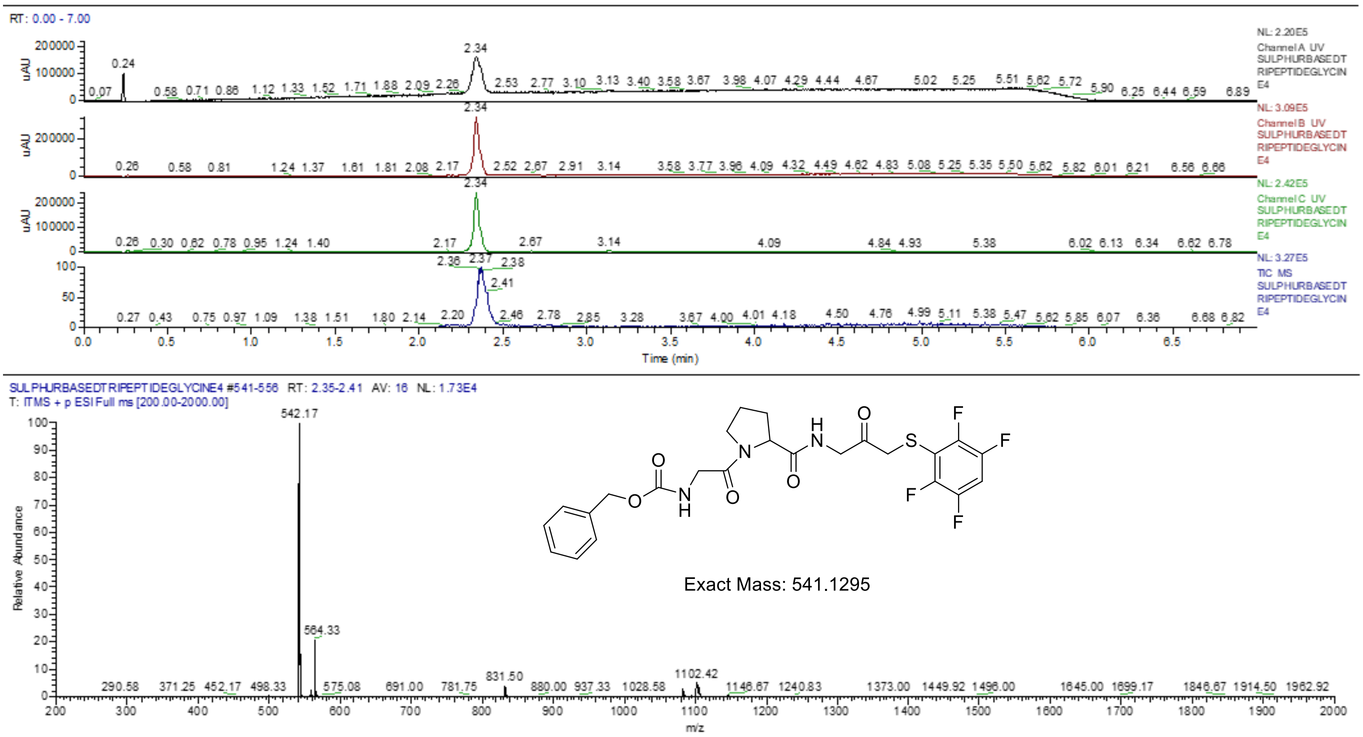

Figure S42. LCMS of Compound **GD17**.

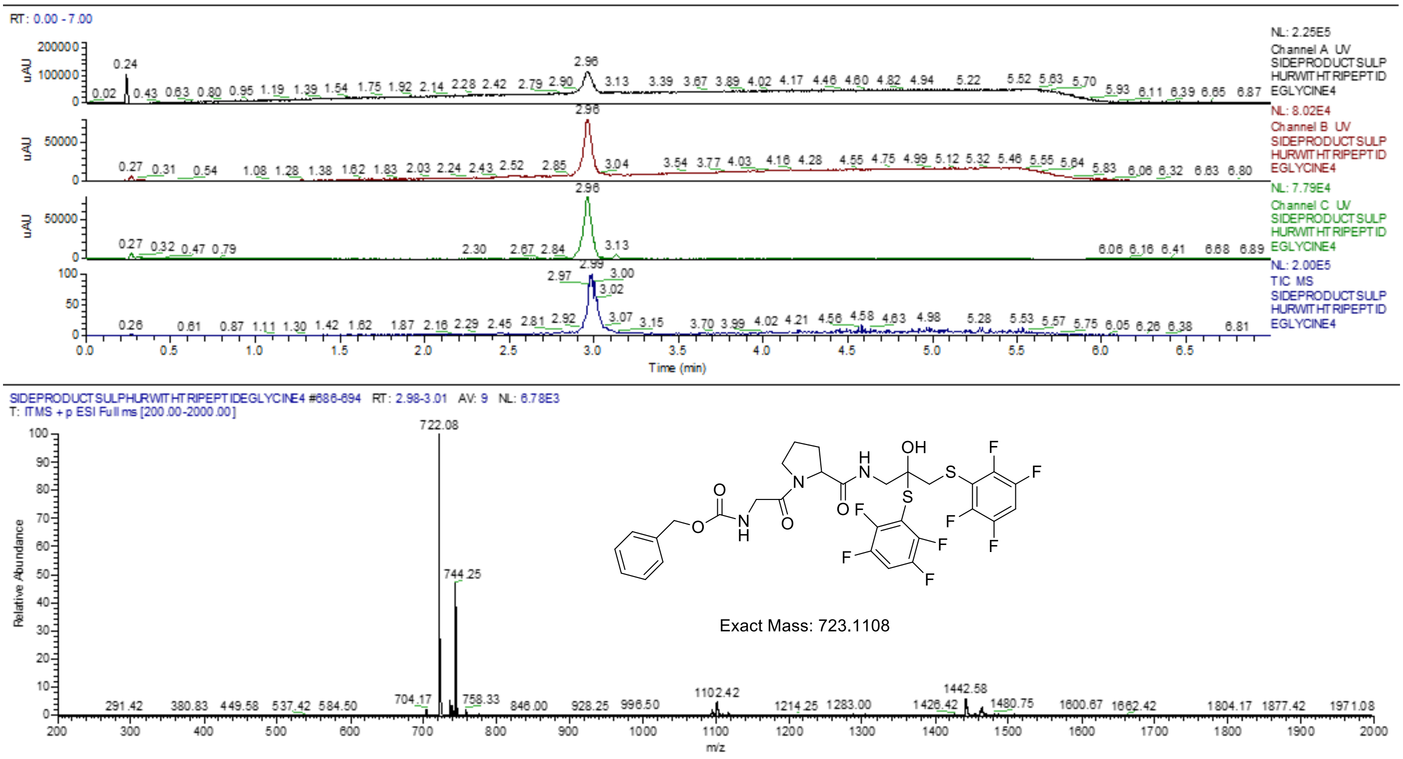

Figure S43. LCMS of Compound **GD18**.

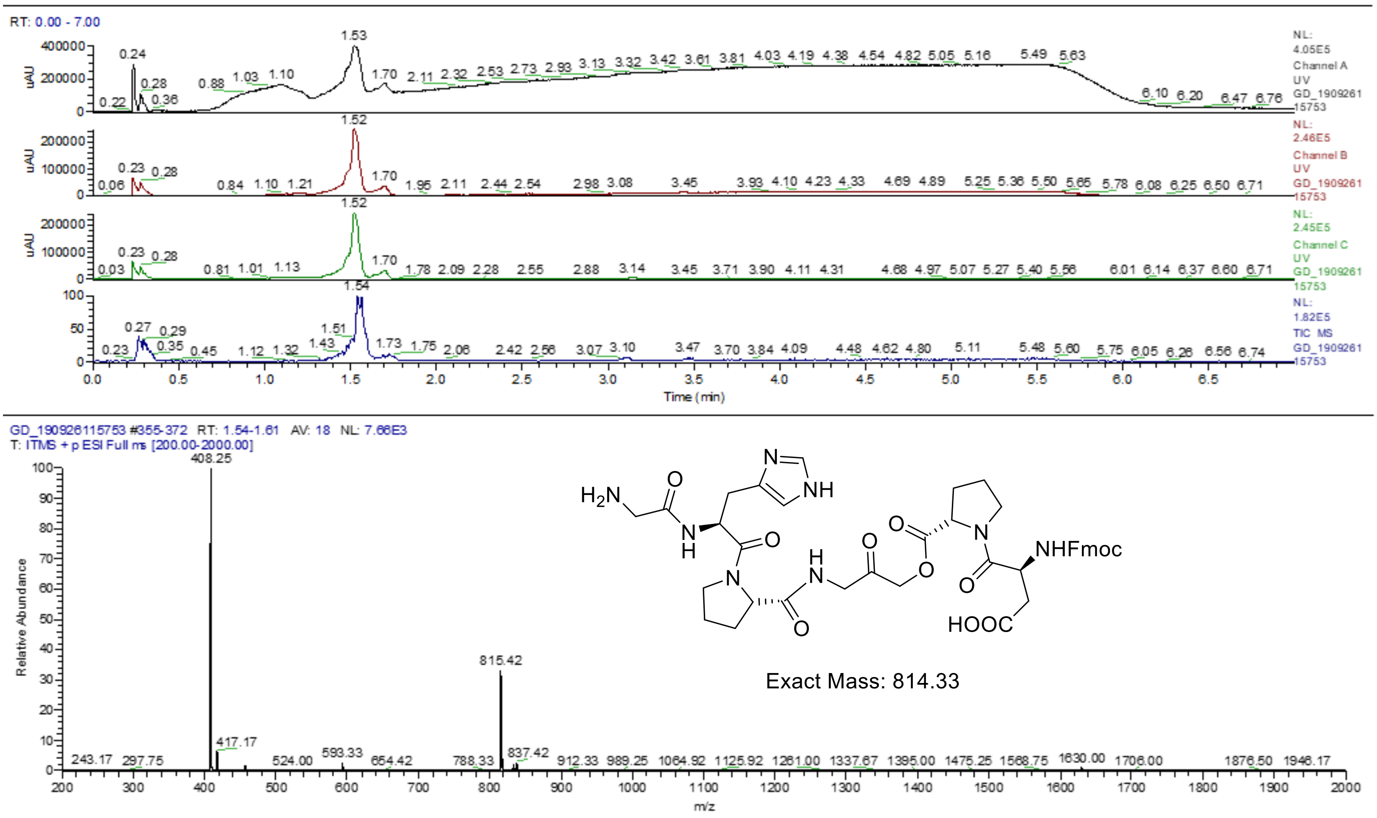

Figure S44. LCMS of Compound **GD19.**

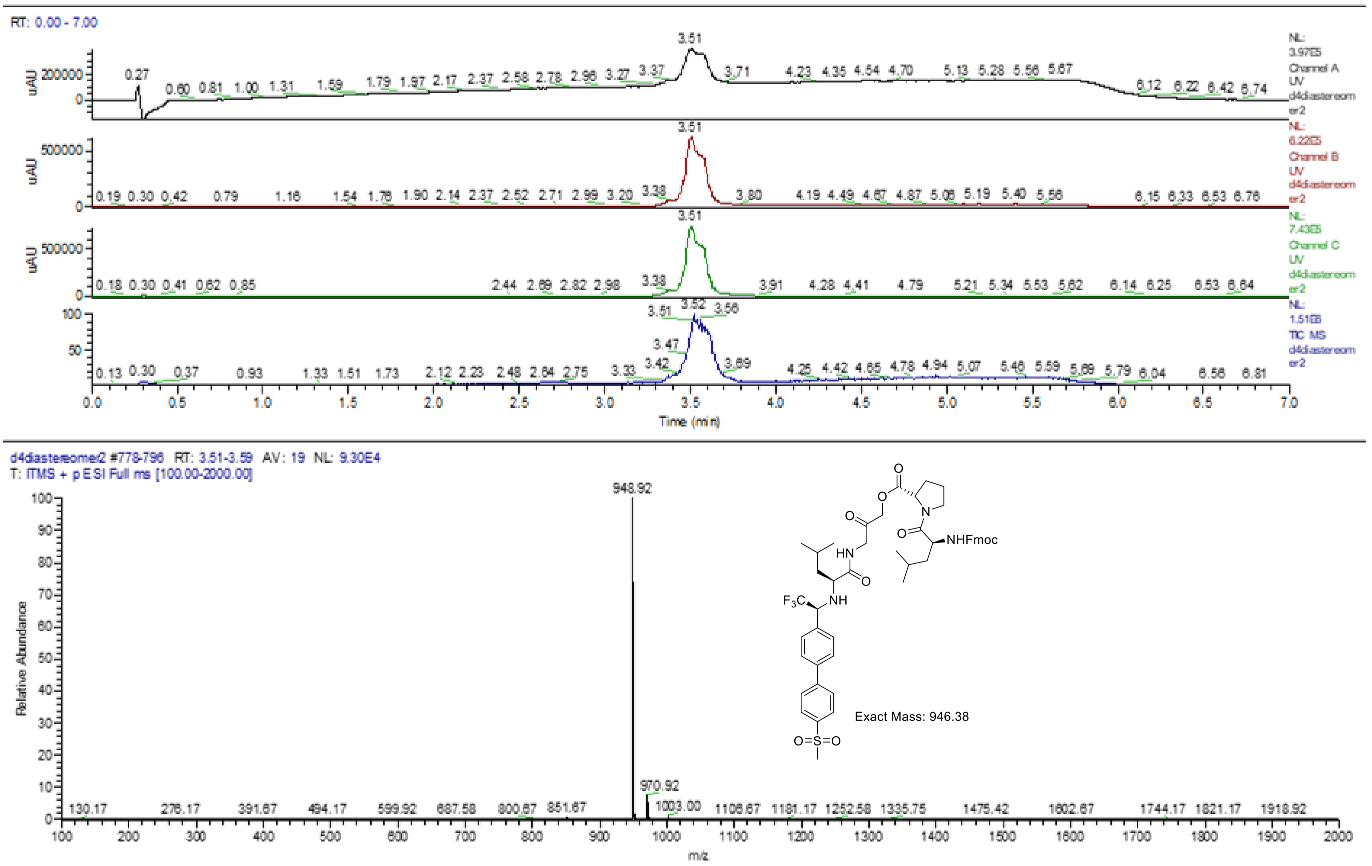

Figure S45. LCMS of Compound **GD20**.

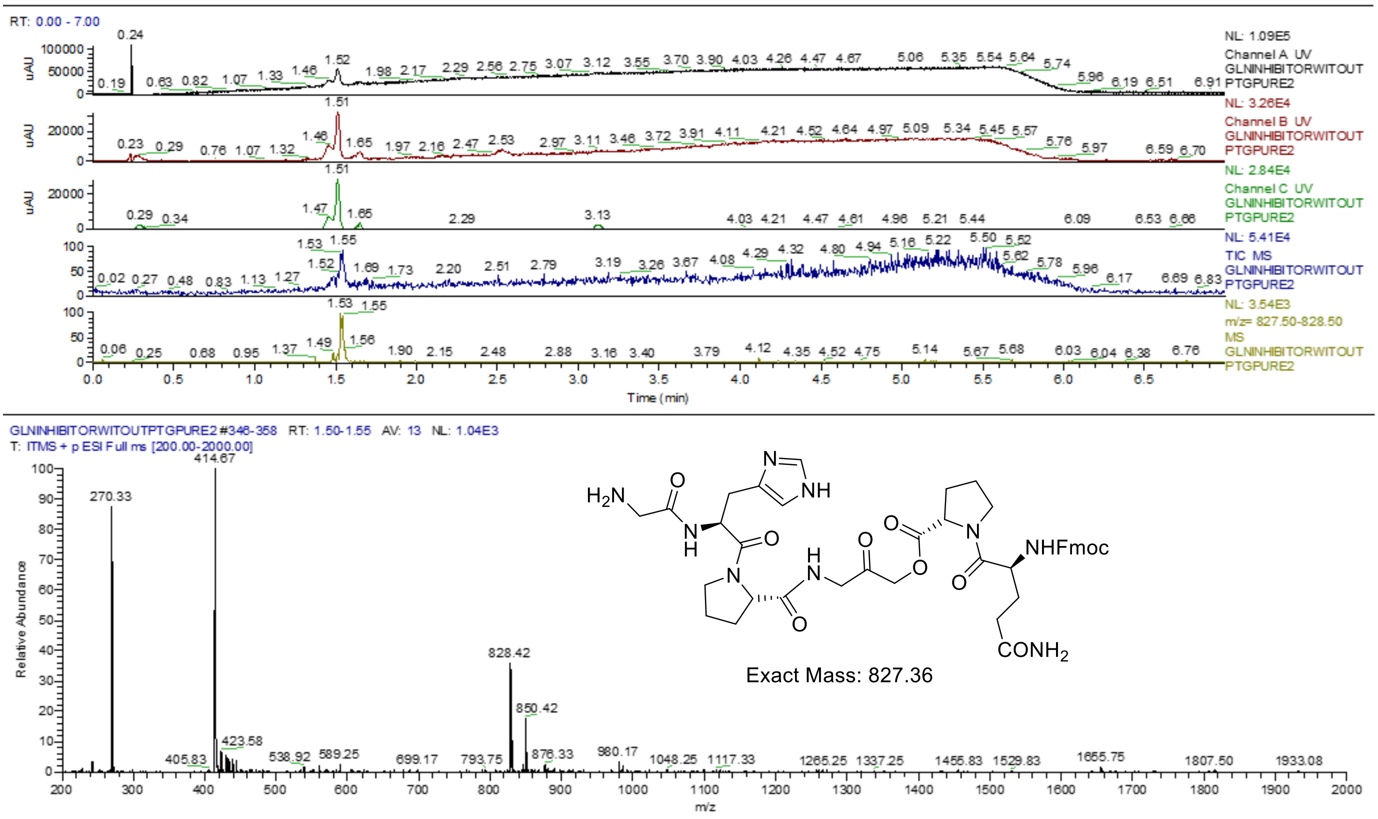

Figure S46. LCMS of Compound **GD22**.

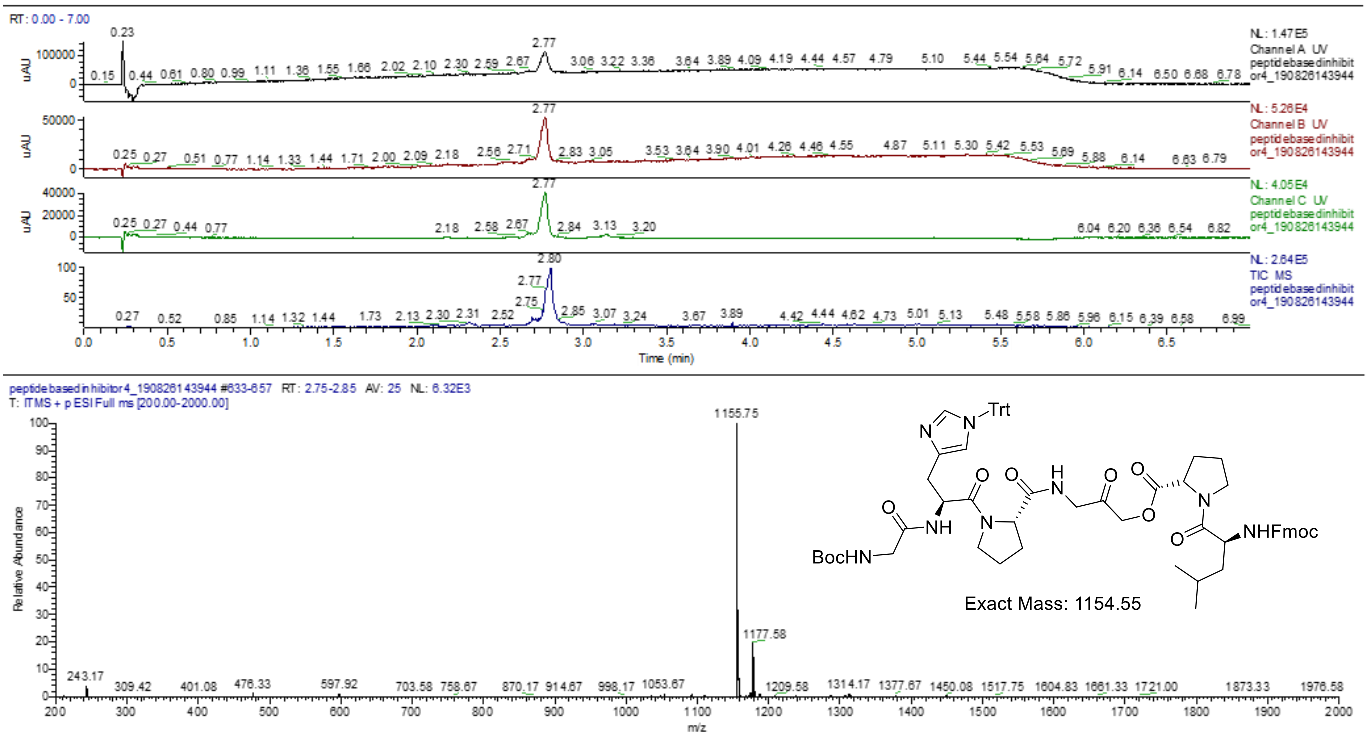

Figure S47. LCMS of Compound **GD23**.

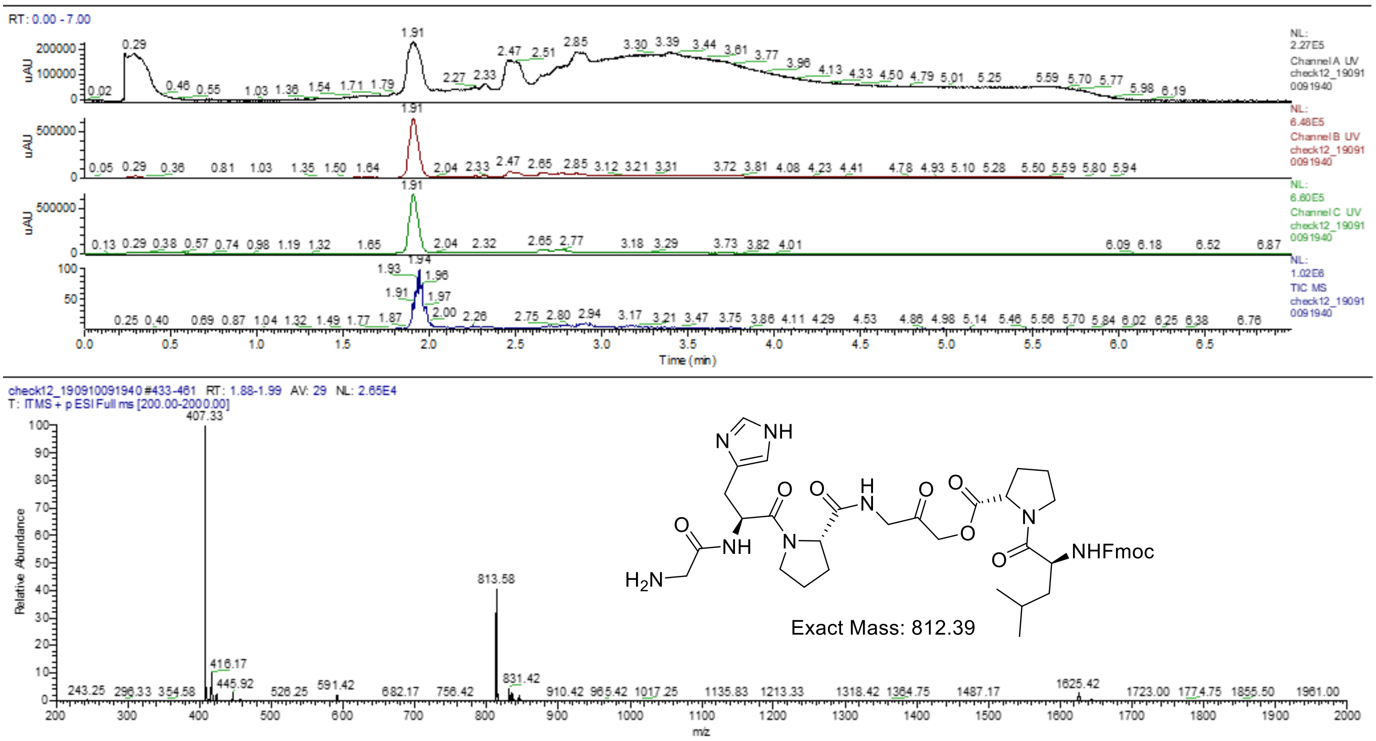

Figure S48. LCMS of Compound **GD24**.

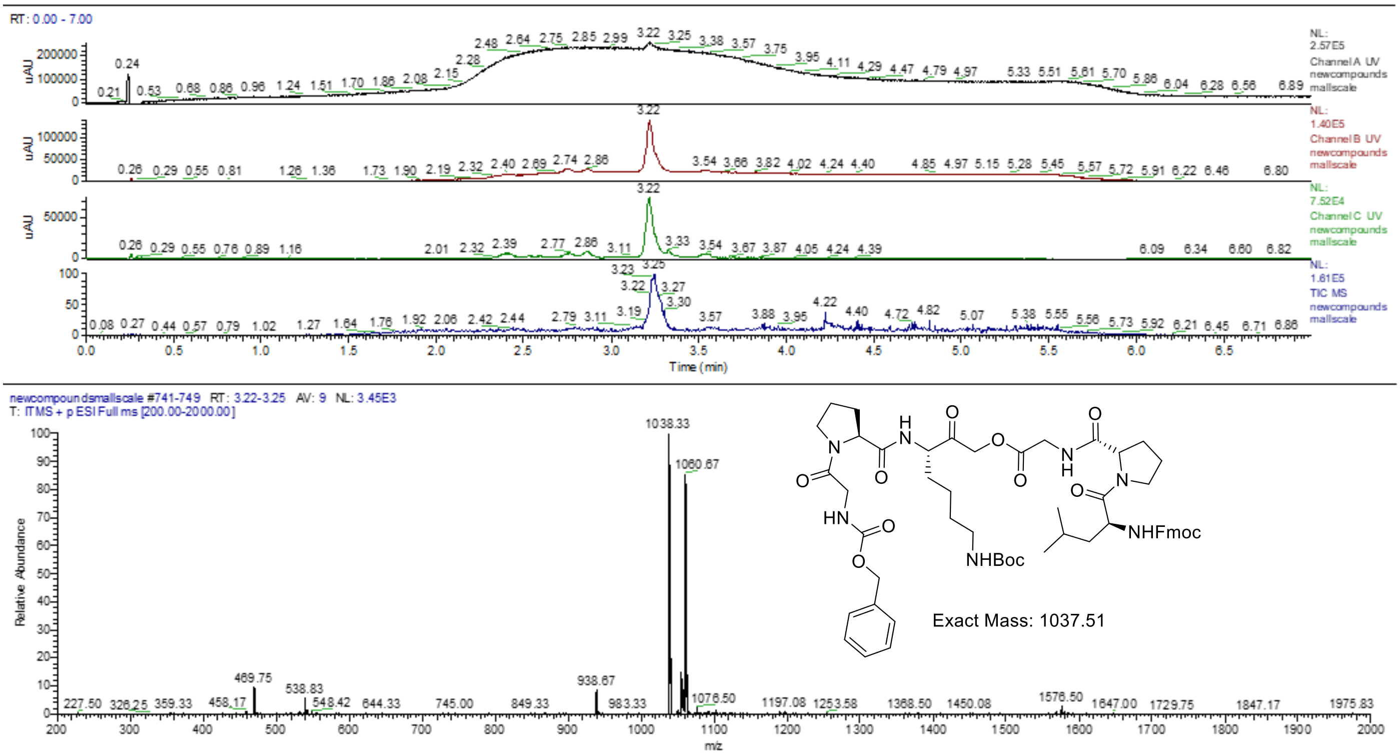

Figure S49. LCMS of Compound **GD25**.

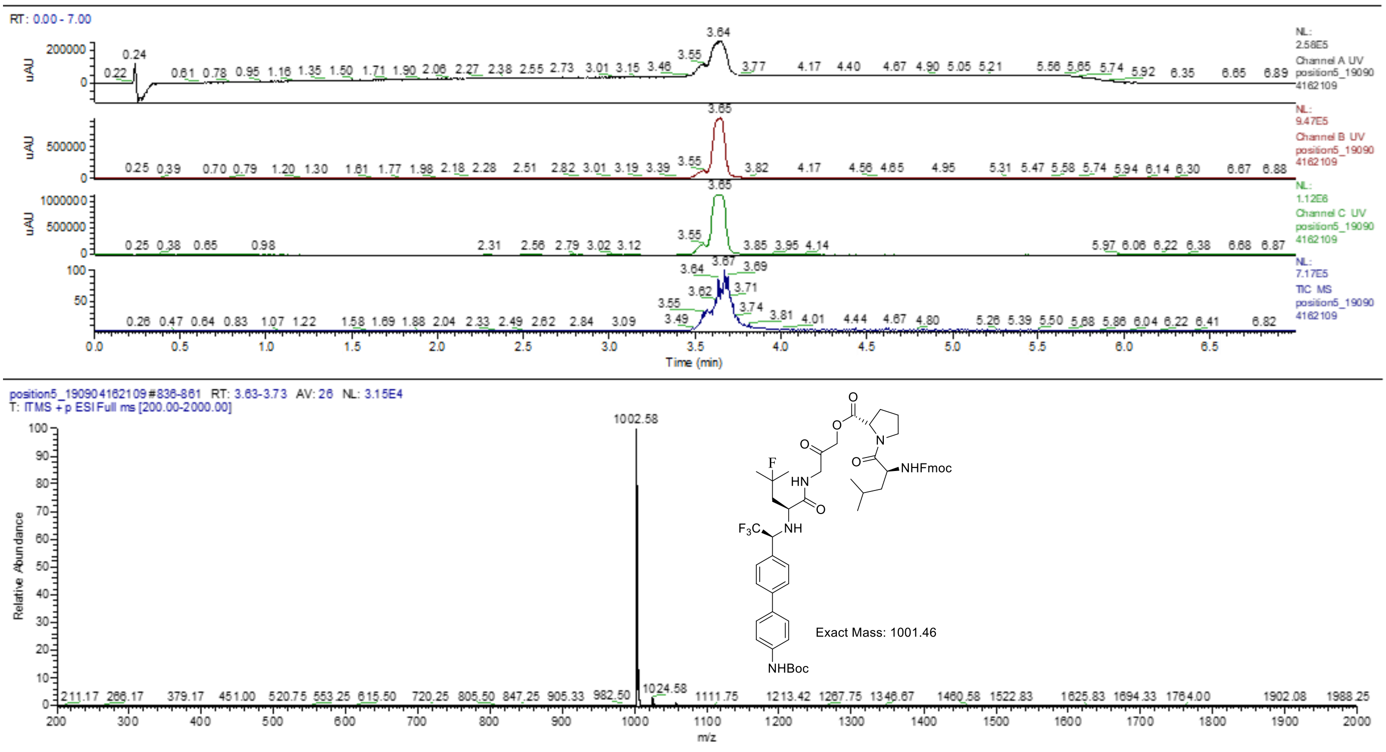

Figure S50. LCMS of Compound **GD26**.

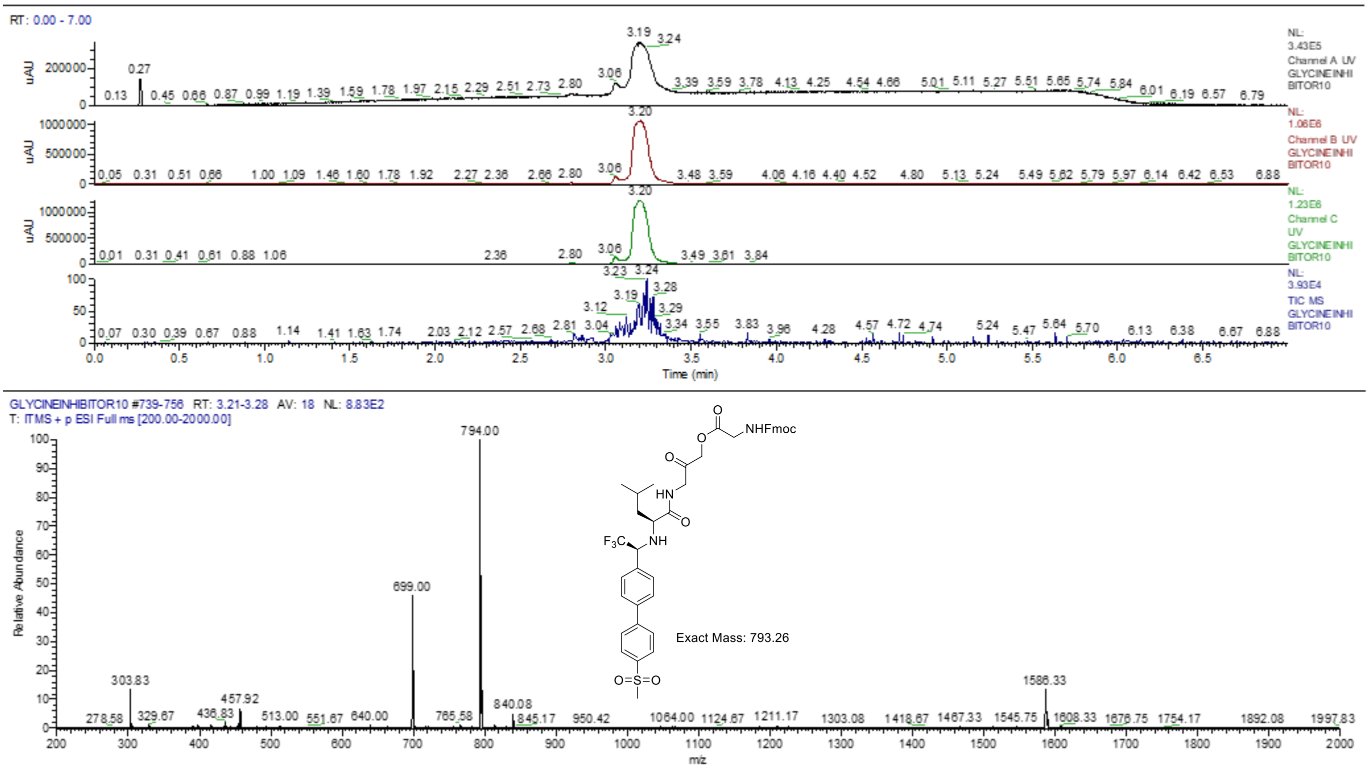

Figure S51. LCMS of Compound **GD27**.

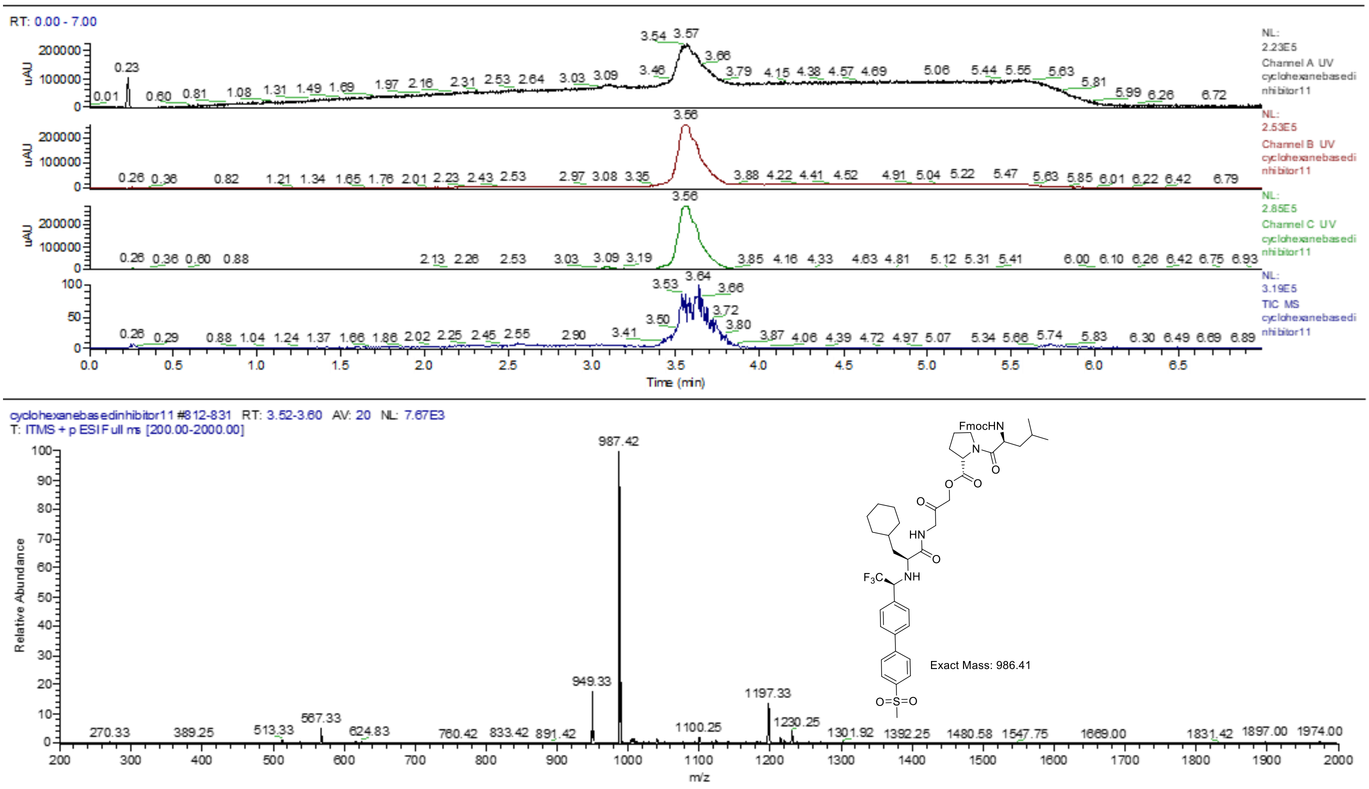

Figure S52. LCMS of Compound **GD28**.

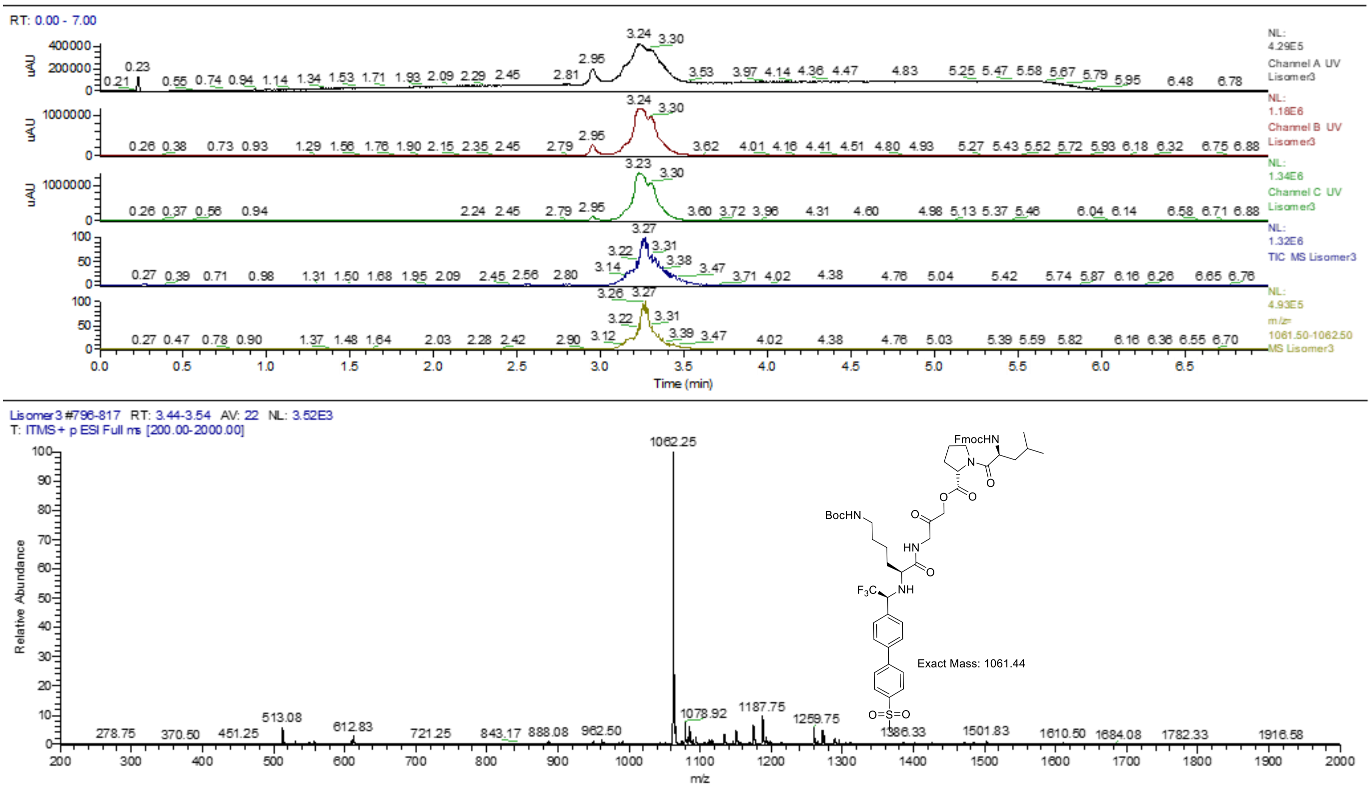

Figure S53. LCMS of Compound **GD29**.

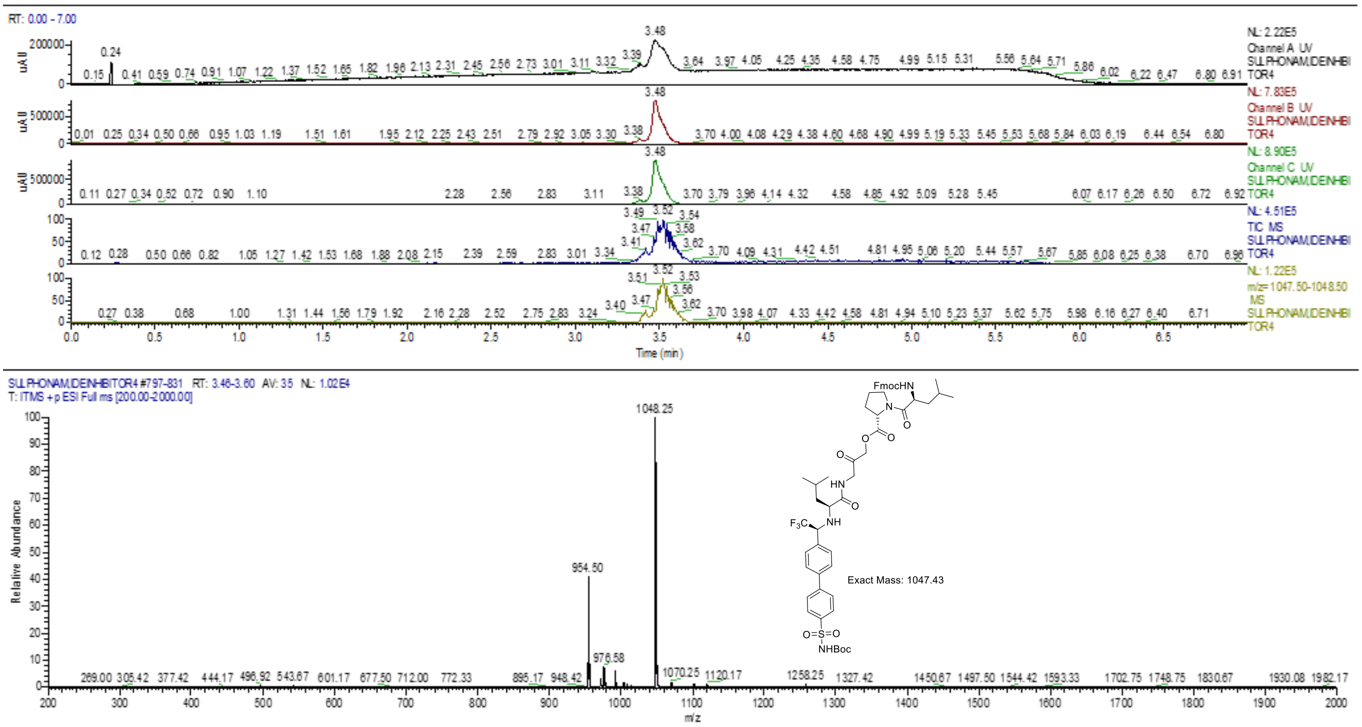

Figure S54. LCMS of Compound **GD31**.

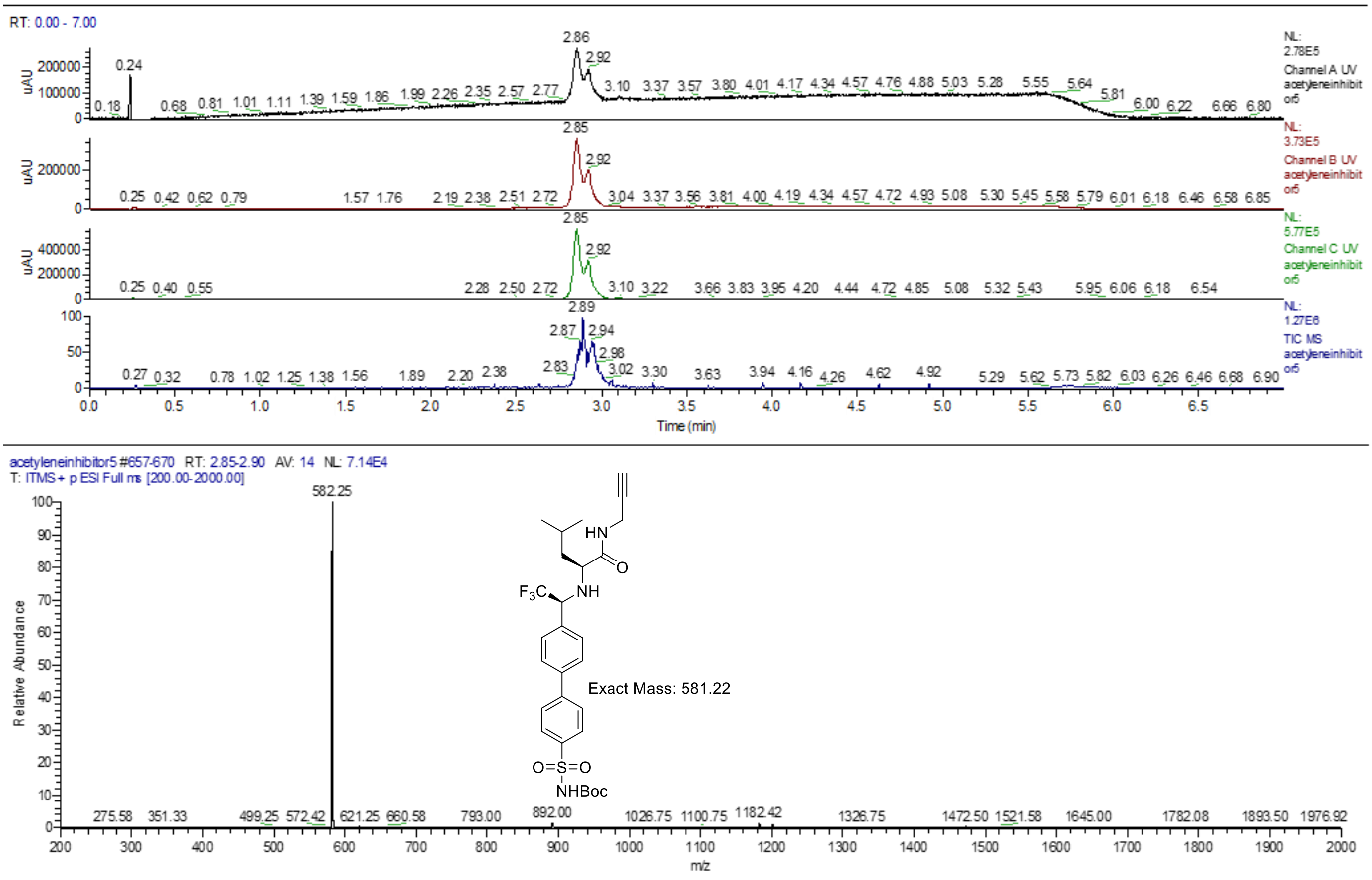

Figure S55. LCMS of Compound **GD33**.

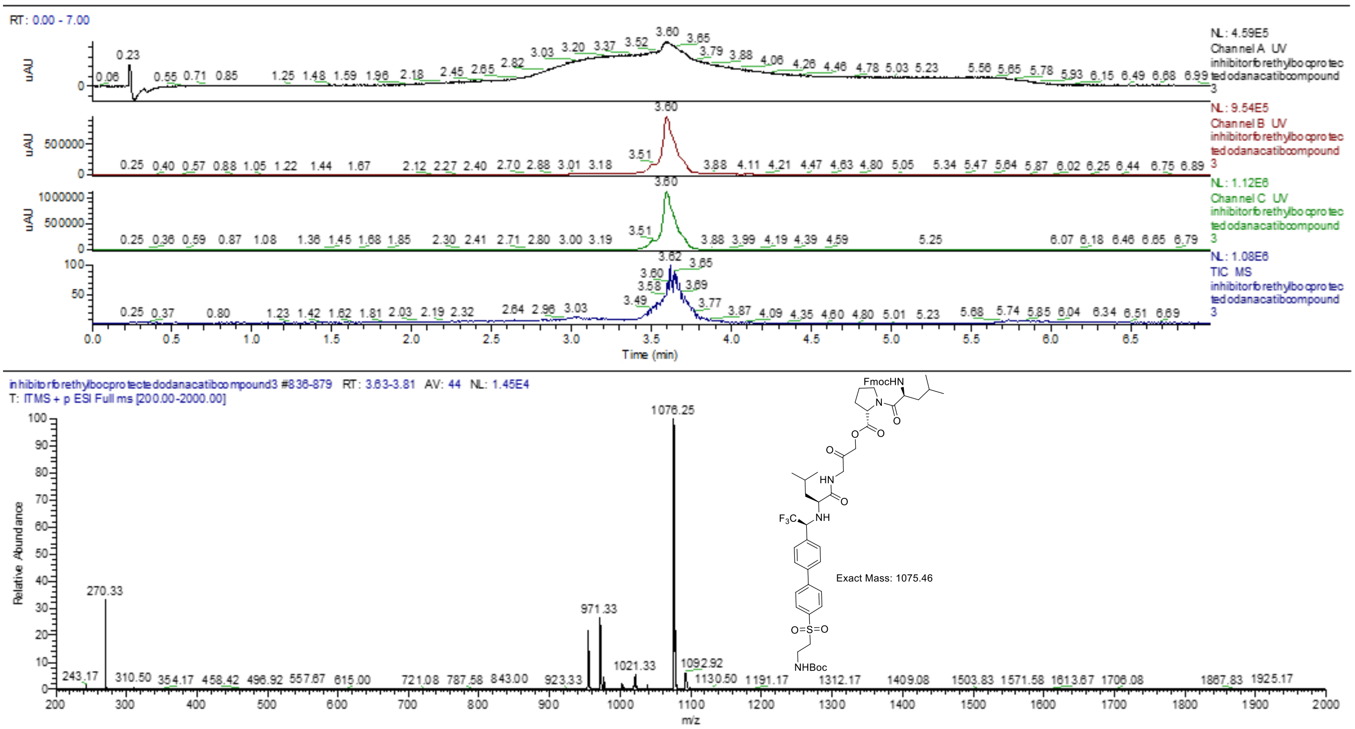

Figure S56. LCMS of Compound **GD34**.

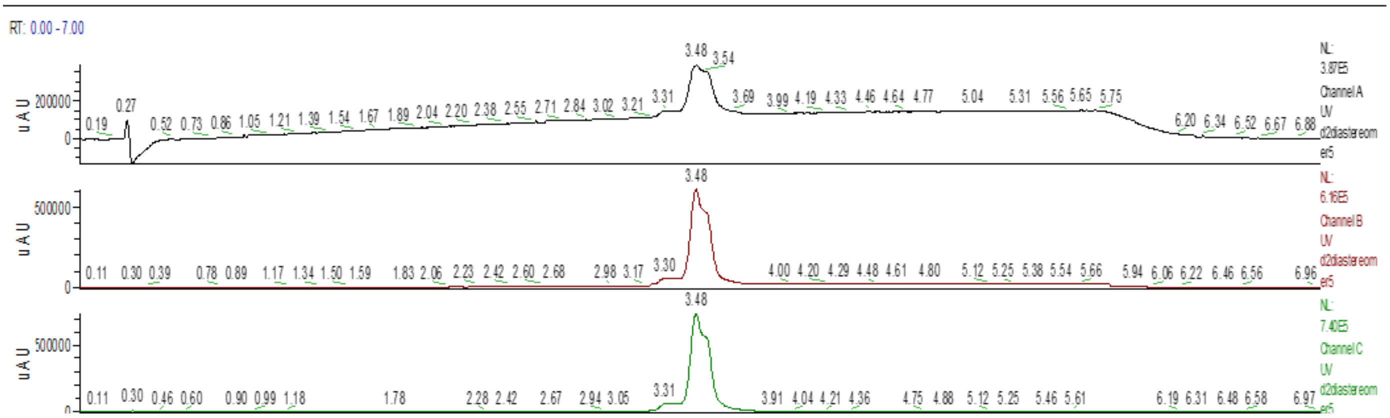

Figure S57. Analytical HPLC grade purity of Compound **GD35**.

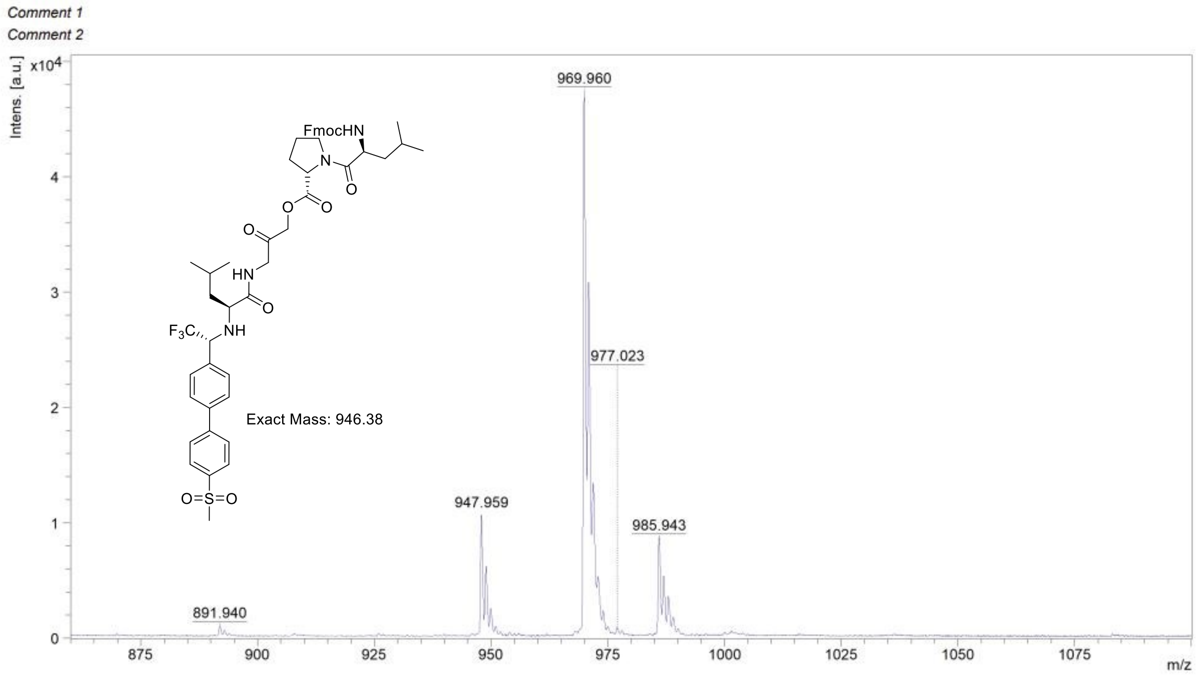

Figure S58. MALDI of Compound **GD35**.

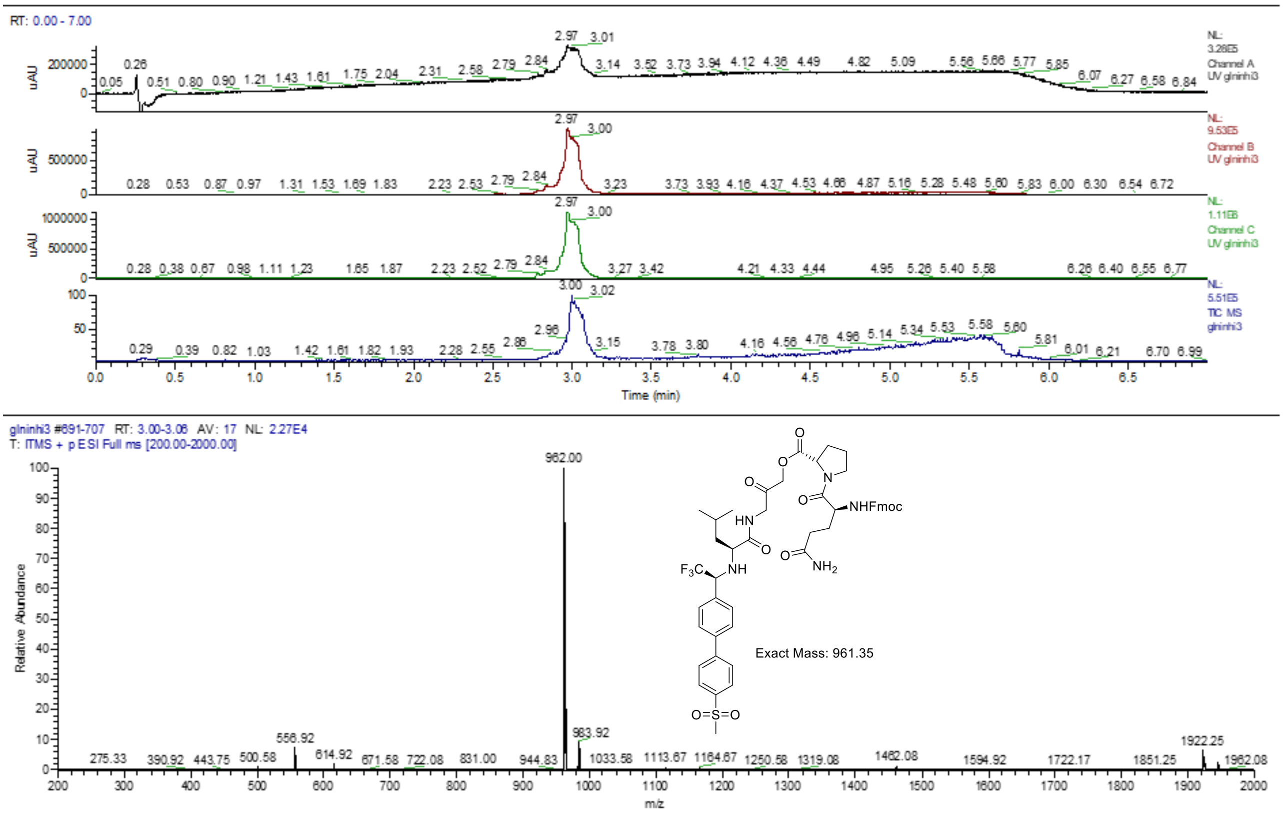

Figure S59. LCMS of Compound **GD38**.

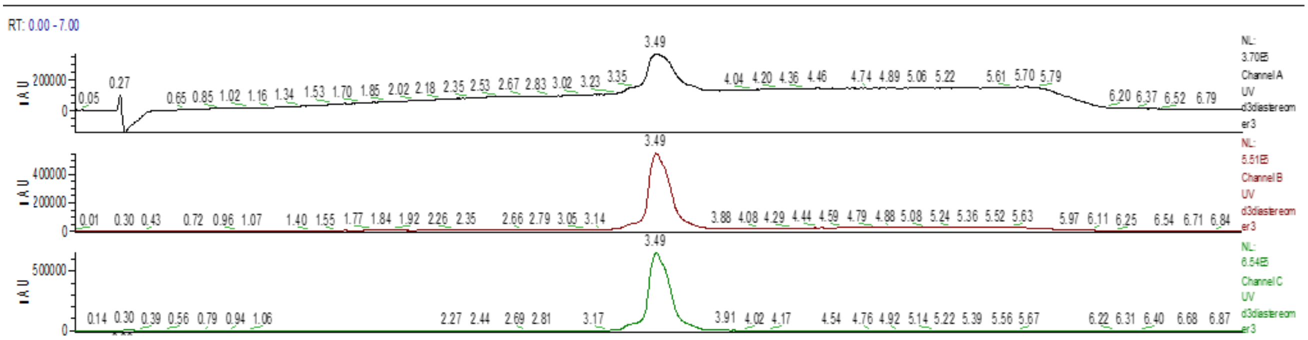

Figure S60. Analytical HPLC grade purity of Compound **GD39.**

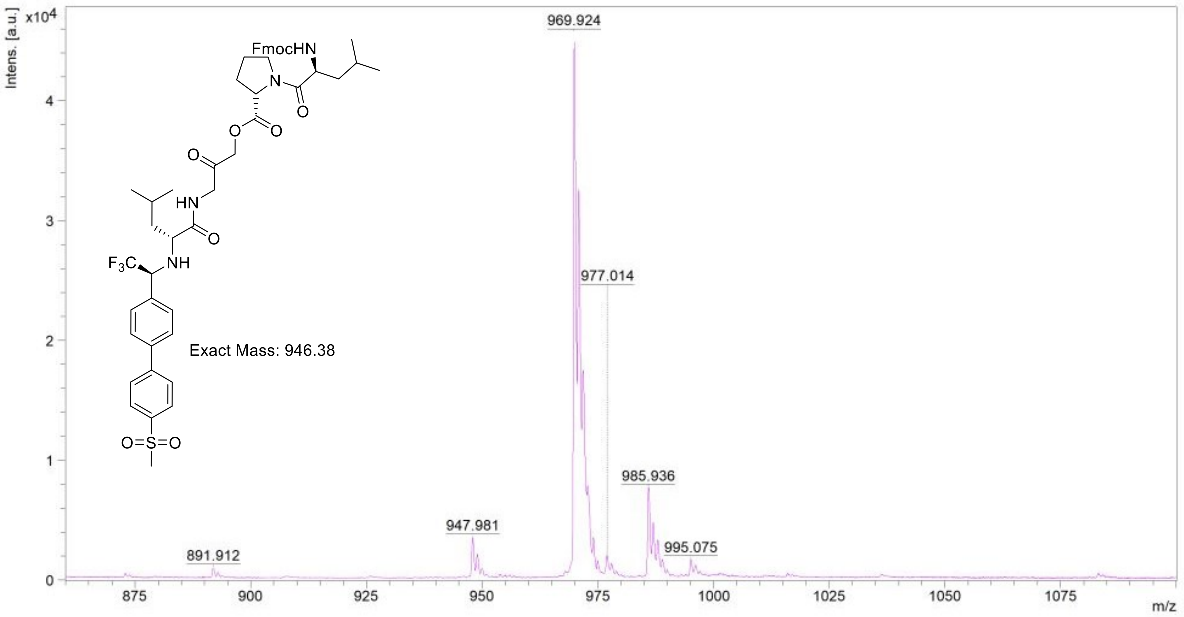

Figure S61. MALDI of Compound **GD39**.

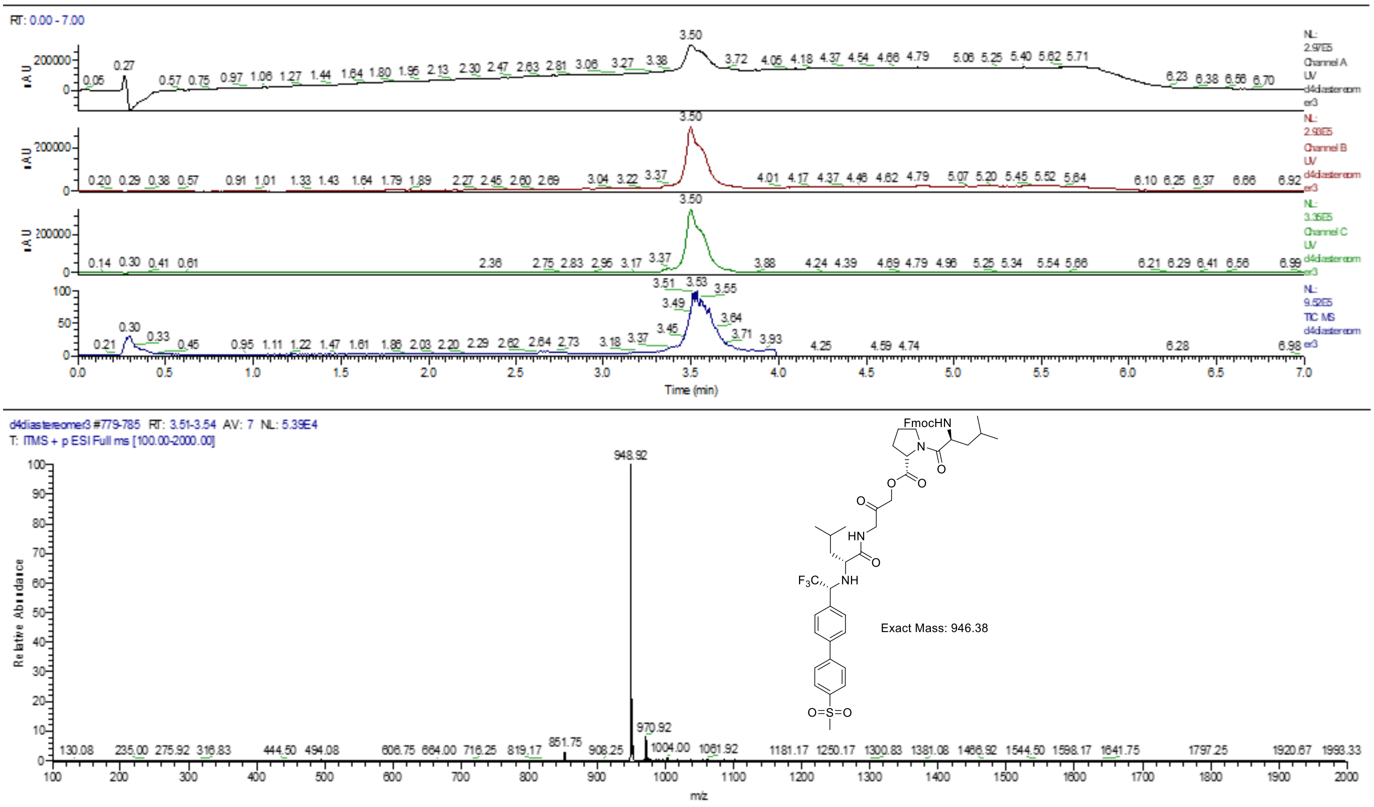

Figure S62. LCMS of Compound **GD40**.

References

1. Mons, E.; Jansen, I. D. C.; Loboda, J.; van Doodewaerd, B. R.; Hermans, J.; Verdoes, M.; van Boeckel, C. A. A.; van Veelen, P. A.; Turk, B.; Turk, D.; Ovaa, H. The Alkyne Moiety as a Latent Electrophile in Irreversible Covalent Small Molecule Inhibitors of Cathepsin K. *J. Am. Chem. Soc.* **2019**, *141* (8), 3507.
2. Lausi, A.; Polentarutti, M.; Onesti, S.; Plaisier, J. R.; Busetto, E.; Bais, G.; Barba, L.; Cassetta, A.; Campi, G.; Lamba, D.; Pifferi, A.; Mande, S. C.; Sarma, D. D.; Sharma, S. M.; Paolucci, G., Status of the crystallography beamlines at Elettra. *Eur. Phys. J. Plus* **2015**,*130(43),* 1-8.
3. Kabsch, W., Integration, scaling, space-group assignment and post-refinement. *Acta Crystallogr., Sect. D* **2010**,*66(2),* 133-144.

4. Winn, M. D.; Ballard, C. C.; Cowtan, K. D.; Dodson, E. J.; Emsley, P.; Evans, P. R.; Keegan, R. M.; Krissinel, E. B.; Leslie, A. G. W.; McCoy, A.; McNicholas, S. J.; Murshudov, G. N.; Pannu, N. S.; Potterton, E. A.; Powell, H. R.; Read, R. J.;Vagin, A.; Wilson, K. S., Overview of the CCP4 suite and current developments. *Acta Crystallogr., Sect. D: Biol. Crystallogr.* **2011**,*67(Pt 4),* 235-242.
5. Turk, D., MAIN software for density averaging, model building, structure refinement and validation. *Acta Crystallogr., Sect. D: Biol. Crystallogr.* **2013**,*69(Pt 8),* 1342-1357.
6. Pražnikar, J.; Turk, D. Free kick instead of cross-validation in maximum-likelihood refinement of macromolecular crystal structures. *Acta Crystallogr., Sect. D: Biol. Crystallogr.* **2014**, *70*, 3124– 3134.
7. Andrejasic, M.; Praznikar, J.; Turk, D., PURY: a database of geometric restraints of hetero compounds for refinement in complexes with macromolecular structures. *Acta Crystallogr., Sect. D* **2008**,*64(11),* 1093-1109
8. Merritt, E. A.; Bacon, D. J., [26] Raster3D: Photorealistic molecular graphics. In Methods in Enzymology, *Academic Press:* **1997**, *277*, 505-524.
9. Ben-Bassat, H.; Vardi, D. V.; Gazit, A.; Klaus, S. N.; Chaouat, M.; Hartzstark, Z.; Levitzki, A. Tyrphostins suppress the growth of psoriatic keratinocytes. *Exp. Dermatol.* **1995**, *4 (2),* 82–88.
